# Anion Binding to Ammonium and Guanidinium Hosts: Implications for the Reverse Hofmeister Effects Induced by Lysine and Arginine Residues

**DOI:** 10.1021/acs.joc.4c00242

**Published:** 2024-04-25

**Authors:** Jacobs H. Jordan, Corinne L.D. Gibb, Thien Tran, Wei Yao, Austin Rose, Joel T. Mague, Michael W. Easson, Bruce C. Gibb

**Affiliations:** † The Southern Regional Research Center, Agricultural Research Service, US Department of Agriculture, 1100 Allen Toussaint Blvd., New Orleans, Louisiana 70124, United States; ‡ Department of Chemistry, Tulane University, New Orleans, Louisiana 70118, United States

## Abstract

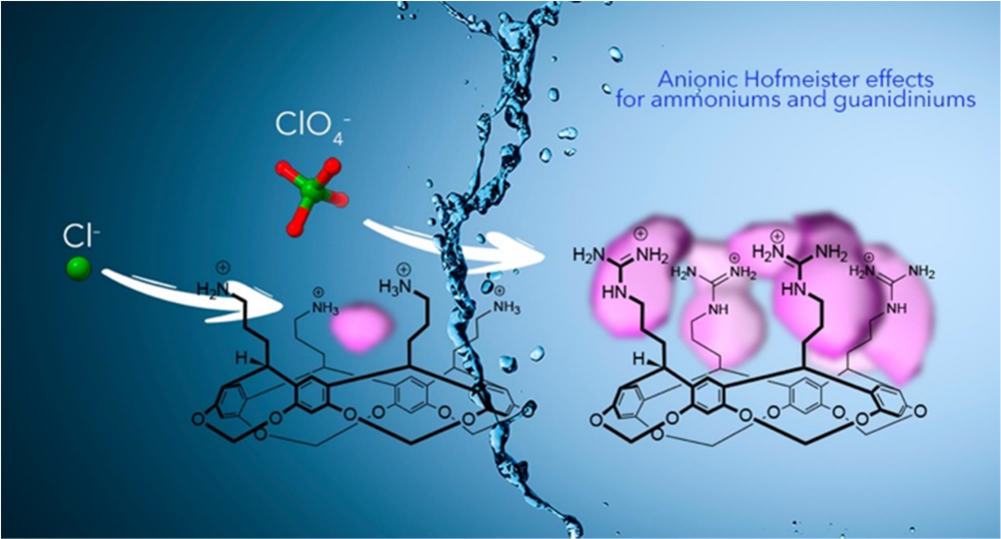

Anions have a profound effect on the properties of soluble proteins. Such Hofmeister effects have implications in biologics stability, protein aggregation, amyloidogenesis, and crystallization. However, the interplay between the important noncovalent interactions (NCIs) responsible for Hofmeister effects is poorly understood. To contribute to improving this state of affairs, we report on the NCIs between anions and ammonium and guanidinium hosts **1** and **2**, and the consequences of these. Specifically, we investigate the properties of cavitands designed to mimic two prime residues for anion-protein NCIs—lysines and arginines—and the solubility consequences of complex formation. Thus, we report NMR and ITC affinity studies, X-ray analysis, MD simulations, and anion-induced critical precipitation concentrations. Our findings emphasize the multitude of NCIs that guanidiniums can form and how this repertoire qualitatively surpasses that of ammoniums. Additionally, our studies demonstrate the ease by which anions can dispense with a fraction of their hydration-shell waters, rearrange those that remain, and form direct NCIs with the hosts. This raises many questions concerning how solvent shell plasticity varies as a function of anion, how the energetics of this impact the different NCIs between anions and ammoniums/guanidiniums, and how this affects the aggregation of solutes at high anion concentrations.

## Introduction

Although in biochemistry the ammonium and guanidinium side chains of lysine and arginine are often treated as chemically synonymous, these cations have fundamentally different supramolecular repertoires.^1,2^ Thus, a guanidinium ion can form attractive Coulombic interactions,^1^ counterintuitive cation pairing,^2−15^ edge-to-face hydrogen bonds (HBs),^1,4^ and van der Waals (VdW) interactions with its pair of faces.^16^ It is not inappropriate to describe the guanidinium ion as an orientational amphiphile.^17^ In contrast, the supramolecular repertoire of an ammonium group is narrower; it can only form classic Coulombic interactions and, if it possesses one or more free N–H groups, monodentate HBs.

These differences in the supramolecular repertoire apparently have many ramifications. Thus, the complex amphiphilicity and/or cation pairing of guanidiniums are likely behind its unusual solvation,^8−10,18−20^ its complex denaturation properties,^21−23^ why poly arginines self-associate but poly lysines do not,^4,24^ the affinity of guanidiniums for phospholipid bilayers,^25^ and the extraordinary ability of arginine-rich polypeptides to passively penetrate across cellular membranes,^26−36^ (so-called “arginine magic”^28^). The complex amphiphilicity of guanidinium also likely explains the ability of arginine side chains^37,38^ to induce liquid–liquid phase separation,^39,40^ and contribute to the stability of membraneless organelles.^41^ This broad supramolecular repertoire of guanidiniums is, however, a double-edged sword. For example, although both ammoniums and guanidiniums have been used extensively by the supramolecular community for anion binding,^1,42−44^ membrane transport,^45^ and sensing,^46−48^ the balance of research lies with ammoniums because they do not possess the handling difficulties of “sticky” guanidiniums.

Beyond anion binding, sensing, and transport with synthetic hosts, the supramolecular differences between ammonium and guanidinium derivatives are key to the histone code.^49−52^ Moreover, arginine, lysine, and histidine residues are also instrumental to how anions in buffer and salt solutions affect biologics stability,^53,54^ protein aggregation,^55^ amyloidogenesis,^56−66^ crystallization,^67−70^ and more generally, Hofmeister effects.^71−73^ On this last point, anion binding to lysine, arginine, and histidine is particularly important in the reverse (or inverse) Hofmeister effect, whereby charge-diffuse anions induce the aggregation and precipitation of proteins at pH values below their isoelectric point (pI).^74−83^

A previous study in our lab examined the reverse Hofmeister effect in a cavitand possessing eight *tetra*-alkylammonium groups.^84^ This work showed that anions preferentially bound to one of its two host pockets: a classically “hydrophobic pocket” composed of aromatic rings and a “crown” of four *tetra*-alkylammoniums formed by the pendent groups of the host. Higher concentrations of anions, particularly charge-diffuse ones, induced the reverse Hofmeister effect and precipitated the host. However, in cases where the anion preferentially bound to the hydrophobic pocket, the ability of the anion to induce precipitation could be attenuated when the pocket was occupied by a guest molecule.

**Scheme 1:**
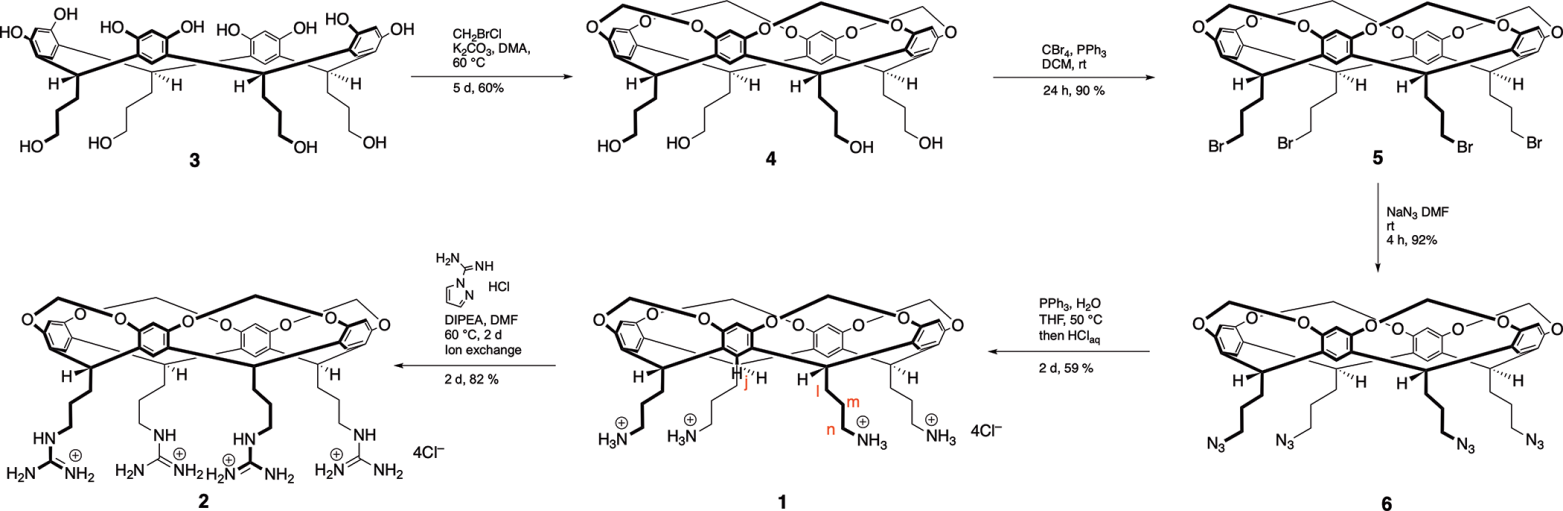
Synthesis of Cavitands **1** and **2** The reporter atoms used in ^1^H NMR titration experiments H_j_, H_l,_ and H_n_, are highlighted in red in the former. Synthetic details are provided in the text and Supporting Information.

As part of our ongoing studies of Hofmeister effects, we have explored simpler cavitands possessing ostensibly only one anion-binding site. Such hosts have allowed a detailed understanding of the role of buffer in controlling anion guest affinity and helped answer the question as to whether or not screening effects should be built into affinity models.^85^ Building on this work, we envisioned that similar hosts may shed light on the aqueous supramolecular properties of ammoniums and guanidiniums and hence improve our understanding of how these positively charged groups interact with anions and induce Hofmeister effects. Considering that lysine and arginine account for ∼85% of all positively charged residues in eukaryotic systems, we therefore targeted novel hosts **1** and **2** possessing ammonium and guanidinium groups, respectively (Scheme 1). By mimicking lysine and arginine clusters in the surface of proteins, we envisioned that the presence of four charges would enhance anion binding such that the structure of different complexes and their thermodynamics of formation could be, respectively, qualified and quantified. Thus, we present here the synthesis of novel *tetra*-ammonium and *tetra*-guanidinium cavitands **1** and **2**, and their affinity for a range of anions using both ^1^H NMR and isothermal titration calorimetry (ITC). Additionally, we present X-ray crystallographic analysis of select complexes as well as molecular dynamics (MD) simulations examining the localization of anions to the hosts. Finally, we consider the consequences of anion binding and present critical precipitation concentrations (CPCs), i.e., the concentration of the anion required to induce precipitation of the hosts within a set time frame. Taken together, these studies reveal new details about the intrinsic supramolecular properties of ammonium and guanidinium complexes. We anticipate that these findings will be of import to the design of aqueous-based receptors and, more generally, to studies of Hofmeister effects.

## Host Synthesis

The syntheses of hosts **1** and **2** are shown in Scheme 1 (see Supporting Information, Sections 1–3 for full information). As previously described,^85^ resorcinarene **3** was available in 95% yield by the acid-catalyzed condensation of resorcinol and 2,3-dihydrofuran.^86^ Building from this, resorcinarene **3** was bridged with bromochloromethane in 60% yield to yield cavitand **4**.^86^ An Appel reaction upon **4** then gave tetrabromide **5** in 90% yield.^87^ Subsequently, treatment with sodium azide gave tetra-azide **6** in 92% yield, which was then converted to *tetra*-ammonium salt **1** in 59% yield via Staudinger reduction^88^ and treatment with excess HCl_aq_. Cavitand **1** could then be converted to the *tetra*-guanidinium **2** by treatment with 1*H*-pyrazole-1-carboxamidine hydrochloride.^89^ Here, product isolation took advantage of the reverse Hofmeister effect and the poor solubility of perchlorates in aqueous media. Thus, the addition of excess sodium perchlorate to the crude reaction product gave a precipitate of the guanidinium perchlorate.^84^ Ion exchange then gave the desired chloride salt **2** in an 82% yield.

## Results

Based on earlier work,^84^ we surmised that anions would bind to the crown of ammonium (guanidinium) groups formed by the pendent groups of host **1** (**2**), and to investigate this possibility, we first turned to affinity determinations by NMR spectroscopy.

### Anion Affinity by ^1^H NMR

**Figure 1:**
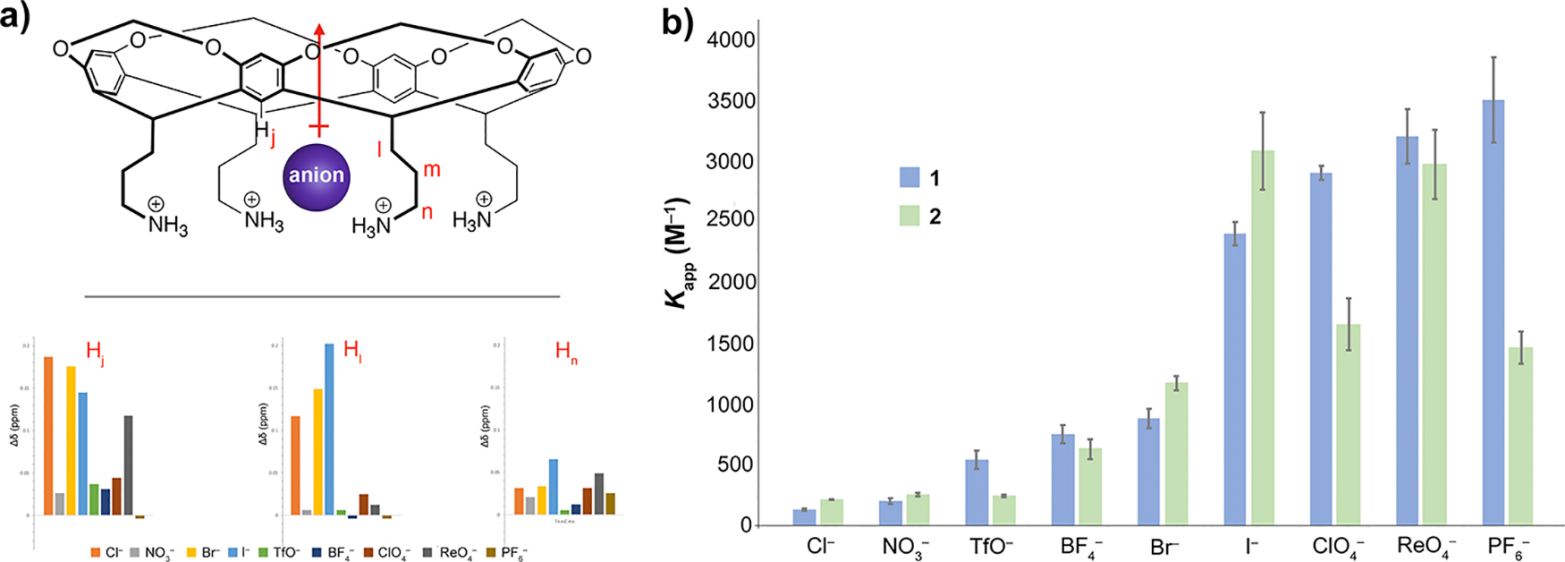
(a) Representation of the host **1** anion complex as judged by ^1^H NMR host signal shifts (bar graphs) for reporter atoms H_j_, H_l_, and H_n_. Errors in signal shifts are ±0.05 ppm. The indicated anions are ordered with increasing affinity. (b) Apparent anion affinity constants (*K*_app_) for hosts **1** and **2**. All solutions were unbuffered but remained at pH 5.2 and 5.9 ± 0.1 during titration of the *tetra*-ammonium **1** and *tetra*-guanidinium **2**, respectively. Shown error bars correspond to experimental errors from the triplication of data.

We determined the affinity of anions for the charged crowns of **1** and **2** by using ^1^H NMR titration experiments (all experiments in unbuffered D_2_O). In doing so, we determined Δδ_max_, the theoretical maximal shift of a signal at an infinite salt concentration where the host is fully complexed. These signal shifts in hosts **1** and **2** were illuminating. The trends in the data for either host were similar. Figure 1a shows data for **1** (see Supporting Information Section 4A, Figure S36 for the data for **2**). Thus, the largest shifts, Δδ_max_ ∼ 0.20 ppm, were observed for host atoms H_j_ and H_l_ (Figure 1a). The H_n_ atoms underwent much smaller shifts (Δδ_max_ ≅ 0.07 ppm), while the shifts of H_m_ were negligible. Focusing on the H_j_ and H_l_ signals, the monatomic anions induced much larger downfield shifts than those for the different polyatomic anions. Moreover, for the aromatic H_j_ reporter atoms, the downfield shifts induced by the halides decreased as the electronegativity decreased, whereas the reverse was true for the α-methylenes adjacent to the resorcinarene bowl (H_l_). This difference between the effects of monatomic and polyatomic anions was negligible for the weaker reporter H_n_.

Using the corresponding sodium salts, we determined the affinity of Cl^–^, Br^–^, I^–^, NO_3_^–^, BF_4_^–^, TfO^–^, ClO_4_^–^, ReO_4_^–^, and PF_6_^–^ (Figure 1b, see Supporting Information Section 4B for full details). In each case, we treated the four Cl^–^ counteranions of the host as nonbinding spectators. Strictly speaking, these are in competition for the pocket of the host,^85^ and correspondingly, we report here the apparent affinity constant (*K*_app_). All titration data fitted 1:1 binding; there was no evidence of a 1:2 guest complexation. *En masse*, this data reveals that smaller anions such as the halides and nitrate had a slight preference for guanidinium **2**, whereas the polyatomic anions were preferentially bound to ammonium **1**. Within this group of guests, some interesting selectivities were observed. The greatest differences in affinity were seen for ClO_4_^–^ and for PF_6_^–^. These anions bind 1.5 and 2.2 kJ mol^–1^ more strongly to ammonium host **1**. In contrast, within error, BF_4_^–^ and ReO_4_^–^ bound with equal affinity to both **1** and **2**, whereas in the case of NO_3_^–^, there was a small but significant preference for host **2**.

### ITC Analysis of Anion Binding

**Figure 2:**
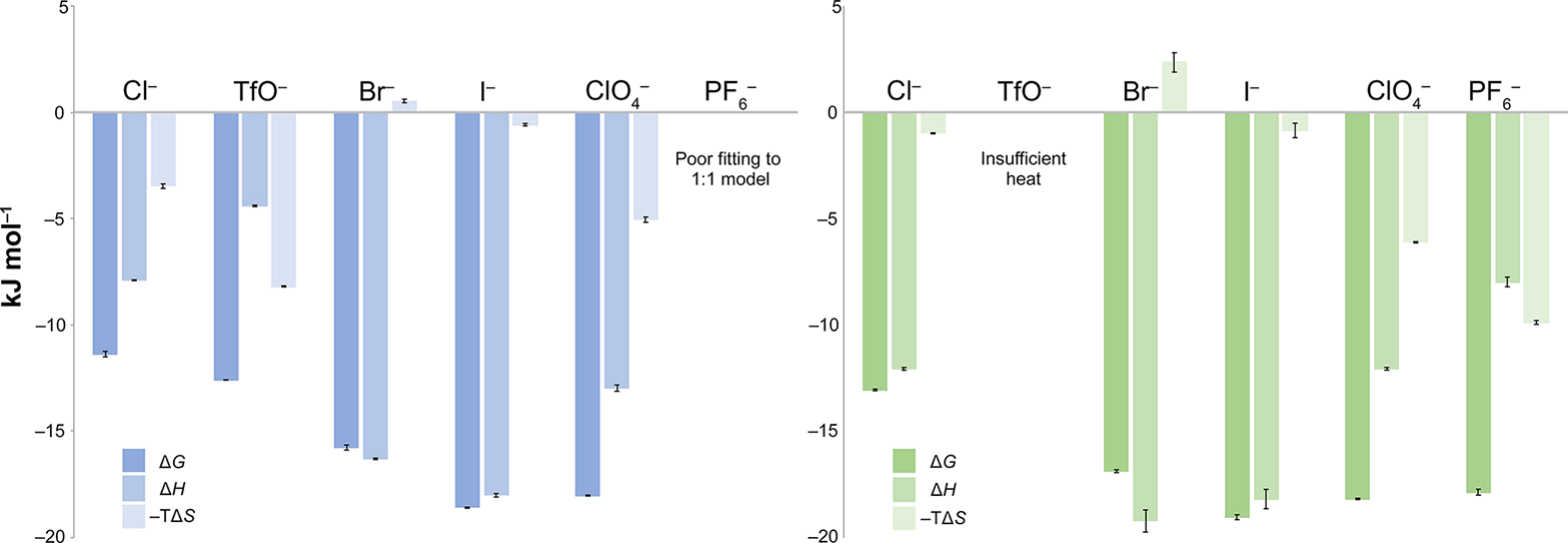
Thermodynamic parameters for anion binding to *tetra*-ammonium **1** (blue) and *tetra*-guanidinium **2** (green). All solutions were 10 mM phosphate buffer in 18.2 MΩ cm^–1^ H_2_O, at pH 3.0 ± 0.1. Shown error bars correspond to experimental errors from the triplication of data.

To gain more thermodynamic information about anion binding, we performed ITC experiments. For this work, we selected the sodium salts of Cl^–^, Br^–^, I^–^, TfO^–^, ClO_4_^–^, ReO_4_^–^, and PF_6_^–^ to ensure a spread of charge-diffusivity among the guests. However, under the buffer conditions selected, ReO_4_^−^ induced precipitation. This anion was therefore not studied by ITC. The thermodynamic parameters (Δ*G*, Δ*H*, and −*T*Δ*S*) of anion binding are presented in Figure 2 (see Supporting Information, Section 4C for full details). Because of the use of phosphate buffer in these studies, anion affinity as measured by ITC was slightly weaker than that measured by ^1^H NMR because of phosphate competition for the central binding site.^85^

Two host–guest combinations failed to provide the data. The binding of PF_6_^–^ to host **1** did not fit a 1:1 model (or higher models), indicating some degree of aggregation during the titration experiment. In contrast, the binding of TfO^–^ to host **2** failed to produce sufficient levels of heat for reliable data, suggesting that the association was largely driven by entropy. The weak association is perhaps not surprising considering this was the next weakest binding event, as determined by NMR (Figure 1).

In the remaining cases, all binding events were exothermic. The least exothermic binding was observed for TfO^–^ to host **1** (Δ*H* = −4.4 kJ mol^–1^). In contrast, binding of Br^–^ to host **2** was found to be the most exothermic (−19.2 kJ mol^–1^). Iodide binding to this host was only slightly less exothermic (−18.2 kJ mol^–1^), but Cl^–^ bound with a considerably lower exothermicity of −12.1 kJ mol^–1^. Remaining with the halides, a different ordering of the enthalpies of binding was evident for host **1**. Here I^–^ complexation was the most exothermic and Cl^–^ again the least: −18.0, −16.3, and −7.9 kJ mol^–1^ for I^–^, Br^–^, and Cl^–^, respectively. The polyatomic anions TfO^–^, ClO_4_^–^, and PF_6_^–^ liberated less heat upon binding than the halides. Thus, ClO_4_^–^, the polyatomic anion binding with the greatest exothermicity (Δ*H* = −13.0 and −12.1 kJ mol^–1^ for **1** and **2**, respectively), surpassed only Cl^–^ in the liberation of heat when binding to **1**. Rather, the higher binding constants of the polyatomic anions arose because they were relatively strongly promoted by entropy. Indeed, in the case of TfO^–^ binding to **1** and PF_6_^–^ binding to **2**, the contribution to binding from entropy outweighed that from enthalpy (Δ*H* = −4.4, −*T*Δ*S* = −8.2 kJ mol^–1^, and Δ*H* = −8.0, −*T*Δ*S* = −9.9 kJ mol^–1^, respectively). Even in the case of enthalpy-dominated ClO_4_^–^, the entropy contribution to complexation was stronger than any of the halides, especially in the case of **2**.

### X-ray Structures of Selected Complexes

We turned to X-ray crystallography to gain structural insight into the complexes that were formed. All host–guest combinations were screened, resulting in four suitable crystals (see Supporting Information, Section S4E). Thus, we obtained the structures of the Cl^–^, Br^–^, and ClO_4_^–^ complexes of host **1** and the ClO_4_^–^ complex of host **2** by the addition of excess sodium salt and slow evaporation. In all cases, it was not possible to identify specific locations for the sodium cations.

**Figure 3:**
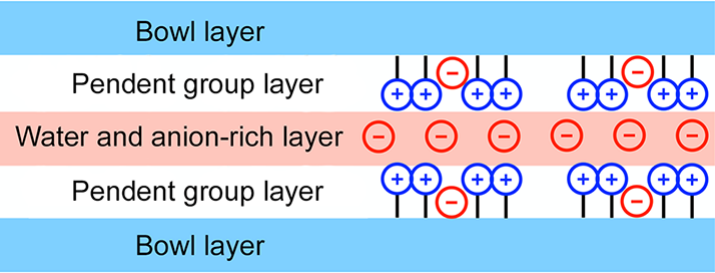
Schematic of the general layered form of the X-ray structures obtained. The anion distribution shown is for visualization purposes only.

**Figure 4:**
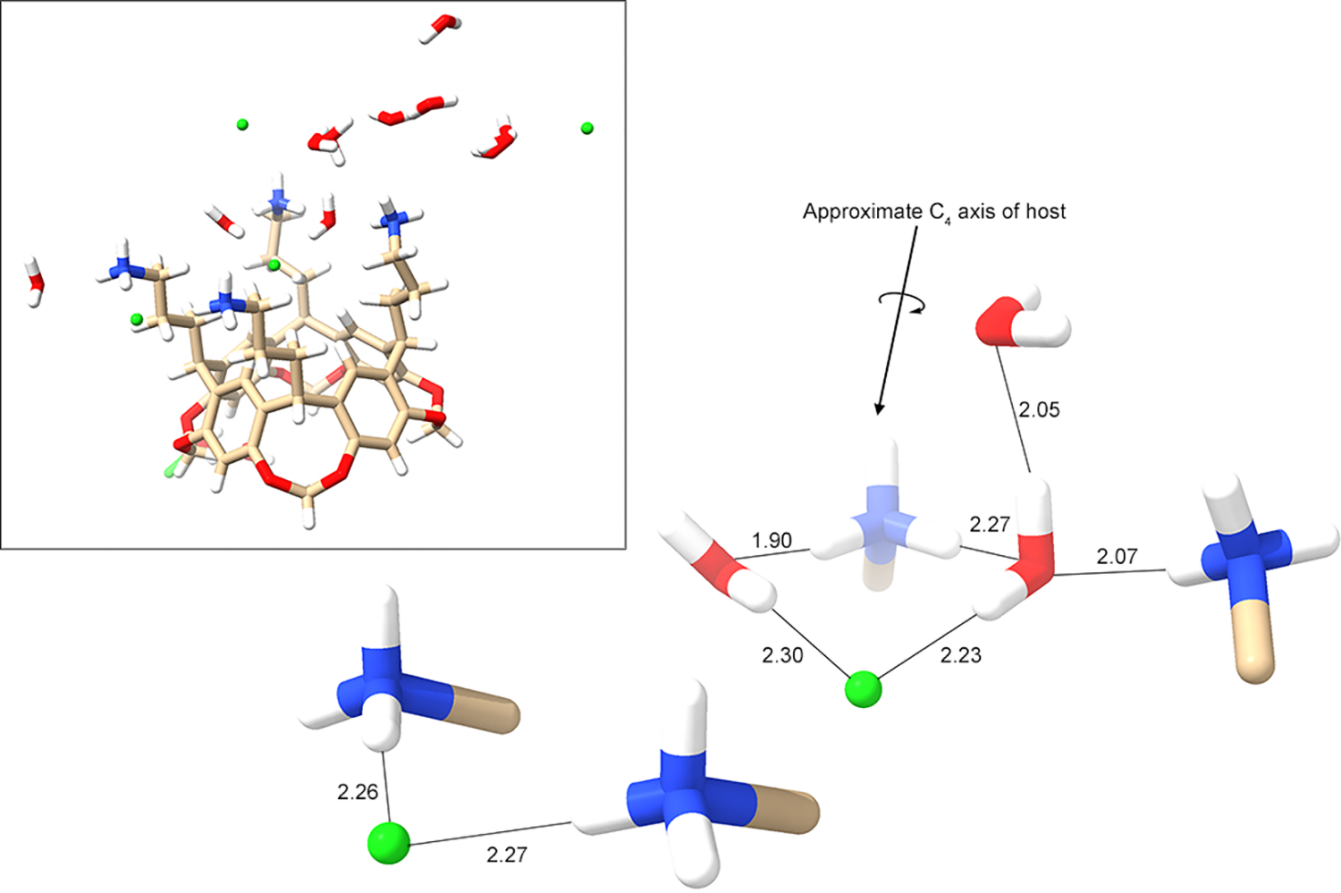
Core region of the unit cell of the complex between **1** and Cl^–^, showing the bound guest (*lower center-right*), the four ammoniums and their α-carbon atoms of the pendent groups, and their local waters and (second) Cl^–^ atom. Ordered and disordered water and Cl^–^ in the water and ion-rich layer (Figure 3) have been omitted for clarity, as have two waters and a disordered chloride associated with the rim of the cavitand bowl. For orientation, the same perspective of the complete unit cell is shown (*insert*). Interatomic distances are highlighted, revealing the bridging of two ammoniums by a Cl^–^ atom (*left*) and the key HB network that contributes to the stabilization of the host–guest complex.

**Figure 5:**
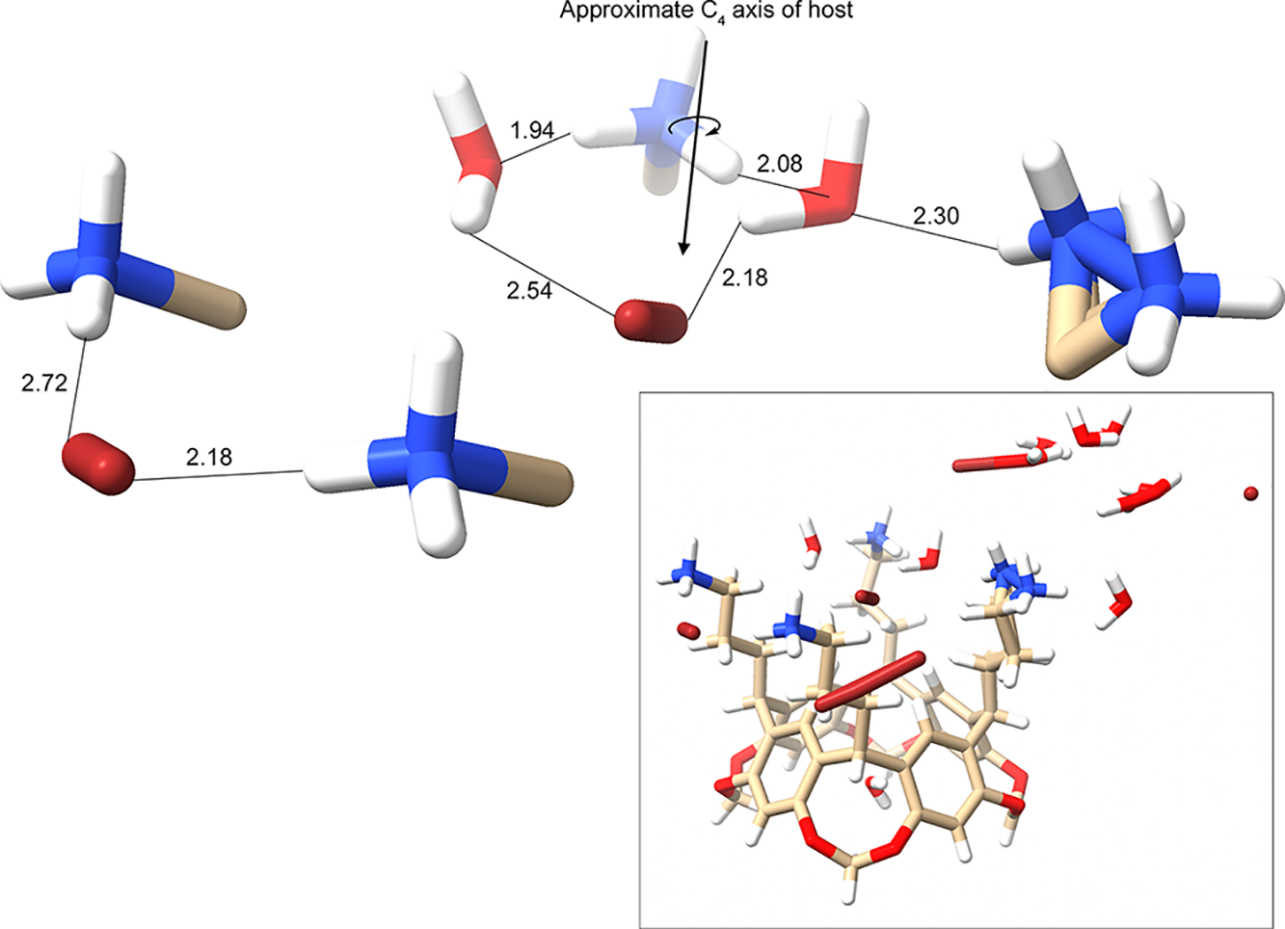
Core region of the unit cell of the complex between **1** and Br^–^, showing the bound disordered guest (*upper center*), the four pendent ammoniums and their α-carbon atoms (of the pendent groups), their local waters, and a second Br^–^ atom bridging one pair of ammonium groups (*center left*). Ordered and disordered water and Br^–^ in the water and ion-rich layer (Figure 3) have been omitted for clarity, as has ordered water inside the cavitand aromatic bowl. For orientation, the same perspective of the complete unit cell is shown (*insert*). The noted interatomic distances involving the disordered Br^–^ atoms are the average of the two extreme bromide positions. In the case of the HB between the water and the disordered ammonium, because of the significance of the disorder, the distance given is the shortest possible interatomic separation.

**Figure 6:**
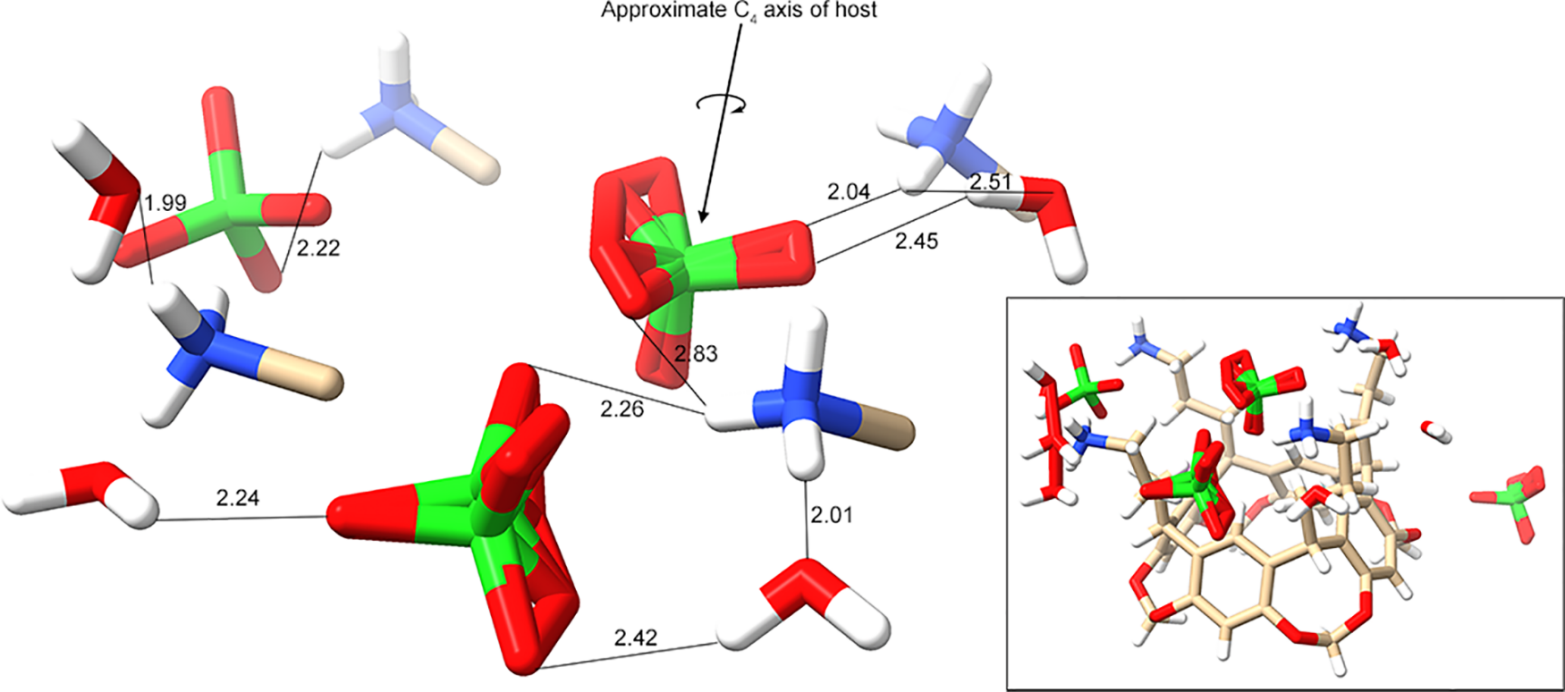
Core region of the unit cell of the complex between **1** and ClO_4_^–^ showing the bound disordered guest (*upper center*), the four ammoniums and their α-carbon atoms (of the pendent groups), and their local waters and ClO_4_^–^ ions. One of the waters (*lower left*) is disordered (76% occupancy shown), as is one of the peripheral ClO_4_^–^ ion (*front center*). Ordered and disordered waters in the water and ion-rich layer (Figure 3) have been omitted for clarity, as has a disordered ClO_4_^–^ associated with the outside of the cavitand aromatic bowl. For orientation, the same perspective of the complete unit cell is shown (*insert*). Key interatomic distances are highlighted, showing the network of noncovalent interactions in the host–guest complex. Where disorder is present, the noted distances correspond to the atomic position that results in the shortest possible interatomic separation.

**Figure 7:**
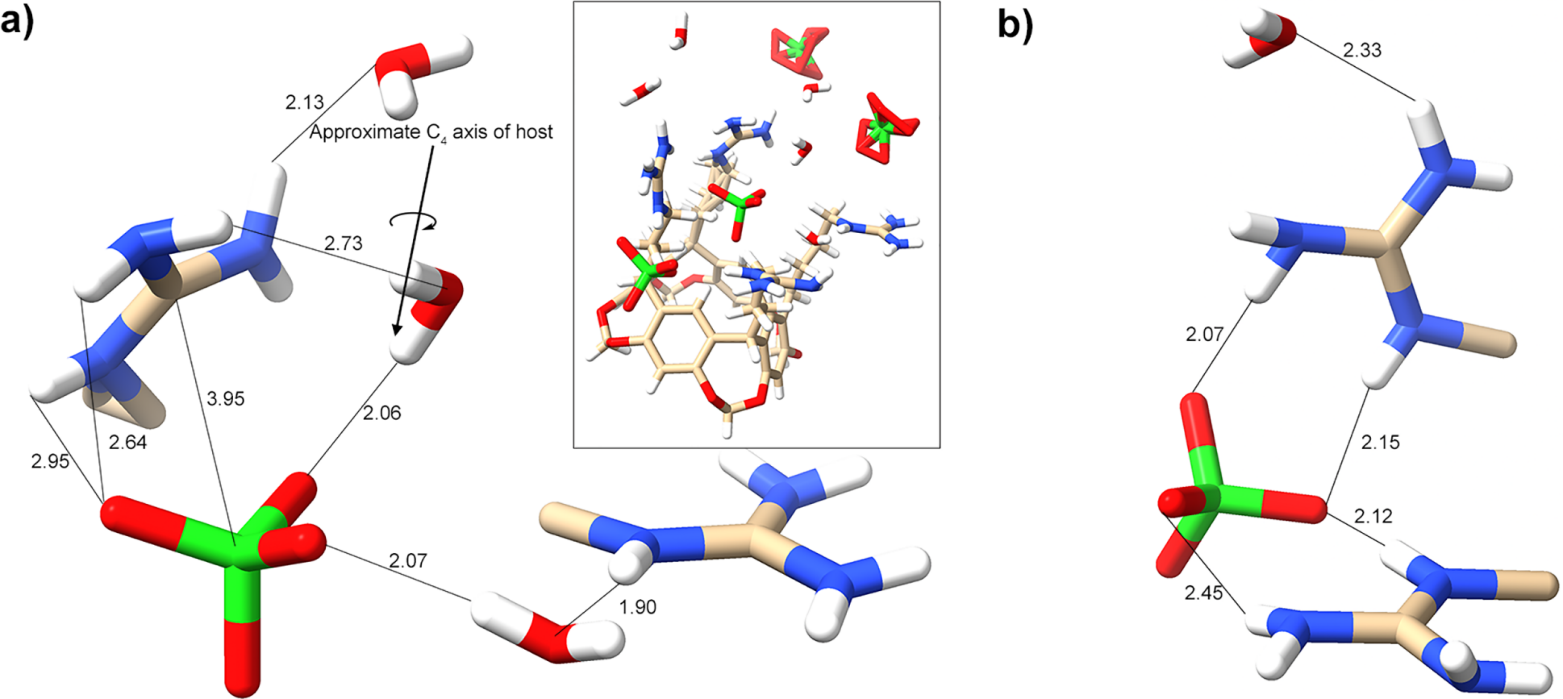
(a) Core region of the unit cell of the complex between **2** and ClO_4_^–^ showing the bound guest (*lower left*), the two proximal guanidiniums and their α-carbon atoms of the pendent groups and their local waters. Both local waters form short HBs to the guest. Ordered and disordered waters and ions in the water and ion-rich layer (Figure 3) have been omitted for clarity, as has a disordered perchlorate associated with the outside of the cavitand bowl. For orientation, the same perspective of the complete unit cell is shown (*insert*). Key interatomic distances are highlighted, showing the network of noncovalent interactions in the host–guest complex. (b) Secondary ClO_4_^–^ binding site showing the four HBs between the pair of guanidinium groups and the guest. In this representation, the cavitand bowl (not shown) is positioned below and to the right of the shown atoms. Figure 11 shows the two binding sites relative to one another.

**Figure 8:**
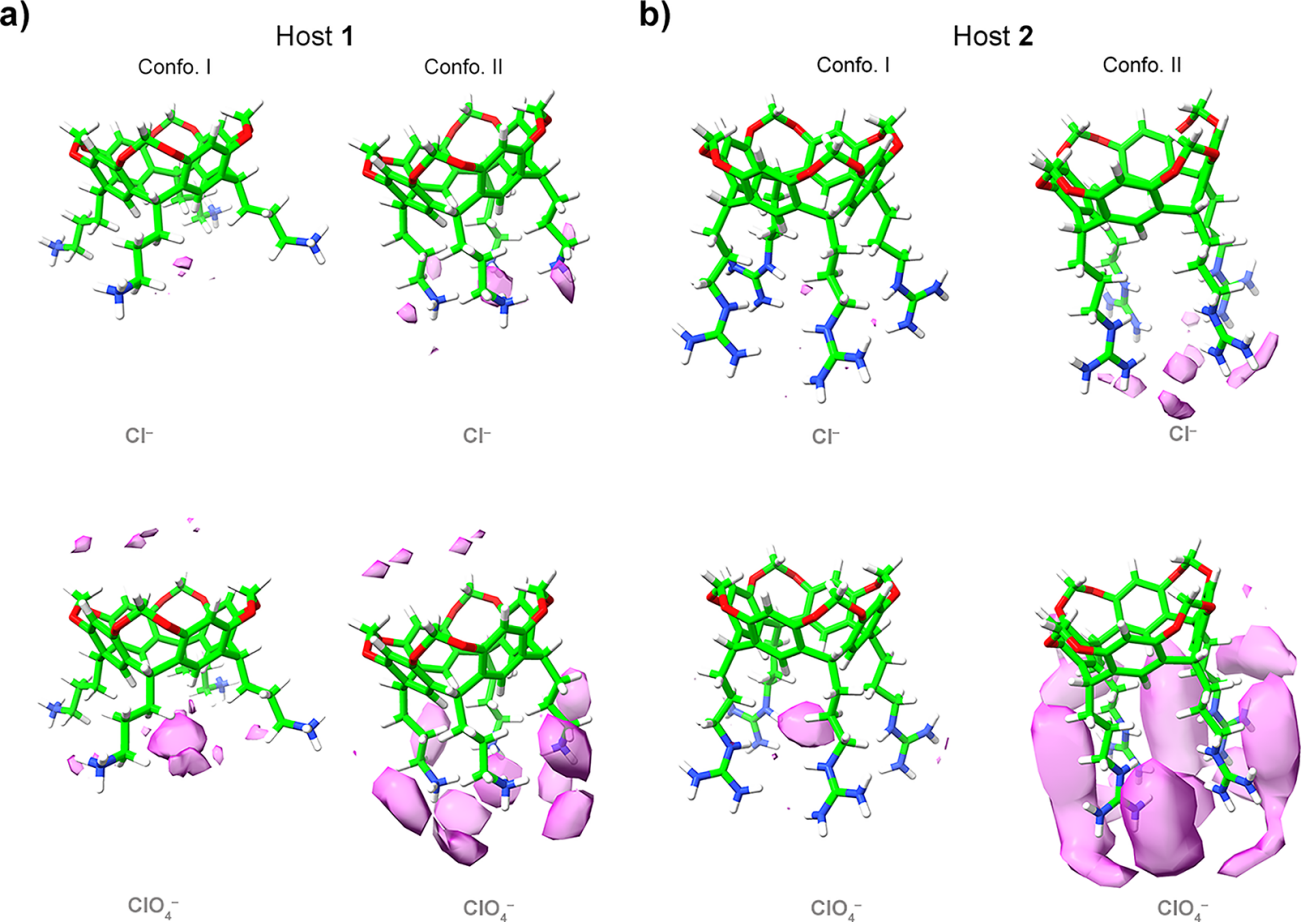
Probability maps of Cl^–^ (*upper row*) and ClO_4_^–^ (*lower row*) derived from MD simulations showing anion associating with (a) host **1** and (b) host **2**. Each host is shown in two conformations (Confo. I and II). The pink “anion clouds” represent probability thresholds of 50× the ion density determined in the bulk. Images were generated using ChimeraX.

**Figure 9:**
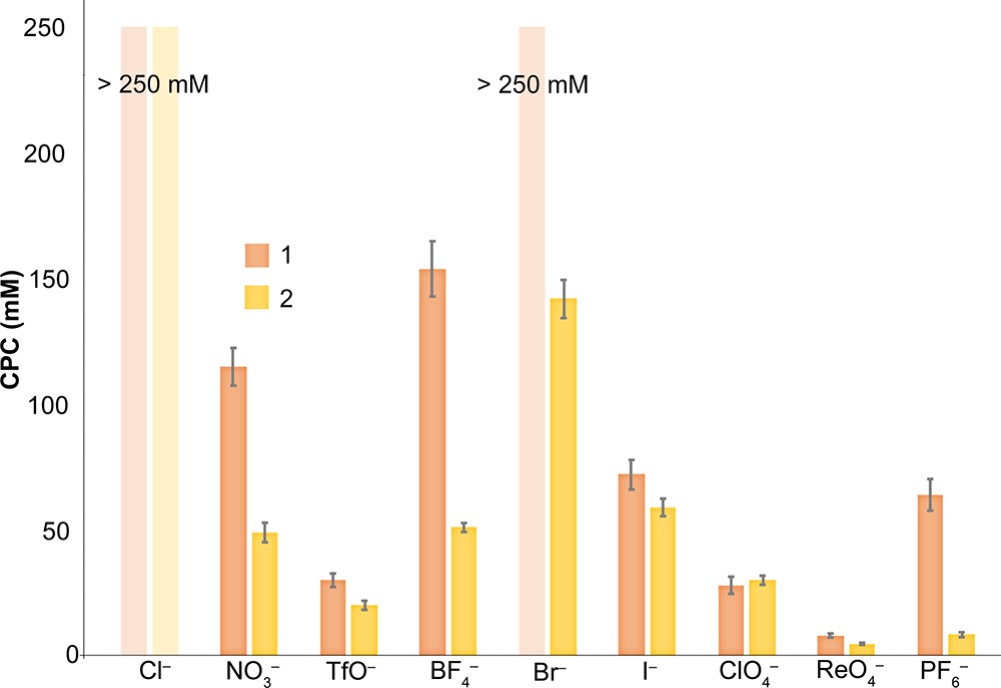
Critical precipitation concentration (CPC) values of anions for hosts **1** and **2** (2 mM) were derived from UV–vis turbidity experiments. The CPC data for Cl^–^ and both hosts, and Br^–^ and host **1**, were in excess of 250 mM. All solutions were 10 mM phosphate buffer in 18.2 MΩ cm^–1^ H_2_O, at pH 3.0 ± 0.1. The error bars are those arising from the triplication of the data.

Figure 3 depicts the layered organization in the obtained structures. Thus, two planes of cavitands were layered pendent-group-to-pendent groups, separated by a layer rich in (dis)ordered anions and waters. The distribution of anions between the pendent group layers and the water/anion-rich layer varied from structure to structure. However, it was always noted that at least two anions were located in a pendent group layer, with one of these in the core binding site between the four pendent groups of individual cavitands (for clarity, only this guest is shown in Figure 3). Our discussion here focuses on the binding motif in and around this core site (Figures 4–9 were generated using ChimeraX^90^). Full details of the solved structures are provided in the Supporting Information (Section 4E).

### Chloride Complex of Host **1**

In the crystal structure of the chloride complex (see Supporting Information Figures S90 and S93), there is a central Cl^–^ guest lying within the binding pocket defined by the four pendent groups. The Cl^–^ guest is slightly off-center, forming close contacts to one of the H_j_ protons, as well as one H_l_ and H_n_ atom on each of the two pendent groups adjacent to said H_j_ atom (see Figure 1 for labeling). Specifically, the Cl^–^ guest is off-center by ∼0.5 Å and results in four Cl^–^···H_j_ distances of 3.12, 3.52, 3.66, and 4.12 Å [average (av) = 3.60]. The closest contacts of the guest to the host are, however, the inward-pointing H_l_ atom of the two pendent groups adjacent to the close contact H_j_ atom (2.73 and 2.93 Å), as well as the inward-pointing H_n_ atoms on the same two pendent groups (2.74 and 3.05 Å). The conformations of the other two pendent groups are such that the aforementioned contacts between the guest and the H_l_, H_m_, or H_n_ atoms are longer (Cl^–^···H_l_ = 3.48 and 4.05 Å, Cl^–^···H_n_ = 3.29 and 6.50 Å).

The adoption of conformations that allow H_l_ and H_n_ atoms to point inward to contact the guest results in the corresponding terminal ammonium groups to be relatively remote from the guest. One pair of ammoniums is more distal (Cl^–^···N distances of 5.07 and 5.10 Å) than the other pair (Cl^–^···N distances of 4.28 and 4.82 Å). For the former pair, there are no direct or indirect interactions with the guest (within the same unit cell). Rather, this most distal pair of ammoniums is bridged by a second Cl^–^ atom (Figure 4). In contrast, the pair of proximal ammoniums stabilizes the bound guest indirectly via an HB network also involving three local waters. Here, the proximal ammonium pair forms a HBing bridge with a water that is itself HBed to the guest and a second water molecule (*upper-most* water in Figure 4). Additionally, one of these proximal ammoniums is HBed to water that forms a second HB to the Cl^–^ guest. Thus, in the unit cell, the guest can be viewed as a dihydrate: Cl^–^(H_2_O)_2_ involving short water-halide contacts (2.23 and 2.30 Å).

### Bromide Complex of Host **1**

Barring some disorder, the complex with Br^–^ is qualitatively identical to that of Cl^–^. Thus, in the crystal structure (Figure 5; see also Supporting Information Figures S94–S97), there is a central Br^–^ guest lying within the pocket defined by four pendent groups. All of the Br^–^ ions, including this guest, are disordered, and disorder is also noted for one of the pendent groups of **1**. As with the Cl^–^ complex, the halide is off-center as judged by the distances to the four H_j_ atoms. More importantly, using the closer of the two anion positions, the average H_j_···Br^–^ distance is shorter than the average H_j_···Cl^–^ distance in the corresponding complex (3.47 versus 3.60 Å). If the more remote position of the guest anion is selected, this average distance is only slightly longer than that of the chloride (average H_j_···Br^–^ = 3.79 Å). Thus, despite the greater contact radii of Br^–^ (1.85 versus 1.75 Å for Cl^–^), by either measure the guest is able to bind slightly more deeply into the pocket, and moreover, because of its greater size, Br^–^ is able to interact with more H_j_ atoms.

As with the Cl^–^ complex, there are also close contacts between the guest and the H_l_ and H_n_ atoms on the pendent groups into which it leans into. The shortest contact for H_l_ was observed to be 2.78 Å, while the shortest contact with H_n_ was 3.06 Å. Thus, given the greater contact radii of Br^–^, the guest is able to form shorter interactions with the pendent groups.

Figure 5 shows the central noncovalent interactions (NCIs) around the bound Br^–^ atom. The central Br^–^ guest is HBed to two water molecules. Selecting the extremes of the halide positioning, the interatomic distances between each water hydrogen and the halide range from 2.45–2.63 and 1.83–2.53 Å. The average of these is shown in Figure 5. Similarly, the disordered bromide ion forming a HBing (ion) bridge between the pairs of ammoniums gives interatomic distances (N–H···Br^–^) from 1.91–2.45 and 2.37–3.06 Å. Again, the averages are given in Figure 5.

### Perchlorate Complex of Host **1**

Host **1** is fully ordered in the obtained structure of the ClO_4_^–^ complex (Figure 6 and Supporting Information Figures S98–S101), but the central bound ClO_4_^–^ guest is disordered, as are two of the other three anions. In the case of the bound guest, its tetrahedral structure is oriented so that a Cl–O bond is approximately colinear with the C_4_ axis of the host, with the oxygen pointing into the pocket and toward the bowl of the cavitand. The disorder of the guest appears to arise primarily from the incongruity of its 3-fold symmetry and the 4-fold symmetry of the host, i.e., there is less disorder in the Cl–O bond colinear with the C_4_ axis of the host.

The general binding mode of the ClO_4_^–^ complex is similar to that of the Cl^–^ or the Br^–^ complexes. Thus, the central ClO_4_^–^ guest binds within the pendent groups of **1**, with one oxygen atom pointing toward, and approximately equidistant to, the H_j_ protons of the host. By the metric of the position of this oxygen atom relative to the H_j_ protons, this guest is deeply bound, forming four short contacts: H_j_···O = 2.68, 2.74, 2.74, and 2.78 Å. Thus, this oxygen atom is deeply located in the pocket, approximately 0.8–0.9 Å more deeply than Cl^–^ of the chloride complex. There are similar short contacts between the closest respective oxygen atom and the H_l_ and H_n_ atoms of the pendent chains. In the case of the former, the shortest distance is 2.69 Å and the average is 2.76 Å, while in the case of H_n,_ these respective values are 2.85 and 3.20 Å. Thus, of the three guests investigated in the solid state, ClO_4_^–^ forms the most and shortest C–H···O–Cl(O_3_)^−^ HBs with host **1**.

Although the general guest binding mode of the ClO_4_^–^ host–guest complex is similar to that so far discussed, the detailed packing motif and supramolecular interactions in the host–guest core are different (Figure 6). Most apparent, one of the two waters that HBs to the guest in the Cl^–^ and Br^–^ complexes is replaced with another ClO_4_^–^. Moreover, the one remaining water that does HB to the ClO_4_^–^ guest does so only weakly (HOH···O–Cl(O_3_)^−^ = 2.45 Å). Instead, the shortest HB to the ClO_4_^–^ guest is from one of the host ammonium groups (2.04 Å). In the solid state, the other ammoniums are more remote from the guest, but this short contact does demonstrate the potentiality of more-weakly solvated ClO_4_^–^ forming multiple RNH_3_^+^···O–Cl(O_3_)^−^ interactions in the solution phase. This type of host–guest interaction was not observed in either the Cl^–^ or the Br^–^ complexes and may reflect the point that charge-diffuse anions can more easily rearrange their solvation shell to form direct host–guest interactions and/or that ClO_4_^–^ prefers softer HB donors such as RNH_3_^+^.^18^

### Perchlorate Complex of Host **2**

Complementing the ClO_4_^–^ complex of host **1**, we obtained the ClO_4_^–^ complex of host **2** (see Supporting Information Figures S102–S105). In this structure, there was little disorder in the core host–guest complex. However, disorder was noted in the other two ClO_4_^–^ anions in the unit cell (Figure 7a, *insert*). The complexity of the guanidinium supramolecular repertoire is evident in the crystal structure. The two well-ordered perchlorates in the cavitand layer interact with the host in different ways, with one forming a face-to-anion interaction and the second an excellent edge-to-anion interaction (Figure 7). As noted, titration fitting (see above) only indicated 1:1 binding in solution.^91^

In the central binding site, close contacts were again observed between the host atoms H_j_, H_l_, and H_n_ and the bound guest. The ClO_4_^–^ guest is seen to bind as deeply to host **2** as it does in host **1** (H_j_···O = 2.69–2.83 Å, versus 2.68–2.78 Å in **1**). Moreover, by measurement of the H_l_ and H_n_ distances to the nearest oxygen atom of the central ClO_4_^–^, host **2** forms a slightly tighter complex. Thus, in the complex with **2**, the shortest distance involving a H_l_ (H_n_) proton is 2.53 Å (2.49 Å), and the average is 2.77 Å (2.94 Å). These are shorter than those in the complex with **1**, where the corresponding shortest and average distances between H_l_ (H_n_) and the guest are 2.69 (2.85) and 2.76 Å (3.20 Å), respectively.

In addition to these short contacts, the centrally located ClO_4_^–^ guest is only directly interacting with the host through one guanidinium (Figure 7a). This interaction is an off-center face-to-anion interaction [centroid to centroid distance = 3.95 Å, c.f. 3.67 Å in guanidinium perchlorate (space group *R*3*m*)^92^]. The central guest also interacts with the host indirectly. Thus, the guest is HBed to two water molecules (Figure 7a), one of which forms a strong HB with one of the guanidiniums (1.90 Å), and the other of which forms a much weaker interaction (2.73 Å).

The second “binding site” for ClO_4_^–^ involves the second pair of guanidiniums. By turning one guanidinium away from and perpendicular to the C_4_ axis, the host is able to form two pairs of HBs to the ClO_4_^–^ (Figure 7b). This is a common supramolecular motif in synthetic guanidium receptors,^1,93,94^ and in the case in hand, three of the four HBs are relatively strong. Thus, for comparison, the N–H···O–Cl(O_3_)^−^ distances in this site are 2.07, 2.12, 2.15, and 2.45 Å (θ between 145 and 167°), whereas in the guanidinium perchlorate (space group *R*3*m*),^92^ all HBs are 2.20 Å (θ = 174).

### MD Simulations

To further assess anion binding to tetra-ammonium **1** and tetra-guanidinium **2**, we carried out MD simulations of two (fixed) conformations of each host in the presence of excess NaCl or NaClO_4_ and analyzed the localization of each anion using TRAVIS (Trajectory Analyzer and Visualizer^95^). The two conformations differed in the pendent groups of the hosts, with either the H_l_ or H_n_ methylenes turned into the pocket (Confo. I) or the H_m_ and NH(R) termini turned into the pocket (Confo. II; Figure 8). Each MD simulation [GROMACS 2016.3, Generalized Amber Force Field (GAFF),^96^ AM1-BCC] utilized 5000 explicit water molecules (TIP 4p-Ew^97^), 26 Na^+^ ions, and 30 Cl^–^/ClO_4_^–^ ions and was performed for 500 ns over 2 fs steps (Supporting Information, Section 5E). The shown “anion clouds” (Figure 8) reveal ion density probability thresholds of 50× the ion density in the bulk. These simulations reveal how weakly solvated ClO_4_^–^ associates relatively strongly with both hosts.

In Confo. I, there is little evidence of Cl^–^ binding to **1**, but more charge-diffuse ClO_4_^–^ associates with the core binding site between the pendent groups. Interestingly, the simulations also suggest some association of ClO_4_^–^ with the rim of the cavitand bowl, primarily with the acidic methylene acetal bridges. Confo. II possesses a narrower pocket but a more intense electrostatic potential field (EPF) induced by the proximal charge groups. In host **1**, this leads to the association of Cl^–^ in the central binding pocket. However, Confo. II is evidently slightly too tight for ClO_4_^–^, and as a result, anion concentration is “pushed” to the outer surfaces of the four pendent groups. It is a similar situation with host **2**. The data with ClO_4_^–^ are particularly illustrative. With a more open pocket (Confo. I), anion binding is focused purely on the inner pocket, but there is insufficient space for the anion to bind to the core pocket in Confo. II and so anion accumulation on the outer surfaces (sides and base) of the pendent group array is extensive.

### CPCs

The reverse (or inverse) Hofmeister effect, whereby charge-diffuse anions induce the aggregation and precipitation of proteins at pH values below their isoelectric point (pI),^74−83^ is in large part controlled by charge neutralization. To investigate the reverse Hofmeister effect in hosts **1** and **2**, we determined the CPCs of select anions to probe how the nature of the anions affected the host solubility. Thus, we obtained CPC values, defined here as the lowest concentration required to produce detectable precipitate (UV–vis) within 15 min. The obtained data are presented in Figure 9. Experimental details are provided in the Supporting Information (Section 4D).

As anticipated, the most charge-dense anions had difficulty salting out either host. Thus, Cl^–^ did not induce the precipitation of either host up to 250 mM salt. This was also the case for Br^–^ and host **1**. Bromide was, however, capable of inducing the precipitation of host **2** at a relatively high concentration of 142 mM. This greater sensitivity of **2** to the presence of excess anion was reproduced in all other anions investigated with the exception of ClO_4_^–^, which precipitated either host with equal ease at 28–30 mM. For **1**, the order of precipitation strength observed was (lowest to highest): BF_4_^–^, NO_3_^–^, I^–^, PF_6_^–^, TfO^–^, ClO_4_^–^, and ReO_4_^–^. Indicative of the anion-specific interactions with each host, a different order was obtained for host **2**: Br^–^, I^–^, BF_4_^–^, NO_3_^–^, ClO_4_^–^, TfO^–^, PF_6_^–^, and ReO_4_^–^. Differing anion-specific interactions were also evident in the remarkable difference in the ability of PF_6_^–^ to induce the precipitation of **1** and **2** (64 and 8.3 mM, respectively) and to a lesser degree the precipitation power of BF_4_^–^ (154 and 51 mM for **1** and **2**, respectively).

## Discussion

We used a range of techniques to probe binding of the anion to cavitands **1** and **2** and the consequences of this association. The aim of this work is to build a better understanding of the aqueous supramolecular interactions between ammoniums/guanidiniums and anions, and hence highlight some of the important NCIs responsible for the reverse Hofmeister effect.

The anions ranged from relatively charge-dense Cl^–^ to charge-diffuse PF_6_^–^. In other words, the focus was on anions that have relatively weak solvation shells that can more readily form direct NCIs with other solutes. It is via such NCIs that anions can induce either the salting-in Hofmeister effect (when the solute is neutral or negatively charged) or the reverse Hofmeister effect (when the solute is positively charged).^71,73,98−103^ All studies were carried out under conditions that ensured constant and maximal protonation (+3 or +4).

### Anions Nestle into Nonpolar Pockets

**Figure 10:**
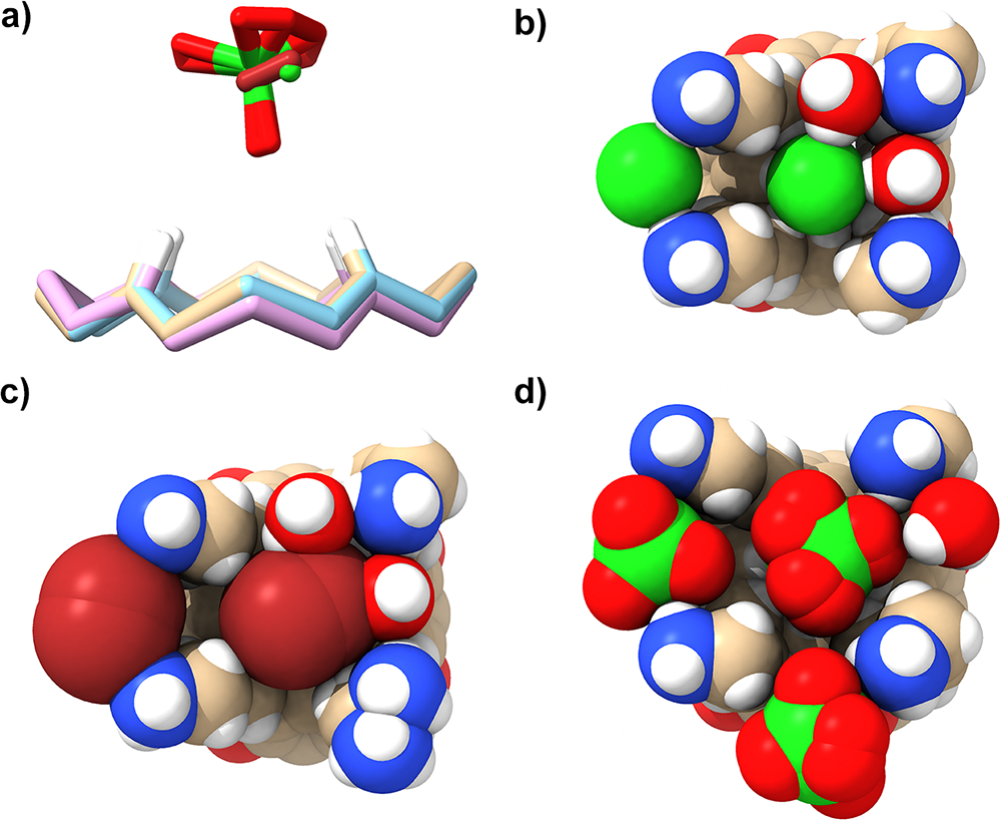
Comparison of the host–guest complexes between **1** and Cl^–^, Br^–^, and ClO_4_^–^. (a) Superimposed partial structures of the complexes showing the depth of guest binding. Here, the lower “macrocycles” (pink, blue, and tan) each represent the ring of carbon atoms constituting the base of the cavitand bowl, and the four indicated H atoms (*white*) are the H_j_ protons. The *tan*, *blue*, and *pink* rings correspond to the atoms in each of the Cl^–^, Br^–^, and ClO_4_^–^ complexes, respectively. Shown above the “macrocycles” are the superimposed guest anions for complexes: Cl^–^ (*green sphe*re, *off-center right*), Br^–^ (*brown rod*), and ClO_4_^–^ showing their relative positions in the binding pocket. (b–d) Space-filling models of the binding sites of the Cl^–^, Br^–^, and ClO_4_^–^ complexes, looking into the open end of each pocket (with the cavitand bowls mostly hidden at the rear). Each representation shows the key host–guest interactions from each unit cell, the centrally bound anion guest, select proximal waters, and other proximal anions. In (c), the disordered Br^–^ anions and one disordered ammonium group are shown. In (d), the disorder in two of the ClO_4_^–^ anions is also shown.

Cavitands **1** and **2** are composed of rigid bowls that act as scaffolds for four pendent groups terminated by either ammoniums or guanidiniums. In combination, the results demonstrate here that the principal anion-binding site in the two hosts is around the C_4_ axis between their pendent groups. ^1^H NMR Δδ_max_ values at infinite salt concentrations provide a “low-resolution” picture of anion binding. A survey of the data from different anions reveals that the signals from protons H_j_, H_l,_ and H_n_ (Figure 1) undergo the largest shifts, whereas the signal shifts of the H_m_ protons are negligible. The obtained X-ray data suggest why this is so; in the different complexes, the pendent groups adopt conformations that generally turn the H_l_ and H_n_ protons inward to form the inner walls of the pocket, while the H_m_ protons point outward (Figure 10, generated using ChimeraX^90^). Focusing on these Δδ_max_ values, it is evident that the monatomic halides induce the largest shifts and that these are particularly large for H_j_ and H_l_. These shifts are downfield, approaching 0.2 ppm.^104^ There are two possible causes for such large shifts in the presence of halides: either they indicate halide binding that is deeper into the crown of pendent groups than is the case with the polyatomic anions or that it is a reflection of their monatomic nature and relatively high electron density. The obtained X-ray results suggest an answer (Figure 10a). Thus, the tetrahedrality of ClO_4_^–^ means that it can bind more deeply into the pocket than the halides, but only in the sense that one Cl–O bond inserts deep into the pocket to allow the oxygen atom to form four short contacts with the C–H_j_ groups of host **1**. As gauged by the centroids of each anion, the anions all bind to roughly the same depth. Thus, the observed large ^1^H NMR shifts induced by the halide complexation are not a reflection of their depth of binding but of their charge density.

Figure 10b–d shows the key host–guest NCIs in the complexes of **1** and Cl^–^, Br^–^, and ClO_4_^–^ from the perspective of the four ammonium groups being closest to the viewer. The “gaps” in the walls of each pocket are bridged with the respective anions from the adjacent unit cell. As defined by the H_j_···X^–^ distances, the central Cl^–^ guest is off-center, displaced to the *left* in Figure 10b, to interact with the two pendent groups. Thus, Cl^–^ is evidently a bit small for the pocket of **1**, and despite some contraction of the pocket (compare the size of the parallelograms defined by the ammonium groups in Figure 10b–d), it can only form limited interactions with less than half of the H_j_, H_l_, and H_n_ atoms. In the unit cell, the only other interactions of the guest are HBs to two waters (at 12 and 3 o’clock) that also bridge pairs of adjacent ammoniums. Thus, in the solid state, the guest is a diaquo species.

It is a similar situation with the Br^–^ guest. In Figure 10c, the guest is also displaced to the *left*. The larger Br^–^ guest is disordered over two locations, suggesting that although bigger and capable of forming more direct short contact C–H···X^–^ interactions with the H_j_, H_l_, and H_n_ atoms, it is smaller than ideal. Other than this disorder, the Br^–^ complex is essentially identical to the Cl^–^ complex.

The size and tetrahedrality of ClO_4_^–^ allow it to form direct contacts with the four H_j_ protons, multiple H_l_ and H_n_ protons, and one ammonium termini of host **1** (*top right* in Figure 10d). This last close contact is shorter than the weak HB to the ClO_4_^–^ from adjacent water (3 o’clock) in the unit cell. We link this apparent “disinterest” in direct HBing to water at least in part to the lower free energy of hydration of perchlorate; the anion is more charge-diffuse and—unlike Cl^–^ and Br^–^—prefers to HB directly with the more charge-diffuse and less electronegative N–H group rather than HB to a water molecule.

**Figure 11:**
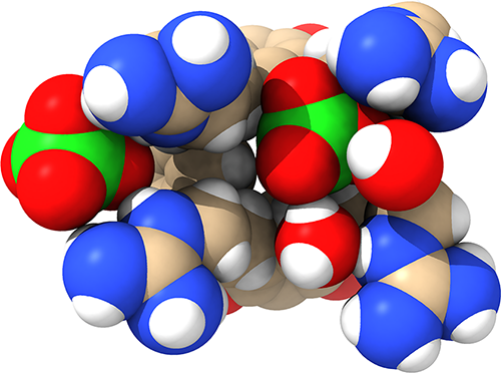
Comparison of the binding sites in the solid-state structure of the complex between **2** and ClO_4_^–^. This space-filling model of the binding sites looks down into the central pocket such that the cavitand bowl is mostly hidden at rear. The two HBed waters to the centrally bound perchlorate (right) are shown. Other more peripheral waters, as well as the two disordered perchlorates, have been omitted for clarity.

The case of ClO_4_^–^ binding to host **2** is more complex, showing two well-defined binding sites despite the solution data showing a well-defined 1:1 complex formation. The spatial relationship of these two sites is shown in Figure 11 (generated using ChimeraX^90^). At the core site, the structure shows the ClO_4_^–^ leaning into the pendent group that also forms direct interactions with its terminal guanidinium group (*top right*). The second guanidinium at this site (*lower right*) has turned “open” to expose the guest to the water- and ion-rich layer (Figure 3), allowing a strong HB water bridge between one N–H of the guanidinium edge and the bound ClO_4_^–^. This open conformation allows a second water (3 o’clock) to HB to the centrally bound guest. As Figure 11 shows, with the remaining two guanidiniums turned into closed (*top left*) and open positions (*bottom left*), host **2** has *pseudo-*2-fold symmetry. However, presumably because of space restrictions and ion–ion repulsion, the second ClO_4_^–^ (*left*) binds outside the pocket, forming two pairs of HBs to the host. These are close to ideal, differing little in geometry from that seen in the crystal structure of guanidinium perchlorate.^92^

In each of these X-ray structures, it is evident that the primary guest is nestled down into the pocket, somewhat below the plane defined by the four charge groups. This is true even for relatively strongly solvated Cl^–^, which like all examples here, is hydrated when bound only on one of its hemispheres. Thus, the anions do not bind by minimizing their separation to the four cationic charges while maintaining a full solvation shell (by binding either in the plane of the ammoniums or just outside the pocket at the tips of the pendent groups). Rather, in the solid state, it is energetically preferable for an anion to partially desolvate and nestle into the pocket in a manner reminiscent of sodium halide binding into membranes of zwitterionic phosphatidylcholines.^105^

To further examine anion binding, we carried out explicit water model MD simulations and anion trajectory analysis.^95^ These probability calculations have two limitations. First, the only NCIs accounted for are Coulombic (ion–ion), ion–dipole, and VdW interactions (as a Lennard-Jones potential; dispersion effects were not accounted for). Second, the conformation of the host must be fixed after the initial optimization. As a work-around for this latter problem, two conformations were selected (Figure 8). In Confo. I, the H_l_ and H_n_ methylenes are turned inward to create a larger pocket and more distal ammoniums. This last point presumably leads to a relatively more diffuse EPF. In contrast, in Confo. II, the H_m_ methylenes are turned inward, the central binding pocket smaller/narrower, and the ammonium groups closer together.

These MD simulations reveal the greater probability of ClO_4_^–^ accumulation on the host surface. Thus, despite forming relatively weak Coulombic and ion-dipole interactions, the greater VdW interactions that ClO_4_^–^ can form with the host are key. In other words, water is a poor competitor for both the host and the guest and therefore not a good interferant in direct host–guest contacts.

The MD simulations also suggest a role for the pendent group conformation. In the more open Confo. I, ion-accumulation is very “focused” (small probability threshold volume) on the pocket. This is especially so in the case of Cl^–^ binding, indicating that the observed binding site in the solid state is reproduced in the solution phase despite the approximations in the simulations. On the other hand, the probability of ClO_4_^–^ binding is not so focused but still very much centered on the core site. This reduced focus is presumably a result of the weakness or greater plasticity/adaptability of the ClO_4_^–^ solvation shell and the greater number of NCIs between anion and host.

**Figure 12:**
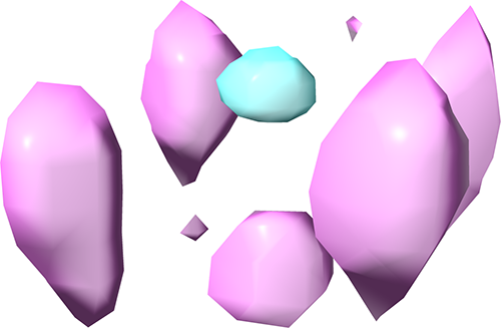
MD simulation data showing the probability distribution of ClO_4_^–^ (representing thresholds of 200× the ion density determined in the bulk) around the pendent groups of Confo. I (*cyan, core binding*) and Confo. II (*pink, exterior binding*) of host **2**. For clarity, the host atoms are hidden, but as shown, the cavitand bowl lies at the top of the image, and the four pendents run vertically down between the inner (*cyan*) and outer (*pink*) probability zones. For orientation, the central pink zone at the base of the image corresponds to anion binding via edge-to-anion HBing, outside of the pocket and at the tips of the guanidiniums.

In Confo. II, the combination of a smaller pocket and presumptive stronger EPF alters Cl^–^ binding. For example, in **1**, the pocket is slightly too small for Cl^–^. As a result, the probability of guest binding is moved to the exterior of the host. This effect is exaggerated with larger ClO_4_^–^, where anion accumulation is on the outside of the host. Consider also the case of the binding of ClO_4_^–^ to Confo. I and II of **2**, where the larger size of the guanidiniums exacerbates this redistribution of the anion. Figure 12 (generated using ChimeraX^90^) merges the data for both conformations of this complex (cf. Figure 8), with the threshold increased to 200× bulk anion density and the host atoms hidden to clarify the differences. As can be seen, in open Confo. I, the highest probability of finding ClO_4_^–^ is deep in the pocket (*cyan*). In contrast, in Confo. II, there is no pocket binding; because of the size of the guest and the guanidiniums, the probability of finding the anion is at the host outer surface. This includes a small zone (Figure 12, *lower center*) on the C_4_ axis of the host corresponding to (outer) edge-to-anion HBing to the guanidinium groups as well as the four grooves running between the alkyl chains of the pendent group cluster.

While the energetic costs of completely desolvating an anion are large, the costs of rearranging a solvation shell or partial desolvation are not well understood.^106^ What compensates for this energetic costs of removing one “hemisphere” of the solvation shell of a bound anion, as is the case here? The X-ray data and MD simulations highlight that C–H···anion VdW interactions are key, but presumably another contributor is the relatively low permittivity of the pocket that allows stronger Coulombic interactions between host and guest.

Unfortunately, the complexity of the thermodynamics of the rearrangement of the solvation shell of ions and/or their partial desolvation, as well as the conformational flexibility of the pendent groups, makes detailed interpretation of the ITC data difficult. Free energies of ion hydration (Δ*G*_hyd_) are known,^107^ but these values are of limited utility where complexation involves only partial desolvation. Regarding this, it has been established that for anions in the gas phase, the majority of the Δ*G*_hyd_ is gained with the coordination of the first few waters,^106^ i.e., in the clustering reactions (*n* – 1, *n*): X^–^(H_2_O)_*n*−1_ + H_2_O ⇌ X^–^(H_2_O)_*n*_, the stepwise equilibrium constants are highest at low *n* values. It is also understood that the stepwise equilibrium constants are generally higher for charge-dense anions, i.e., ion-specific.^106^ On the other hand, simulations have established many salient points regarding the solvation of hard spheres. Thus, it has been established that continuum (implicit) models of water cannot reproduce multiple facets of ion hydration,^108,109^ and that explicit water models, although much better, yield Δ*G*_hyd_ values that are model dependent.^109^ In terms of the thermodynamics of ion complexation in water, to the best of our knowledge, information from simulation is limited. It has been established that the hydration of cations and anions is asymmetric—for the same ion size, the free energy of hydration of anions is more favorable than for cations—and that this is rooted in the asymmetry of charge distribution in the water.^109^ It has also been established that solvation differences are largest for small ions and diminish with increasing ion size, converging to a hydrophobic-like hydration structure for the largest, most charge-diffuse ions.^109,110^ This is presumably linked to the observation that large anions such as dodecaborates bind strongly to cyclodextrins.^111^ To our knowledge, though, our understanding of the structural and thermodynamical details of anion hydration in the context of forming NCIs with a host is limited.

### Contrasting Supramolecular Repertoires of Ammoniums and Guanidiniums

In general, polyatomic anions bound more strongly than monatomics. This can be attributed to the weaker solvation of charge-diffuse anions,^107^ and hence more C–H···anion^–^ (VdWs) interactions and stronger Coulombic interactions in the low dielectric pocket. These presumably contribute to the observed reversal of halide affinity (I^–^ > Br^–^ > Cl^–^) to that observed by guanidiniums in free solution.^112^

However, the structural details of anion binding in the solution phase are unresolved. Why does host **2** display a stronger affinity for ReO_4_^–^ relative to ClO_4_^–^? These anions are increasingly being investigated in affinity studies, and work has revealed host-specific selectivities. For example, ReO_4_^–^ has a higher affinity for cavitands^84,113,114^ and for foldamers that form halogen/chalcogen interactions with the anion.^115^ In contrast, ClO_4_^–^ binds more strongly than ReO_4_^–^ to bambusurils.^116^ In the solid-state structures of guanidinium perchlorate^92^ and perrhenate,^117^ the two N–H···O–XO_3_^–^ distances in the guanidinium edge-to-anion interaction are much shorter and symmetrical in the case of ClO_4_^–^, suggesting ClO_4_^–^ forms a stronger pair of HBs. Conceivably then, the stronger affinity for ReO_4_^–^ versus ClO_4_^–^ for **2** may suggest a face-to-anion interaction motif. This idea coincides with steric restrictions for edge-to-anion interactions in **2** (MD simulations) and the X-ray structure of the complex between **2** and ClO_4_^–^.

Despite the fact that ReO_4_^–^ binding could not be studied by ITC because of precipitation issues, calorimetry provided further information about guest binding. Thus, within the group of guests that proved to be amenable, there are some interesting selectivities. For example, the least exothermic binding observed was for TfO^–^ to host **1** (Δ*H* = −4.4 kJ mol^–1^). This weak exothermicity and the inability to obtain data for TfO^–^ binding to host **2** suggest that the CF_3_SO_2_^–^ group appended to the third, charged oxygen is simply too long and/or bulbus to bind between the four pendent groups of **1** or **2**. As a result, the anion can only associate by “head-first” partial insertion of its sulfonate group into the interpendent group space. The low exothermicity of TfO^–^ binding may also suggest that the observed weak binding is a manifestation of the fluorophobic effect, but we find this suggestion problematic considering the high affinity of TfO^–^ for the nonpolar pocket of deep-cavity cavitands.^84^

Another point of interest from the ITC data is that the observed preference for either host to bind larger halides is an enthalpic phenomenon; there is a substantial 10.1 kJ mol^–1^ difference between the Cl^–^ and I^–^ binding enthalpies for host **1**. More generally, anion binding to **1** and **2** is always exothermic, and the binding of polyatomic anions evolves less heat than monoatomics. Correspondingly, there is a substantially favorable entropy change for the binding of polyatomic guests, whereas halide binding involves generally smaller favorable changes in entropy and sometimes entropy changes that counter complexation. In contrast, for 1:1 host–guest complex formation involving charge-diffuse anions binding to the nonpolar pockets of cavitands,^113^ bambusurils,^116^ cyclodextrins,^111,118,119^ and foldamers,^115^ complexation is driven by enthalpy and entropically penalized. Presumably, the proximal charge groups of the host are a significant factor behind this different thermodynamic signature, leading to Coulombic interactions and solvation effects that are quite distinct.

To examine the reverse Hofmeister effect, we investigated the precipitation of the hosts in the presence of different salts (Figure 9). Charge-dense Cl^–^ is incapable of inducing the aggregation of either host. In contrast, Br^–^ does not precipitate **1**, but precipitates **2** at 142 mM. Nitrate and BF_4_^–^ are better precipitators, while perrhenate is an extreme precipitator, inducing the precipitation of **1** and **2** at 8.0 and 4.7 mM, respectively. The data in Figure 9 also demonstrate how it is possible to use the reverse Hofmeister effect to separate mixtures. Thus, 150 mM BF_4_^–^ can selectively precipitate **1**, whereas 150 mM Br^–^ can selectively precipitate **2**. In short, Figure 9 acts as a guide for the isolation and manipulation of ammoniums or guanidiniums.

As anticipated, *en masse*, the data show that guanidiniums are more sensitive to the presence of anions than ammoniums. Only ClO_4_^–^ is capable of precipitating the two hosts at approximately the same concentration. Why are guanidiniums so sensitive? These simple models provide some guidance. For example, in the absence of buffer, the final solutions from titrations (∼80% 1:1 complex) were stable for multihour periods. Moreover, assuming that no higher complexes can form, at the identified CPC concentrations between 82 and 99% of the host is in the bound state (in most cases, the CPC values correspond to >95% 1:1 complex). Relatedly, there is no correlation between the NMR-derived 1:1 affinity data and CPC values (see Supporting Information, Section 4D, Figure S86).^120^ These points suggest that it is the higher complexes (1:2 etc.) that result in collisions leading to aggregation with the greater surface area and supramolecular repertoires of guanidiniums enhancing the probability of aggregation.

## Summary and Conclusions

The combination of NMR, ITC, X-ray, and MD simulations presented here emphasizes the multitude of NCIs that guanidiniums can form and how—at least qualitatively—this repertoire far surpasses that of ammoniums. The data also emphasize the ease by which charge-diffuse anions can partially desolvate and form direct noncovalent contacts with hosts. There is much to learn about the energetic costs of anion-water NCIs and how these compare to the energy gains interacting with hosts and how these can assist our understanding of complex proteinaceous systems.

## Data Availability

The data underlying this study are available in the published article and its Supporting Information.

## Supporting Information Available

Details of the synthesis and characterization of hosts **1** and **2**; protocols for NMR and ITC-based affinity determinations; CPC determinations; characterization of the solid-state structures of the presented four complexes; and computational studies MD simulations, trajectory analysis, and dipole moment calculation (PDF)
