# Supplementary material for: Anion Binding to Ammonium and Guanidinium Hosts: Implications for the Reverse Hofmeister Effects Induced by Lysine and Arginine Residues
Source: J Org Chem. 2024 Apr 25;89(10):6877–91. doi: 10.1021/acs.joc.4c00242 (PMC11110012; doi:10.1021/acs.joc.4c00242)
Supplement: Supplementary file 1 — jo4c00242_si_001.pdf [file jo4c00242_si_001.pdf]

SUPPORTING INFORMATION FOR:

Anion binding to ammonium and guanidinium hosts: implications for the reverse Hofmeister effects induced by lysine and arginine residues

Jacobs H. Jordan[1,§], Corinne L.D. Gibb[2,§], Thien Tran[2], Wei Yao[2], Austin Rose[2], Joel T. Mague[2], Michael W. Easson[1] and Bruce C. Gibb[2*]

[1] The Southern Regional Research Center
Agricultural Research Service
US Department of Agriculture
1100 Allen Toussaint Blvd.
New Orleans, LA 70124, USA

[2] Department of Chemistry
Tulane University
New Orleans, LA 70118, USA
*bgibb@tulane.edu

Jacobs H. Jordan orcid.org/0000-0002-0238-3864
Corinne L. D. Gibb orcid.org/0000-0002-2985-6799
Thien Tran orcid.org/0009-0001-9359-5829
Wei Yao orcid.org/0000-0002-4229-0486
Austin Rose orcid.org/0009-0005-9275-0371
Joel T Mague orcid.org/
Michael W. Easson orcid.org/0000-0002-2268-1922
Bruce C. Gibb orcid.org/0000-0002-4478-4084

[§]These authors contributed equally to this work.

Table of Contents

Experimental Section

1. Materials, instrumentation, and sample preparation procedures S4
   A. General methods, materials and instrumentation S4
   B. MALDI-TOF sample procedure S4
   C. ESI MS sample procedure S4
   D. NMR solution sample preparation procedure. S4
   E. Precipitation assay sample preparation procedure S4
   F. ITC sample preparation procedure. S5
   G. Errors S5

2. Experimental procedures for new compounds .... S6 — S8
   A. Synthetic schemes for the synthesis of hosts **1** & **2.** S6
   B. Synthesis of tetrahydroxy cavitand **4** S6
   C. Synthesis of tetrabomide cavitand **5** S6
   D. Synthesis of tetrakis(azido) cavitand **6** S7
   E. Synthesis of tetrakis(ammonium) cavitand **1** S7
   F. Synthesis of tetrakis(guanidinum) chloride cavitand **2** S8

Data & Results

0. Data & **Results**

Spectral Data S9 — S34
   A. Tetrahydroxy cavitand **4** S9
   B. Tetrabomide cavitand **5** S11
   C. Tetra(azido) cavitand **6** S15
   D. Tetrakis(amino) cavitand **1a** S19
   E. Tetrakis(ammonium) chloride cavitand **1** S22
   F. Tetrakis(guanidinium) chloride cavitand **2** S29

4. Analytical Data .... S35— S91
   A. NMR Shift (Δδmax) Summary S35
   B. NMR Data .... S36 — S55
      a. ^{1}H NMR titrations of host **1** with salts .... S38 — S46
      b. ^{1}H NMR titration of host **2** with salts .... S47 — S55
   C. Isothermal Titration Calorimetry Data .... S56 — S68
      a. ITC protocols for titration of host **1** with salts S56
      b. ITC protocols for titration of host **2** with salts S57
      c. ITC Data for titration of host **1** with salts .... S58 — S62
      d. ITC data for titration of host **2** with salts .... S63 — S68
   D Critical precipitation assay data .... S69 — S72
      a. Precipitation Screening S69
      b. Critical Precipitation Concentration (CPC) Determination S69
      c. Relationship between CPC and thermodynamics of binding and ionic radii S70 — S72

E. Crystallographic analysis of complexes **1.Cl⁻**, **1.Br⁻**, **1.ClO4⁻**, and **2.ClO4⁻**. .... S73 — S91
a. General Crystallography Details S73
b. Crystal and refinement data S74
c. Responses to “Alerts” S75
d. Packing Diagrams for Structures **1.Cl⁻**, **1.Br⁻**, **1.ClO4⁻** and **2.ClO4⁻** .... S76 — S91

5. Molecular dynamics simulations and spatial distribution functions (SDFs) .... S92 — S97
A. Host modelling S92
B. Molecular Dynamics Simulation details S96
C. Spatial distribution function S97
D. Dipole Moment Calculation S97
6. References .... S98 — S99

Experimental Section

1. Materials, instrumentation, and sample preparation procedures

General methods, materials and instrumentation

Reagents were purchased from commercial suppliers Sigma-Aldrich Corp. or TCI America and were used without further purification. All sodium salts were of purity ≥ 98 % and were used as received. All solvents were purchased from Fisher Scientific and were used as received. All compounds were prepared under a nitrogen atmosphere and the pressure maintained with a nitrogen balloon. For reactions that required heating, the sample was heated with an oil bath at the given temperature. Resorcinarene **3,** was synthesized by a modification to the procedures recently reported[1-4] and cavitand **4** was synthesized by modifications to a scaled procedure reported previously.[3, 5] All ^{1}H NMR spectra were collected on a Bruker 500 MHz or Varian 400 MHz spectrometer at 25 ˚C, and all ^{13}C NMR spectra were collected on a Bruker 300 MHz (75 MHz ^{13}C) at 25 ˚C. Structural assignments were made with additional information from gCOSY, gHSQC, and gHMBC experiments. All titrations utilized deuterium oxide (Cambridge Isotopes, 99.9%+). Δδ values were referenced to the residual solvent signal (δ = 4.70 ppm). Spectral processing was performed using Mnova software (Mestrelab Research, S.L.) with results fitted using the online software BINDFIT[6] or SOLVER in Excel. MALDI-MS and ESI-MS spectra were collected using a Bruker Autoflex II MALDI-TOF mass spectrometer and a Bruker microTOF mass spectrometer, respectively. Precipitation assays involved monitoring samples at a 500 nm wavelength and a temperature of 23 °C, using an Enspire Multimode plate reader (PerkinElmer, Inc – Waltham, MA) equipped with a Xenon flash lamp, quad monochromator, dual temperature controller, and dispenser. In all cases, Corning™ non-treated clear polystyrene ½-area 96-well micro-plates (Fisher Scientific) were used. Isothermal Titration Calorimetric (ITC) experiments were performed using a VP-ITC MicroCalorimeter from Microcal, USA.

MALDI-TOF sample procedure

All samples were prepared using the dried-droplet method using 2.5 µL per droplet. The matrix utilized was *trans*-2-[3-(4-*tert*-butylphenyl)-2-methyl-2-propenylidene]malononitrile (DCTB, 10 mg·mL^{-1} in THF). The matrix (10 µL) was mixed with 20 µL of compound **5** prepared in a 1:1 solvent blend of $CHCl_3$:CH_3CN + 0.1% formic acid (2 mg·mL^{-1}) for a mix ratio of 2:1 (matrix:analyte). For compound **6**, 20 µL prepared in THF (2 mg·mL^{-1}) was added to 10 µL of matrix and 10 uL of a silver trifluoroacetate solution (1 mg·mL^{-1} in THF) for a mix ratio of 2:1:1 (matrix:analyte:ion).

ESI MS sample procedure

All samples were prepared as 10–100 µM concentration solutions in distilled H_2O (dH_2O). ESI-MS spectra acquisitions were acquired using a Bruker microTOF mass spectrometer in positive mode and averaged from 1.0–10.0 minutes. Ions were continuously generated by infusing the aqueous solution samples into the source with a syringe pump at flow rates of 6 µL/min. The parameters were adjusted and are typically as follows: capillary voltage (–4.1 kV); capillary exit voltage (70 V); skimmer voltage (40 V); drying gas temperature (200 °C). The experiments were carried out with a nebulizer gas pressure of 0.3 Bar and a drying gas flow of 4.0 L/min.

NMR solution sample preparation procedure.

All solutions were prepared in unbuffered D_2O. All titrations of host were carried out with 0.4 mM host solutions prepared from a concentrated stock of ~4 mM; the concentration of the stock solution was determined by titration in triplicate with separate 50 mM sodium ethanesulfonate (SES) solutions, and integration of the methyl peak of ethanesulfonate and the H_m and/or H_l peak of the host (Scheme S1, Section 2.A, below). A concentrated salt solution (10–500 mM) was prepared for use in each titration. The pD of the solutions was uncorrected and was 5.2 ± 0.1 for solutions of host **1** and 5.9 ±.0.1 for solutions of host **2.** The host solutions (0.5 mL) were titrated in an NMR tube with careful addition of small aliquots of the sodium salt of the anion.

Precipitation assay sample preparation procedure

Precipitation assays involved: 1) Initial screening of salts, and; 2) critical precipitation concentration (CPC) determinations for each salt. All solutions of hosts (**1**–**2**) were prepared using a 10 mM phosphate buffer in 18.2 MΩ cm^{-1} H_2O, at pH 3.0 ± 0.1.

For the initial screening, separate stock solutions of salts were prepared as 500 mM solutions and diluted as necessary. Host solutions of **1**, and **2** were prepared as 4.0 mM stock solutions. Precipitation screening assays were conducted at a final host concentration of 2.0 mM. Screening assays were conducted from 250 mM to 0.49 mM salt concentration by successive serial ½ dilutions at twice the final assay concentration (25 µL of stock host solutions were added to 25 µL previously diluted salt solutions).

For the determination of critical precipitation concentration (CPC) values, measurements were performed in at least triplicate from separate stock solutions of hosts (4.0 mM) and salts, typically prepared between 20–160 mM. The pH of the stock solutions was 3.0 ± 0.1 and adjusted as necessary during dilution. For each CPC determination, 25 µL of host (4.0 mM) were added to wells containing 5–20 µL buffer in fixed increments, and 5–20 µL of salts in fixed increments of variable concentration. The concentration of the diluted stock solution of salt was chosen such that the expected CPC value fell within the range of 10–40% of the stock solution. The final volume was 50 µL and the final host concentration was 2.0 mM. Assessments of precipitation were made at t = 15 min to minimize kinetic effects.

ITC sample preparation procedure.

Solutions of host and guest were prepared in 10 mM phosphate buffer (pH = 3.0 ± 0.05) for hosts **1**, and **2** in 18.2 MΩ H_2O. Solutions of host and guest were prepared from a concentrated stock solution to achieve a final host concentration as described in the individual sections, typically between 0.2–1.0 mM host and 5–400 mM salt. If necessary, the pH was adjusted during dilution. Both the host and guest were prepared and adjusted to the same pH value (± 0.05) as the buffer solution. Before each ITC experiment, both host and salt solutions were degassed for 2–5 min to eliminate air bubbles. The enthalpy (ΔH) and free energy (ΔG, calculated from K)) data shown was the average of a minimum of three determinations. The entropy ($-T\Delta S$) was calculated from the subtraction of the mean free energy and mean enthalpy.

Errors

Results are expressed as the average, when possible, with the coefficient of variation (CV) expressed as a percentage of the mean, when applicable, where s is the sample standard deviation and μ is the sample mean:

$$CV\% = \frac{s}{\mu} \times 100 \qquad \text{Eq. (S1)}$$

2. Experimental procedures for new compounds

Synthetic schemes for the synthesis of hosts **1** & **2**.

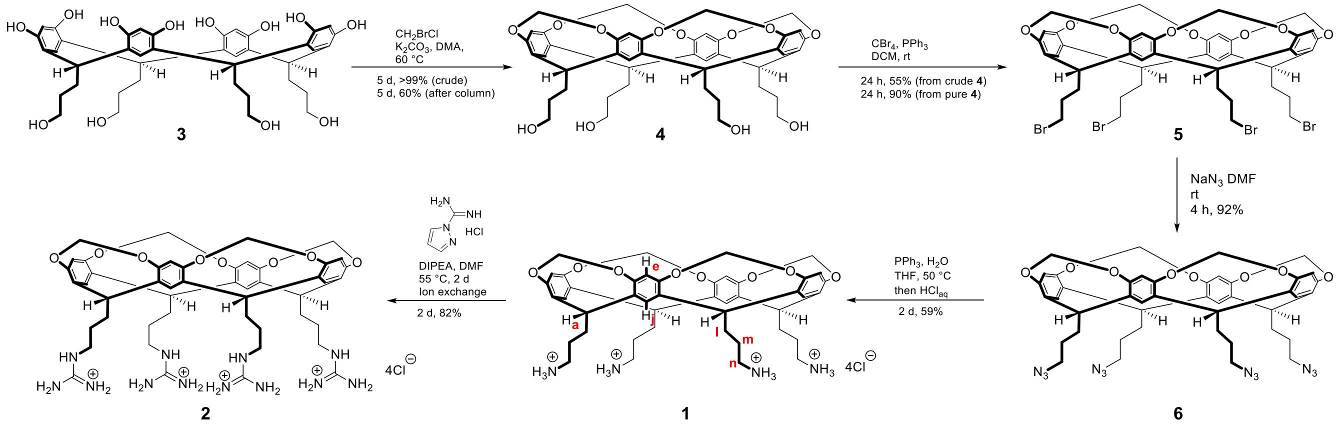

Scheme S1. Syntheses of tetra-ammonium **1** and tetra-guanidinium **2.**

Synthesis of tetrahydroxy cavitand **4**

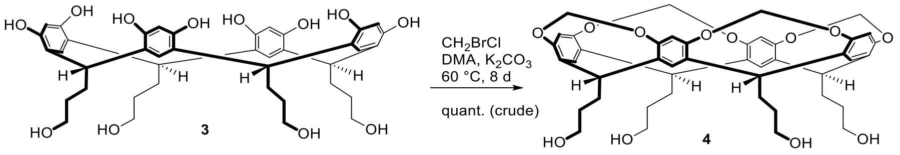

Known compound **3**, (dodecol, 20.4 g, 28 mmol) was added to 500 mL of degassed DMA. The solution was degassed for ~5 min (until all vigorous bubbling has ceased) and 34.4 g (250 mmol, 8.8 equiv.) oven-dried K_2CO_3 was added. The solution was degassed for an additional ~5 min followed by the addition of bromochloromethane (36.0 mL, 554 mmol, 20 equiv.). The reaction was then heated to 60 °C for 5 d (oil bath temperature). After this time, the solvent was removed under reduced pressure and the residue dried overnight at rt. The solids were then suspended in 300 mL of 1 M HCl and sonicated, the fine precipitate collected by filtration, and subsequently washed with water (3 × 300 mL) to give 22.8 g of crude **4** as a tan-colored solid after drying overnight at 110 °C under vacuum (>99%). The crude compound was used without further purification. The compound may be purified by chromatography $CHCl_3$–MeOH (9:1) to give the pure compound (12 g, ~60%), (Figures S1–S2). ^{1}H NMR (400 MHz, DMSO-d_6): δ 7.57 (s, 4H), 6.47 (s, 4H), 5.67 (d, J = 7.7 Hz, 4H), 4.51 (t, J = 8.7 Hz, 4H) 4.45 (t, J = 5.0 Hz, 4H), 4.34 (d, J = 7.6 Hz, 4H), 3.47 (t, J = 6.0 Hz, 8H), 2.37 (d, J = 7.7 Hz, 8H), 1.41 (t, J = 7.2 Hz, 8H).

Synthesis of tetrabomide cavitand **5**

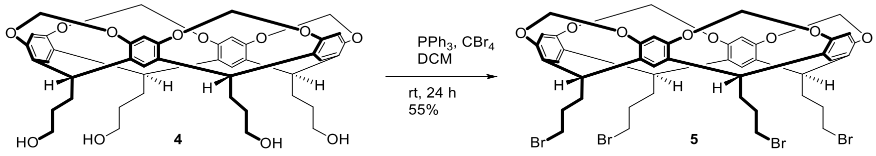

DCM (300 mL) was added to an oven-dried round-bottomed flask containing crude cavitand **4** (3.0 g, 3.9 mmol) and equipped with a magnetic stir bar. While stirred, the solution was sparged with N_2 for 10 minutes and then PPh_3 (6.2 g, 24 mmol, 6.1 equiv.) was added, and the sparging continued. To the stirred solution was added CBr_4 (7.8 g, 24 mmol, 6.0 equiv.) and the flask capped under a nitrogen atmosphere (balloon) and allowed to slowly warm to rt and stirred for 24 h. After, the solution was poured over a chloroform-wet silica plug and flushed with dichloromethane (1.6–2.5 L total, checked by TLC).

The solvent was removed under reduced pressure, the white solids taken up in diethyl ether (150 mL) and sonicated (< 30 s) and collected by filtration and washed with additional diethyl ether (2 × 50 mL). Drying overnight at 110 °C gave the pure compound as a white solid (2.2 g, 55%). For reaction with pure **4** (1.0 g, 1.3 mmol) in 100 mL DCM, the conditions are the same using 2.0 g PPh_3 (7.6 mmol, 5.9 equiv.) and 2.6 g CBr_4 (7.8 mmol, 6.0 equiv.) and work up with ~1.0 L DCM over silica plug to give 916 mg **5** as a white solid (90%), (Figures S3–S7). ^{1}H NMR (400 MHz, $CDCl_3$): δ 7.13 (s, 4H), 6.49 (s, 4H), 5.73 (d, *J* = 7.1 Hz, 4H), 4.75 (t, *J* = 7.9 Hz, 4H), 4.42 (d, *J* = 7.4 Hz, 4H), 3.67 (t, *J* = 6.2 Hz, 8H), 2.42 (m, 8H), 1.94 – 1.73 (m, 8H). ^{13}C{^{1}H} NMR (75 MHz, DMSO): δ 154.7, 138.3, 122.4, 117.3, 99.4, 45.5, 36.2, 31.3, 26.8. *MALDI-TOF MS m/z:* Calcd for $C_{44}H_{44}Br_4O_8H^+$: $[M+H]^+$ 1020.981; Found 1021.028. Anal. Calcd for $C_{44}H_{44}Br_4O_8{\bullet}CH_2Cl_2$: C, 48.90; H, 4.19. Found: C, 48.59; H, 4.10.

Synthesis of tetrakis(azido) cavitand **6**

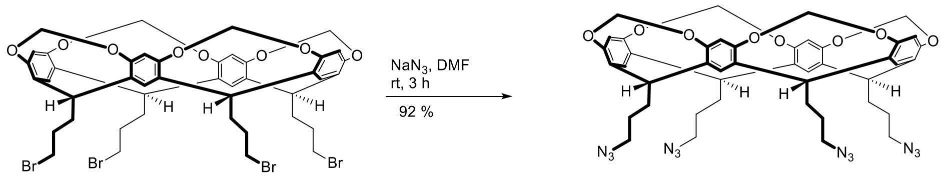

5 **6**

Tetra-bromo cavitand **5** (1.02 g, 1.0 mmol) was added to a dry 50 mL RBF containing 40 mL anhydrous DMF. The flask was warmed to dissolve the solid and then allowed to cool to rt. Sodium azide (390 mg, 6 mmol, 6 equiv.) was added and the clear, yellow solution stirred at rt for 4 h. A white precipitate was observed (NaBr) and the solvent was removed under reduced pressure. The solids were dried for 3 h at rt, sonicated in distilled H_2O, and collected by filtration and washing with water (3 x 30 mL). The precipitate was dried overnight at 110 °C to obtain 797 mg (92%) of the desired product as a white solid (Figures S14–S18). ^{1}H NMR (400 MHz, $CDCl_3$): δ 7.10 (s, 4H), 6.50 (s, 4H), 5.74 (d, *J* = 7.2 Hz, 4H), 4.75 (t, *J* = 8.2 Hz, 4H), 4.41 (d, *J* = 7.2 Hz, 4H), 3.43 (t, *J* = 6.5 Hz, 8H), 2.34 (q, *J* = 8.0 Hz, 8H), 1.66 (p, *J* = 6.5 Hz, 8H). ^{13}C{^{1}H} NMR (75 MHz, $CDCl_3$): δ 155.0, 138.0, 120.2, 116.8, 99.5, 51.3, 36.1, 27.5, 27.0. *MALDI-TOF MS m/z:* Calcd for $C_{44}H_{44}N_{12}O_8Ag^+$: $[M+Ag]^+$ 975.245; Found 975.301. Anal. Calcd for $C_{44}H_{44}N_{12}O_8{\bullet}2H_2O$: C, 58.40; H, 5.35. Found: C, 58.52; H, 4.96.

Synthesis of tetrakis(ammonium) cavitand **1**

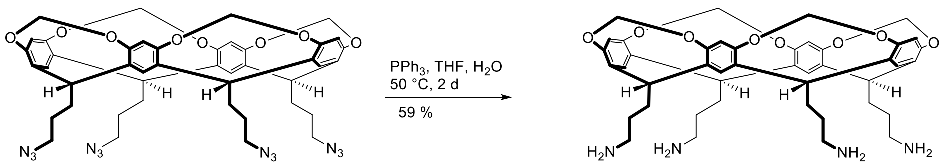

6 **1a**

<u>*Synthesis of **1a**.*</u> Tetra-azido cavitand **6** (434 mg, 0.5 mmol) was added to a dry 100 mL RBF containing 30 mL anhydrous THF. To this was added 1.05 g (4 mmol, 8 equiv.) PPh_3 and the reaction stirred for 2 h at 55 °C (oil bath temperature). After this time, distilled H_2O (0.8 mL, 80 equiv.) was added, and the solution heated to 55 °C for a further 2 d (oil bath temperature). The solution was then cooled to rt, filtered, and then the solvent removed under reduced pressure. The solids were dried for 3 h at rt, taken up in minimal chloroform, and triturated with hexanes. The collected solids were then washed with additional hexanes to obtain 227 mg **1a** as a white solid (59 %). ^{1}H NMR (400 MHz, DMSO-d_6): δ 7.53 (s, 4H), 6.47 (s, 4H), 5.67 (d, *J* = 7.7 Hz, 4H), 4.50 (t, *J* = 8.1 Hz, 4H), 4.34 (d, *J* = 7.7 Hz, 4H), 3.27 – 2.72 (m, 8H), 2.61 (t, *J* = 7.3 Hz, 8H), 2.37 – 2.25 (m, 8H), 1.37 – 1.25 (m, 8H). ^{13}C{^{1}H} NMR (75 MHz, DMSO-d_6): δ 154.5, 138.7, 122.9, 116.9, 99.4, 42.0, 36.6, 32.3, 26.8.

<u>*Conversion to the ammonium chloride salt **1**.*</u> **1a** was taken up in 20 mL chloroform and extracted with 0.5 % HCl (2 × 10 mL). The aqueous layer was then washed once with hexanes and lyophilized to

give product **1** as the ammonium salt. M + 4HCl. (quant., Figure S13–Figure S24). ^{1}H NMR (400 MHz, DMSO-d_6): δ 8.21 (s, 8H), 7.80 (s, 4H), 6.55 (s, 4H), 5.73 (d, *J* = 7.6 Hz, 4H), 4.53 (t, *J* = 8.1 Hz, 4H), 4.41 (d, *J* = 7.7 Hz, 4H), 2.99 – 2.86 (m, 28H), 2.81 – 2.62 (m, 13H), 1.71 – 1.49 (m, 8H). ^{1}H NMR (500 MHz, D_2O): δ 7.44 (s, 4H), 6.62 (s, 4H), 5.73 (d, *J* = 7.6 Hz, 4H), 4.62 (t, *J* = 8.2 Hz, 4H), 4.27 (d, *J* = 7.7 Hz, 4H), 3.04 (t, *J* = 7.9 Hz, 8H), 2.45 – 2.37 (m, 8H), 1.63 (p, *J* = 8.0 Hz, 8H). ^{13}C{^{1}H} NMR (75 MHz, DMSO-d_6): δ 154.5, 138.7, 122.9, 116.9, 99.4, 42.0, 36.6, 32.3, 26.8. *ESI-MS* m/z: $[M{-}4HCl{+}H]^+$ Calcd for $C_{44}H_{53}N_4O_8$ 765.3858; Found 765.4080. $[M{-}4HCl{+}2H]^{2+}$ Calcd for $C_{44}H_{54}N_4O_8$ 383.1965; Found 383.2170. $[M{-}4HCl{+}3H]^{3+}$ Calcd for $C_{44}H_{55}N_4O_8$ 255.8001; Found 255.8150. Anal. Calcd for $C_{44}H_{56}N_4Cl_4O_8{\bullet}H_2O{\bullet}2HCl$: C, 51.83; H, 6.13. Found: C, 52.04; H, 5.78.

Synthesis of tetrakis(guanidinum) chloride cavitand **2**

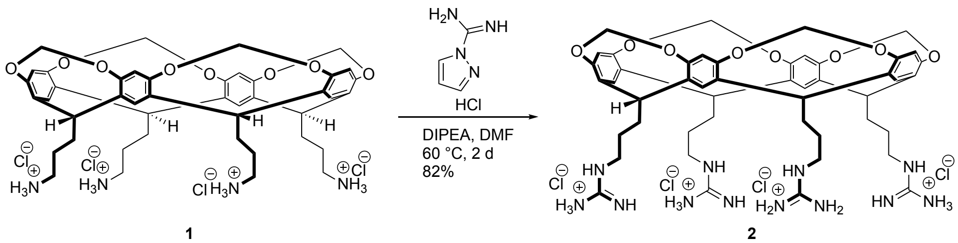

1 2

Tetra-ammonium **1** (100 mg, 0.131 mmol) was added to a dry 50 mL RBF containing 10 mL of anhydrous DMF. The suspension was sonicated and pyrazole-1-carboximide (115 mg, 0.78 mmol, 6 equiv.) was added followed by 0.273 mL (12 equiv.) of diisopropylethylamine. The reaction was then heated to 60 °C for 48 h (oil bath temperature). After this time, the DMF was removed under reduced pressure and dried overnight at rt. The product was suspended in 5 mL of distilled H_2O and 1.5 mL (5 equiv.) of 2 M sodium perchlorate ($NaClO_4$) was added to precipitate the product as the perchlorate (ClO_4^-) salt. The product was then centrifuged in a microcentrifuge for 5 min (15,000 rpm) and the liquid removed. The sodium perchlorate precipitation was repeated twice more to remove any side product and the final solid pellets were combined. The perchlorate salt was then resuspended in 20 ml water, and 10 g DOWEX® Chloride anion exchange resin was added, and the suspension was run through a DOWEX® Chloride anion exchange. The product was passed through another DOWEX® anion exchange resin to assure total conversion to the chloride salt and the final product lyophilized to give a white solid (116 mg, 82%). (Figure S25–Figure S31). ^{1}H NMR (500 MHz, DMSO): δ 8.02 (s, 1H), 7.82 (s, 1H), 7.51 (s, 2H), 7.11 (s, 2H), 6.55 (s, 1H), 5.74 (d, *J* = 7.7 Hz, 1H), 4.59 (t, *J* = 8.3 Hz, 1H), 4.42 (d, *J* = 7.6 Hz, 1H), 3.27 (d, *J* = 6.3 Hz, 2H), 2.64 (d, *J* = 10.6 Hz, 2H), 1.48 (t, *J* = 7.8 Hz, 2H). ^{1}H NMR (400 MHz, D_2O): δ 7.46 (s, 1H), 6.45 (s, 1H), 5.59 (d, *J* = 7.5 Hz, 1H), 4.47 (t, *J* = 8.1 Hz, 1H), 4.13 (d, *J* = 7.7 Hz, 1H), 3.10 (t, *J* = 6.8 Hz, 2H), 2.39 (s, 2H), 1.44 (s, 2H). ^{13}C{^{1}H} NMR (101 MHz, DMSO): δ 157.5, 154.6, 138.4, 123.4, 117.1, 99.4, 41.1, 40.5 40.3, 40.1, 39.9, 39.7, 39.5, 39.3, 36.4, 27.3, 26.7. ^{13}C{^{1}H} NMR (101 MHz, D_2O): δ 156.6, 154.3, 138.0, 121.9, 116.8, 100.1, 41.1, 36.2, 26.4, 26.1. ESI-MS m/z: $[M{-}4Cl]^{4+}$ Calcd for $C_{48}H_{64}N_{12}O_8$ 234.1237; Found 234.1227. Anal. Calcd for $C_{48}H_{64}Cl_4N_{12}O_8{\bullet}3H_2O$: C, 50.89; H, 6.23. Found: C, 50.78; H, 5.97.

Data & Results

3. Spectral Data

Tetrahydroxy cavitand **4**

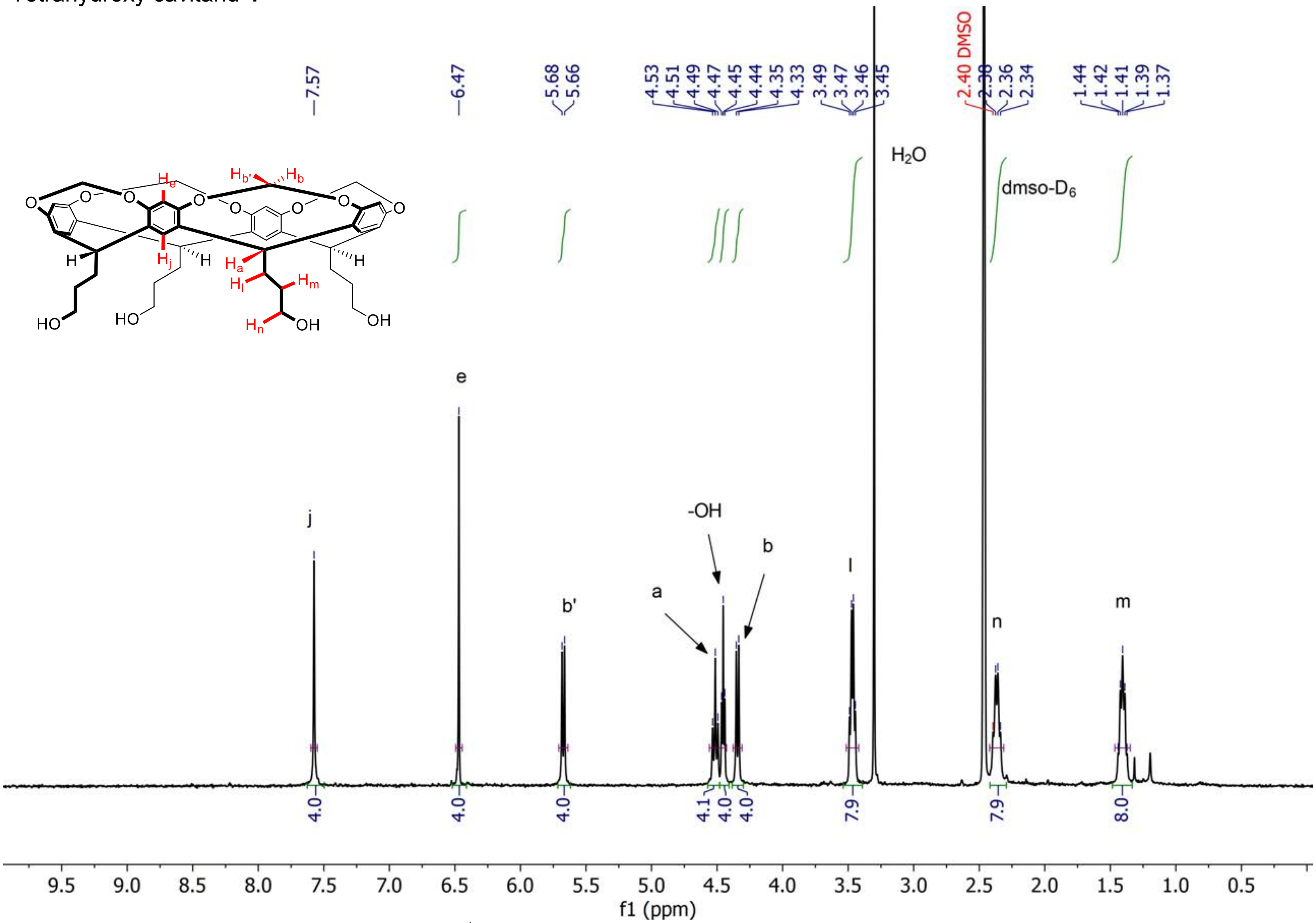

Figure S1. ^{1}H NMR spectrum (DMSO-d_6) of tetra-hydroxy cavitand **4**.

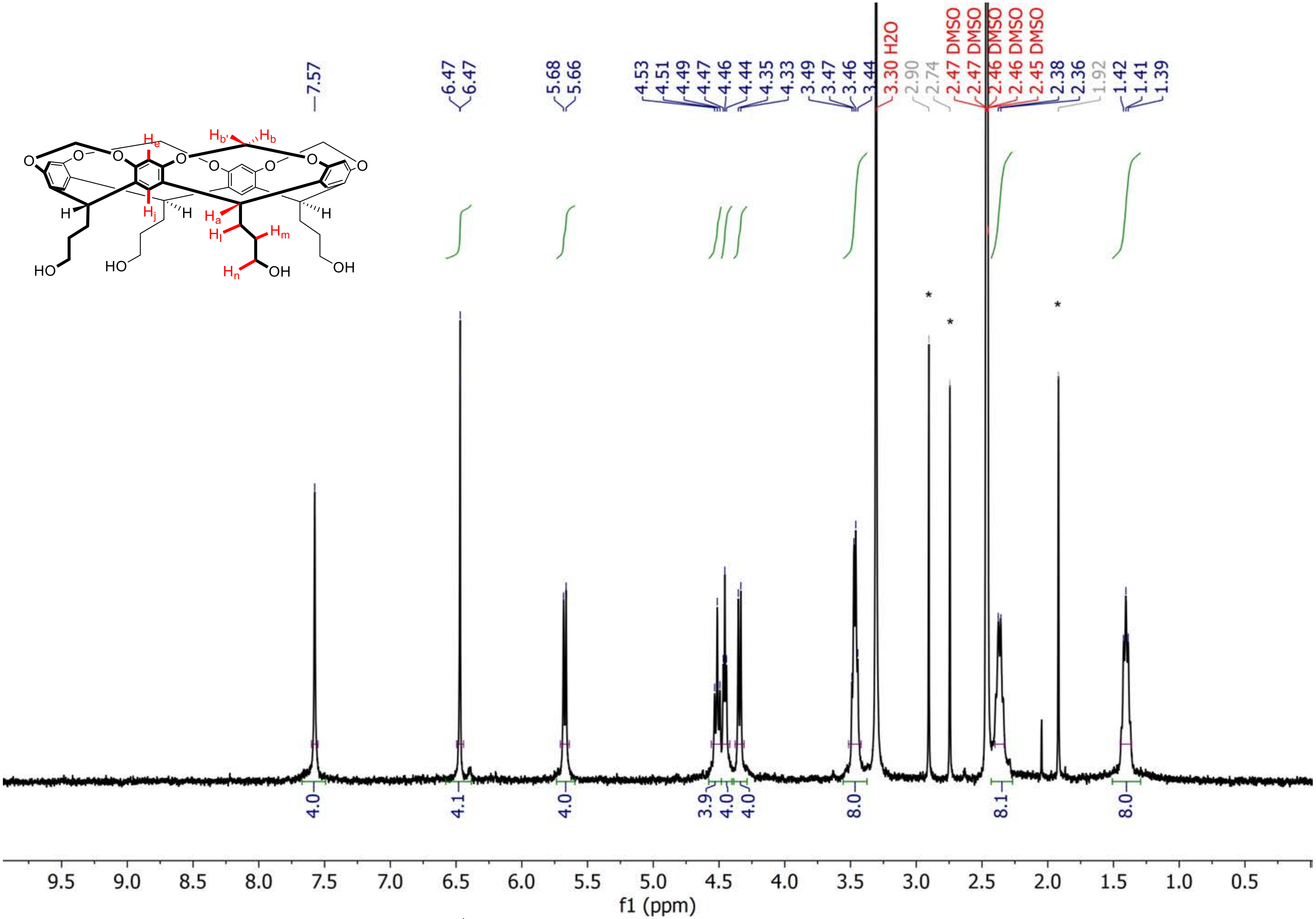

Figure S2. ^{1}H NMR spectrum (DMSO-d_6) of crude tetra-hydroxy cavitand **4**.

Tetrabomide cavitand **5**

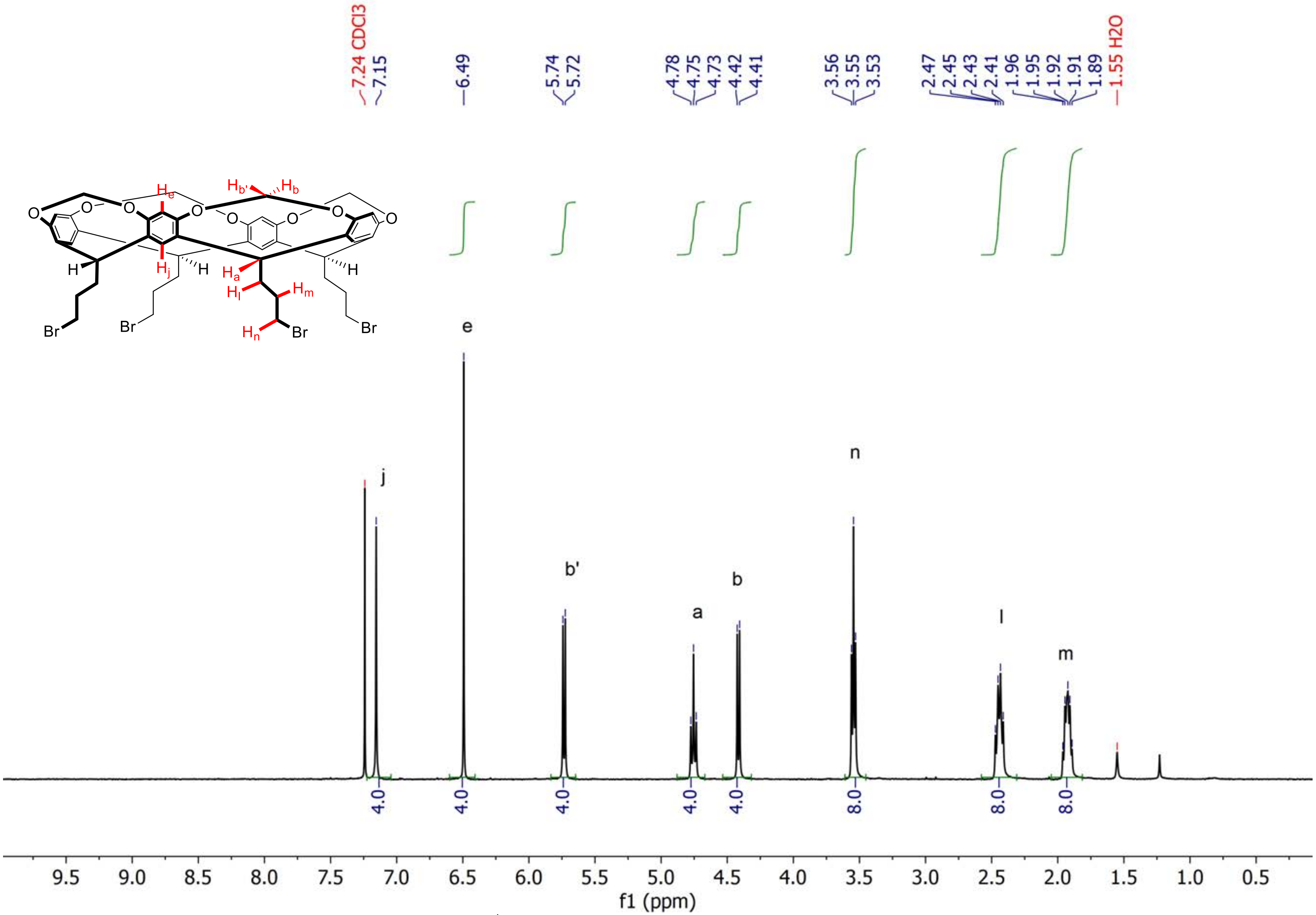

Figure S3. ^{1}H NMR spectrum ($CDCl_3$) of tetra-bromo cavitand **5.**

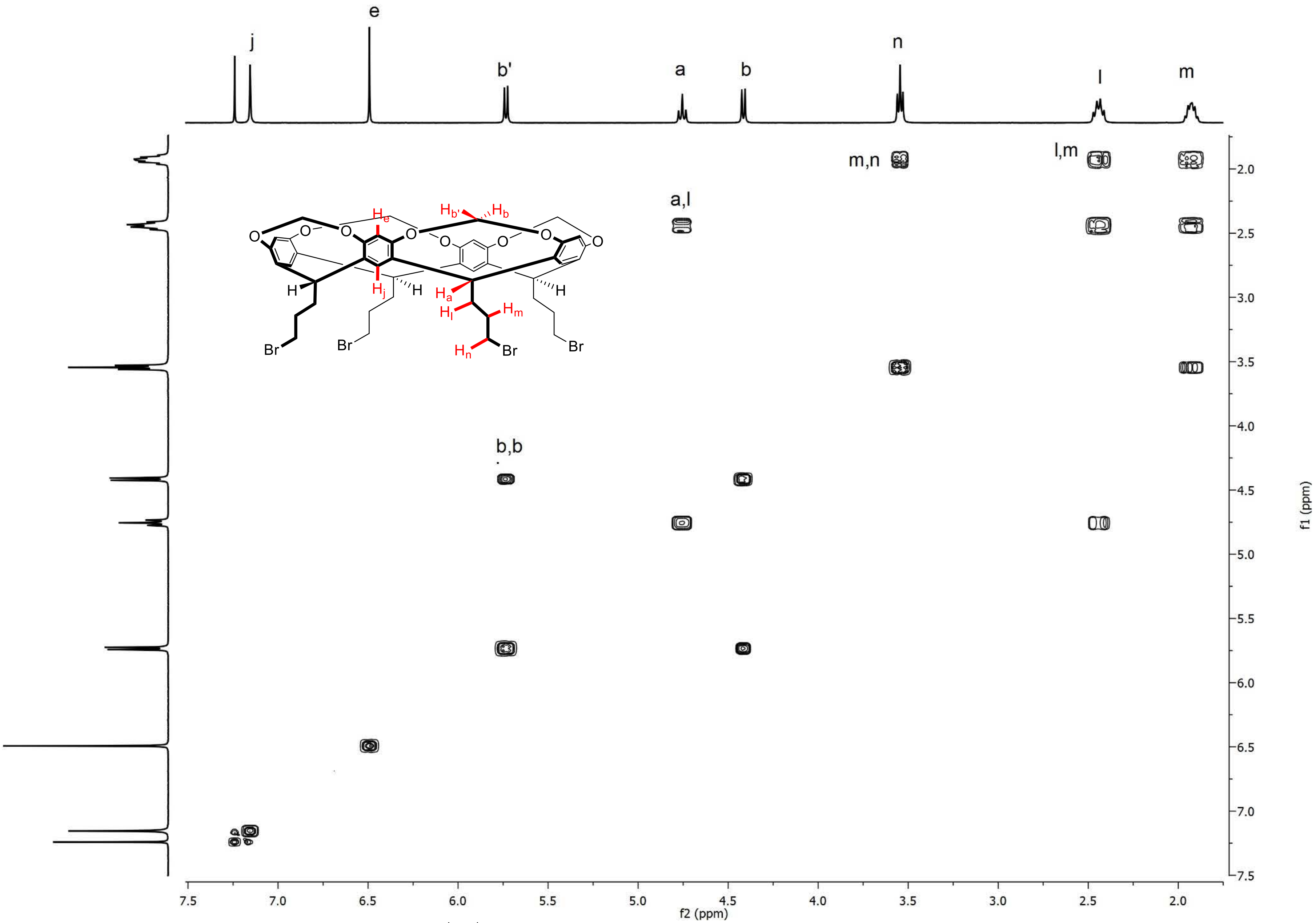

Figure S4. ^{1}H–^{1}H (COSY) NMR spectrum ($CDCl_3$) of tetra-bromo cavitand **5.**

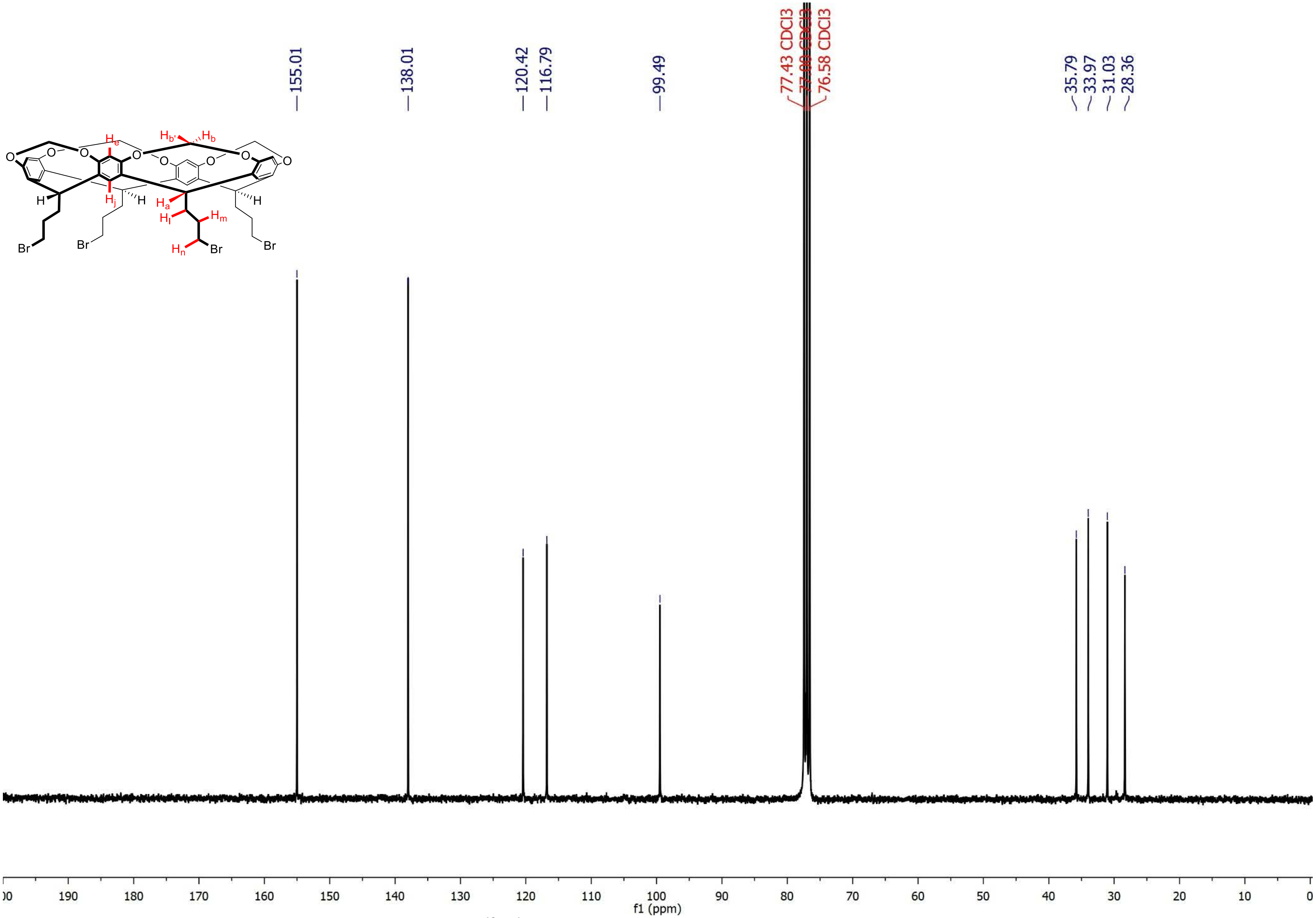

Figure S5. $^{13}C\{^1H\}$ NMR spectrum ($CDCl_3$) of tetra-bromo cavitand **5**.

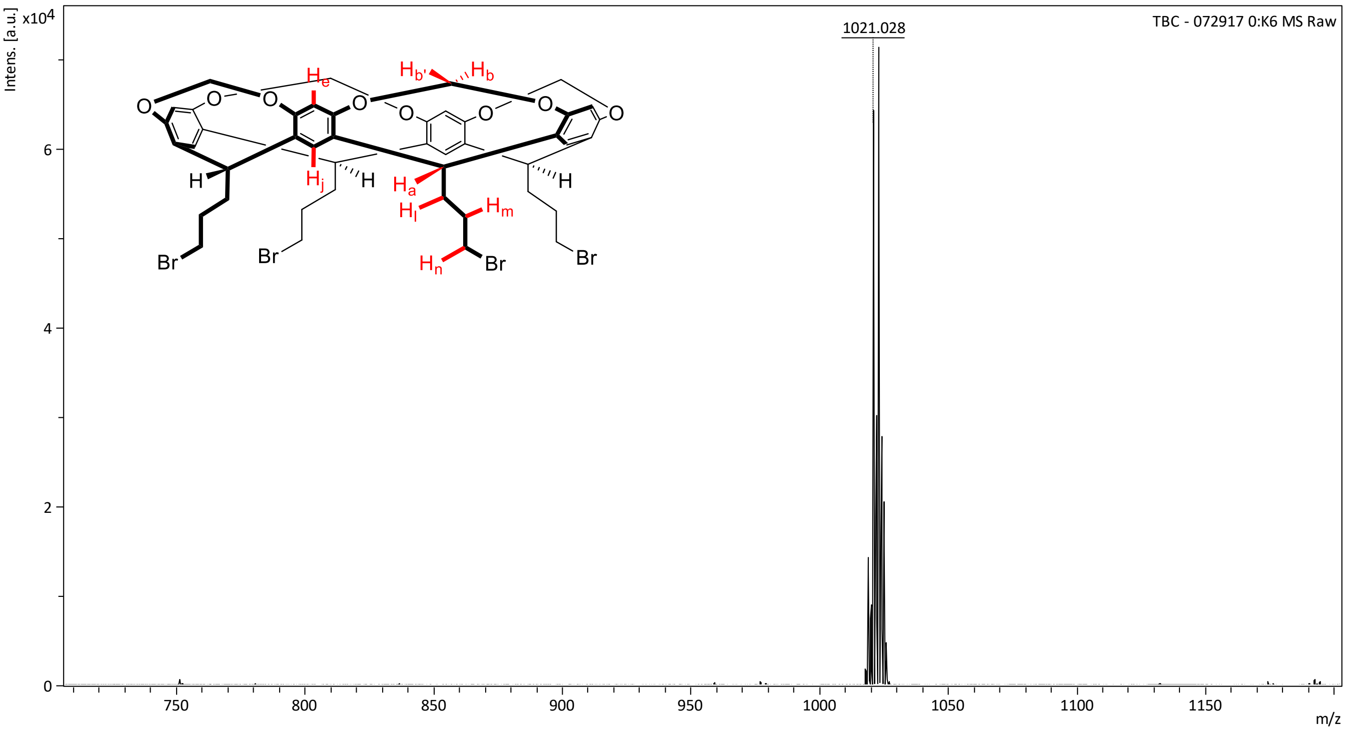

Figure S6. MALDI-TOF MS of tetra-bromo cavitand **5**; (DCTB, 2:1) no counter-ion, 50% v/v $CHCl_3$-CH_3CN and 0.1 % formic acid.

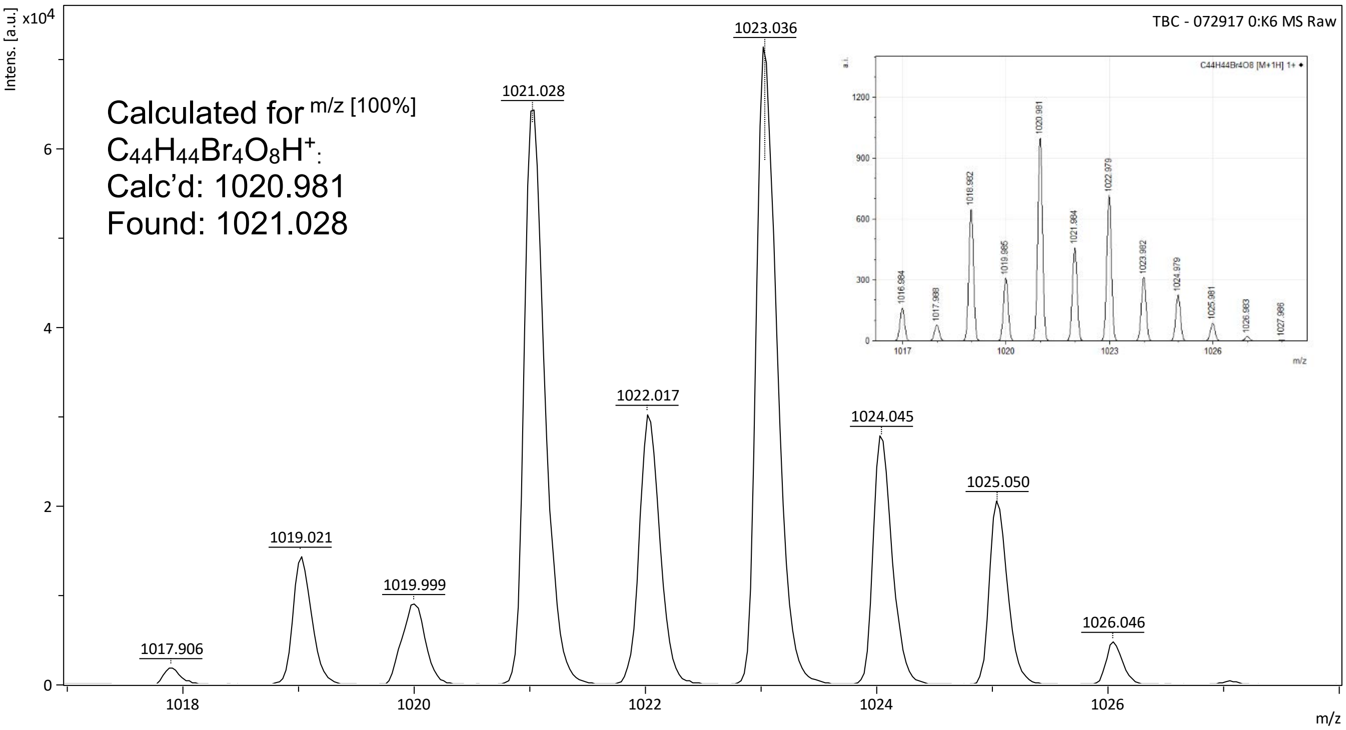

Figure S7. Expanded view of tetra-bromo cavitand **5**, $[M+H]^+$, with theoretical calculation inset.

Tetra(azido) cavitand **6**

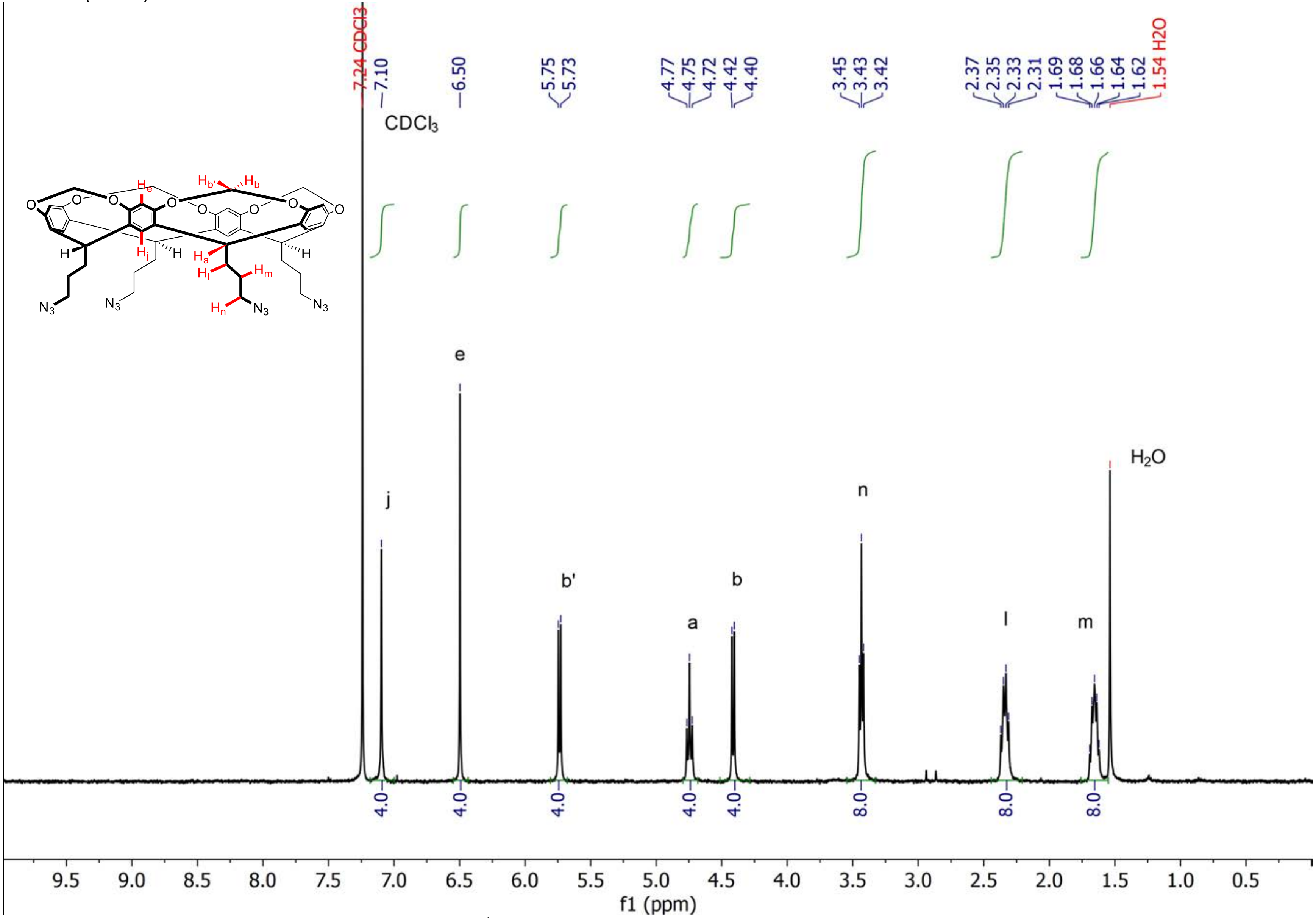

Figure S8. ^{1}H NMR spectrum ($CDCl_3$) of tetrakis(azido) cavitand **6.**

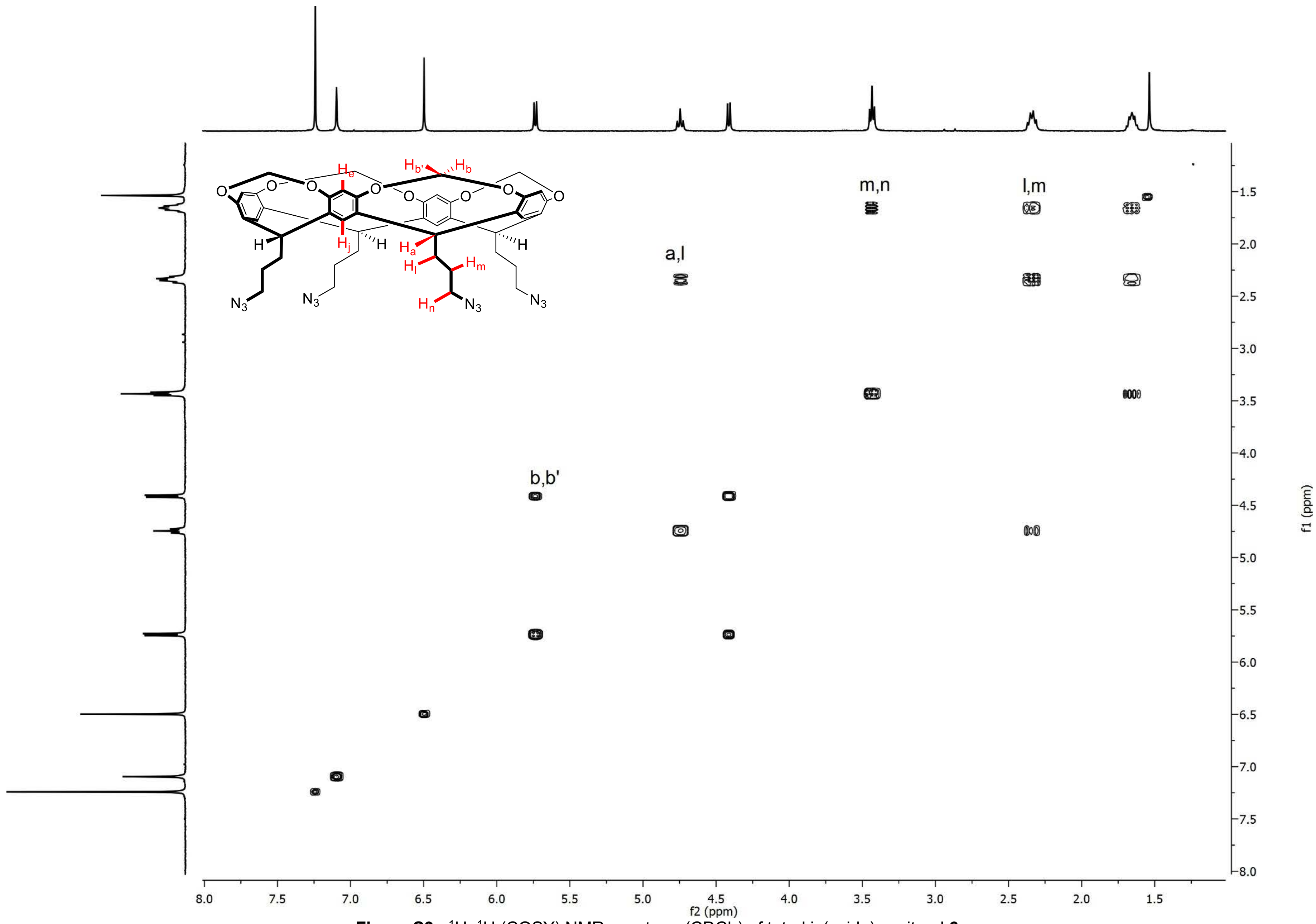

Figure S9. ^{1}H–^{1}H (COSY) NMR spectrum ($CDCl_3$) of tetrakis(azido) cavitand **6.**

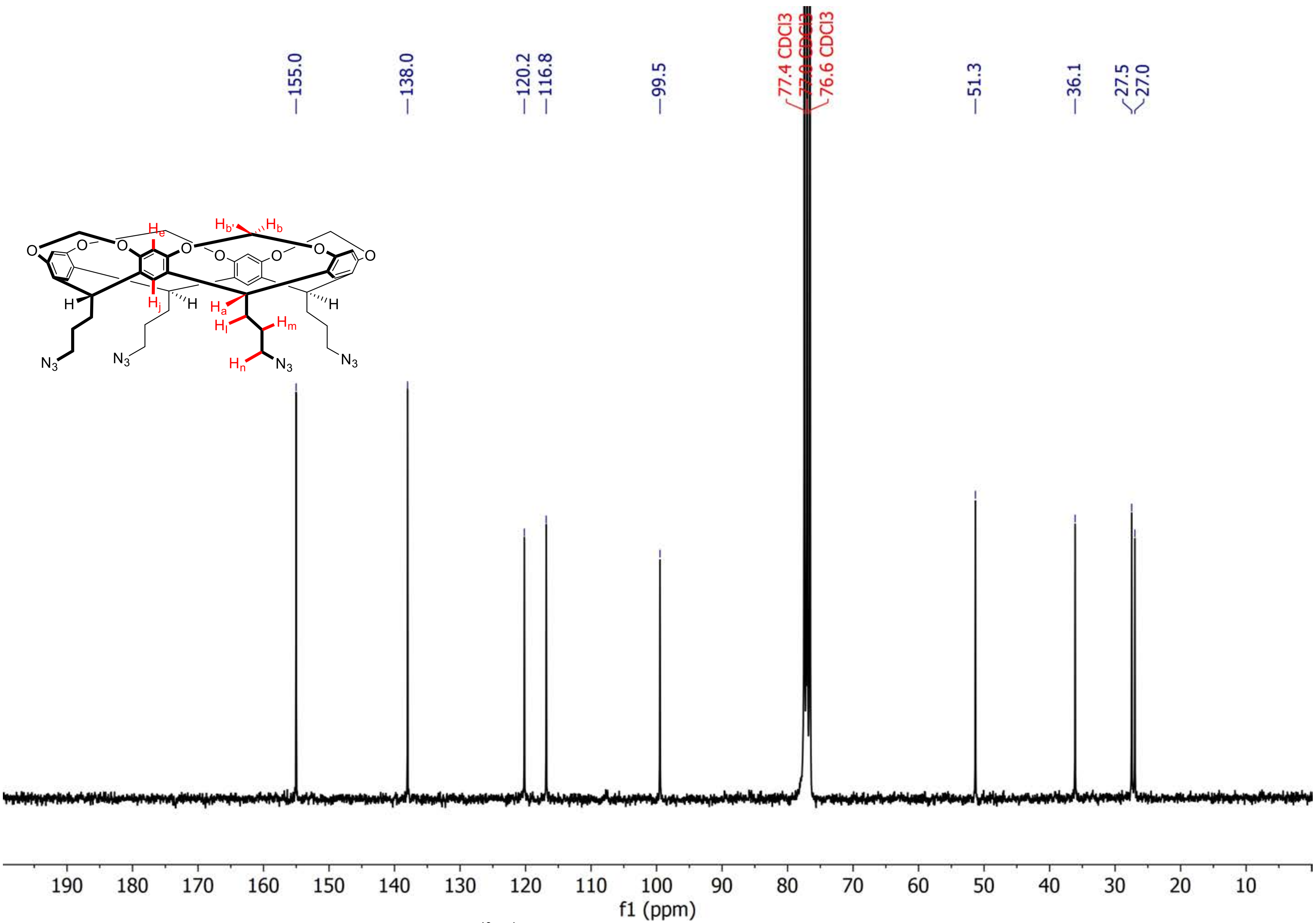

Figure S10. $^{13}C\{^1H\}$ NMR spectrum ($CDCl_3$) of tetrakis(azido) cavitand **6.**

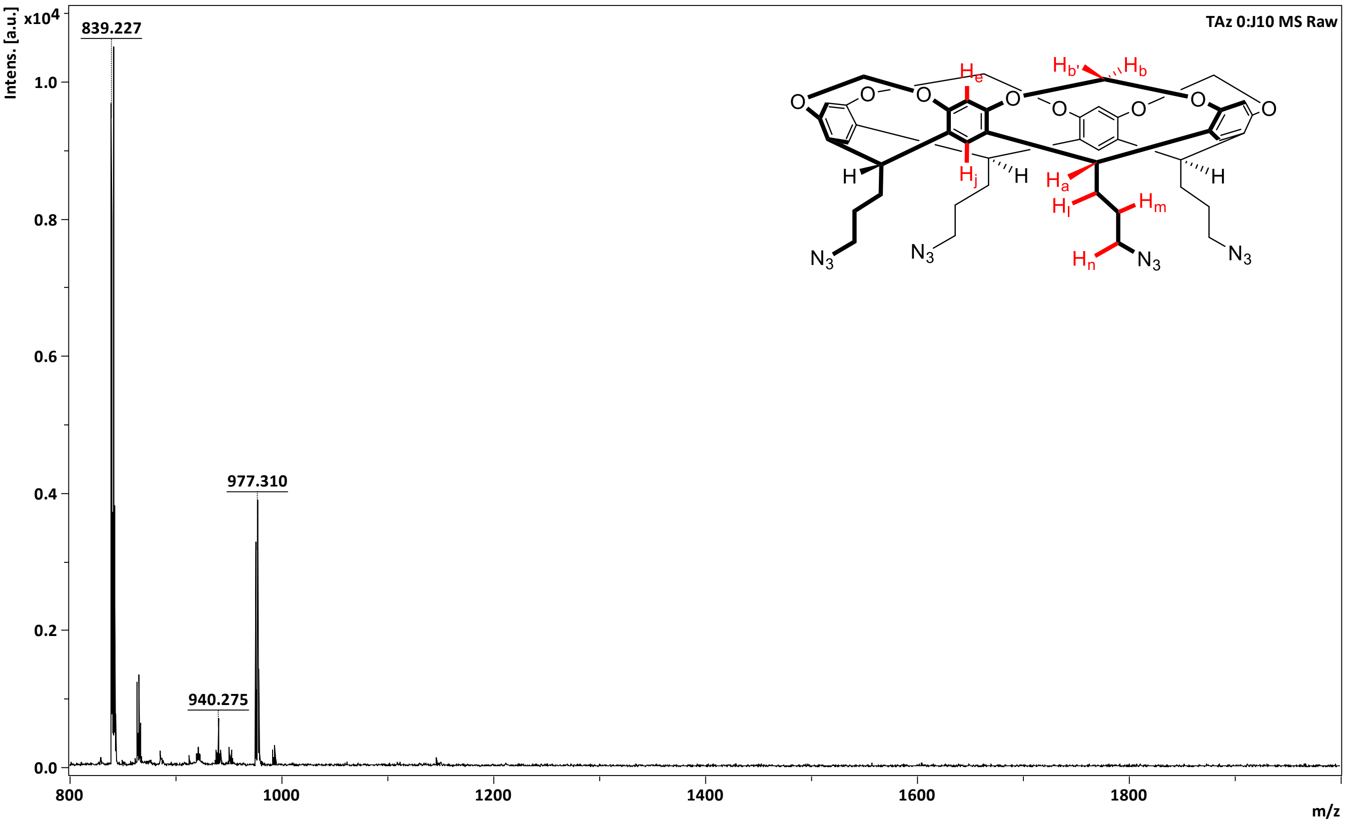

Figure S11. MALDI-TOF MS of tetra-azido cavitand **6**; (DCTB, 2:1:1) $Ag^{+}CF_3CO_2^{-}$, THF.

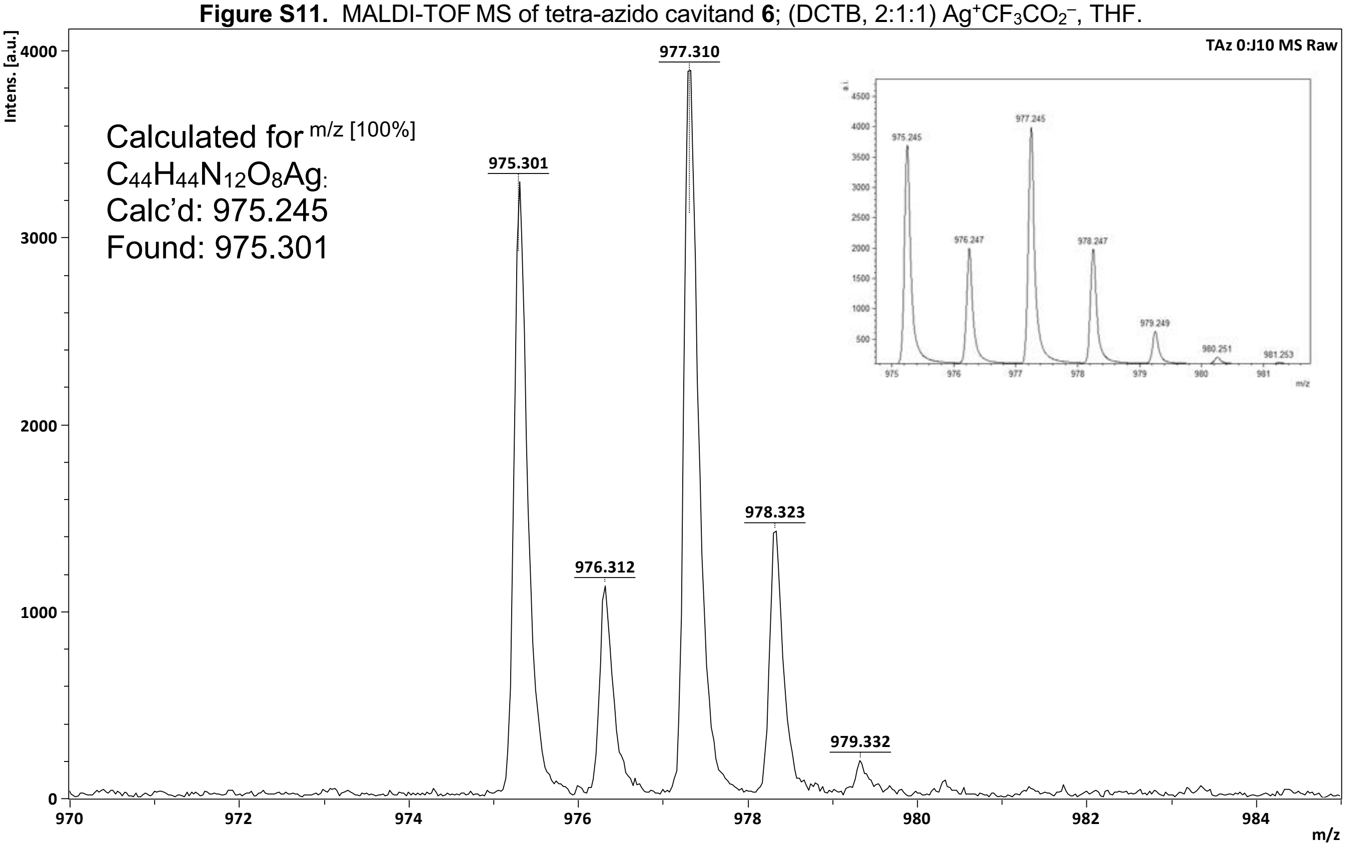

Figure S12. Expanded view of tetra-azido cavitand **6**, $[M+Ag]^{+}$, with theoretical calculation inset.

Tetrakis(amino) cavitand **1a**

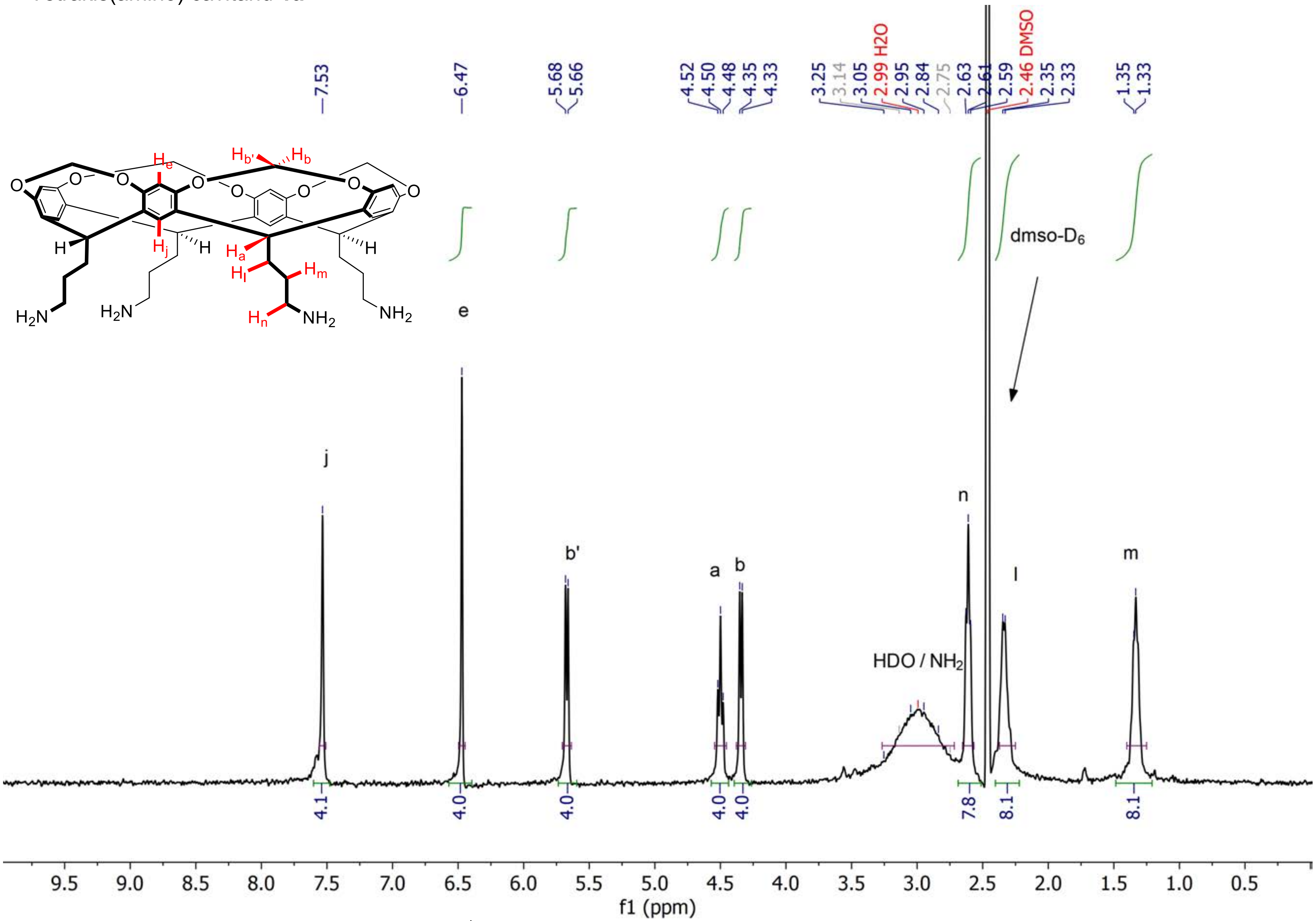

Figure S13. ^{1}H NMR spectrum (DMSO-d_6) of tetrakis(amino) cavitand **1a.**

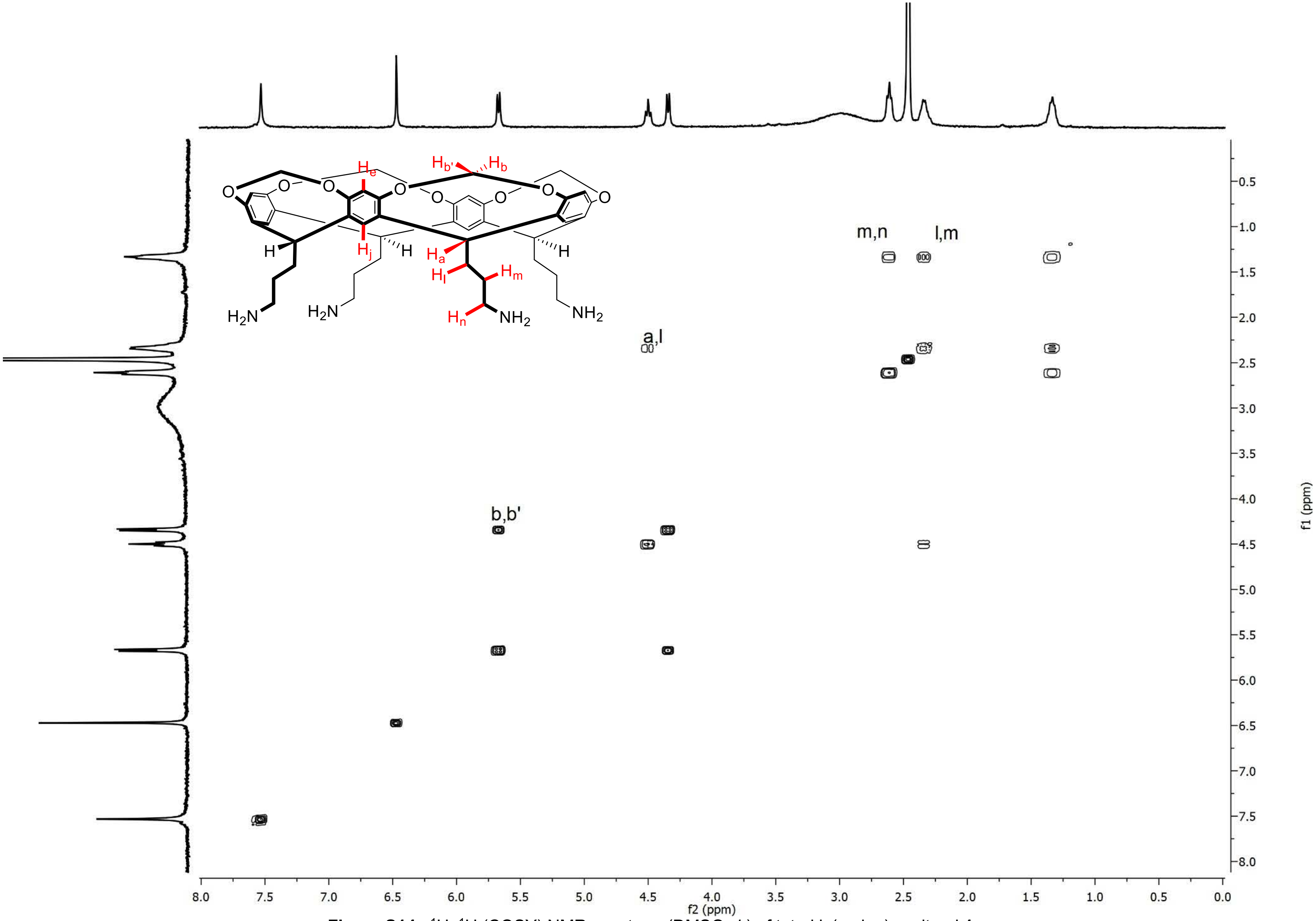

Figure S14. ^1H–^1H (COSY) NMR spectrum (DMSO-d_6) of tetrakis(amino) cavitand **1a**.

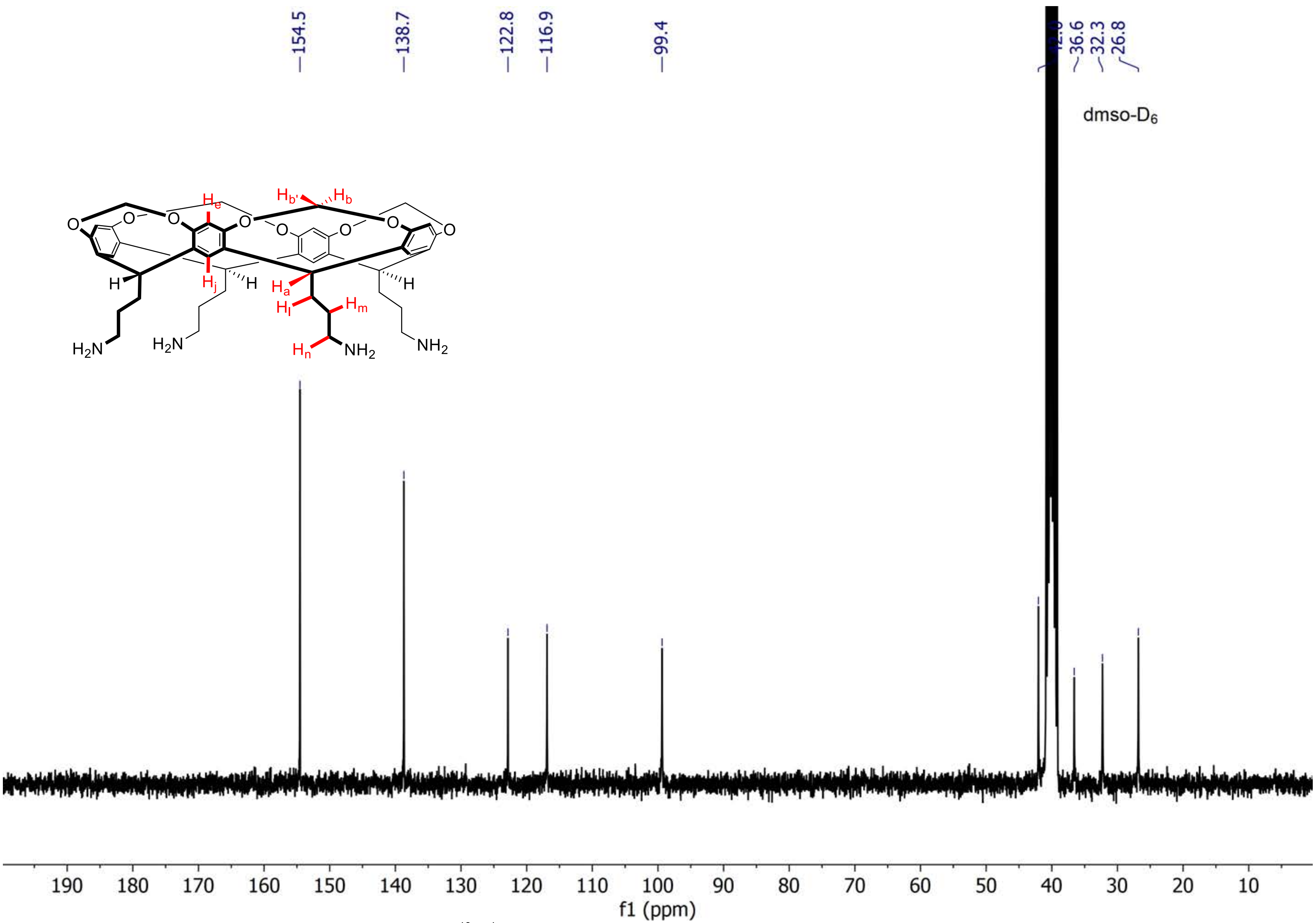

Figure S15. $^{13}C\{^1H\}$ NMR spectrum (DMSO-d_6) of tetrakis(amino) cavitand **1a**.

Tetrakis(ammonium) chloride cavitand **1**

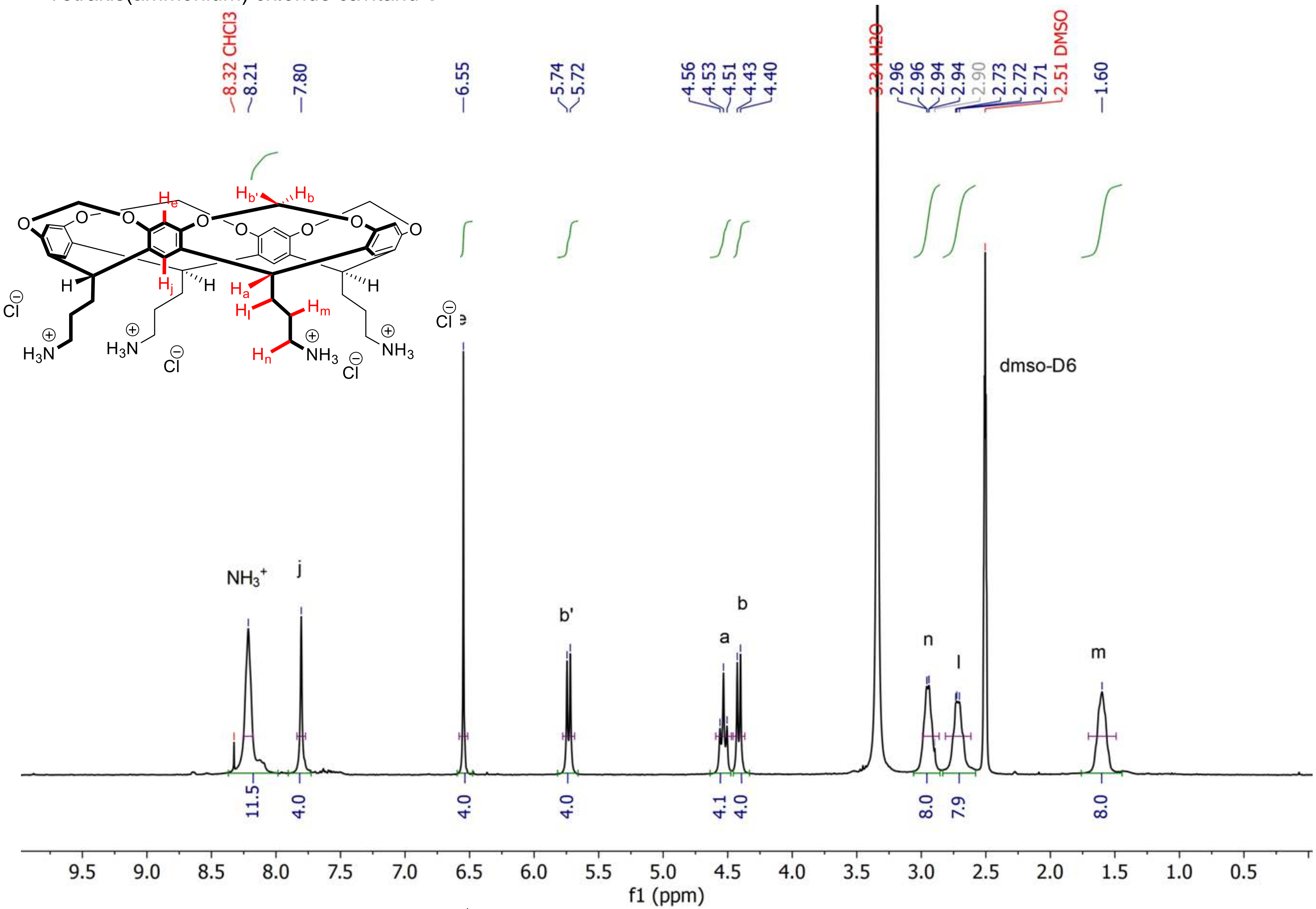

Figure S16. ^{1}H NMR spectrum (DMSO-d_6) of tetrakis(ammonium) cavitand **1.**

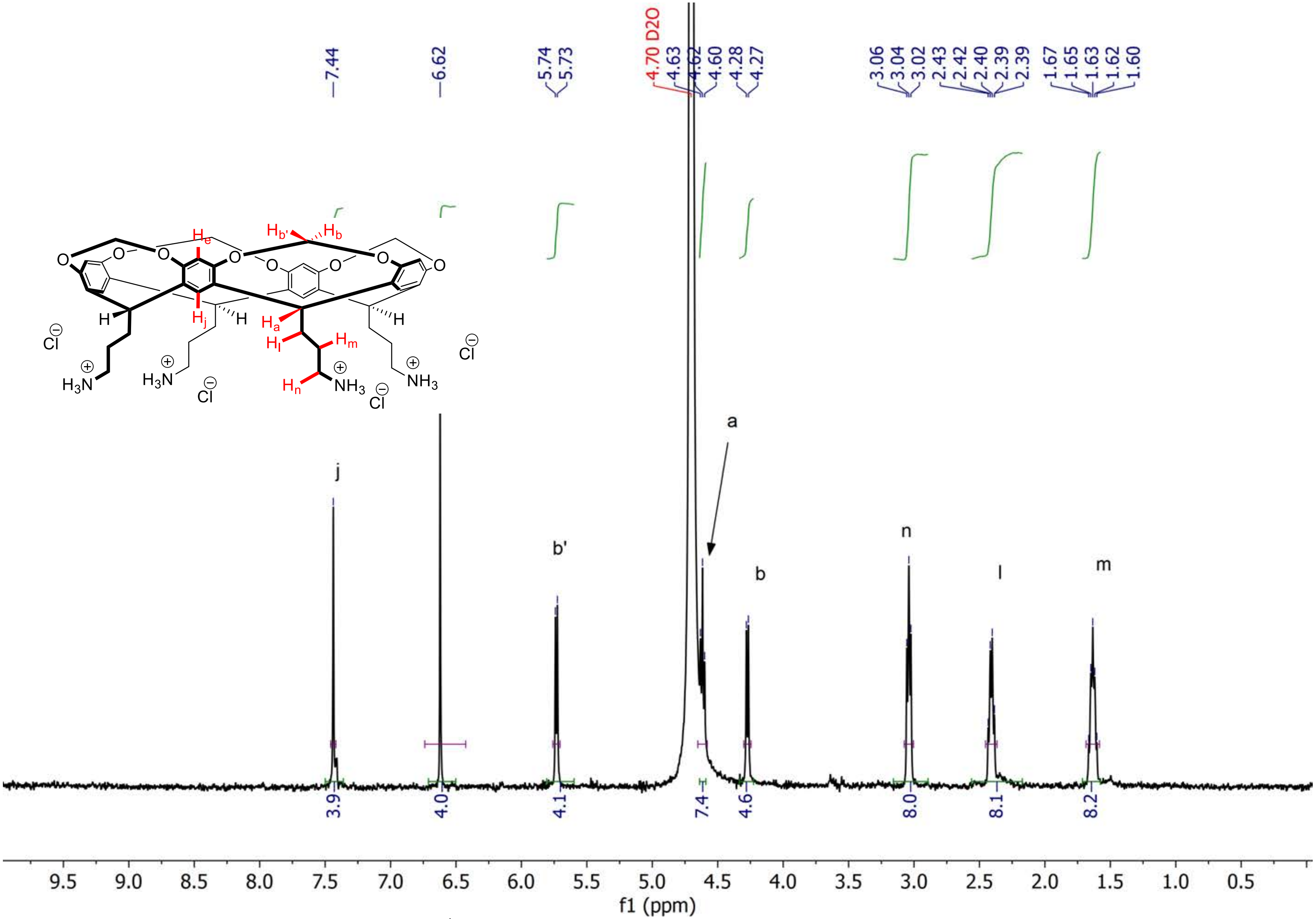

Figure S17. ^{1}H NMR spectrum (D_2O) of tetrakis(ammonium) cavitand **1** (0.4 mM).

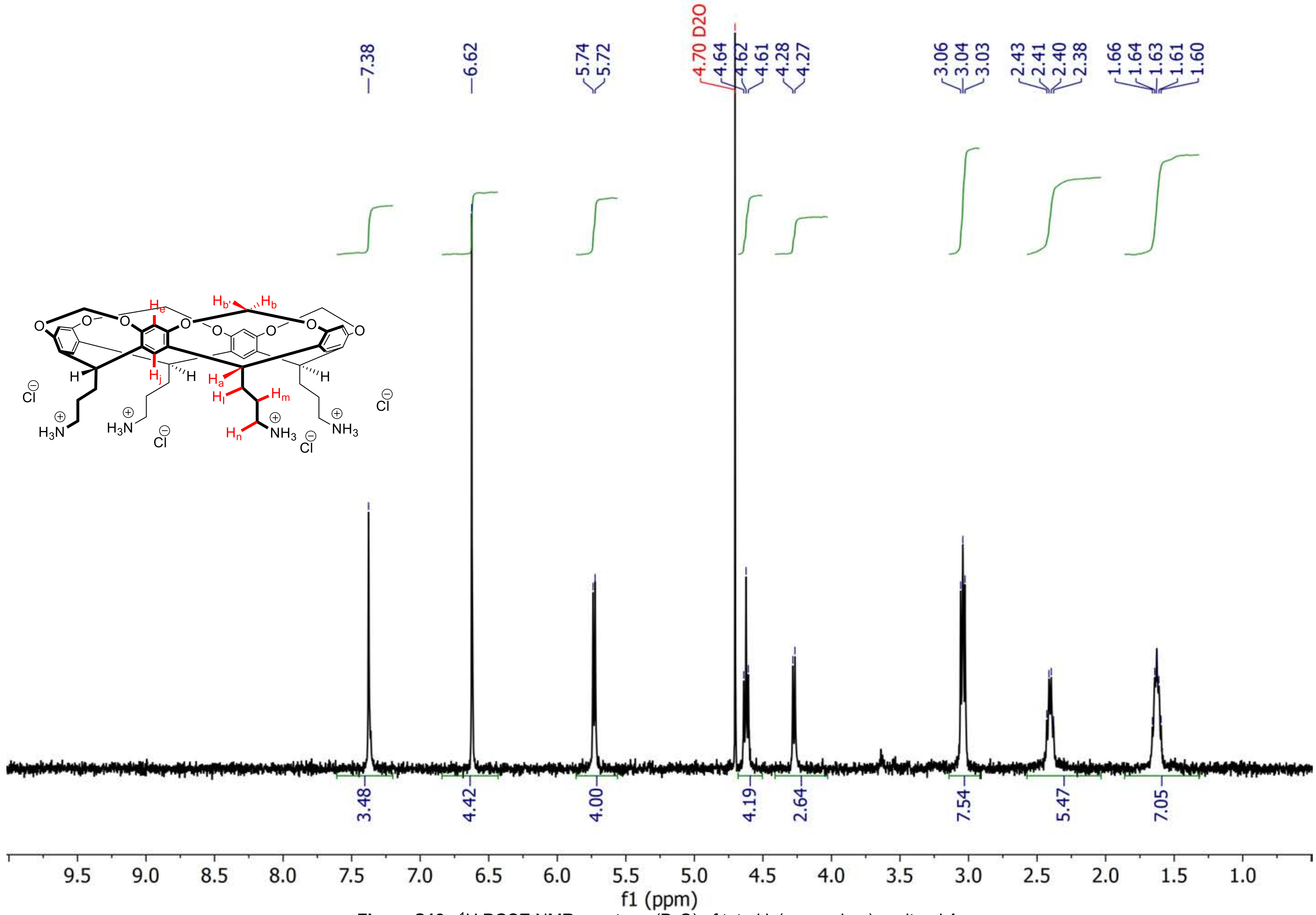

Figure S18. ^{1}H PGSE NMR spectrum (D_2O) of tetrakis(ammonium) cavitand **1.**

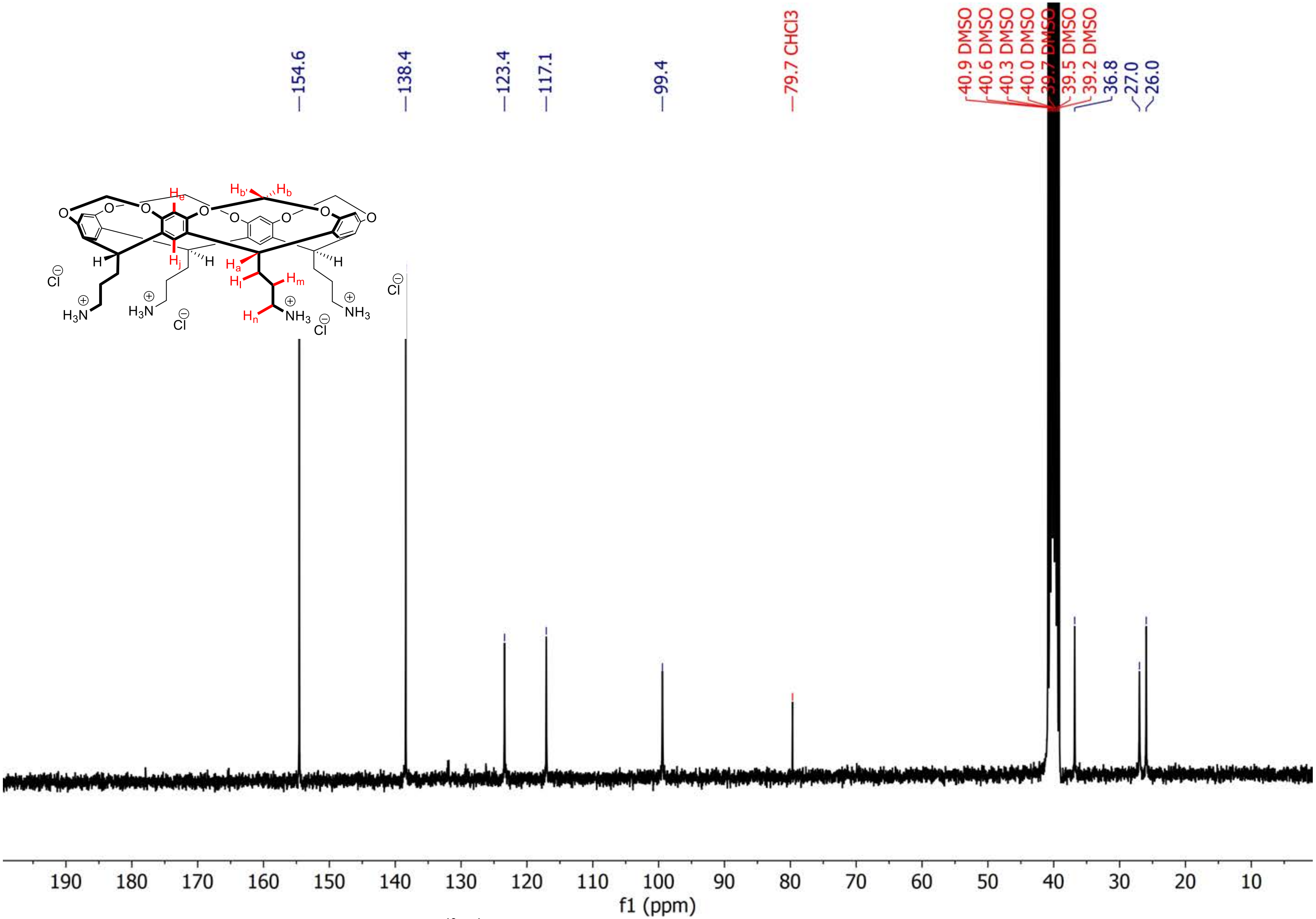

Figure S19. $^{13}C\{^1H\}$ NMR spectrum (DMSO-d_6) of tetrakis(ammonium) cavitand **1**.

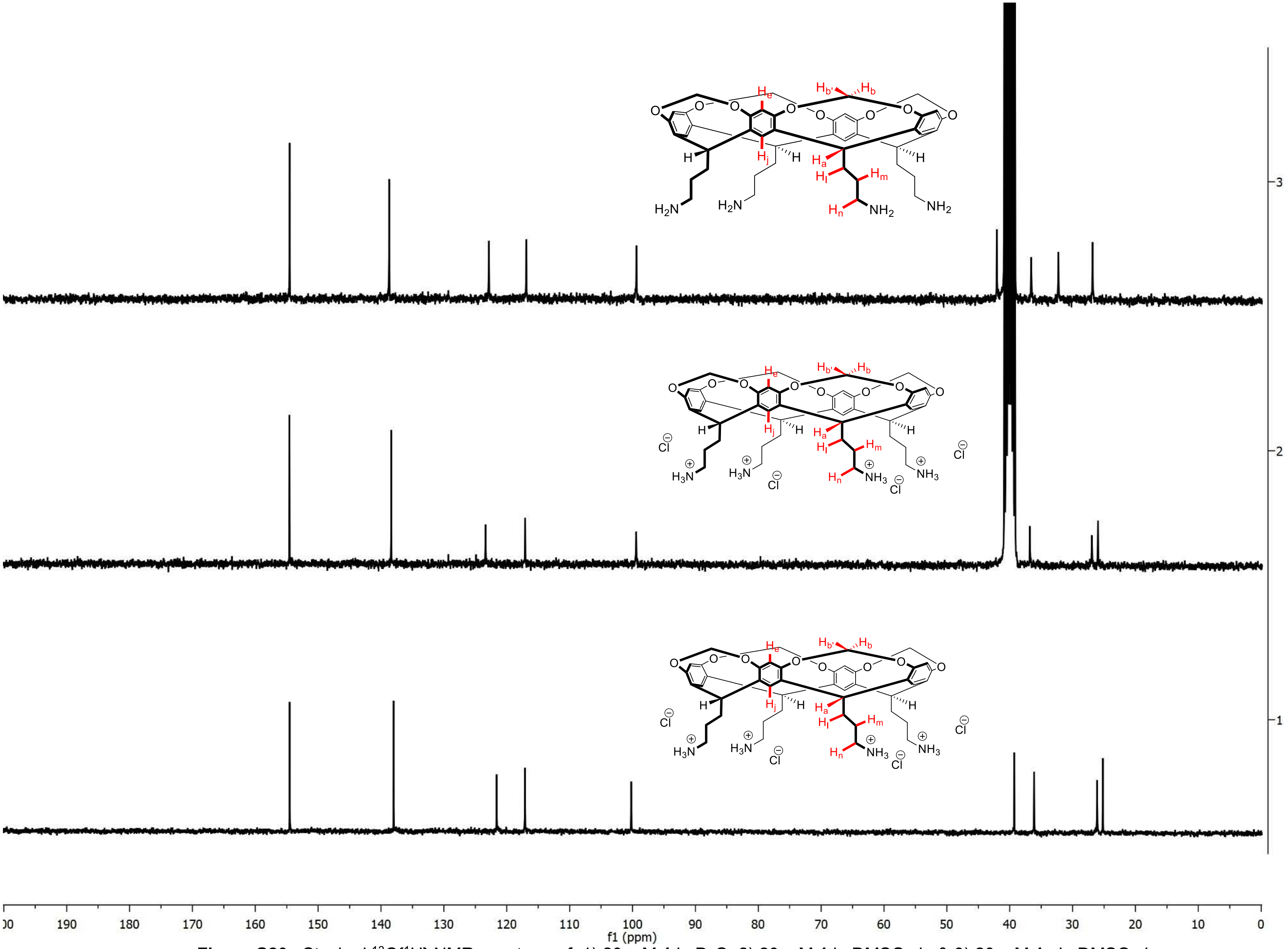

Figure S20. Stacked $^{13}C\{^{1}H\}$ NMR spectrum of: 1) 20 mM **1** in D_2O, 2) 20 mM **1** in DMSO-d_6, & 3) 20 mM **1a** in DMSO-d_6.

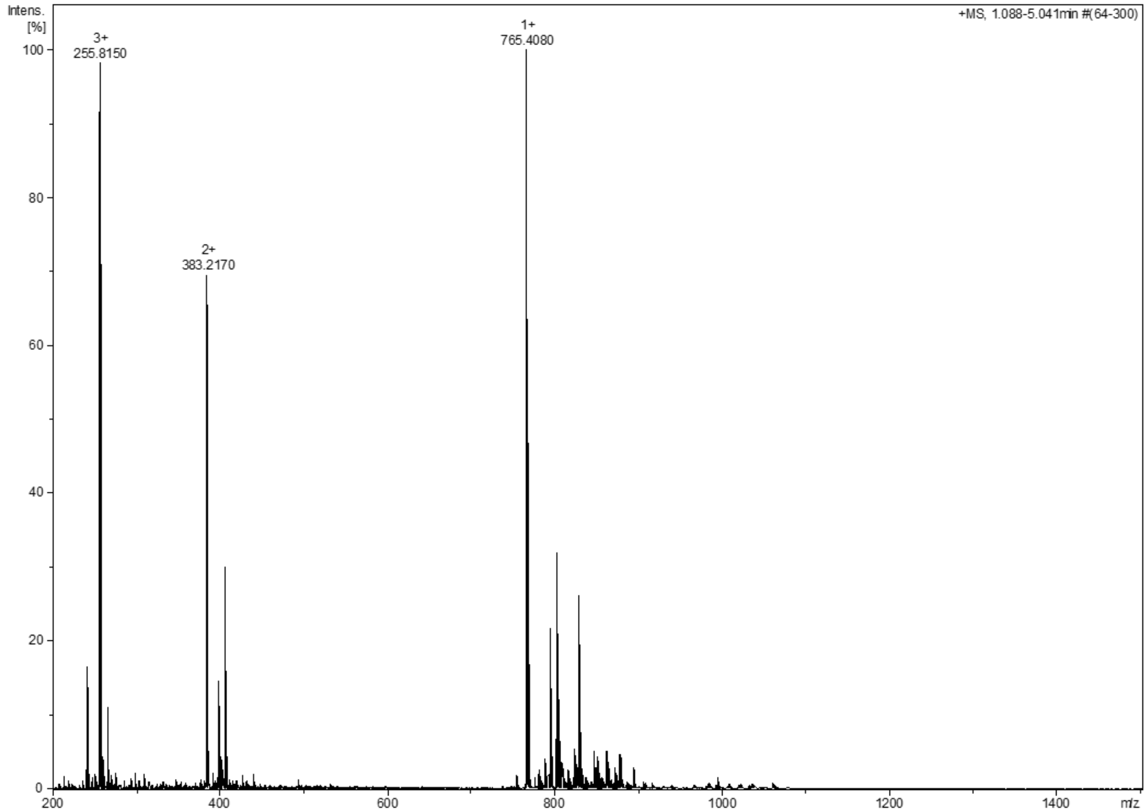

Figure S21. ESI- MS of tetrakis(ammonium) chloride cavitand **1** (20 μM, distilled H_2O).

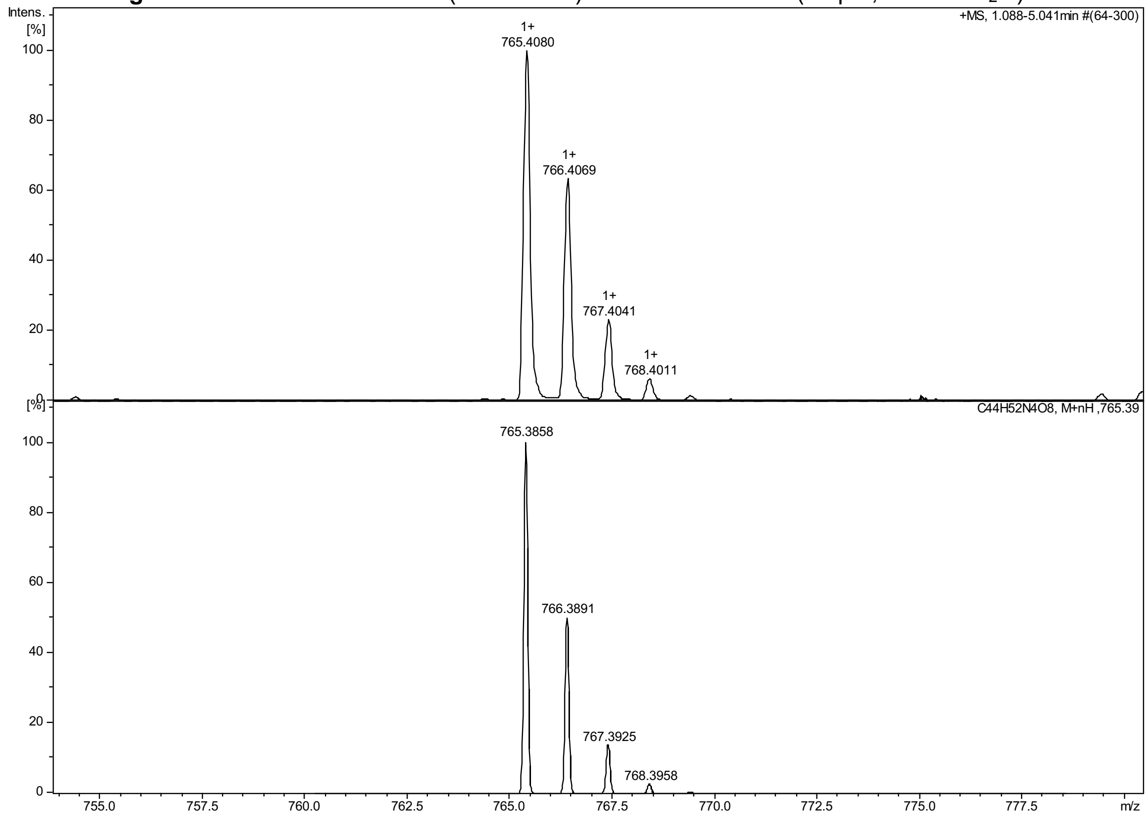

Figure S22. Expanded view of tetrakis(ammonium) chloride cavitand **1**, [M+H]$^+$, with theoretical calculation below.

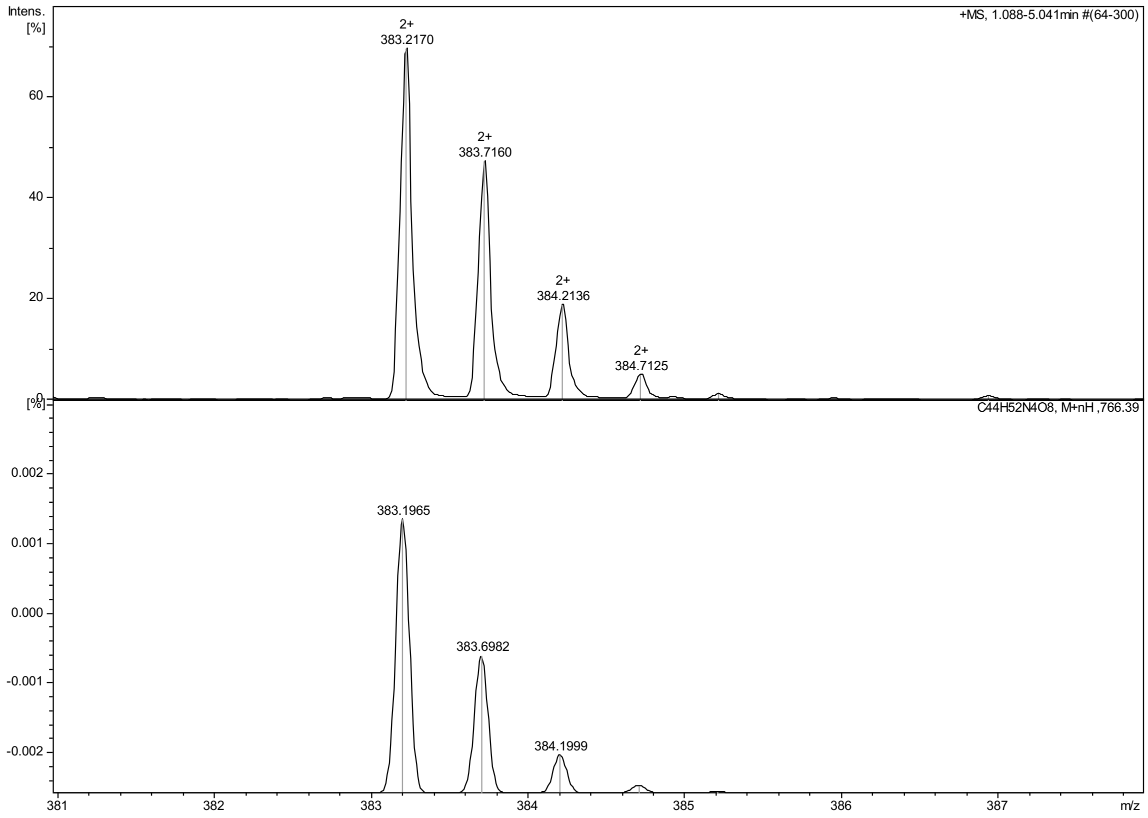

Figure S23. Expanded view of tetrakis(ammonium) chloride cavitand **1**, $[M+2H]^{2+}$, with theoretical calculation below.

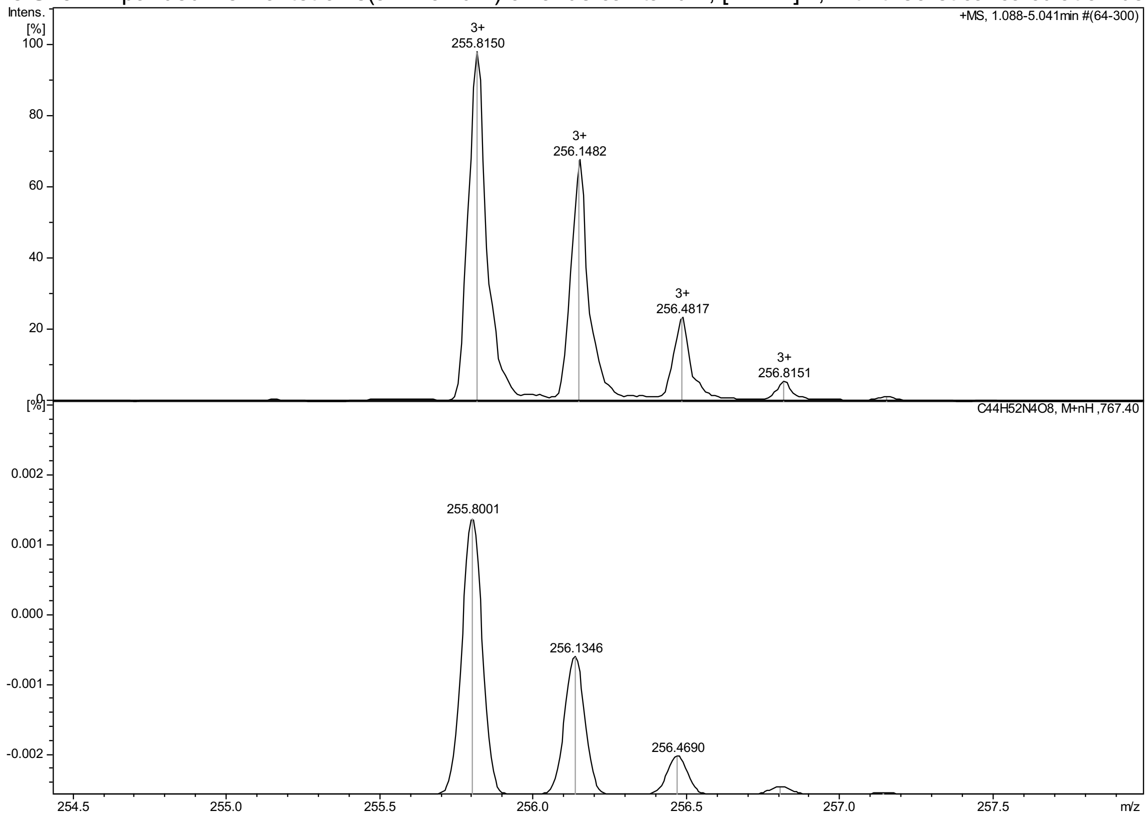

Figure S24. Expanded view of tetrakis(ammonium) chloride cavitand **1**, $[M+3H]^{3+}$, with theoretical calculation below.

Tetrakis(guanidinium) chloride cavitand **2**

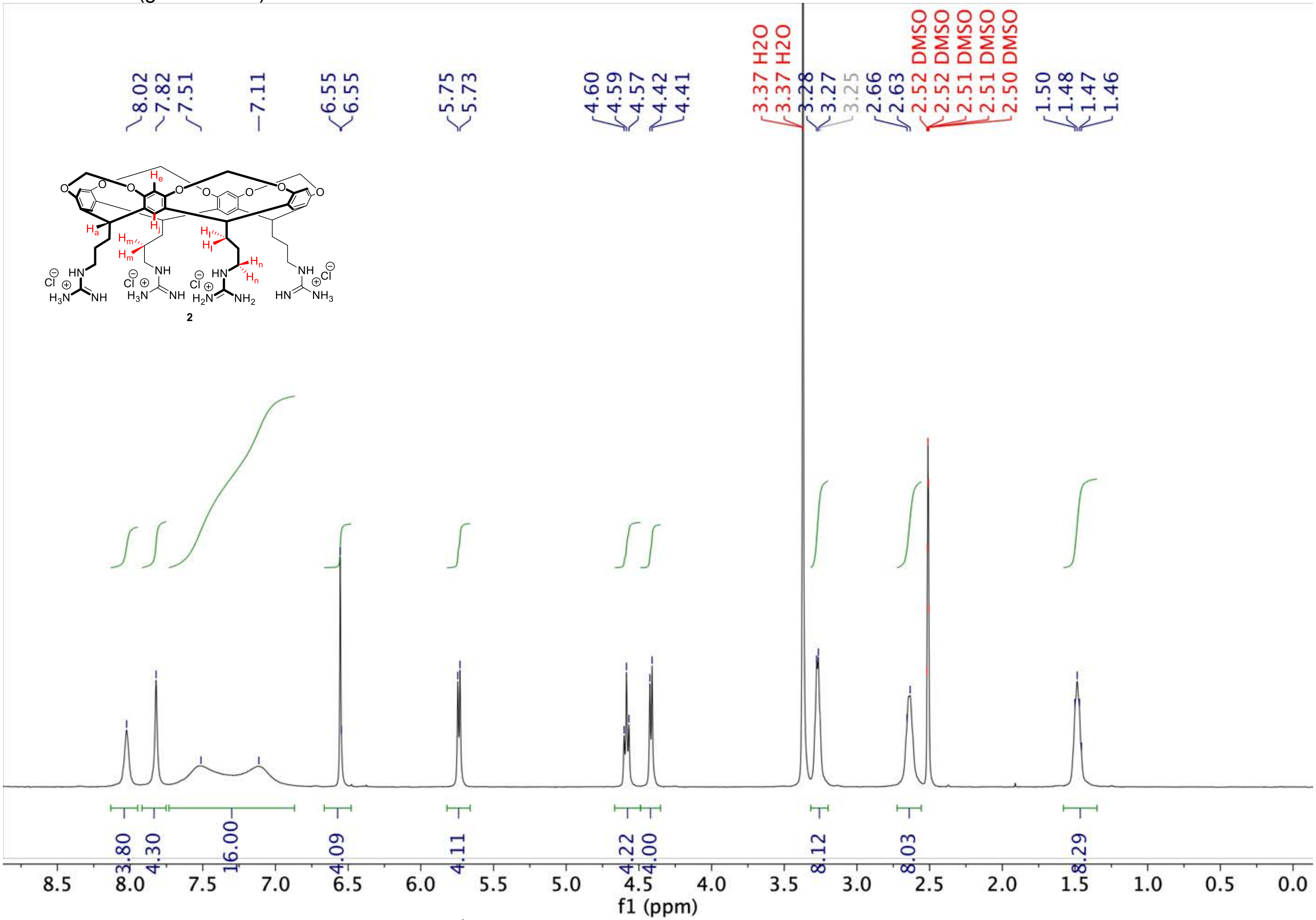

Figure S25. ^{1}H NMR spectrum (DMSO-d_6) of tetrakis(guanidinium) cavitand **2.**

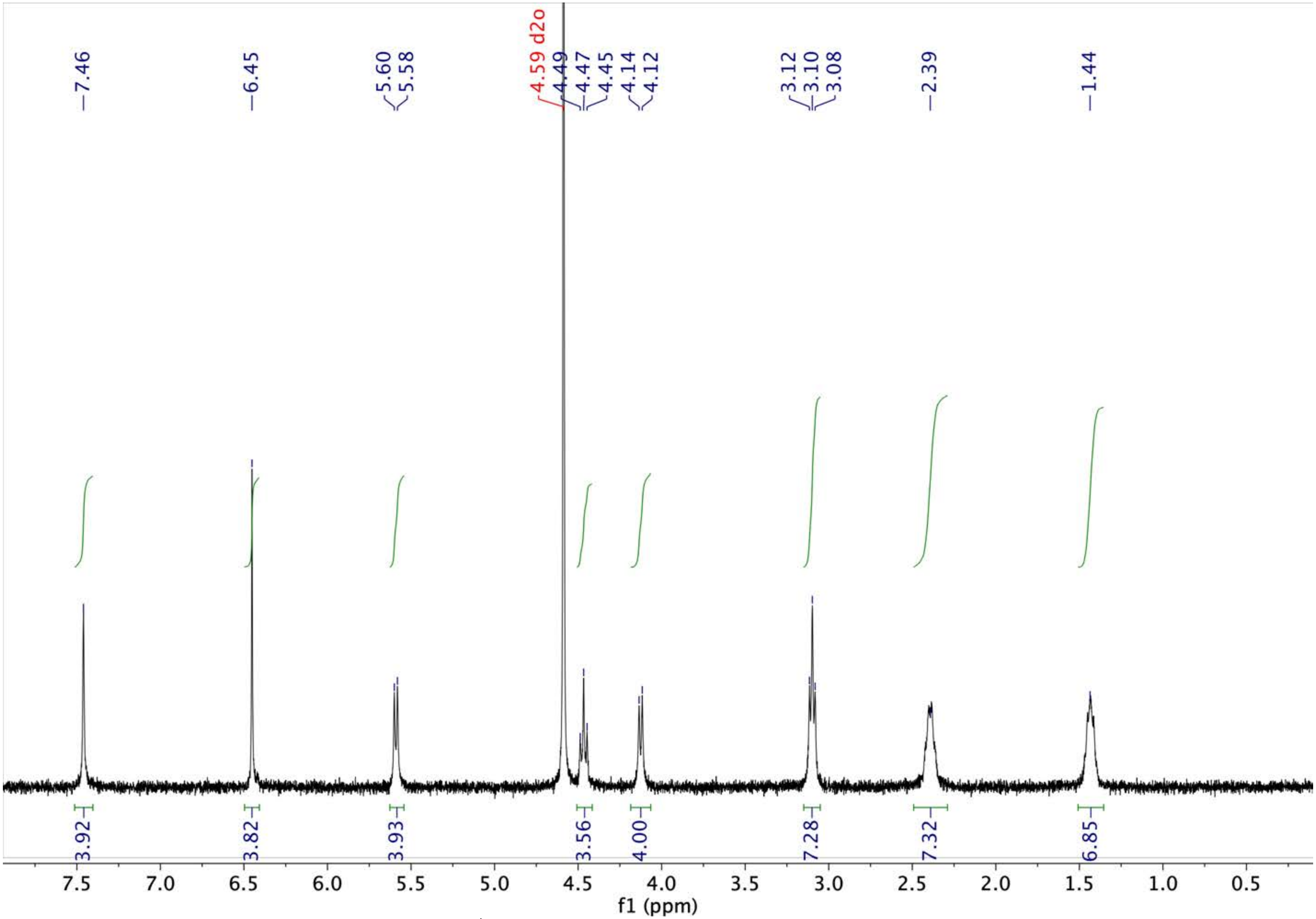

Figure S26. ^{1}H NMR spectrum (D_2O) of tetrakis(guanidinium) cavitand **2.**

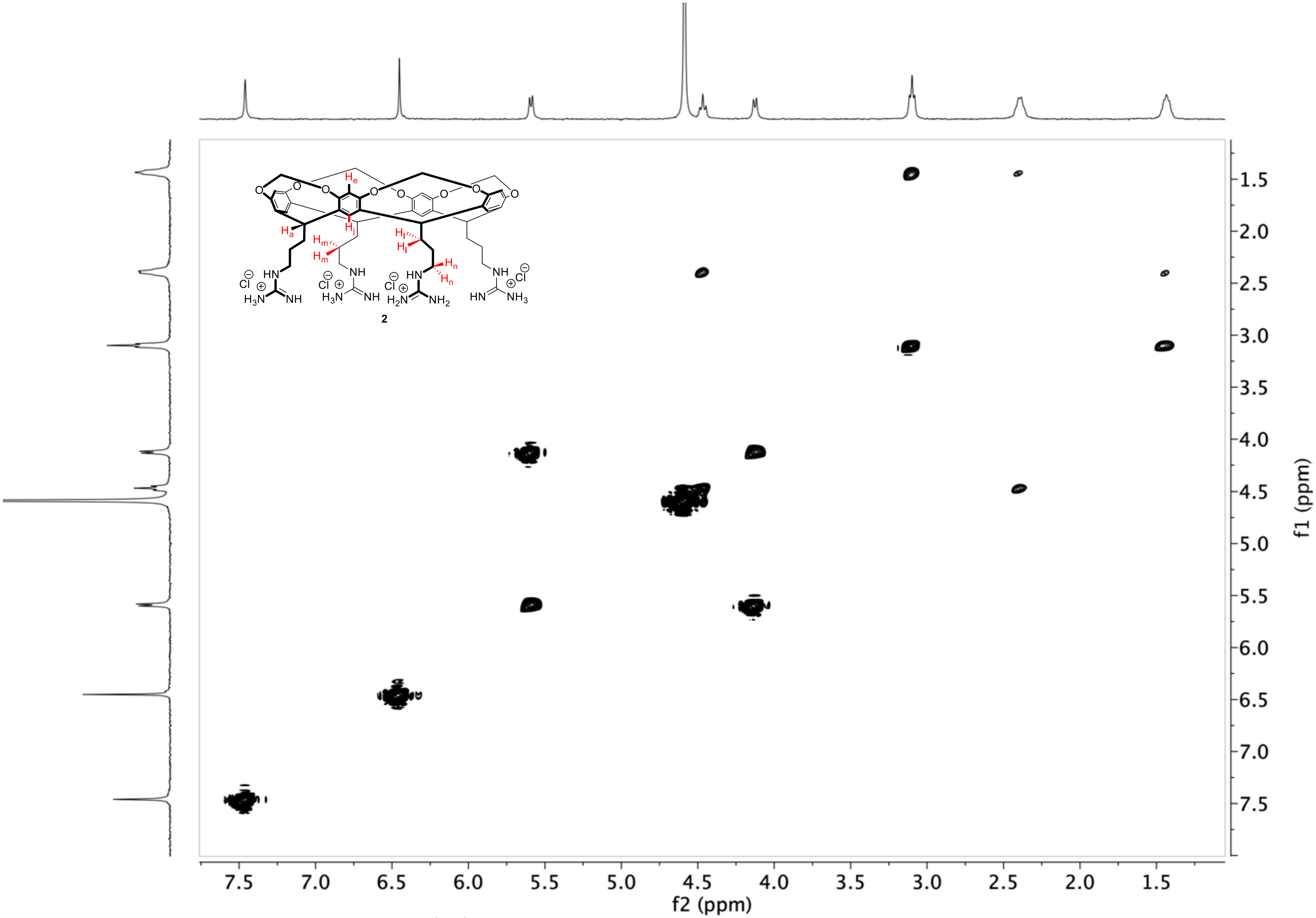

Figure S27. ^{1}H–^{1}H (COSY) NMR spectrum (D_2O) of tetrakis(guanidinium) cavitand **2.**

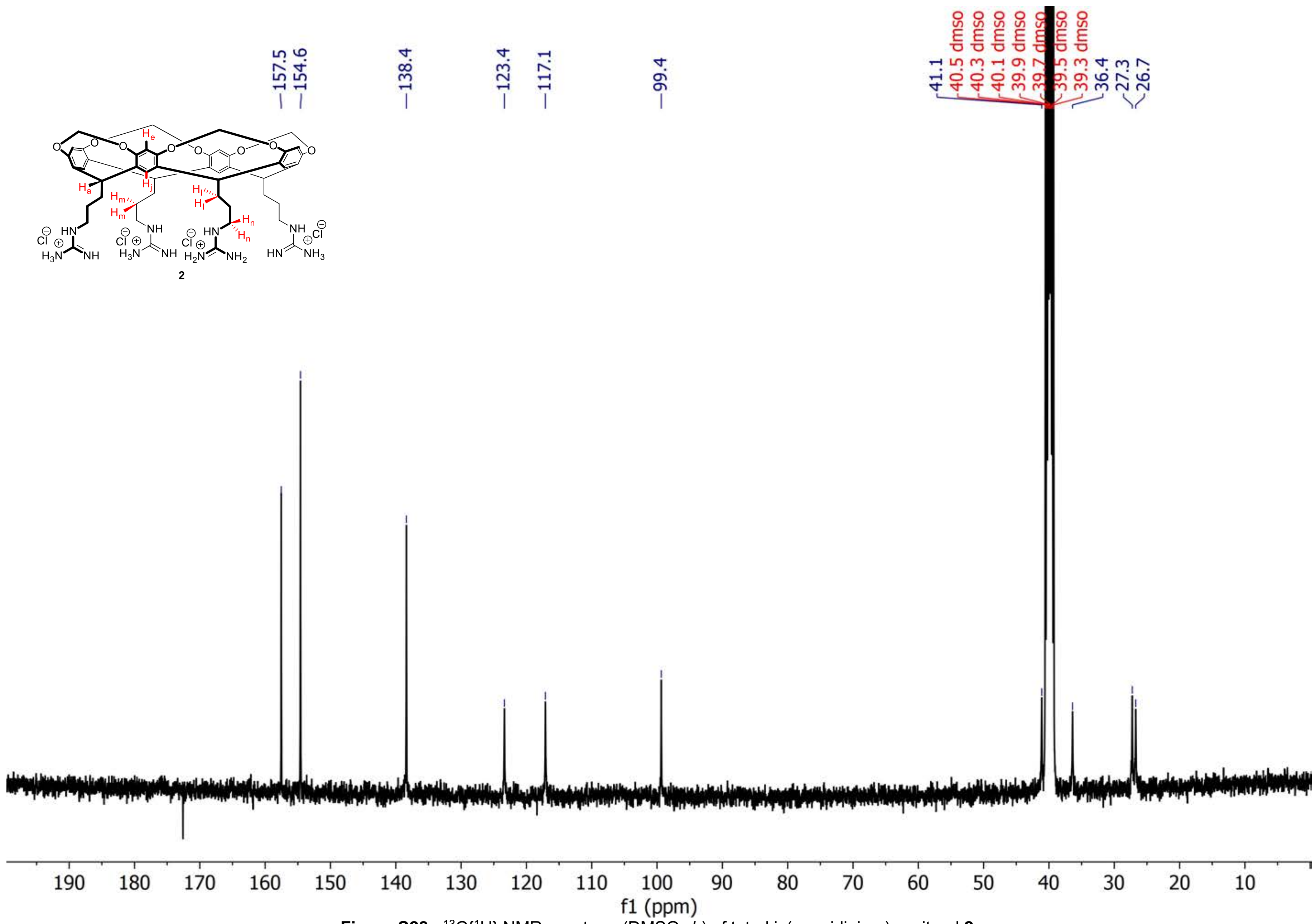

Figure S28. $^{13}C\{^{1}H\}$ NMR spectrum (DMSO-d_6) of tetrakis(guanidinium) cavitand **2**.

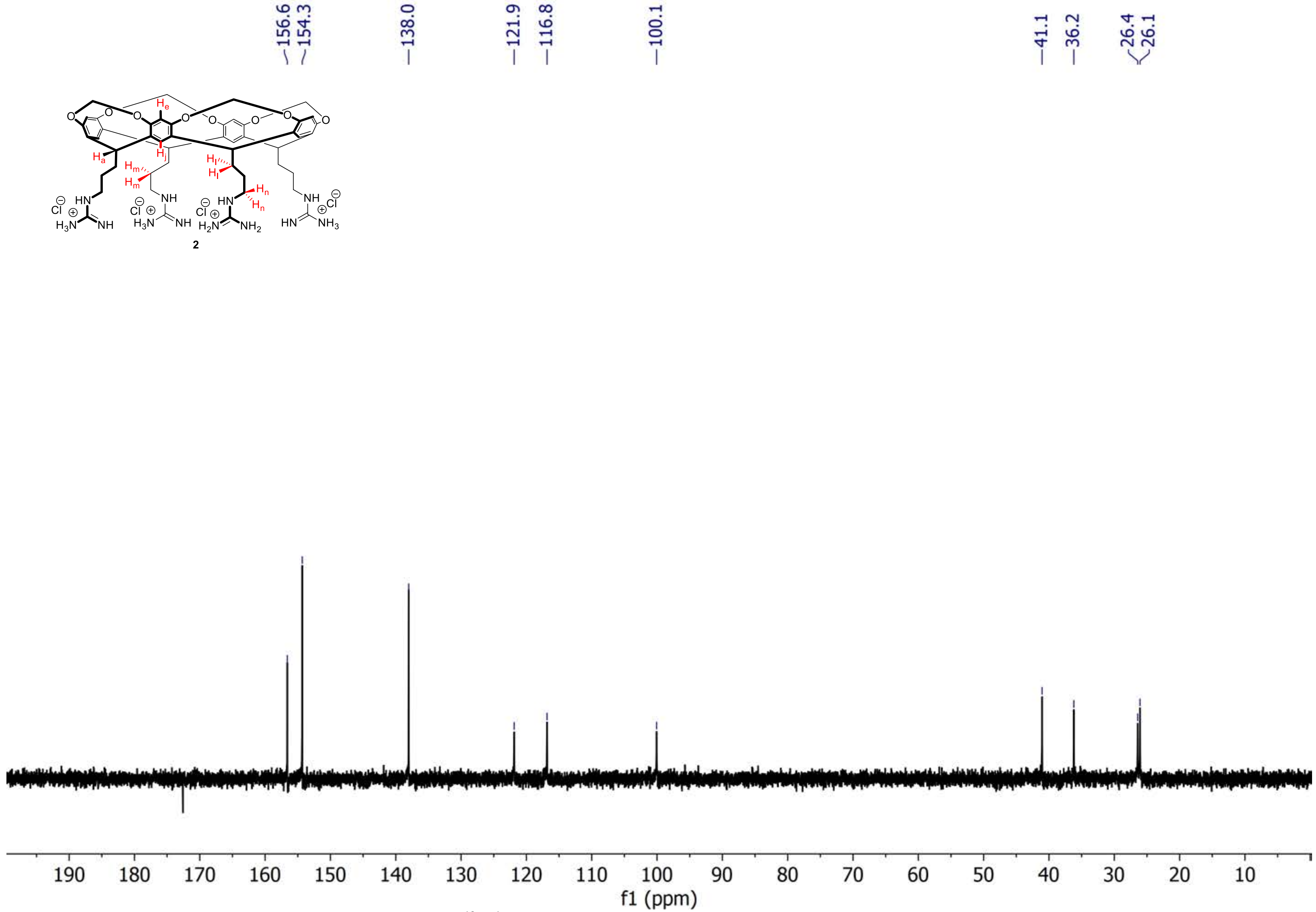

Figure S29. $^{13}C\{^{1}H\}$ NMR spectrum (D_2O) of tetrakis(guanidinium) cavitand **2**.

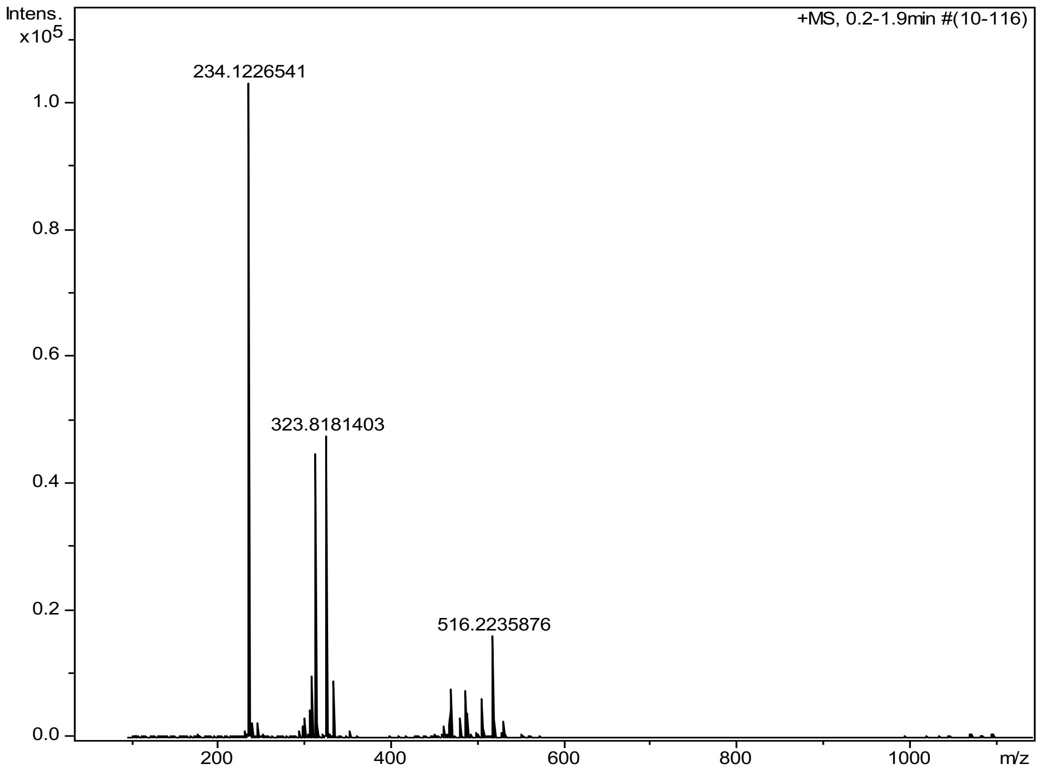

Figure S30. ESI- MS of tetrakis(guanidinium) chloride cavitand **2** (20 µM, distilled H_2O).

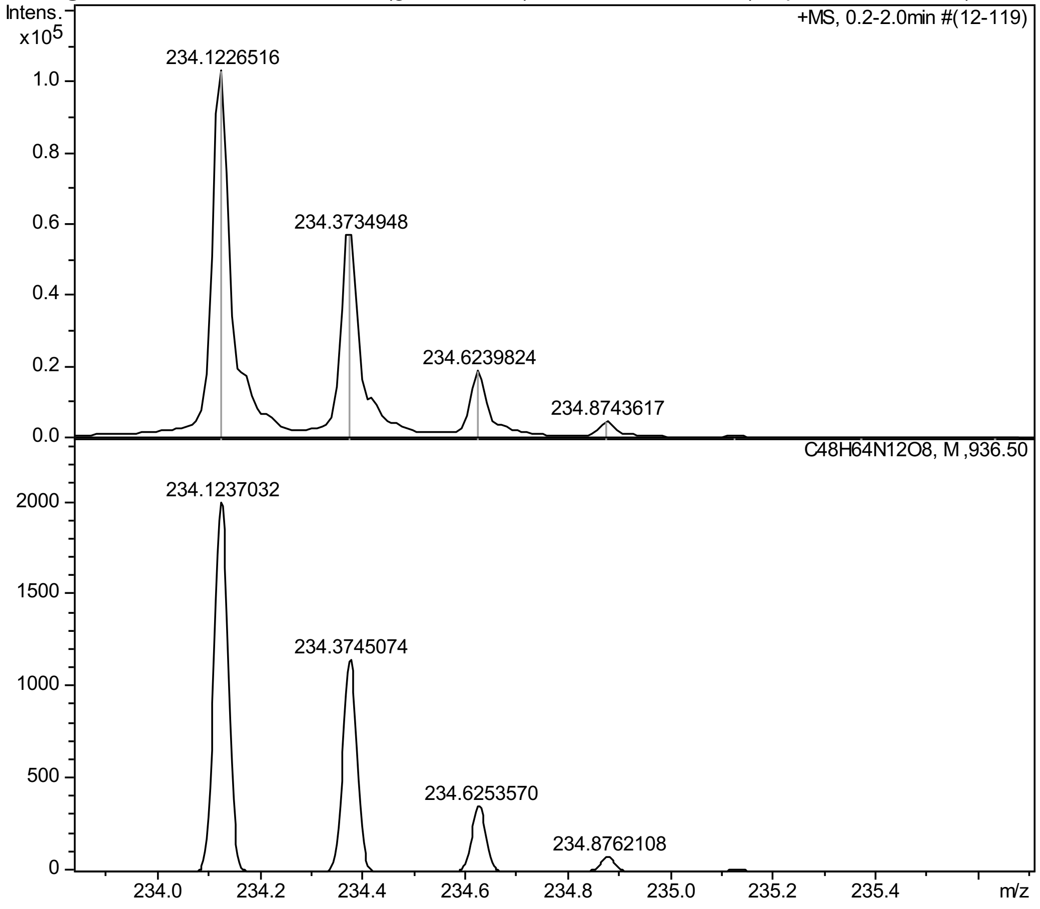

Figure S31. Expanded view of tetrakis(guanidinium) chloride cavitand **2**, $[M-4Cl]^{4+}$, with theoretical calculation below.

4. Analytical Data

NMR Shift (Δδmax) Summary

Figure S32 shows the ^{1}H NMR Δδ values for the H_j, H_l and H_n reporter protons for both hosts **1** and **2**.

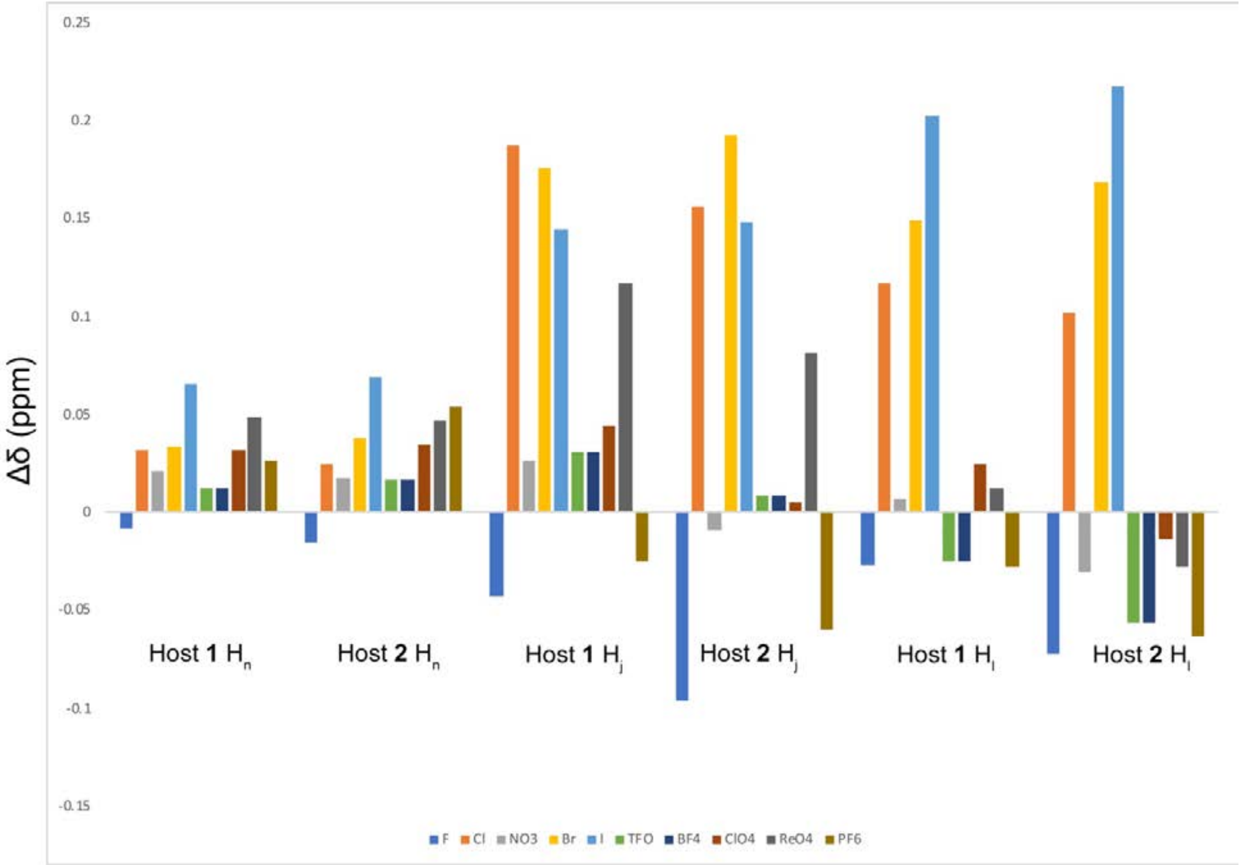

Figure S32. Bar graphs showing the anion-induced ^{1}H NMR signal shifts for reporter atoms H_j, H_l and H_n upon formation of the host **1** and host **2** complexes. Errors in signal shifts are ± 0.05 ppm.

NMR Data

Fitting of titration data using BINDFIT or the solver in Excel was performed maintaining the following assumptions, namely, in the absence of precipitation or aggregation:

$$G_t = [G] + [HG]_{crown} + n[HG_n]_{other} \quad \text{Eq. S2}$$

$$G_t \approx [G] + [HG]_{crown} \text{ since } [G] + [HG]_{crown} >> n[HG_n]_{other} \quad \text{Eq. S3}$$

where $[G_t]$ is the total guest concentration, $[HG]_{crown}$ is the concentration of the complex with the anion binding to the cationic crown at the feet of the cavitand, and $[HG_n]_{other}$ is the concentration of the complexes arising from pseudo-specific complexations.

For the examined salts, titrations monitored a combination of H_j, H_l or H_n of the host resonances (Scheme S1, Section 2.A, above) and were fit globally for all examined resonances by plotting the chemical shift (Δδ) and solving for K_a.

$$\Delta\delta = \frac{K_a[G]}{1+K_a[G]} \quad \text{Eq. S4}$$

Where, Δδ is the chemical shift of the host-guest complex, $[G_0]$ and $[H_0]$ are the total guest and host concentration respectively, and [G] is the free guest concentration which can be solved for using equation S5.

$$[G] = \frac{1}{2}\left(G_0 - H_0 - \frac{1}{K_a}\right) - \sqrt{\left(G_0 - H_0 - \frac{1}{K_a}\right)^2 + 4\frac{G_0}{K_a}} \quad \text{Eq. S5}$$

An example titration of **1** with sodium iodide (NaI) is shown in Figure S33, where the downfield shifts in H_l, H_n, and H_j are readily apparent. For most salts examined, titrations monitoring H_j, H_l or H_n were performed substantially below the critical precipitation concentration (CPC) of the salts to maximize mono-dispersity and ensure homogeneity of the hosts.

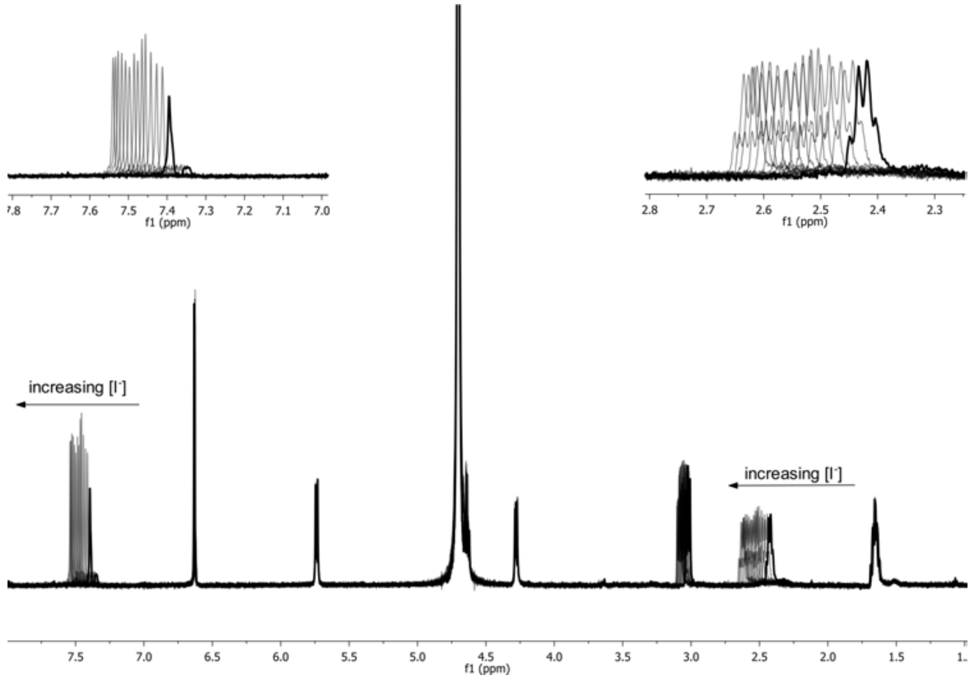

Figure S33. Superimposed image of ^{1}H NMR titration of **1** (0.4 mM) with NaI (15 mM). Expansions show the upfield shift of H_j and H_l upon addition of NaI.

For ^{1}H NMR titrations, the concentration of the host was chosen such that there were minimal (Δδ < 0.01) shifts in the host resonances upon 10% dilution, and also to minimize factors arising from anion-

induced aggregation. A representative titration and binding isotherm is shown for host **1** (Figure S34–Figure S51) and host **2** (Figure S52–Figure S69) for each of the eleven anions investigated in detail. The data obtained for free energy, binding constant and estimates of error in fit are summarized in Table S1.

	ΔG (kJ mol^{-1})			K_a (M^{-1})	CV (%)	*ΔG* (kJ mol^{-1})			K_a (M^{-1})	CV (%)
Anion	Host **1**					Host **2**				
Cl^-	–12.1	±	0.17	130	7	-13.3	±	0.03	210	1
NO_3^-	–13.1	±	0.29	200	12	-13.7	±	0.14	250	6
Br^-	–16.8	±	0.21	880	9	-17.5	±	0.12	1200	5
I^-	–19.3	±	0.08	2400	4	-19.9	±	0.25	3100	10
OTf^-	–15.6	±	0.25	540	14	-13.6	±	0.10	240	4
BF_4^-	–16.4	±	0.25	750	10	-15.9	±	0.31	630	13
ClO_4^-	–19.8	±	0.04	2900	2	-18.3	±	0.32	1700	13
ReO_4^-	–20.0	±	0.59	3200	7	-19.8	±	0.24	3000	10
PF_6^-	–20.2	±	0.25	3500	10	-18.0	±	0.22	1500	9

Table S1: Summary of ^{1}H NMR titration data for binding constant determination. K_a, *ΔG*, and error for hosts **1** and **2** (0.4 mM, D_2O). The pD of the solutions was uncorrected.

a. ^{1}H NMR titrations of host **1** with salts

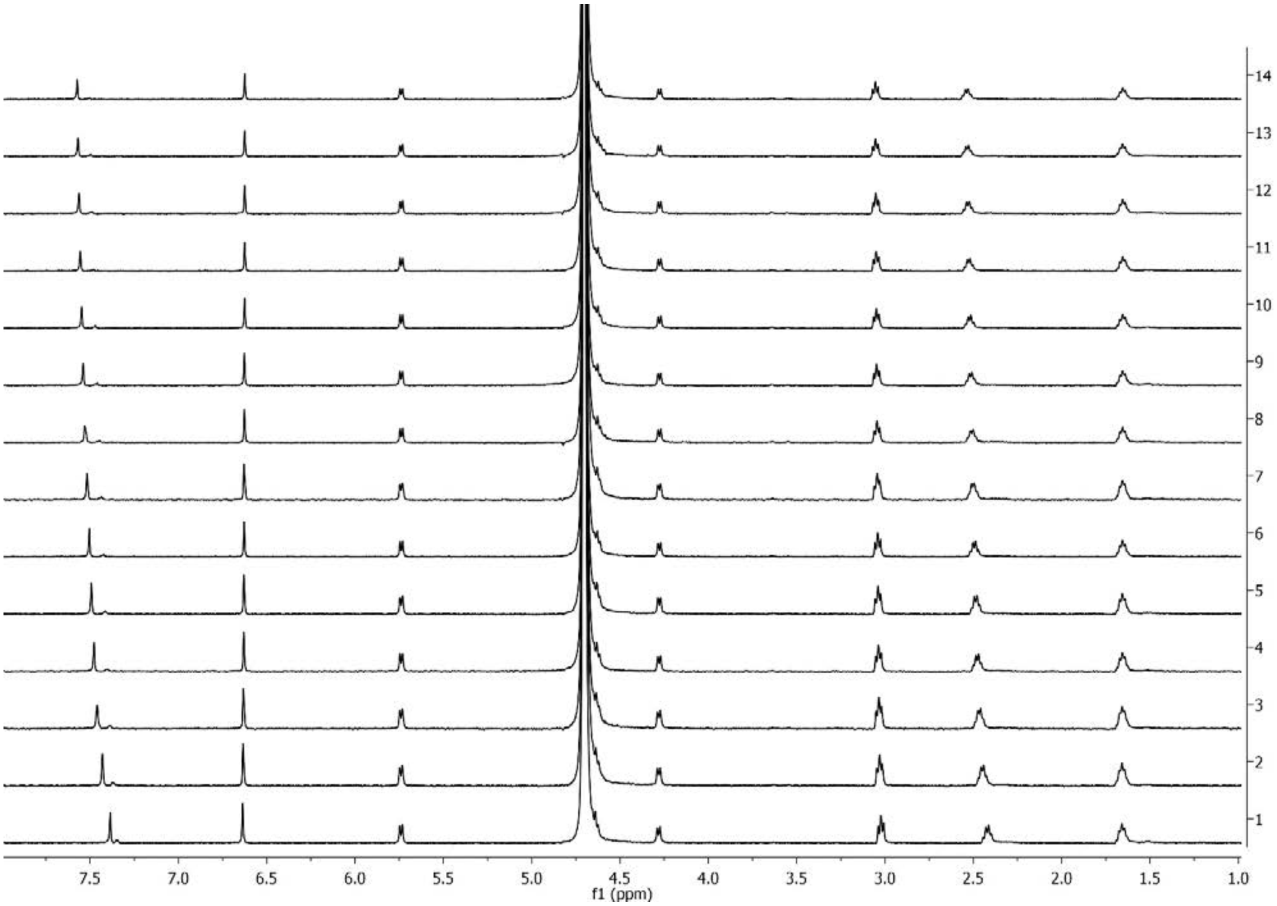

Figure S34. Representative ^{1}H NMR titration of **1** with 300 mM Cl$^-$ up to 103 equiv.

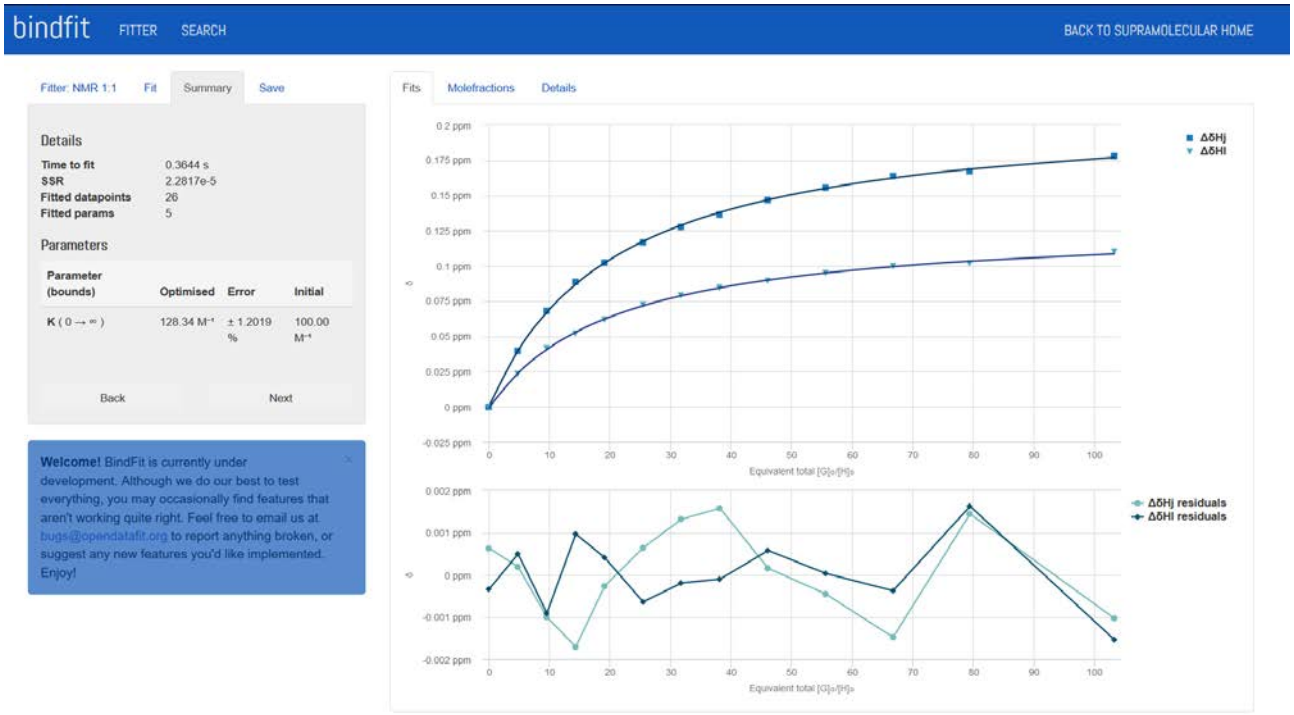

Figure S35. Fit of the data for H_j and H_l from Figure S34 to a 1:1 binding model.

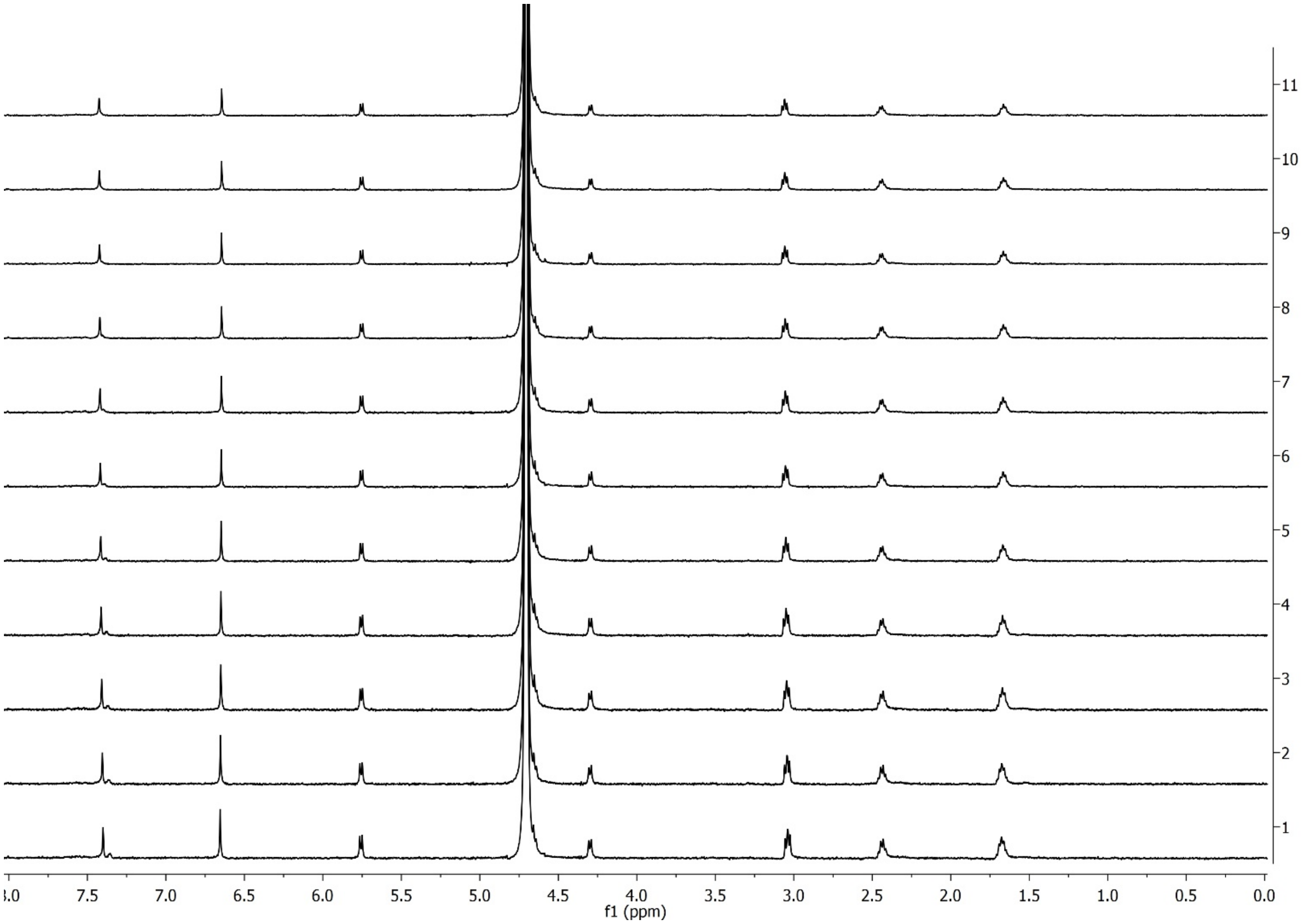

Figure S36. Representative ^{1}H NMR titration of **1** with 200 mM NO_3^- up to 59 equiv.

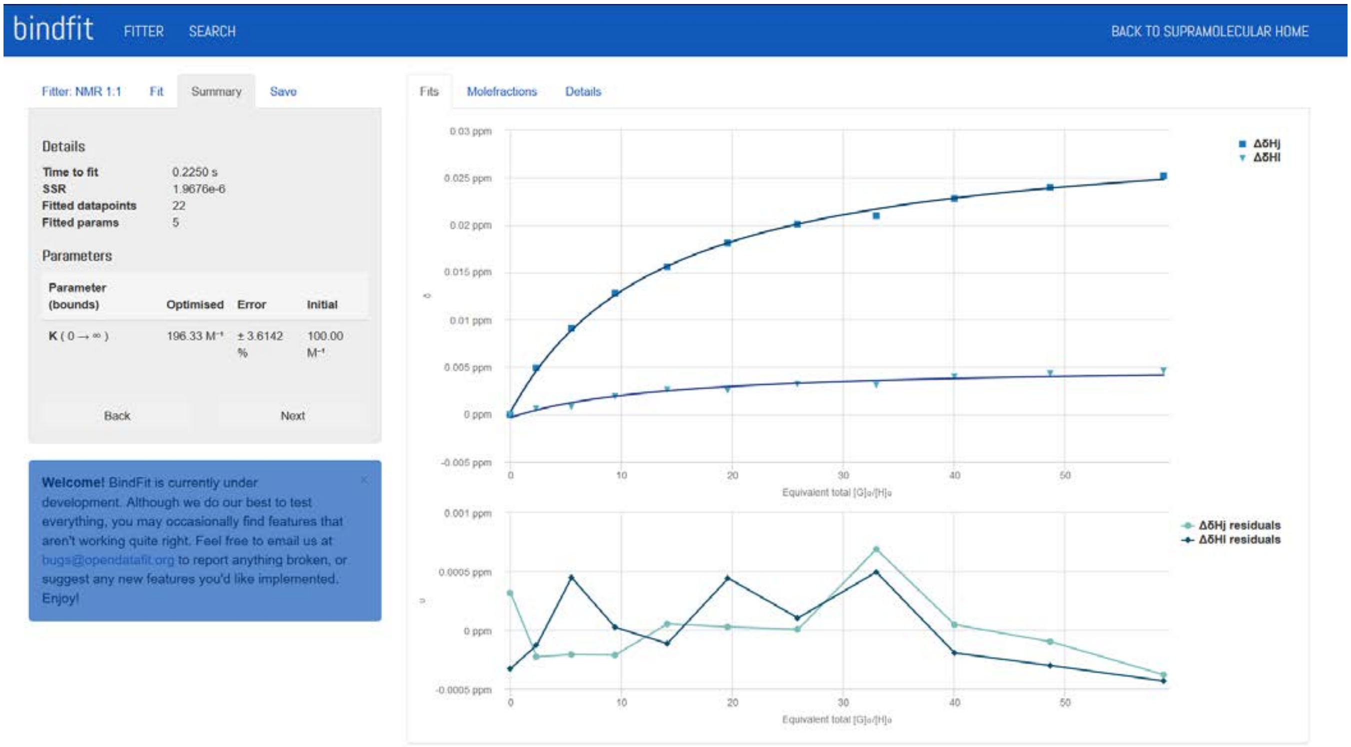

Figure S37. Fit of the data for H_j and H_l from Figure S36 to a 1:1 binding model.

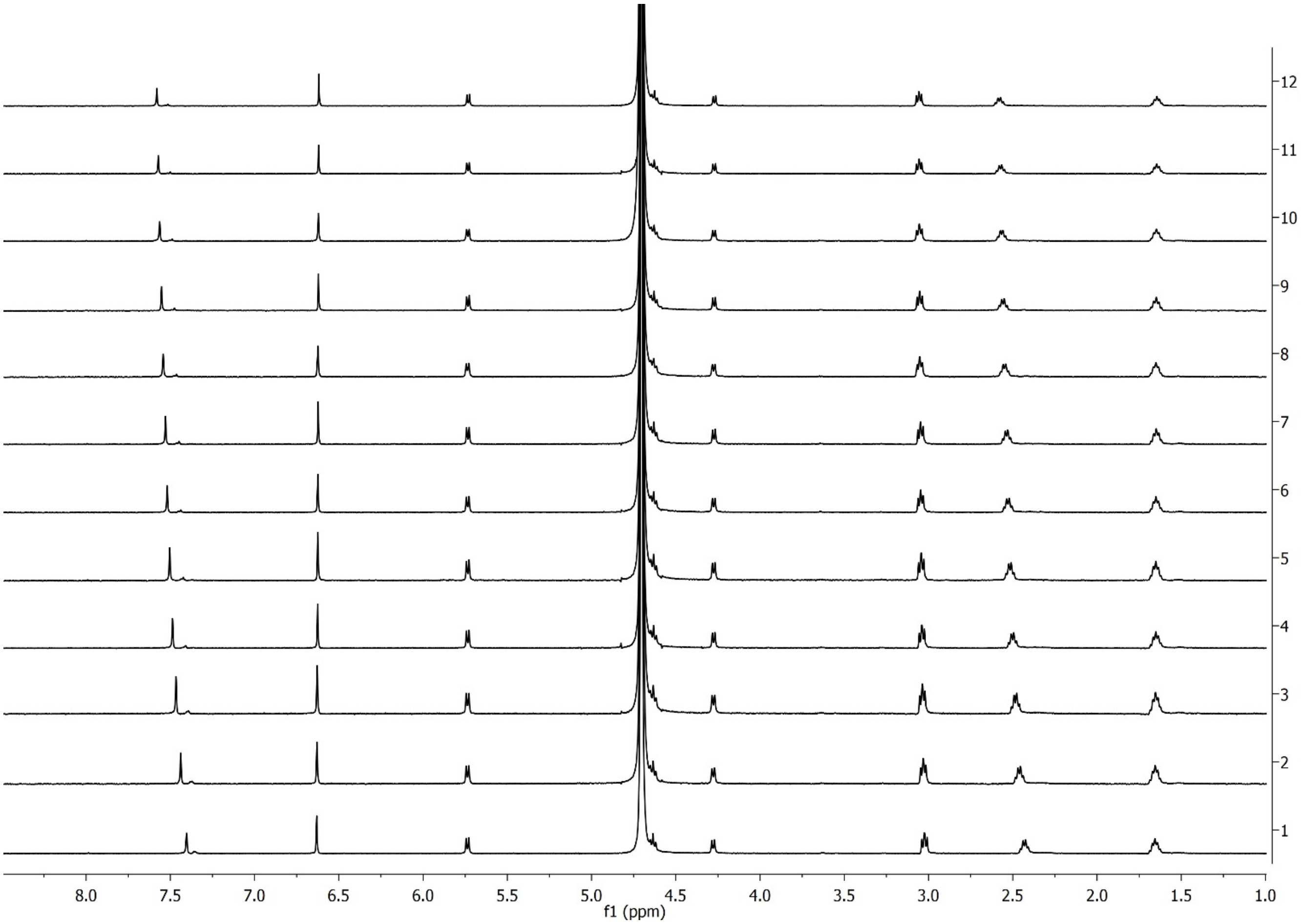

Figure S38. Representative ^{1}H NMR titration of **1** with 61 mM Br^{-} up to 9.6 equiv.

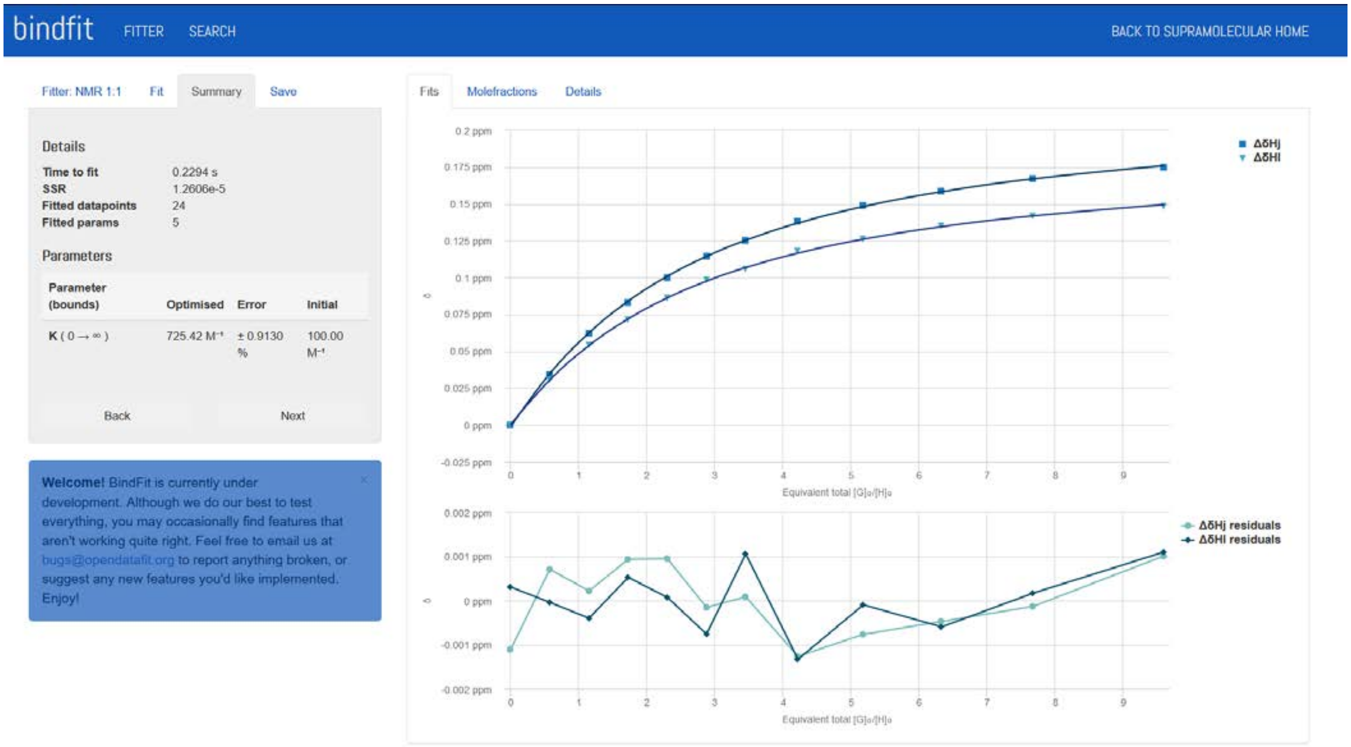

Figure S39. Fit of the data for H_j and H_l from Figure S38 to a 1:1 binding model.

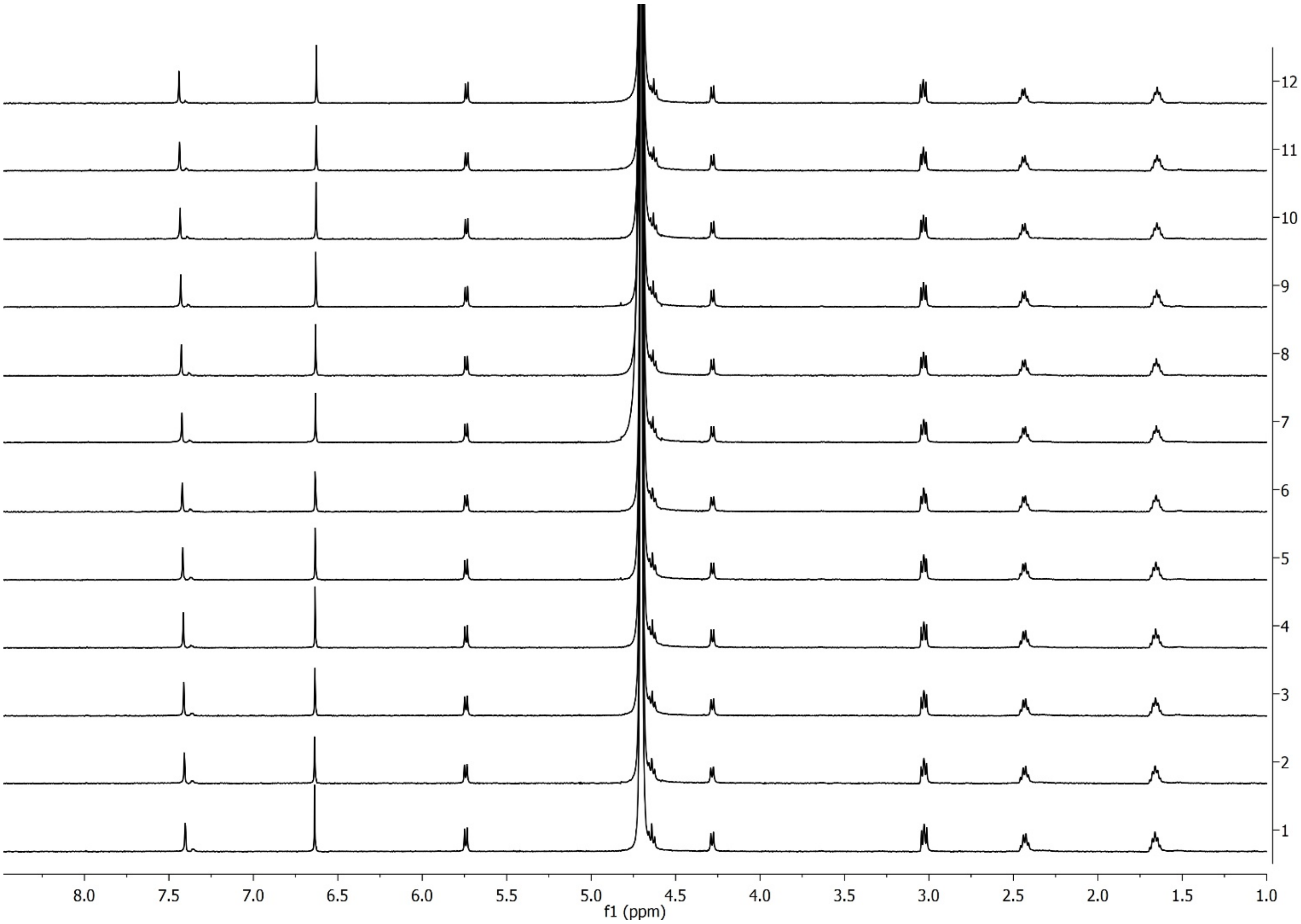

Figure S40. Representative ^{1}H NMR titration of **1** with 48 mM TfO^{-} up to 16.3 equiv.

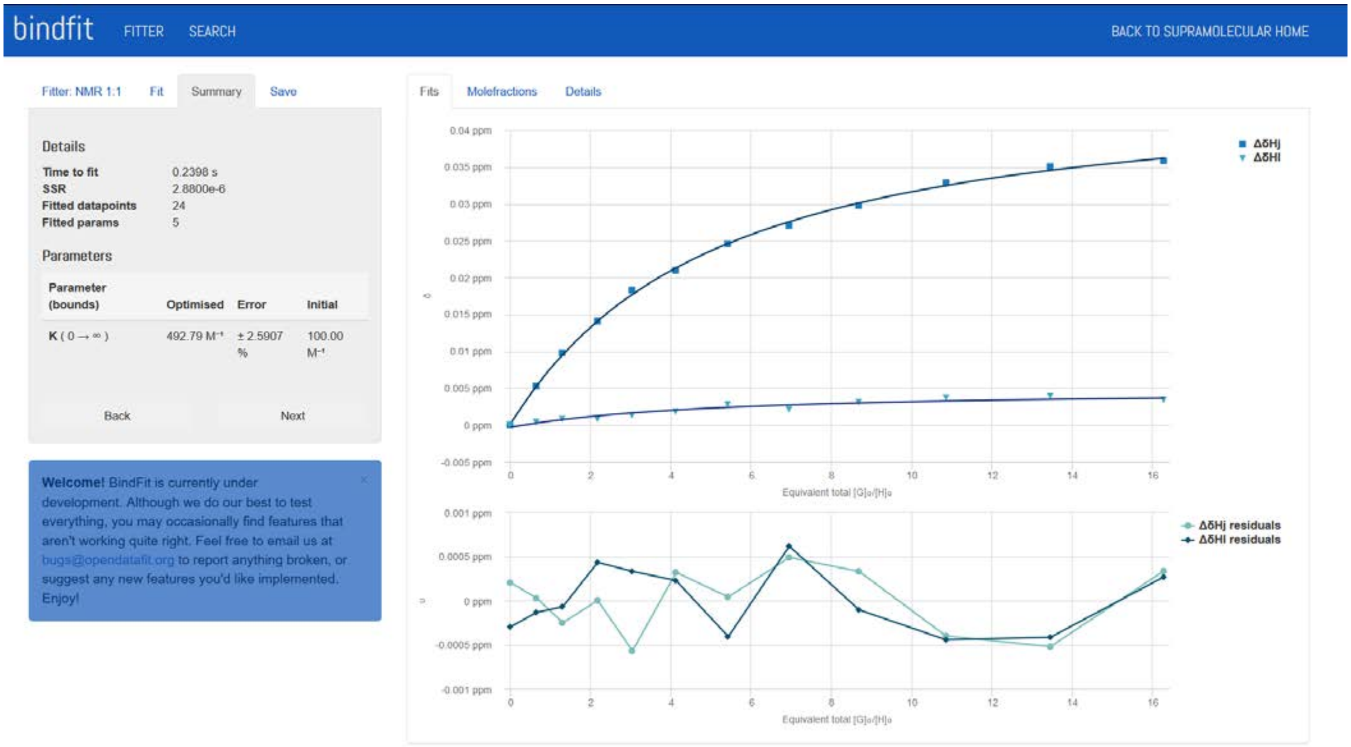

Figure S41. Fit of the data for H_j and H_l from Figure S40 to a 1:1 binding model.

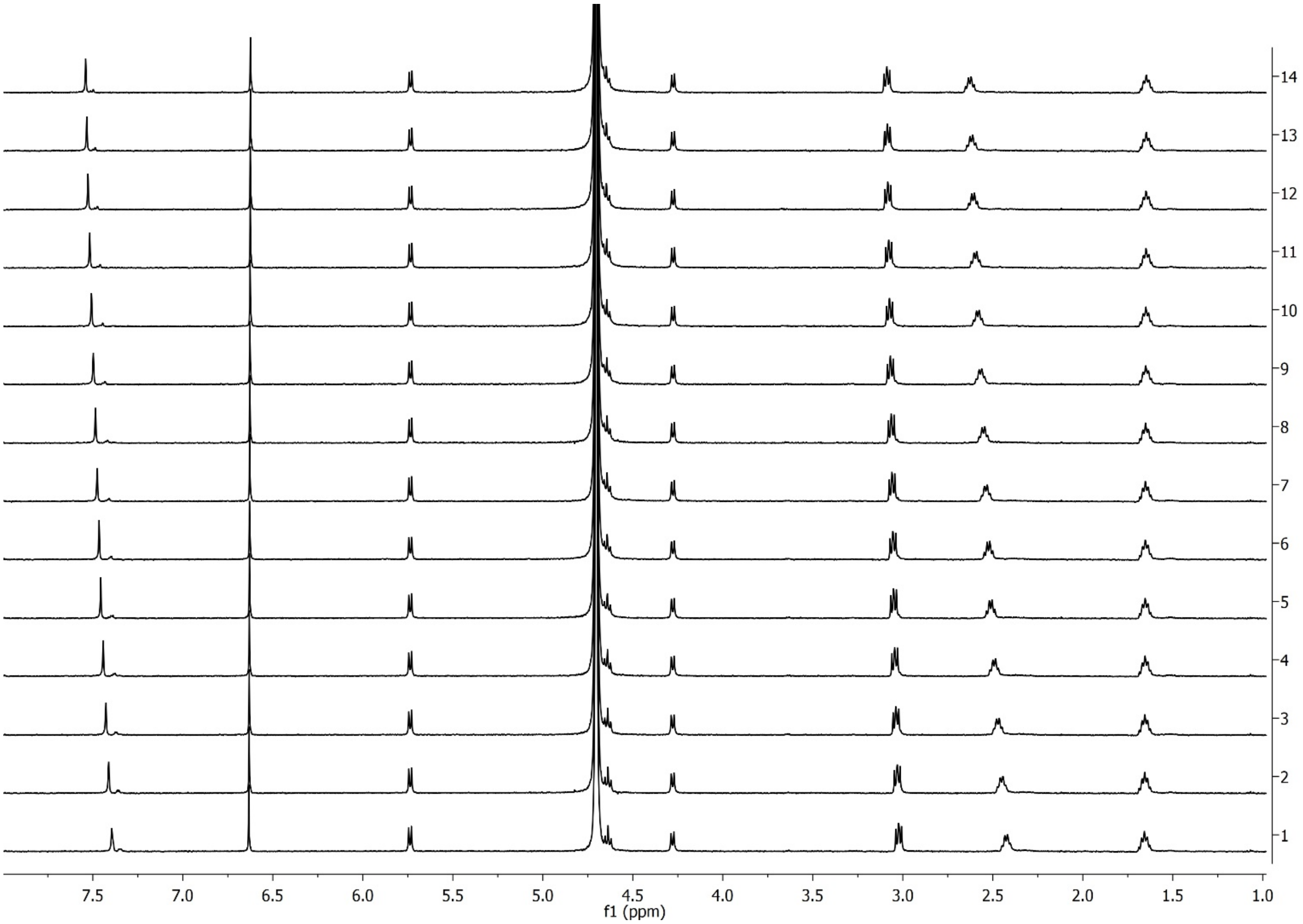

Figure S42. Representative ^{1}H NMR titration of **1** with 25 mM I^{-} up to 5.0 equiv.

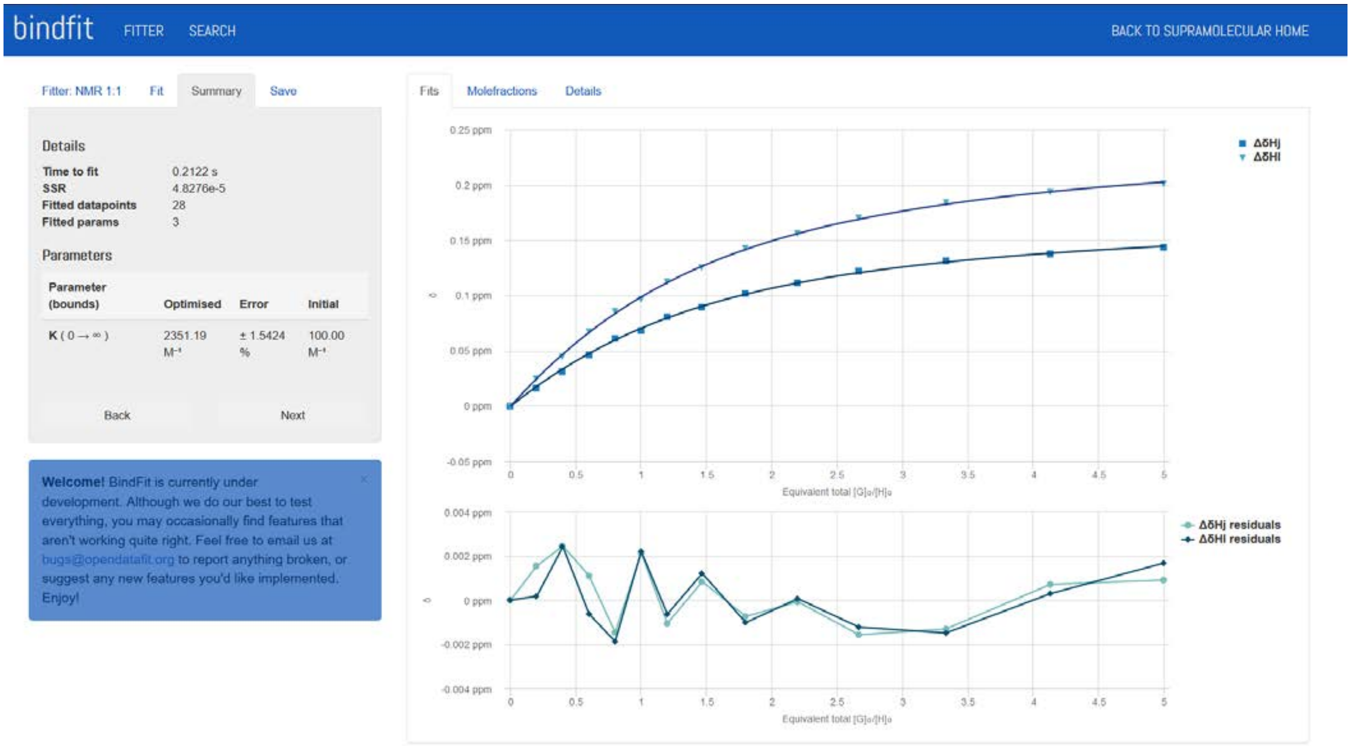

Figure S43. Fit of the data for H_j and H_l from Figure S42 to a 1:1 binding model.

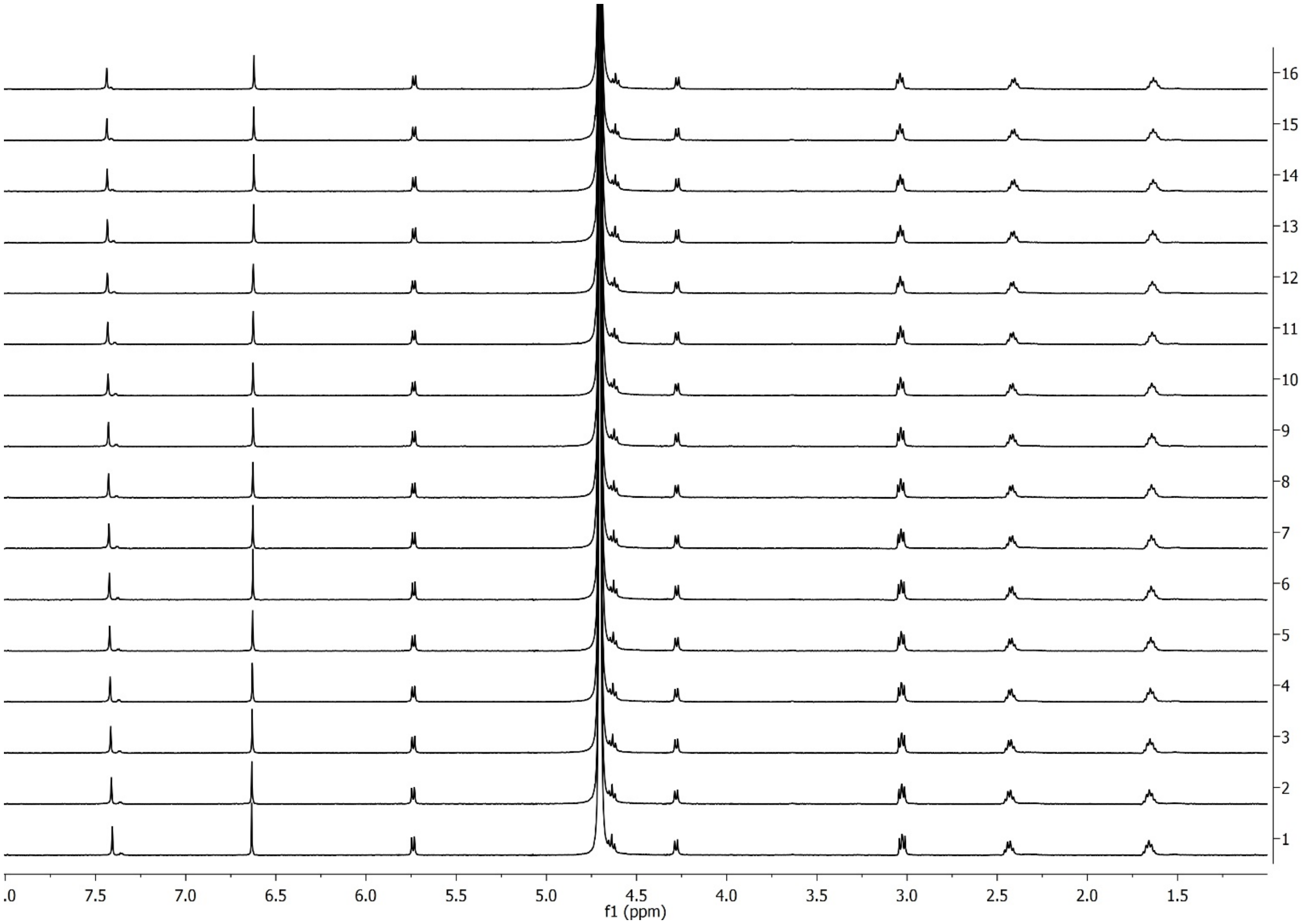

Figure S44. Representative ^{1}H NMR titration of **1** with 82 mM BF_4^- up to 16.4 equiv.

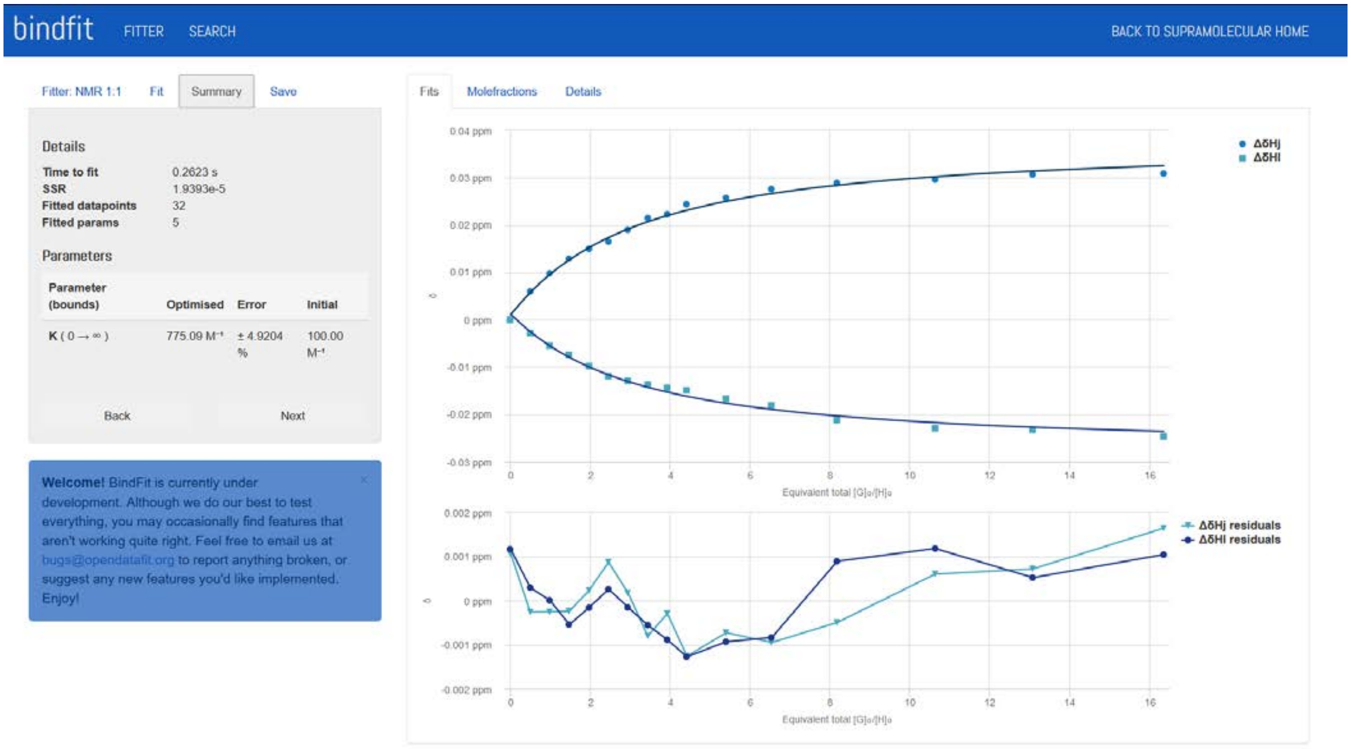

Figure S45. Fit of the data for H_j and H_l from Figure S44 to a 1:1 binding model.

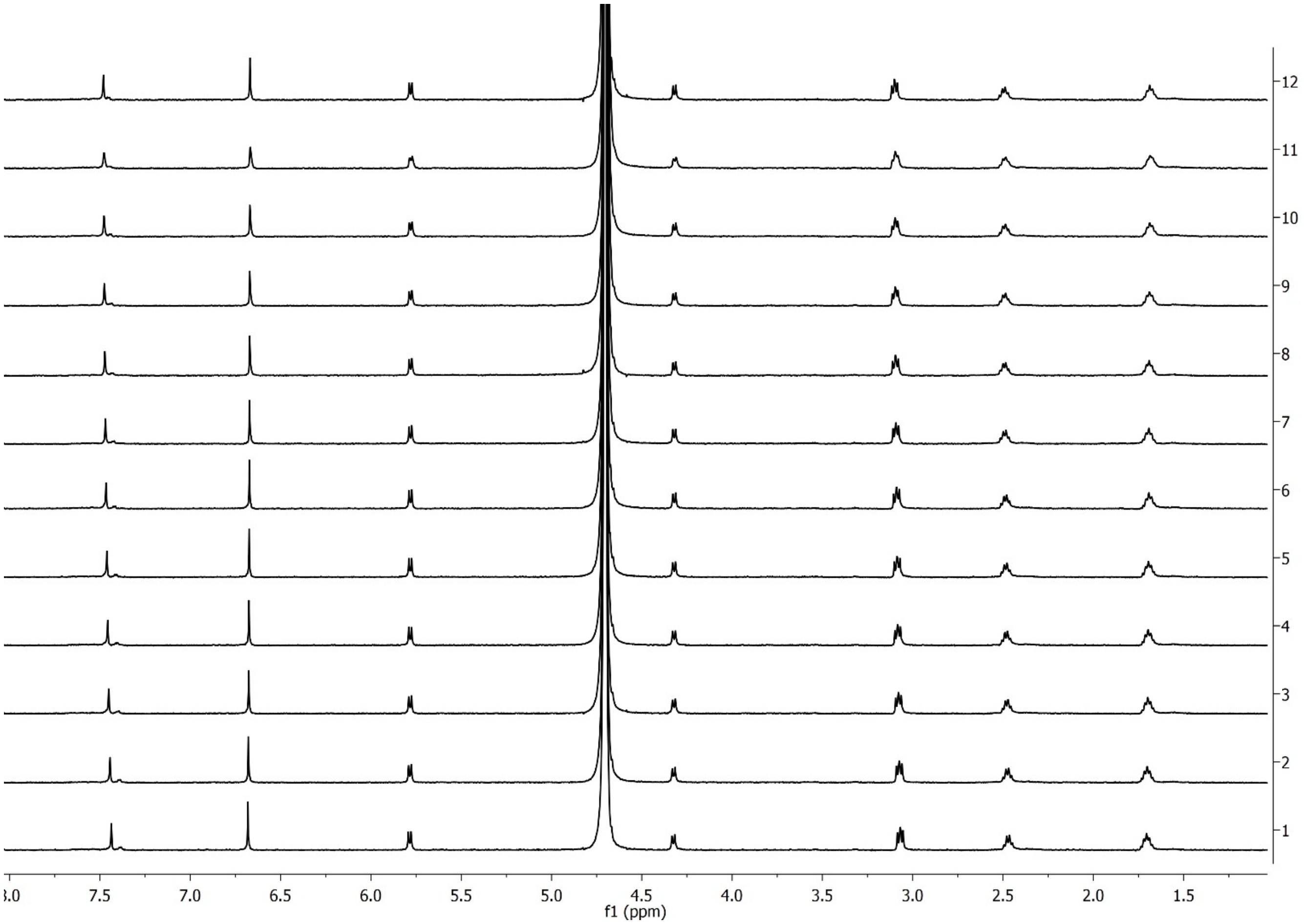

Figure S46. Representative ^1H NMR titration of **1** with 21 mM ClO_4^- up to 4.8 equiv.

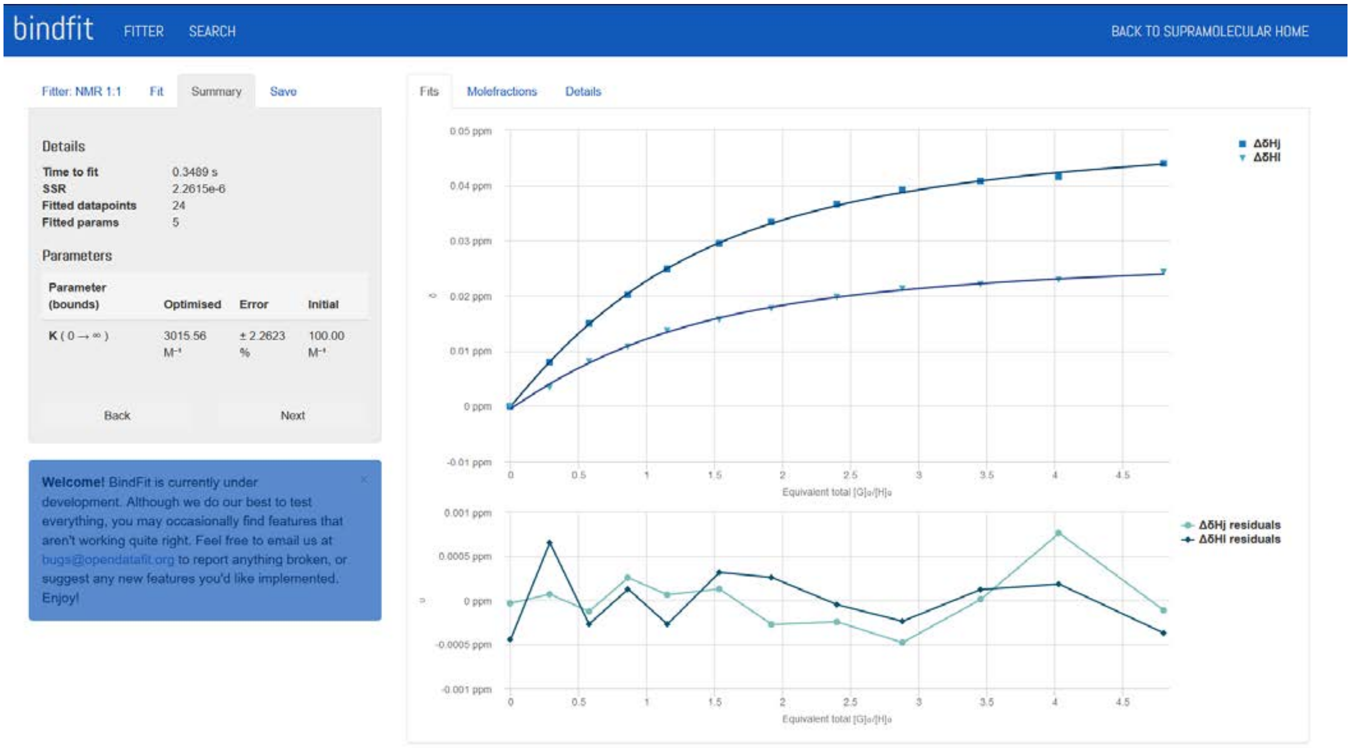

Figure S47. Fit of the data for H_j and H_l from Figure S46 to a 1:1 binding model.

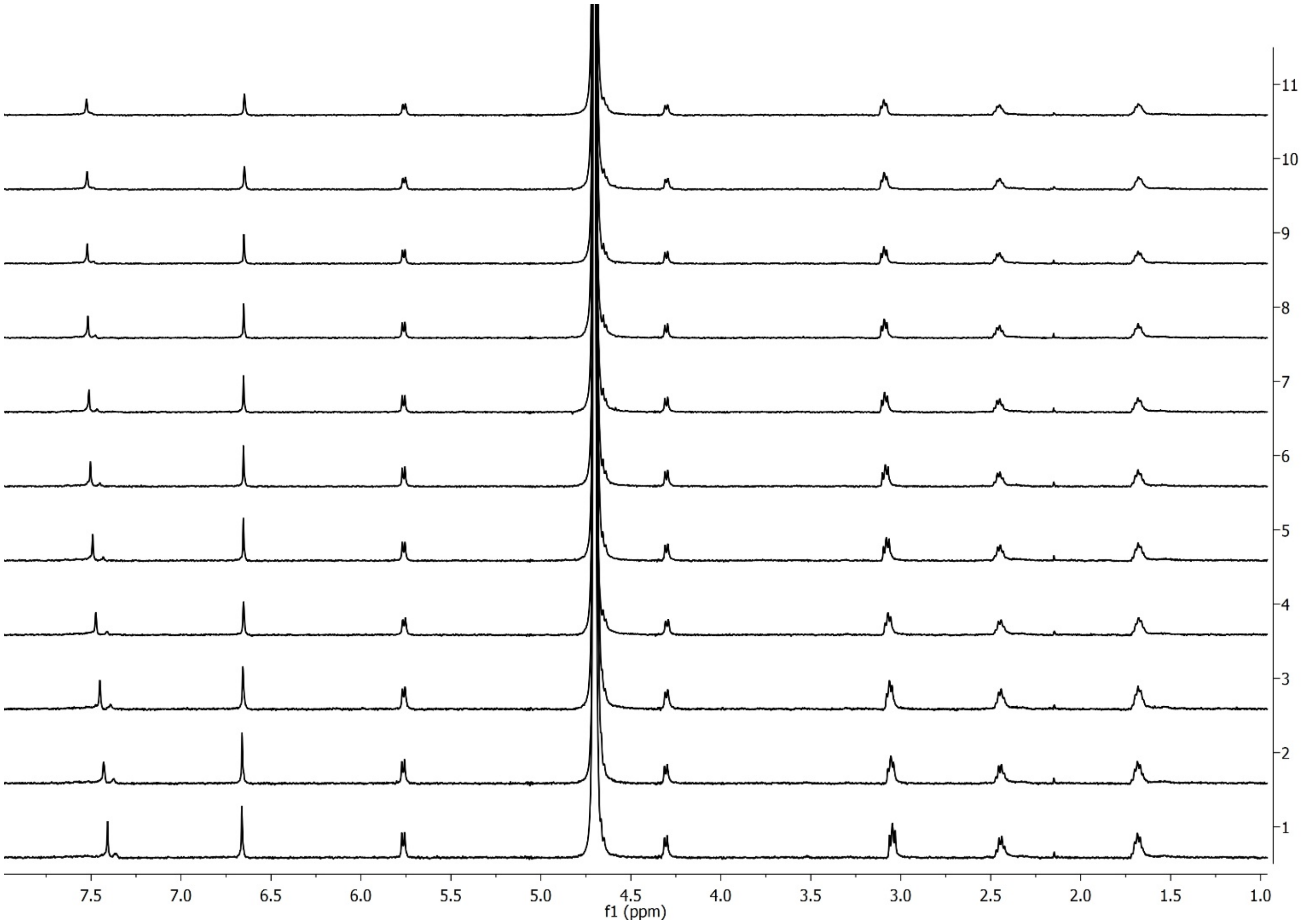

Figure S48. Representative ^{1}H NMR titration of **1** with 20 mM ReO_4^- up to 7.7 equiv.

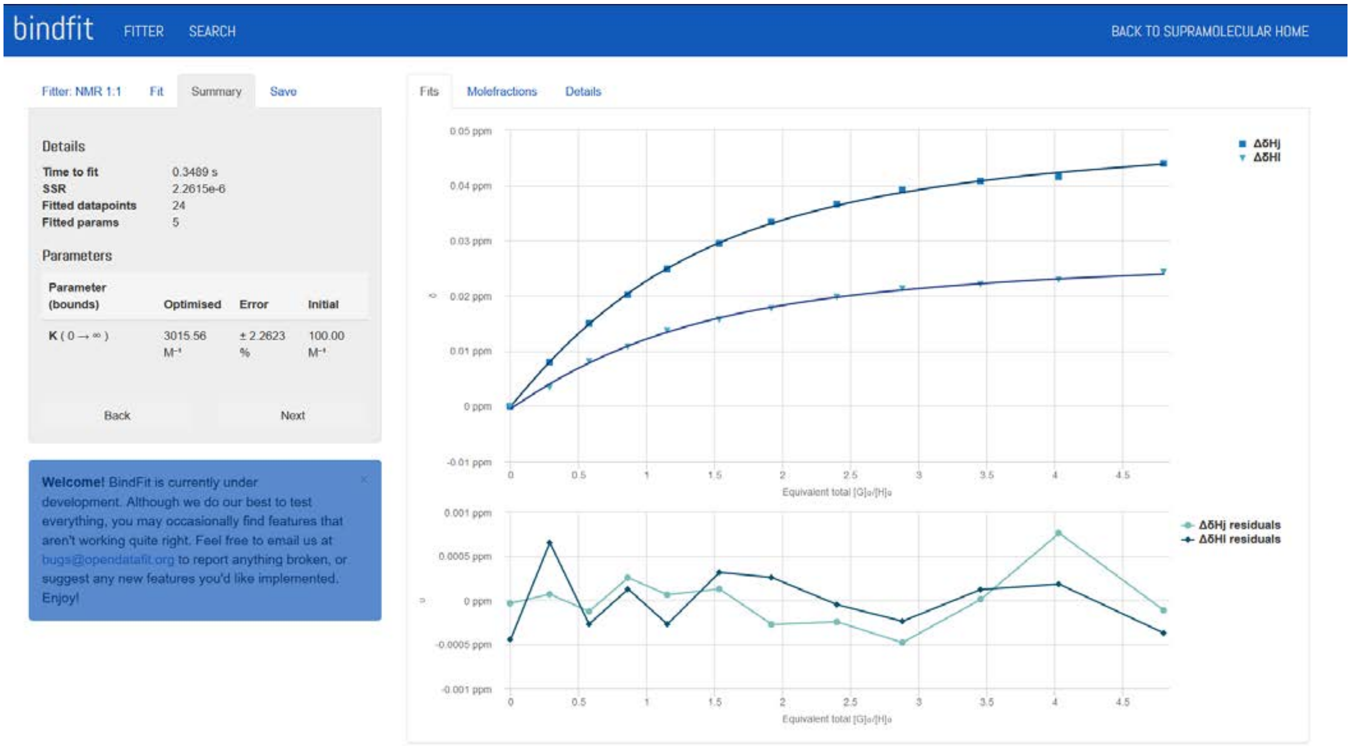

Figure S49. Fit of the data for H_j and H_l from Figure S48 to a 1:1 binding model.

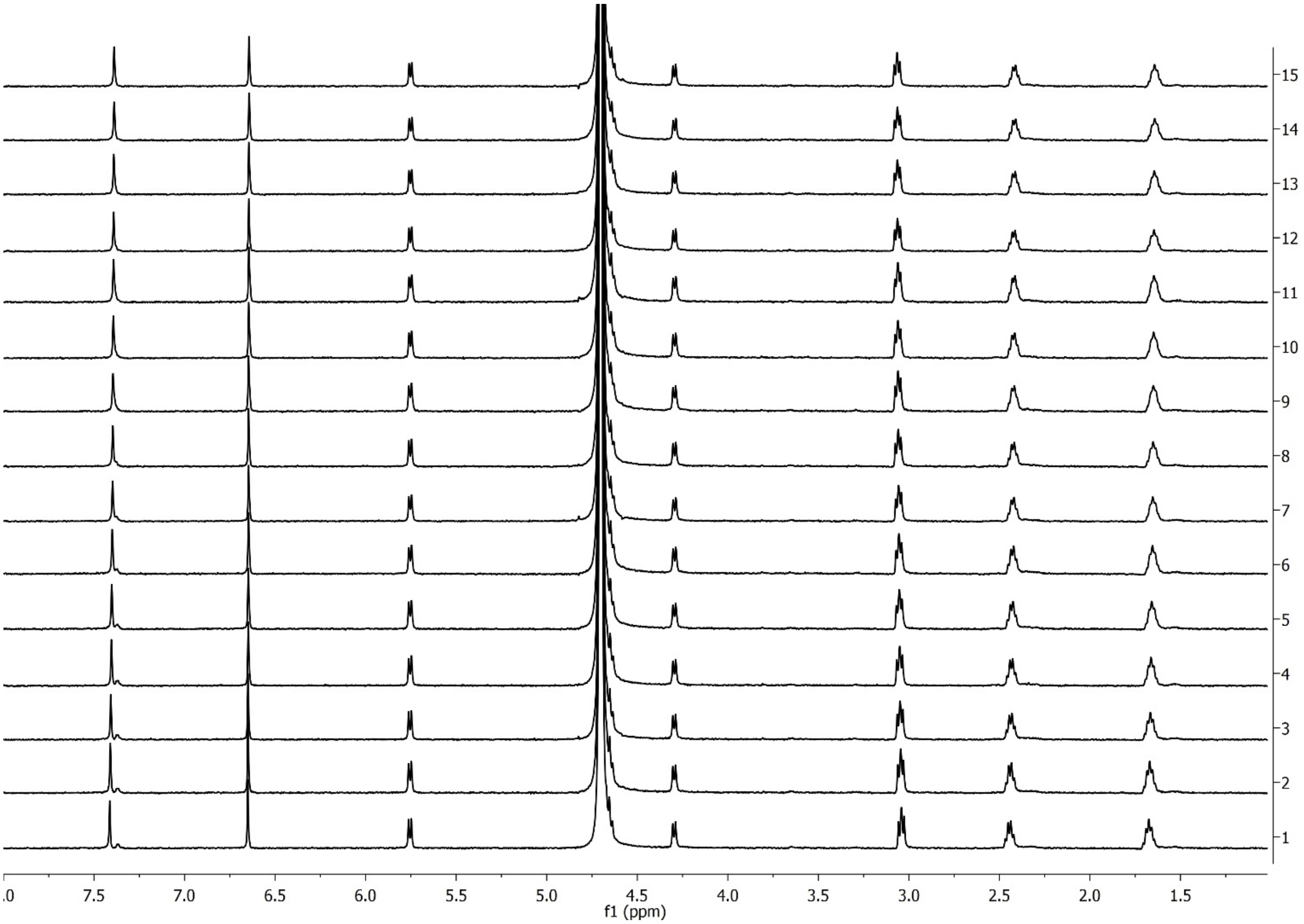

Figure S50. Representative ^{1}H NMR titration of **1** with 30 mM PF_6^- up to 4.9 equiv.

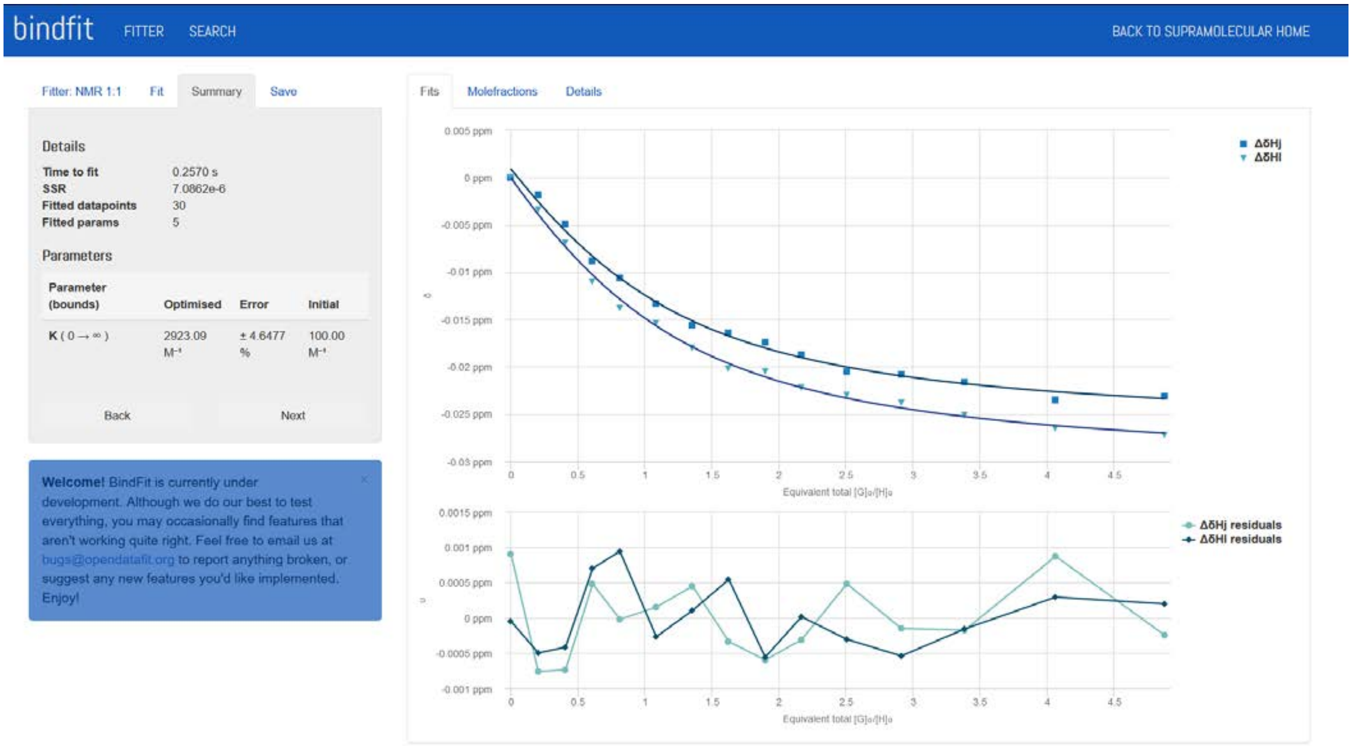

Figure S51. Fit of the data for H_j and H_l from Figure S50 to a 1:1 binding model.

b. ^{1}H NMR titration of host **2** with salts

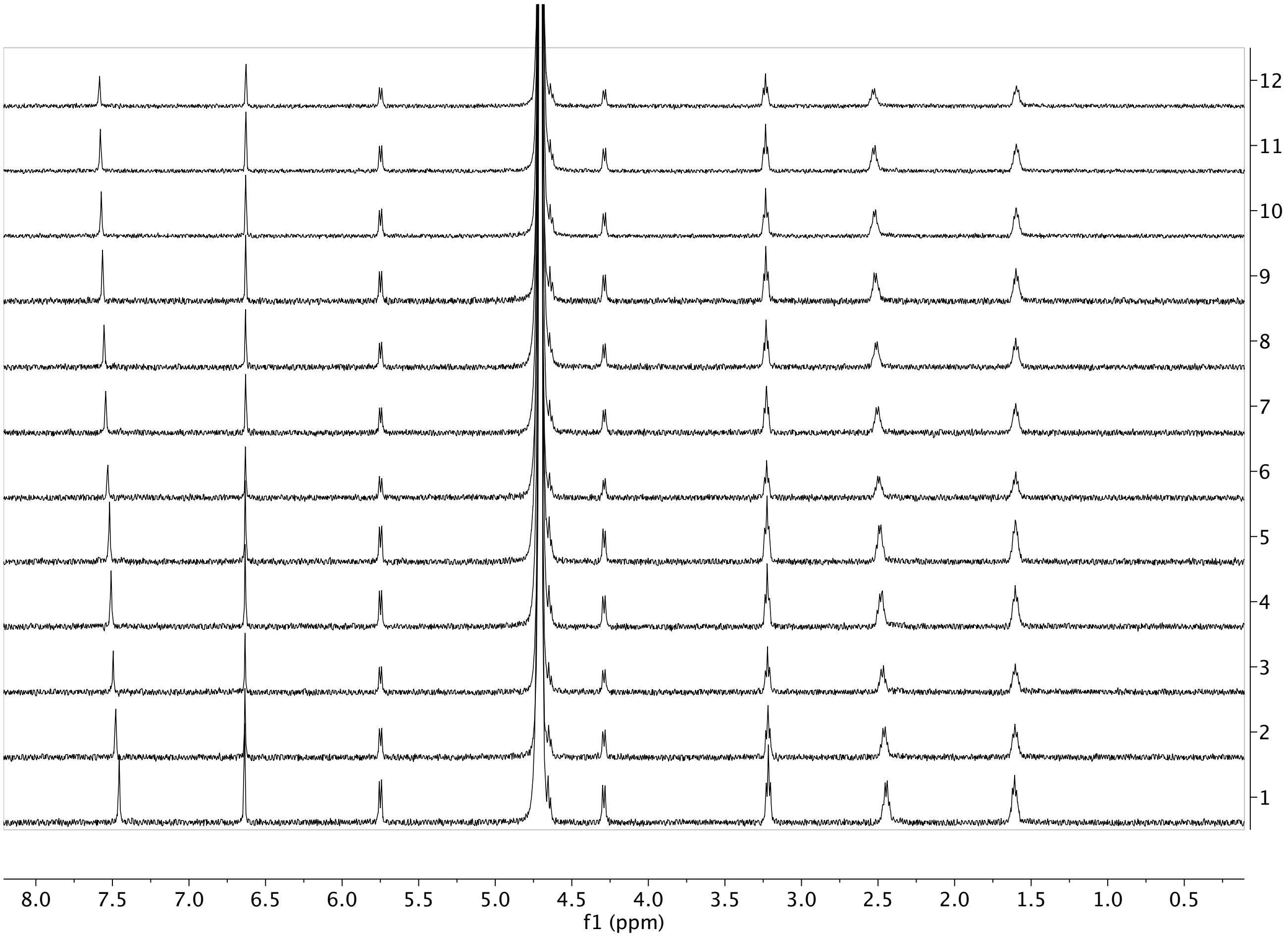

Figure S52. Representative ^{1}H NMR titration of **2** with 200 mM Cl^- up to 50 equiv.

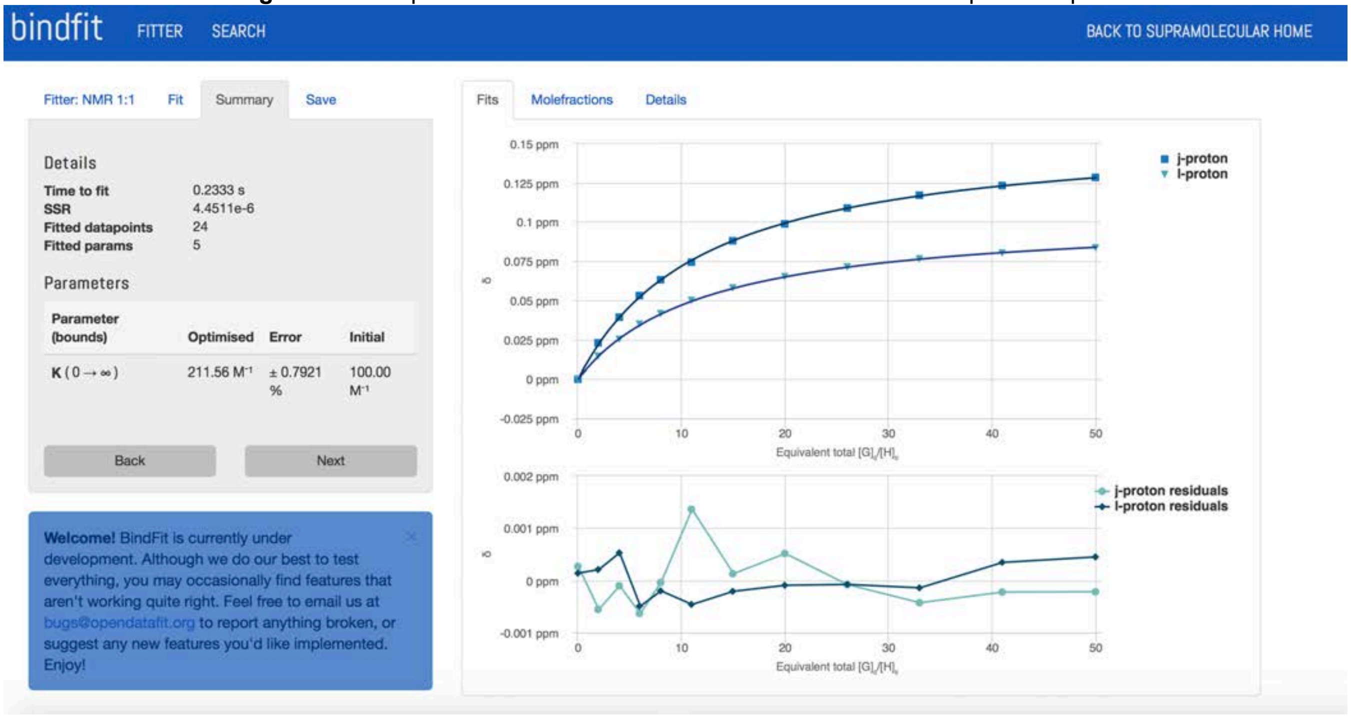

Figure S53. Fit of the data for H_j and H_l from Figure S52 to a 1:1 binding model.

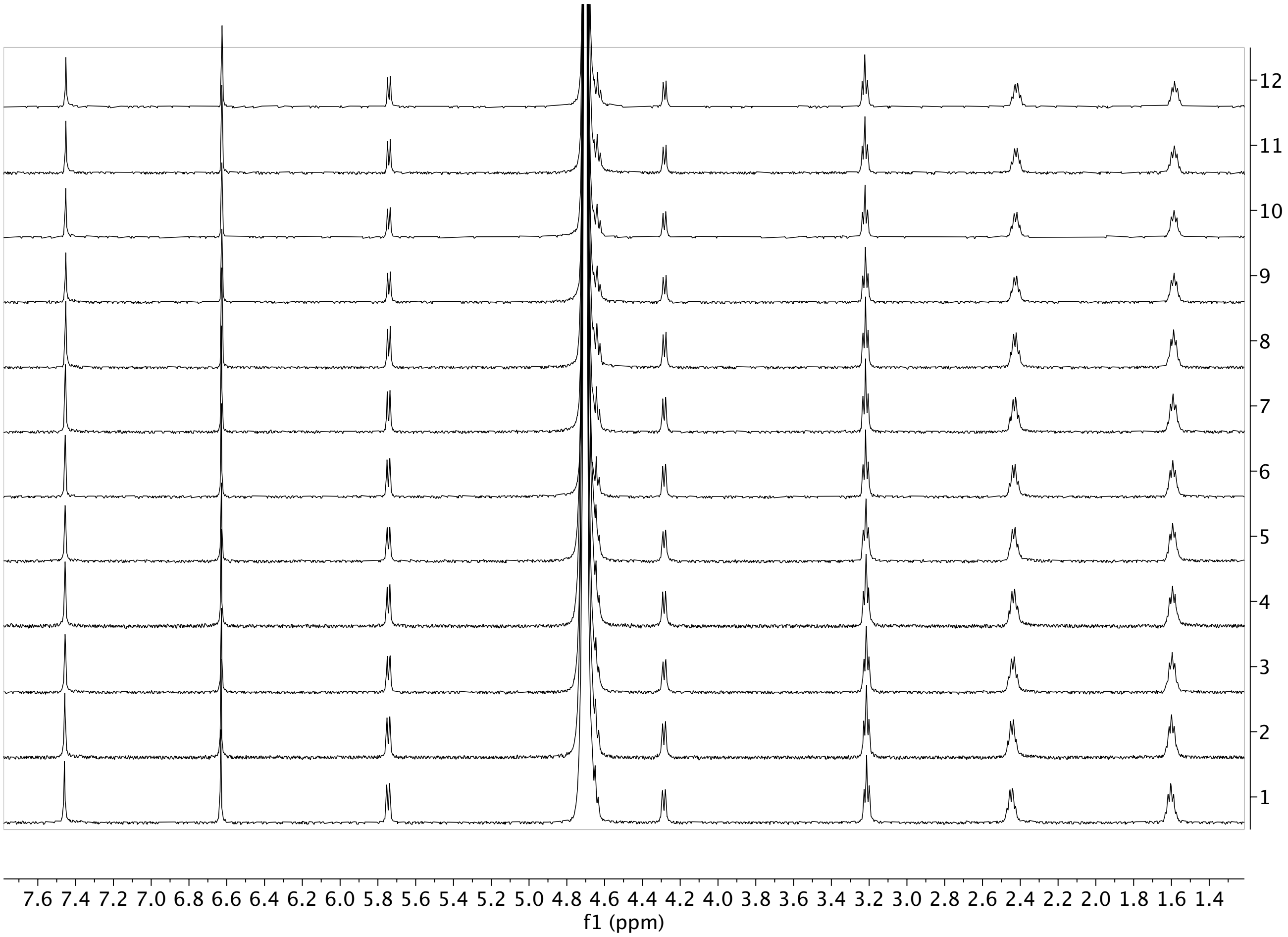

Figure S54. Representative ^{1}H NMR titration of **2** with 150 mM NO_3^- up to 34 equiv.

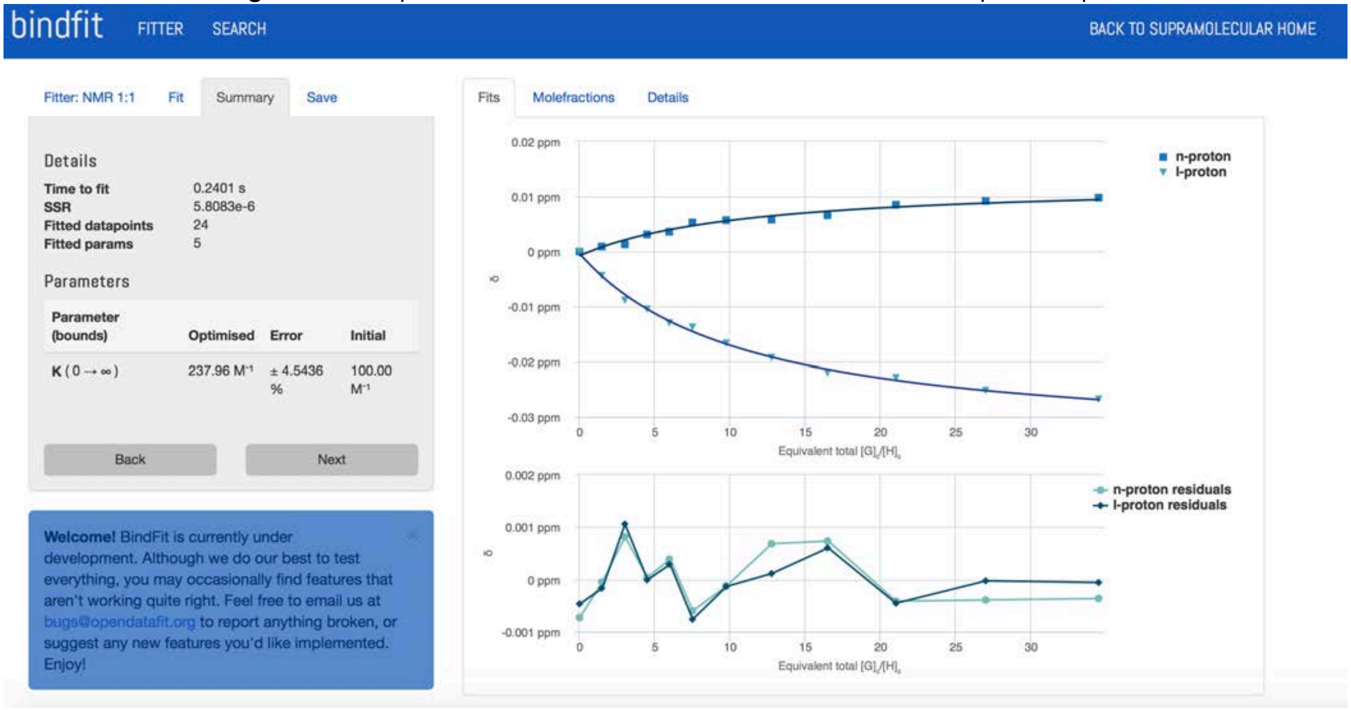

Figure S55. Fit of the data for H_n and H_l from Figure S54 to a 1:1 binding model.

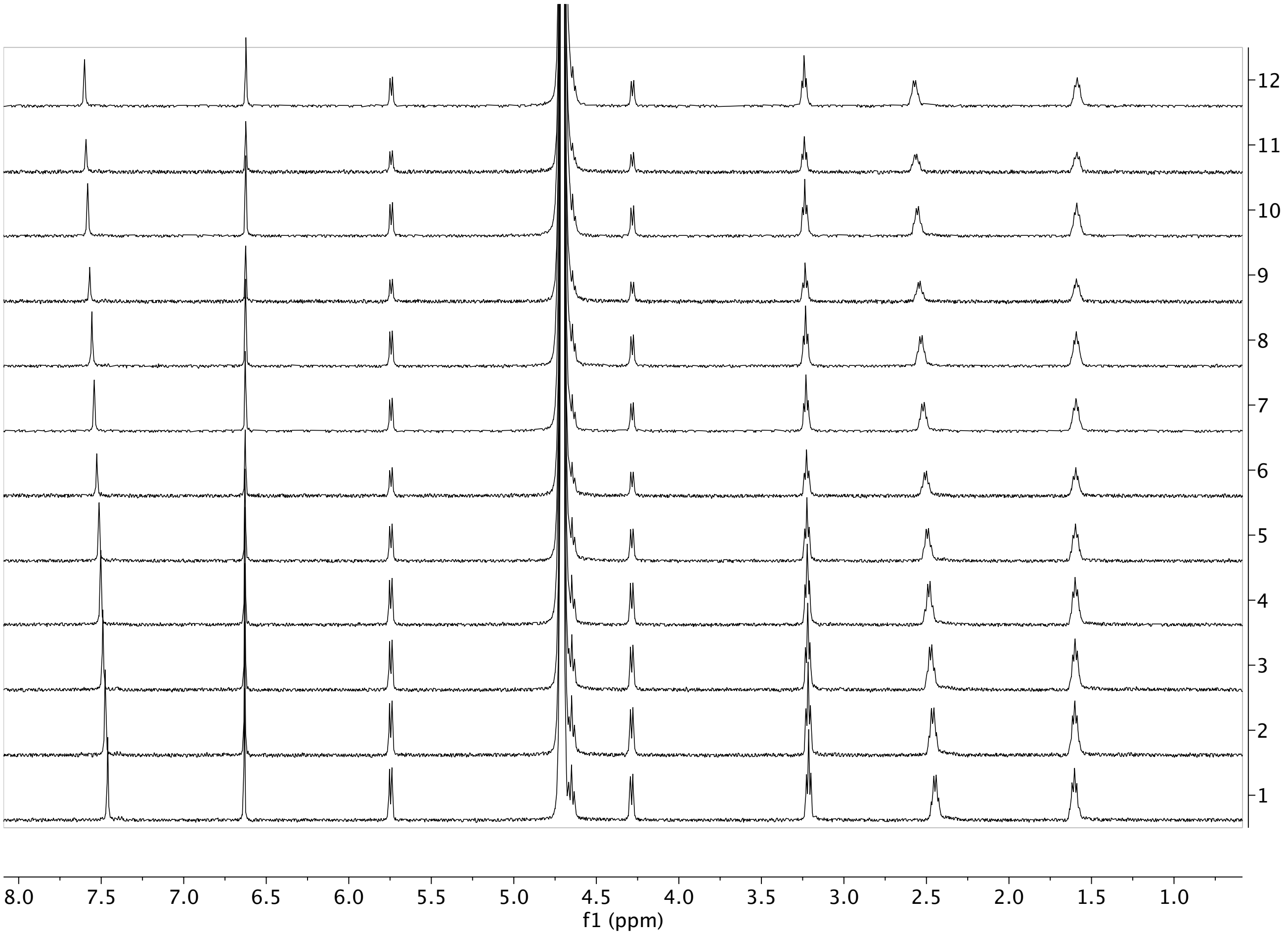

Figure S56. Representative ^{1}H NMR titration of **2** with 61 mM Br^- up to 8.5 equiv.

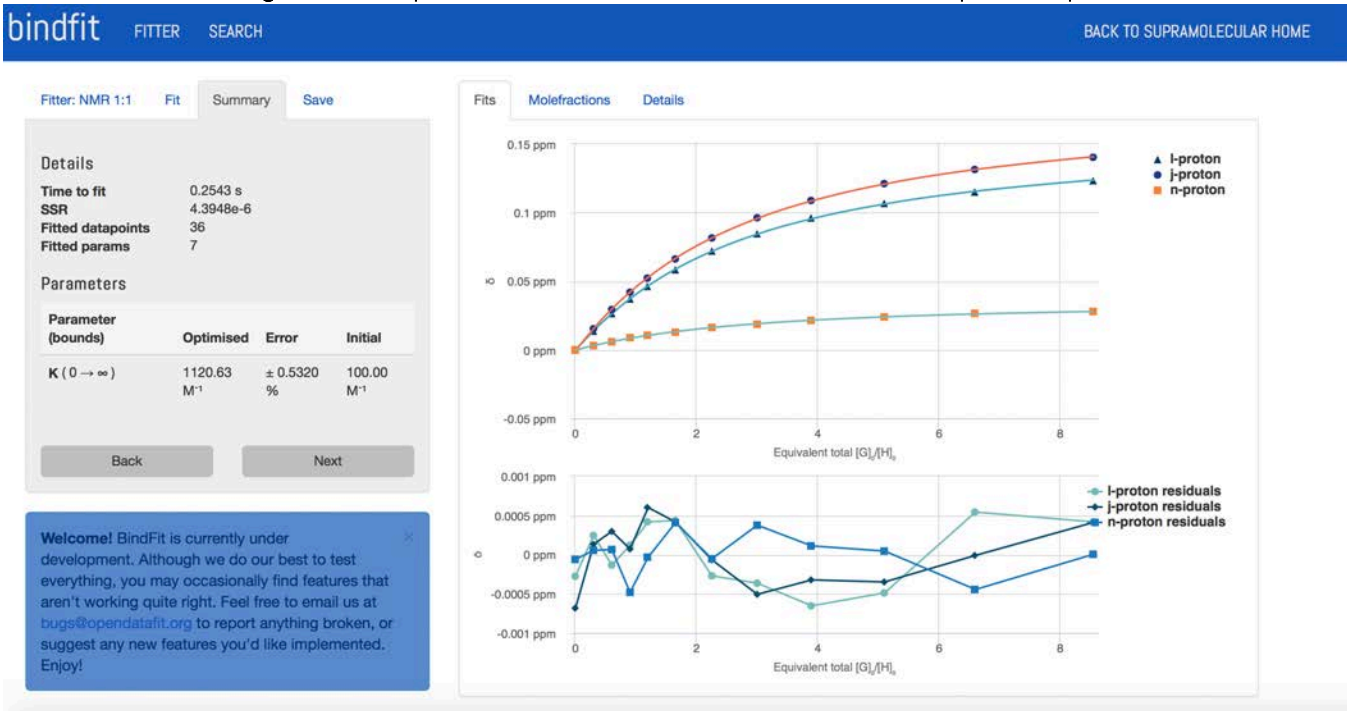

Figure S57. Fit of the data for H_j, H_n and H_l from Figure S56 to a 1:1 binding model.

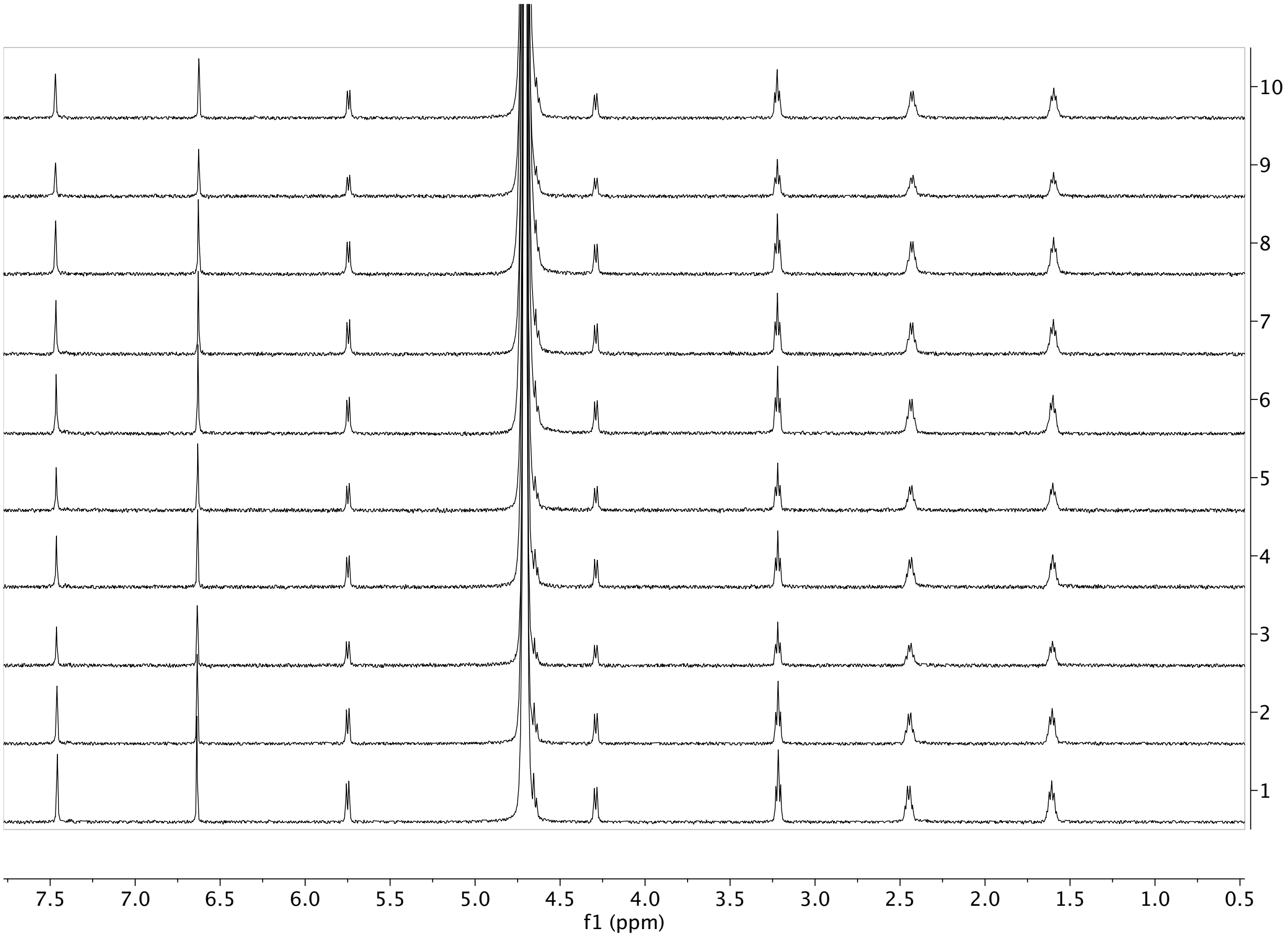

Figure S58. Representative ^{1}H NMR titration of **2** with 48 mM TfO^{-} up to 36 equiv.

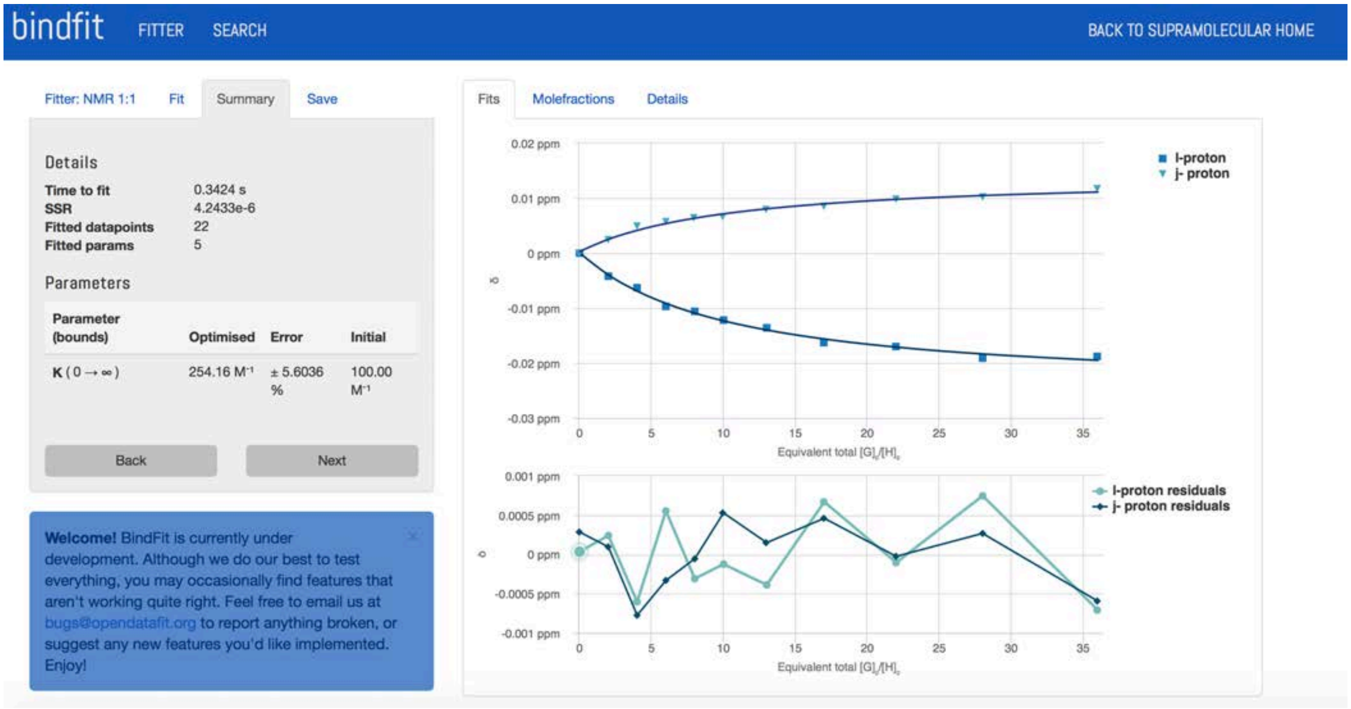

Figure S59. Fit of the data for H_j and H_l from Figure S58 to a 1:1 binding model.

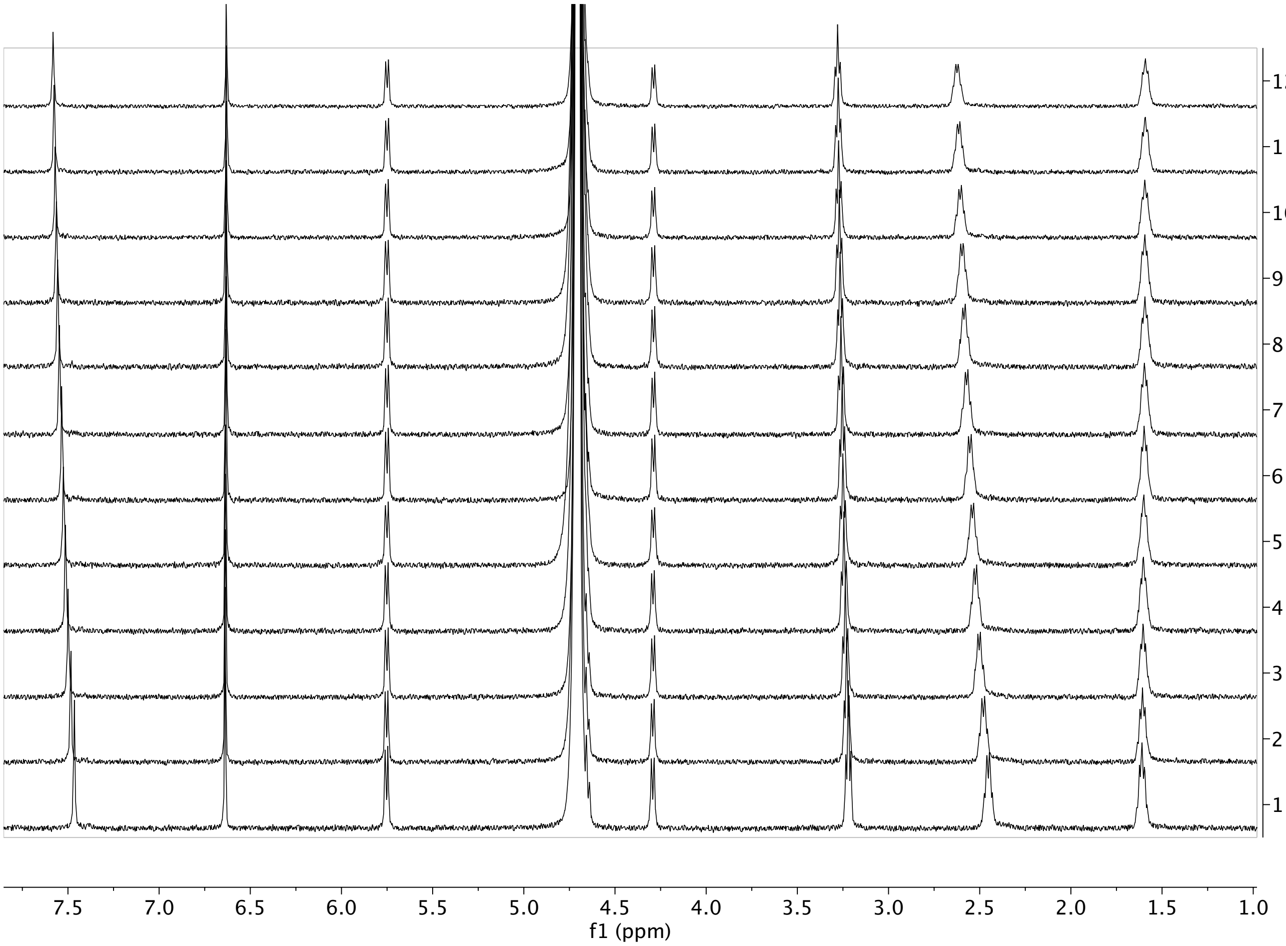

Figure S60. Representative ^{1}H NMR titration of **2** with 25 mM I^{-} up to 4.4 equiv.

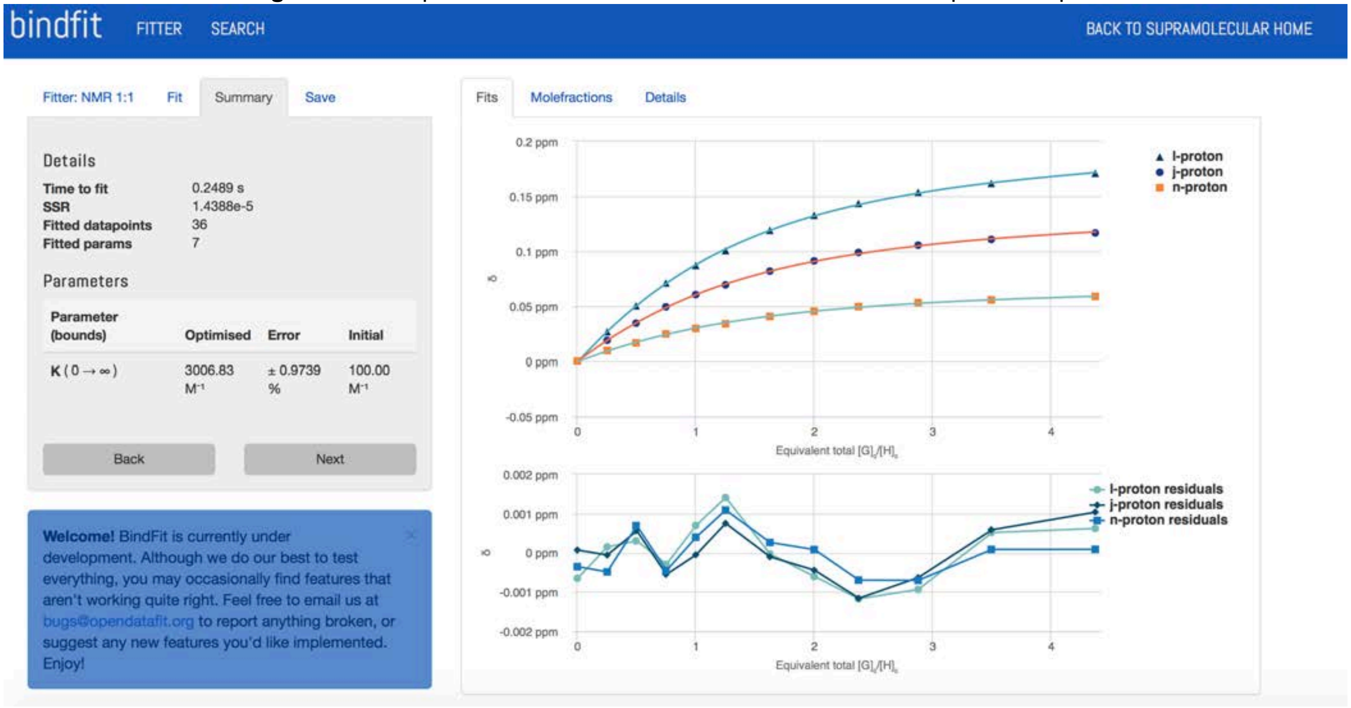

Figure S61. Fit of the data for H_j, H_n and H_l from Figure S60 to a 1:1 binding model.

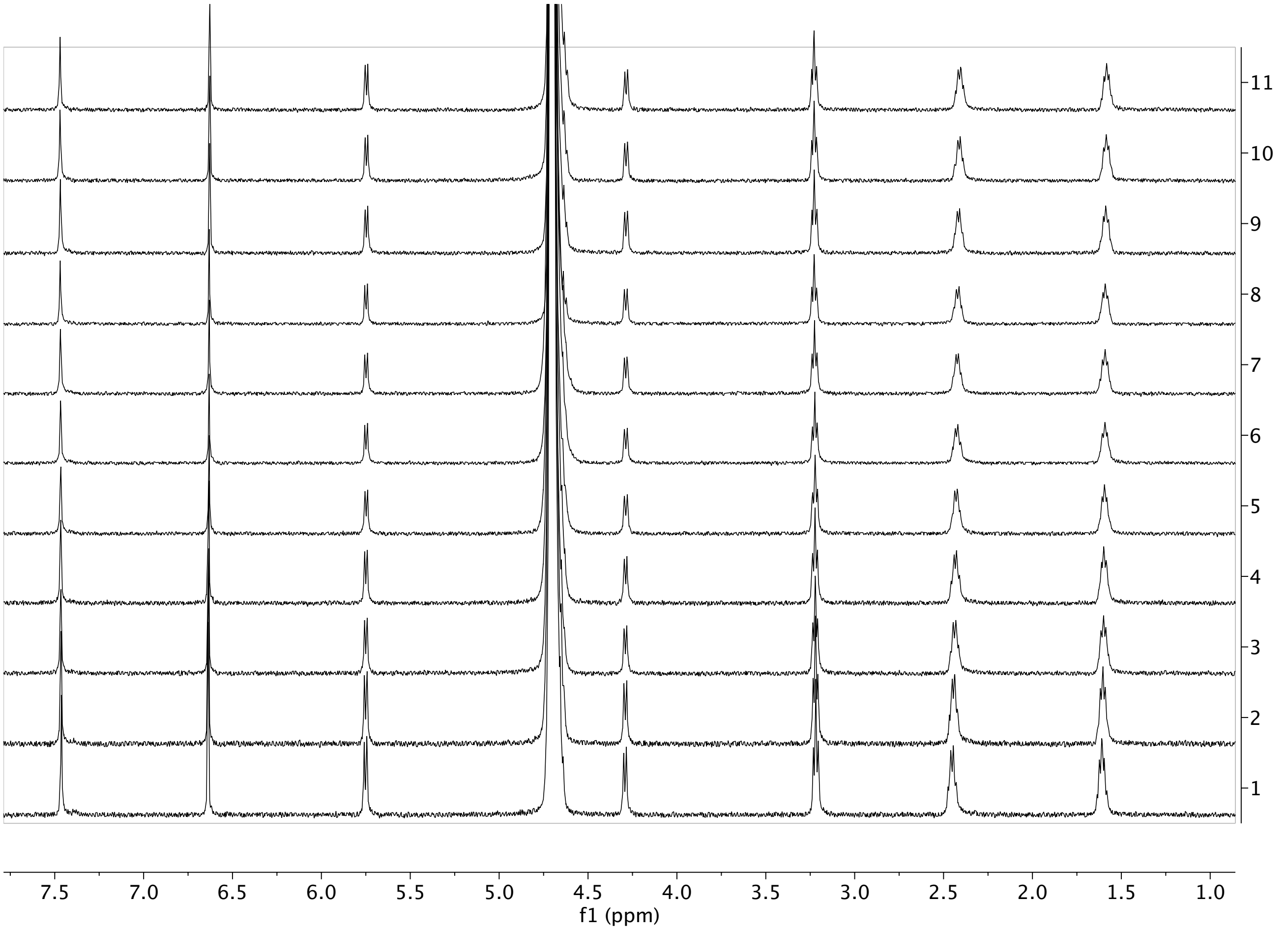

Figure S62. Representative ^{1}H NMR titration of **2** with 82 mM BF_4^- up to 14 equiv.

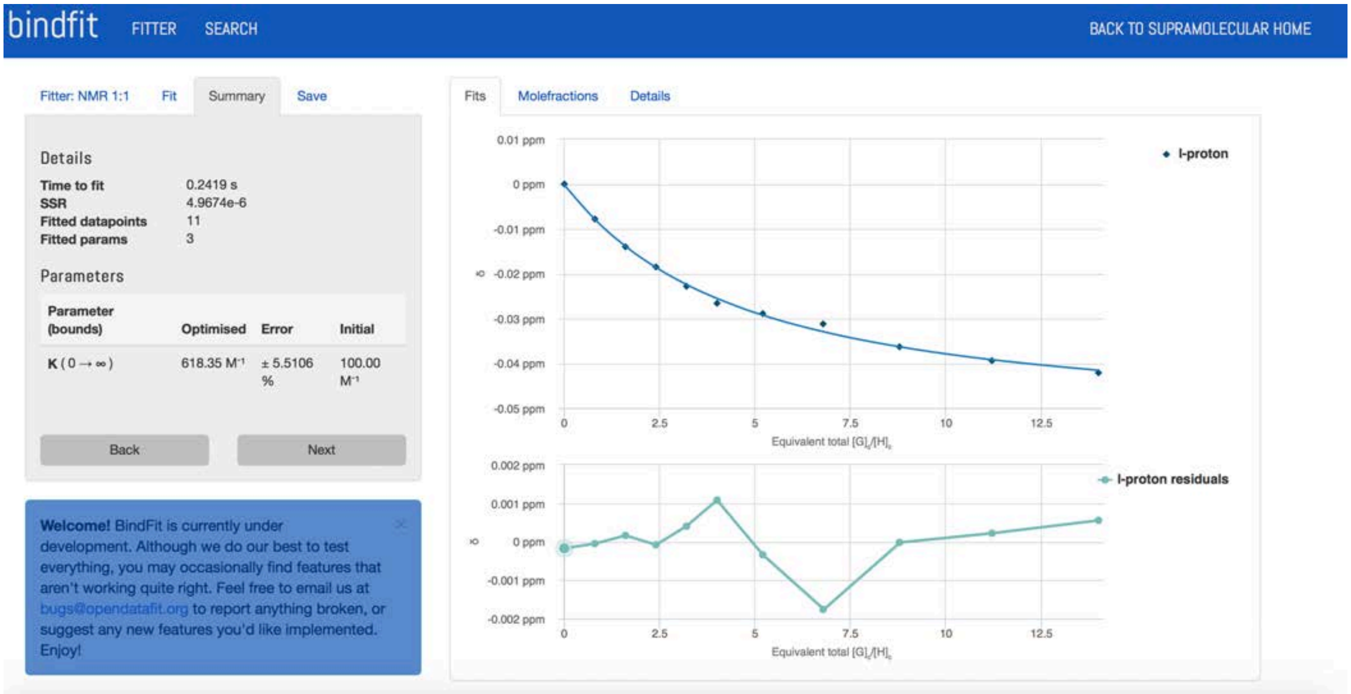

Figure S63. Fit of the data for H_I from Figure S62 to a 1:1 binding model.

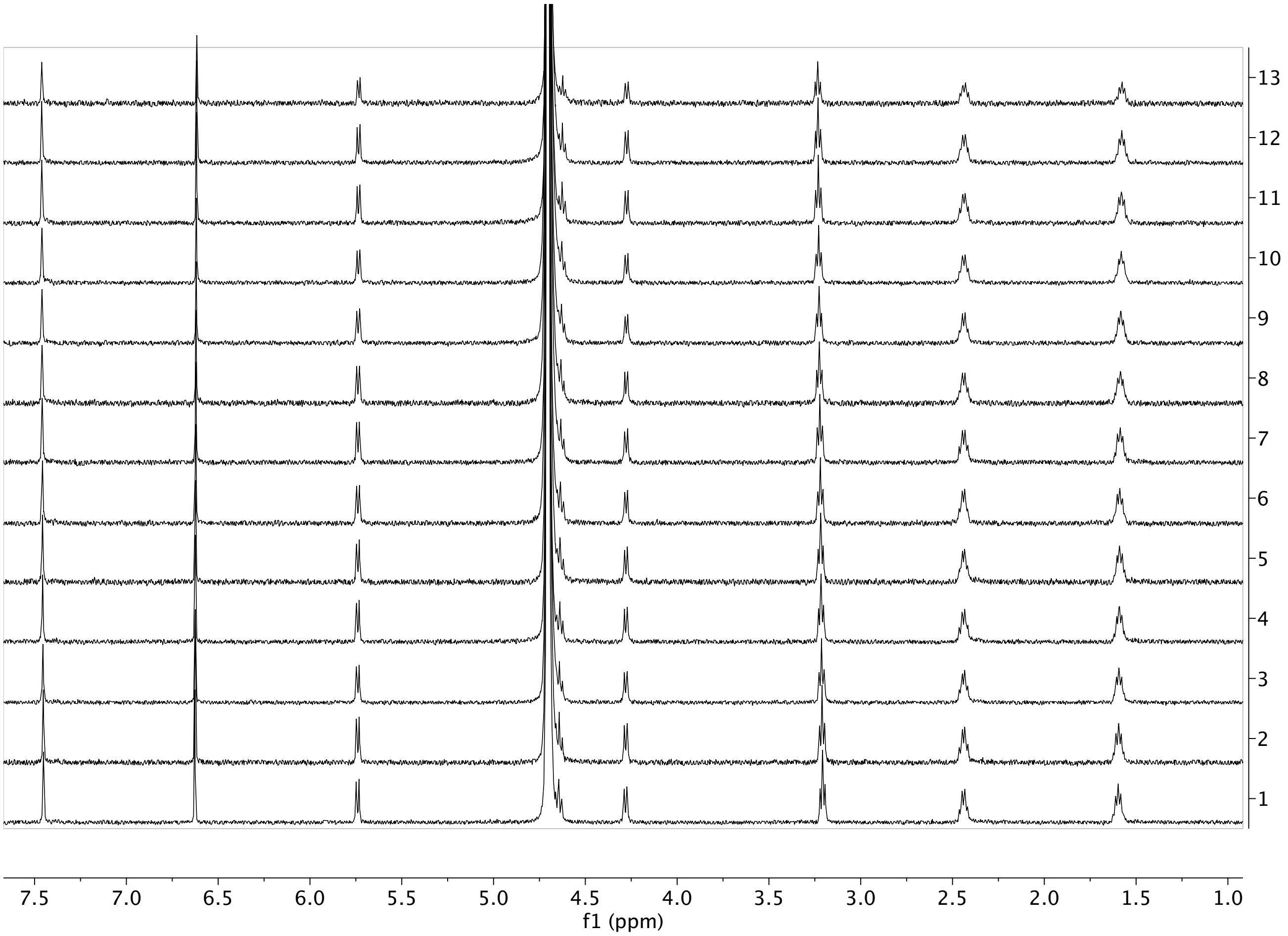

Figure S64. Representative ^{1}H NMR titration of **2** with 21 mM ClO_4^- up to 4.8 equiv.

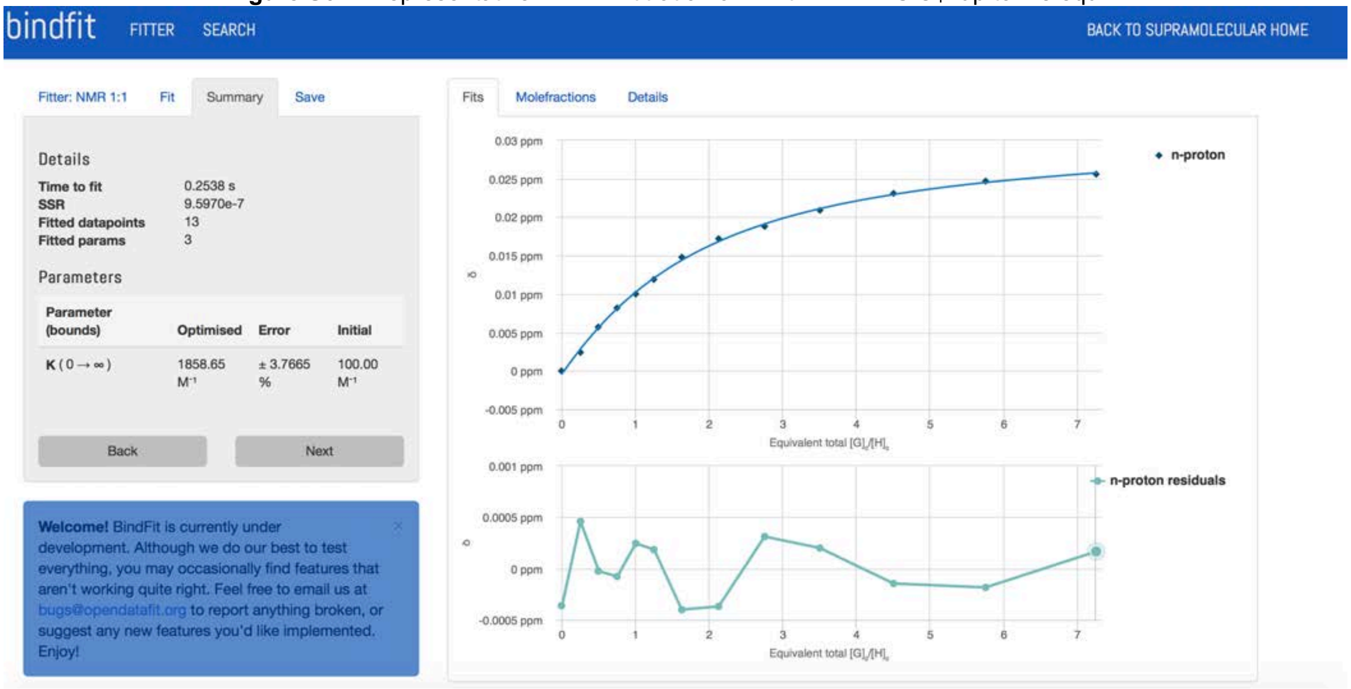

Figure S65. Fit of the data for H_n from Figure S64 to a 1:1 binding model.

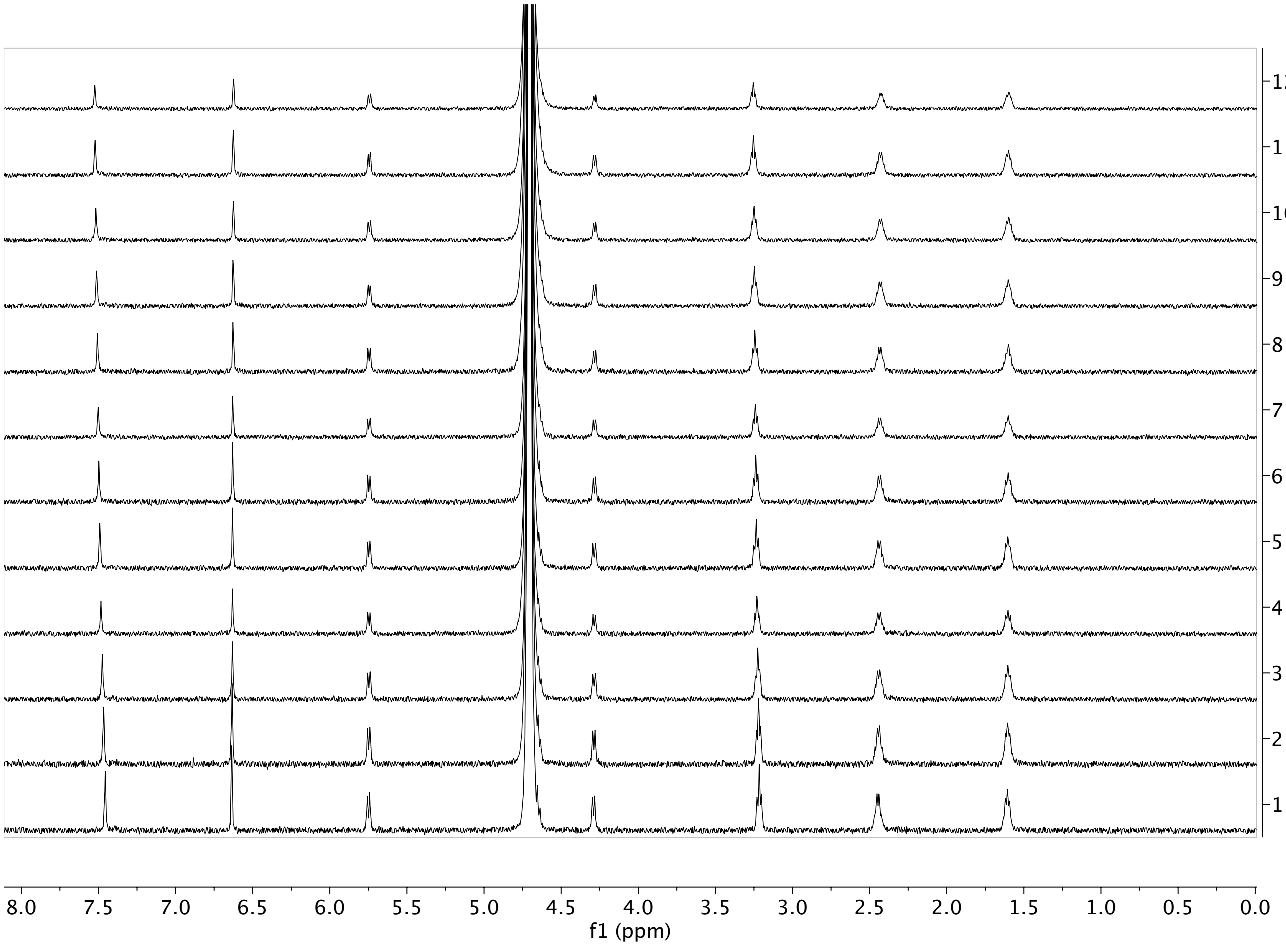

Figure S66. Representative ^{1}H NMR titration of **2** with 20 mM ReO_4^- up to 4.7 equiv.

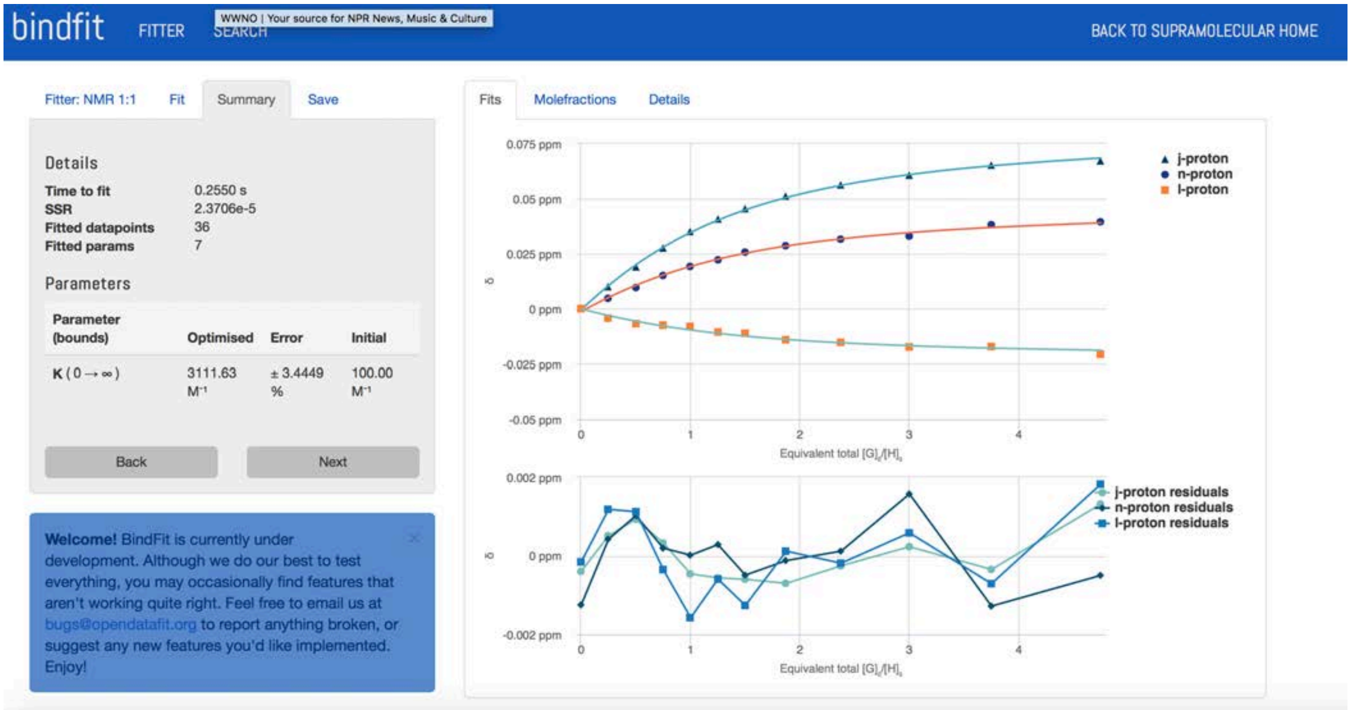

Figure S67. Fit of the data for H_j, H_n and H_l from Figure S66 to a 1:1 binding model.

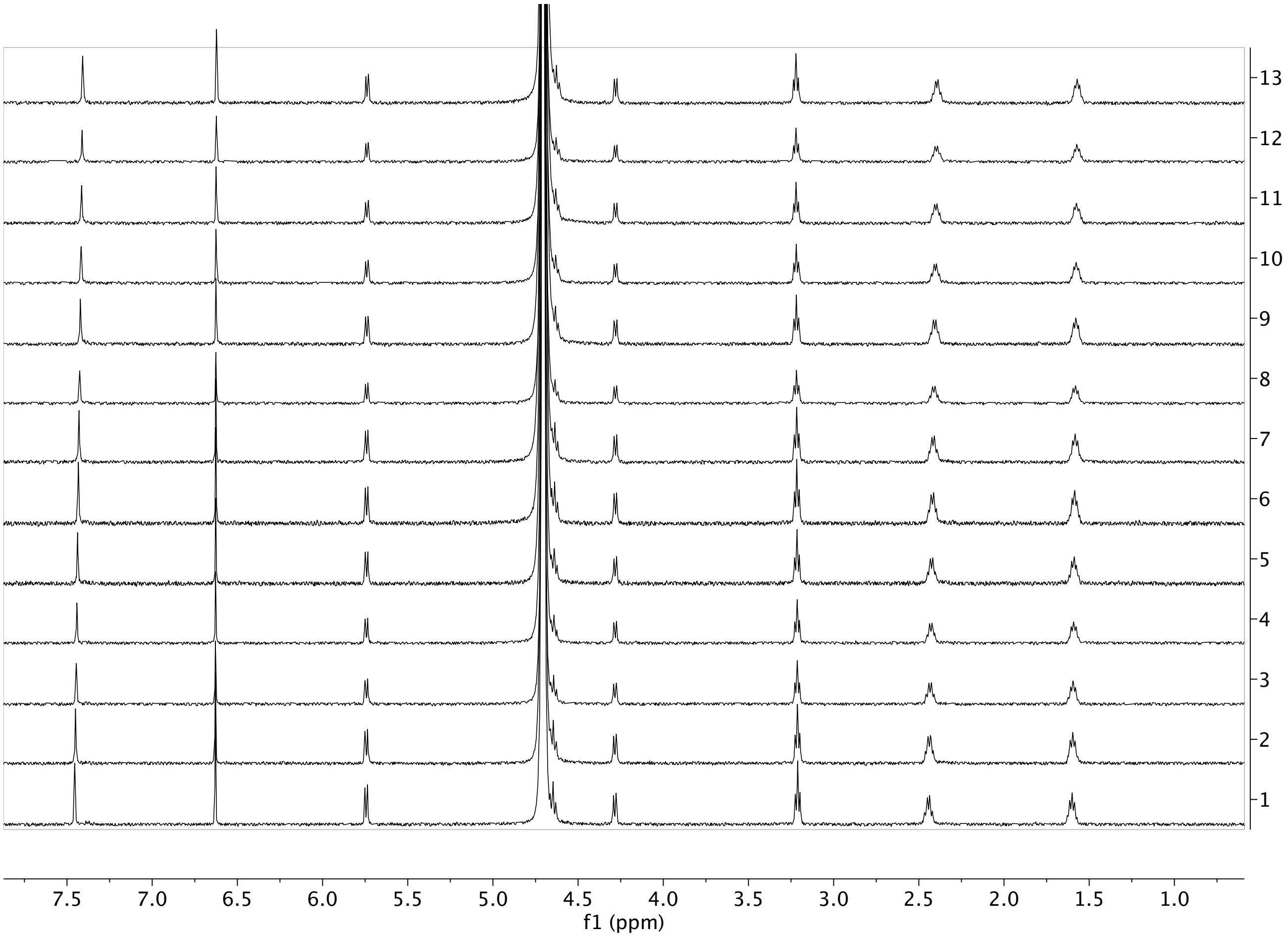

Figure S68. Representative ^{1}H NMR titration of **2** with 30 mM PF_6^- up to 7.1 equiv.

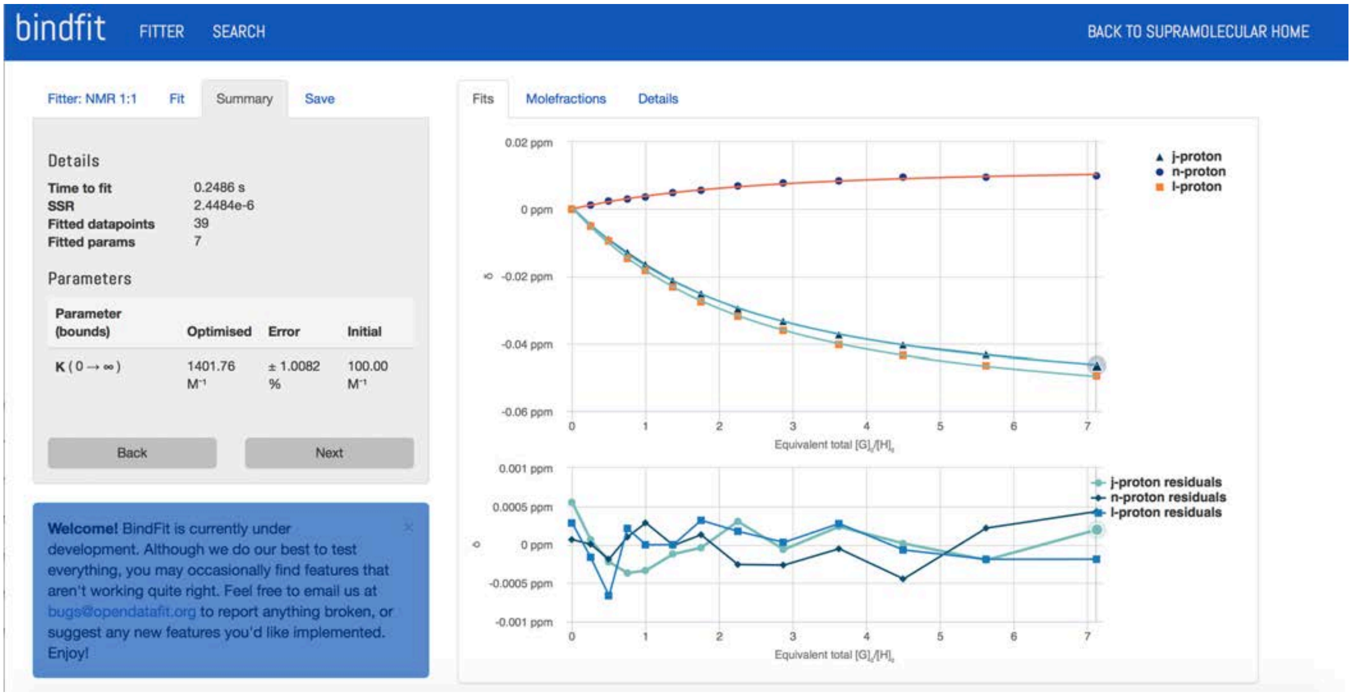

Figure S69. Fit of the data for H_j and H_l from Figure S68 to a 1:1 binding model.

Isothermal Titration Calorimetry Data

Solutions were prepared as described in the solution preparation procedure (Section 1.F). Isothermal Titration Calorimetric (ITC) experiments of hosts **1** and **2** with the selected salts were performed at 298 K using a VP-ITC MicroCalorimeter from Microcal, USA. Each run consisted of 28 consecutive injections of the solutions of guest into the ITC reaction cell charged with a solution of host (**1** or **2**). Experiments were run using a stirring speed of 450 rpm. Integration of the heat released and curve fitting of the resulting binding isotherm to a 1:1 binding model (stoichiometry confirmed by NMR) were performed using ORIGIN 7.0 software included in the VP-ITC. The titration experiments used the computer-controlled injection procedure with variable injection volumes of salt solution into host solution. The variable injection volumes protocols used for each guest are detailed in their respective sections. A time of 250 s between each injection was applied to enable equilibration. The first injection was discarded in all the experiments to remove the effect of guest solution diffusion through the syringe tip during initial equilibration. In all cases, the heat of dilution determined by titration of the solvent with salt in the absence of host was subtracted from the binding titration data prior to curve fitting.

As the Wiseman "c" parameter value (the product of the host concentration and the binding constant, K_a) with most host-guest complexes was < 10, the modified procedures defined by Turnbull[7] and Tellinghuisen[8] were followed. Briefly, variable injection volumes were used to titrate a large excess of salt to ensure host saturation at the end of the experiment. Additionally, based on the NMR data the stoichiometry parameter N was set to 1 during curve fitting. The same modification procedures have been used previously.[9, 10] The same anions were chosen for hosts **1** and **2**. Each experiment was repeated three times, and the K, ΔG, ΔH, $-T\Delta S$ values are reported as the averages in Table S2 for host **1** and **2**. Experimental error was ≤10%.

Host	Guest	K_a (M^{-1})	ΔG° (kJ/mol)	ΔH° (kJ/mol)	$-T\Delta S^\circ$ (kJ/mol)
**1**	ClO_4^-	1453	-18.0	-13.0	-5.0
	I^-	1830	-18.6	-18.0	-0.6
	TfO^-	162	-12.6	-4.4	-8.2
	Br^-	584	-15.8	-16.3	0.5
	PF_6^-	Poor fitting to 1:1 model			
	Cl^-	99	-11.4	-7.9	-3.5
**2**	ClO_4^-	1547	-18.2	-12.1	-6.1
	I^-	2193	-19.0	-18.2	-0.8
	PF_6^-	1363	-17.9	-8.0	-9.9
	Br^-	915	-16.9	-19.2	2.3
	Cl^-	195	-13.1	-12.1	-1.0
	TfO^-	Not enough heat released or too week binding			

Table S2. Thermodynamic data for the binding of sodium salt anions to hosts **1** and **2**. 10 mM sodium phosphate buffer, pH 3.0.

a. ITC protocols for titration of host **1** with salts

Experiments were conducted as described in Section 4.C. The individual injection sequences for titration of host **1** with various anions is shown below and a typical ITC experiment for each anion is shown in Figure S70 – Figure S74.

ClO_4^-: The ITC titration experiment used the computer-controlled 28-injection procedure of 40 mM guest solution into 0.25 mM host solution. The following variable injection volumes were used (μL); V_{1-6} = 3.0; V_{7-10} = 6.0; V_{11} = 9.0; V_{12-16} = 6.0; V_{17-28} = 9.0. DP was set at 15.

I^-: The ITC titration experiment used the computer-controlled 28-injection procedure of 40 mM guest solution into 0.25 mM host solution. The following variable injection volumes were used (μL); V_{1-6} = 3.0; V_{7-10} = 6.0; V_{11} = 9.0; V_{12-16} = 6.0; V_{17-28} = 9.0.

TfO^-: The ITC titration experiment used the computer-controlled 28-injection procedure of 100 mM guest solution into 1.0 mM host solution. The following variable injection volumes were used (μL); V_{1-6} = 3.0; V_{7-16} = 6.0; V_{17-28} = 9.0. DP was set at 15.

Br^-: The ITC titration experiment used the computer-controlled 28-injection procedure of 80 mM guest solution into 0.25 mM host solution. The following variable injection volumes were used (μL); V_{1-6} = 3.0; V_{7-16} = 6.0; V_{17-28} = 9.0. DP was set at 15.

Cl^-: The ITC titration experiment used the computer-controlled 28-injection procedure of 400 mM guest solution into 0.25 mM host solution. The following variable injection volumes were used (μL); V_{1-6} = 3.0; V_{7-16} = 6.0; V_{17-28} = 9.0. DP was set at 15.

b. ITC protocols for titration of host **2** with salts

Experiments were conducted as described in Section 4.C. The individual injection sequences for titration of host **2** with various anions is shown below and a typical ITC experiment for each anion is shown in Figure S75 – Figure S80.

ClO_4^-: The ITC titration experiment used the computer-controlled 28-injection procedure of 40 mM guest solution into 0.25 mM host solution. The following variable injection volumes were used (μL); V_{1-8} = 3.0; V_{9-16} = 6.0; V_{17-28} = 9.0. DP was set at 15.

I^-: The ITC titration experiment used the computer-controlled 28-injection procedure of 40 mM guest solution into 0.25 mM host solution. The following variable injection volumes were used (μL); V_{1-8} = 3.0; V_{9-16} = 6.0; V_{17-28} = 9.0. DP was set at 15.

TfO^-: The ITC titration experiment used the computer-controlled 28-injection procedure of 400 mM guest solution into 1.0 mM host solution. The following variable injection volumes were used (μL); V_{1-6} = 3.0; V_{7-16} = 6.0; V_{17-28} = 9.0. DP was set at 15.

PF_6^-: The ITC titration experiment used the computer-controlled 28-injection procedure of 20 mM guest solution into 0.25 mM host solution. The following variable injection volumes were used (μL); V_{1-8} = 3.0; V_{9-16} = 6.0; V_{17-28} = 9.0. DP was set at 15.

Br^-: The ITC titration experiment used the computer-controlled 28-injection procedure of 60 mM guest solution into 0.25 mM host solution. The following variable injection volumes were used (μL); V_{1-6} = 3.0; V_{7-16} = 6.0; V_{17-28} = 9.0. DP was set at 15.

Cl^-: The ITC titration experiment used the computer-controlled 28-injection procedure of 400 mM guest solution into 0.25 mM host solution. The following variable injection volumes were used (μL); V_{1-6} = 3.0; V_{7-16} = 6.0; V_{17-28} = 9.0. DP was set at 15.

c. ITC Data for titration of host **1** with salts

Perchlorate

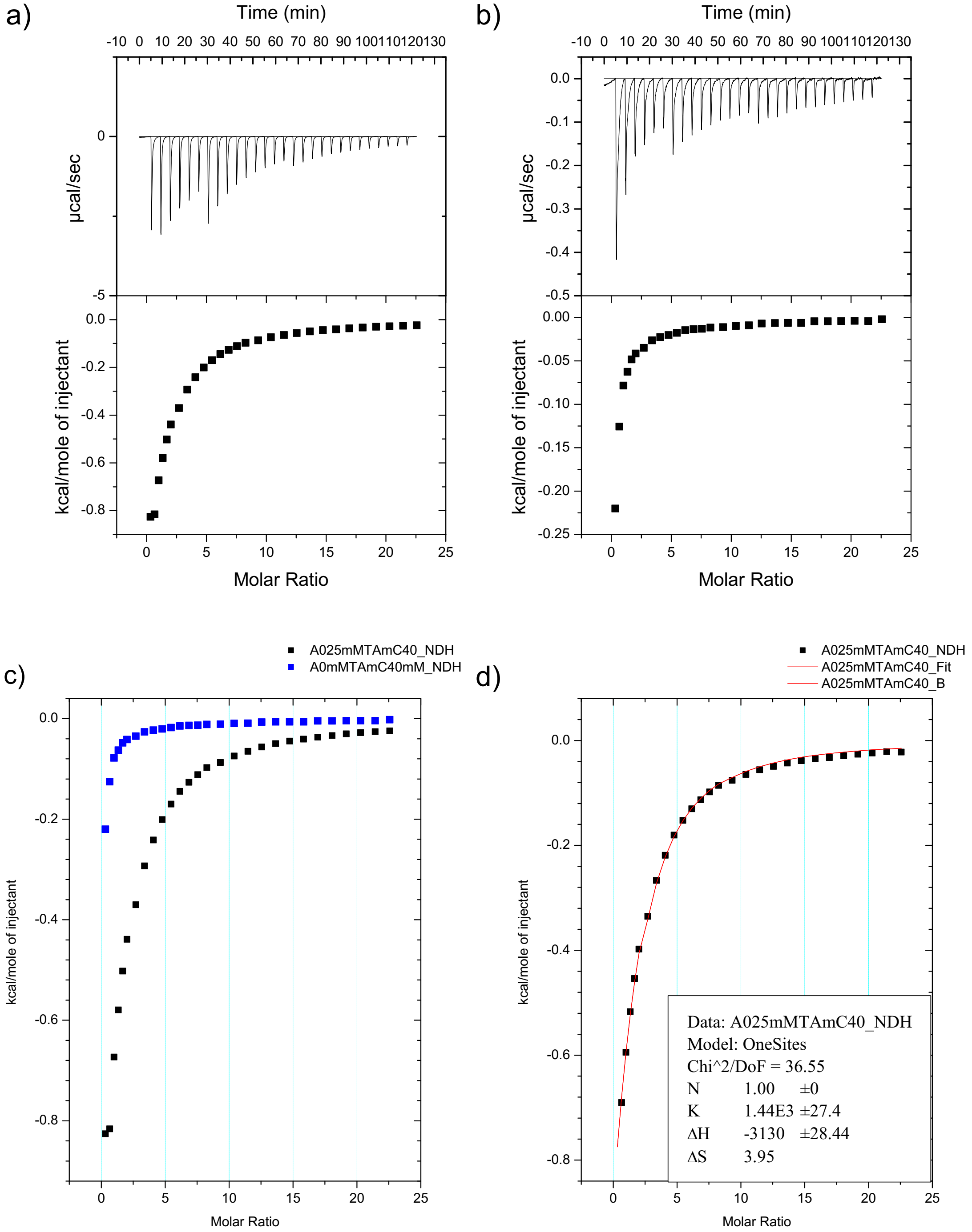

Figure S70. (a) ITC data for the complex formed between **1** and sodium perchlorate. A 40 mM solution of sodium perchlorate was titrated into 0.25 mM solution of **1** equilibrated at 25 ˚C. Both host and guest were in 40 mM phosphate buffer, pH 3.0. (b) ITC titration of sodium perchlorate into buffer alone. (c) An overlay of the ITC data shown in (a) and (b). (d) The resultant final binding curve after subtracting (a) from (b). N-value was set to 1 during curve fitting.

Iodide

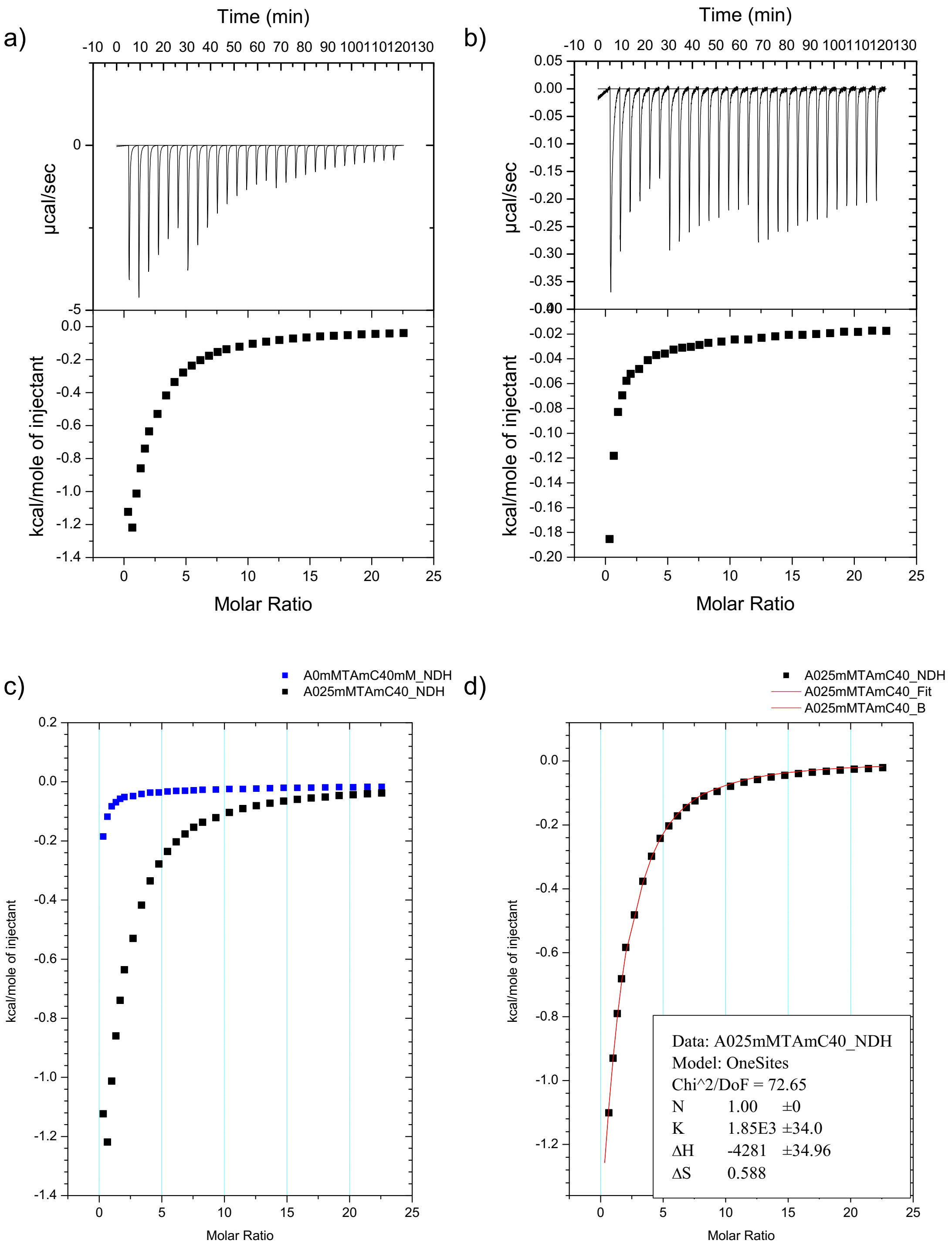

Figure S71. (a) ITC data for the complex formed between **1** and sodium iodide. A 40 mM solution of sodium iodide was titrated into 0.25 mM solution of **1** equilibrated at 25 ˚C. Both host and guest were in 10 mM phosphate buffer, pH 3.0. (b) ITC titration of sodium iodide into buffer alone. (c) An overlay of the ITC data shown in (a) and (b). (d) The resultant final binding curve after subtracting (a) from (b). N-value was set to 1 during curve fitting.

Triflate

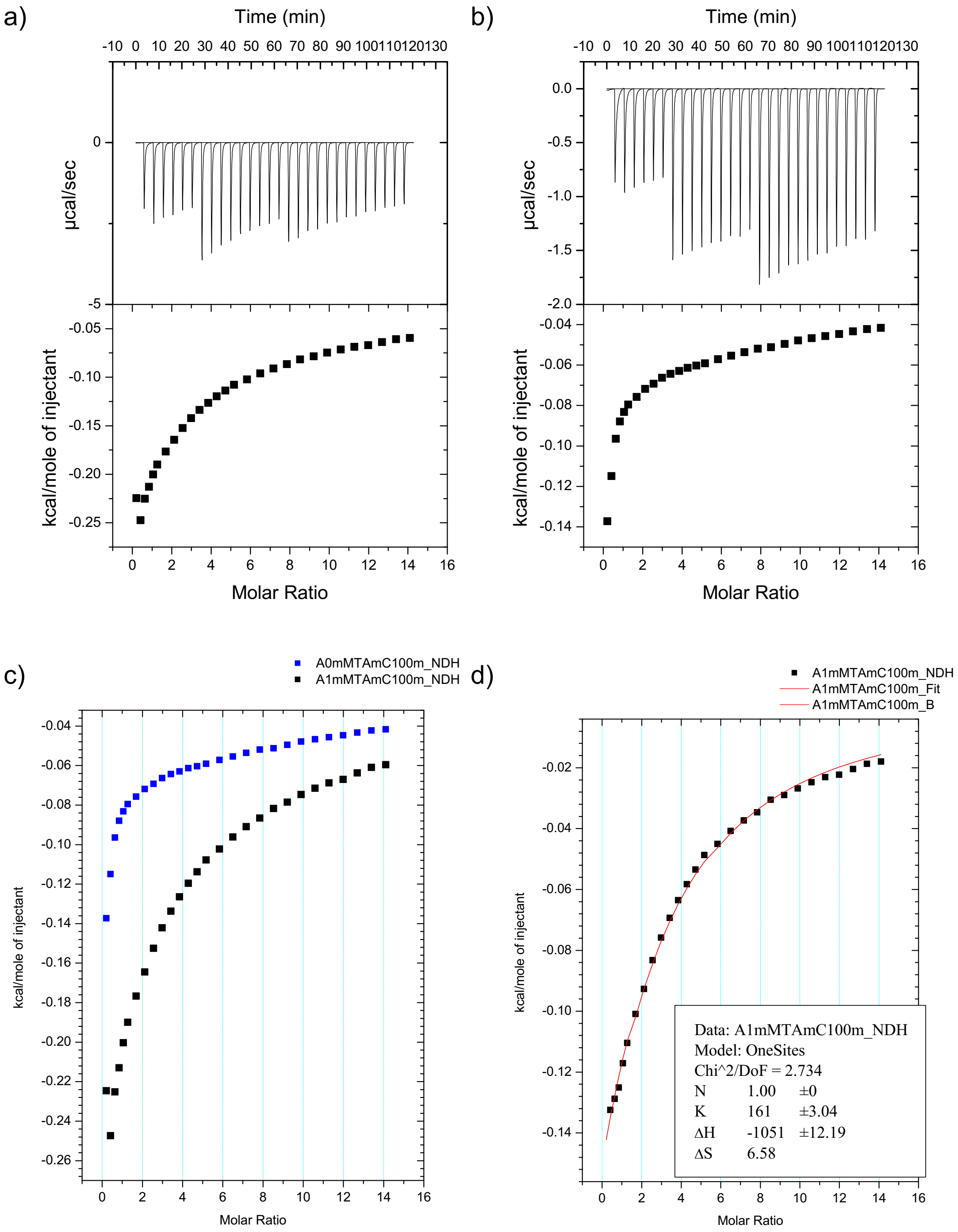

Figure S72. (a) ITC data for the complex formed between **1** and sodium triflate. A 100 mM solution of sodium triflate was titrated into 1.0 mM solution of **1** equilibrated at 25 ˚C. Both host and guest were in 10 mM phosphate buffer, pH 3.0. (b) ITC titration of sodium triflate into buffer alone. (c) An overlay of the ITC data shown in (a) and (b). (d) The resultant final binding curve after subtracting (a) from (b). N-value was set to 1 during curve fitting.

Bromide

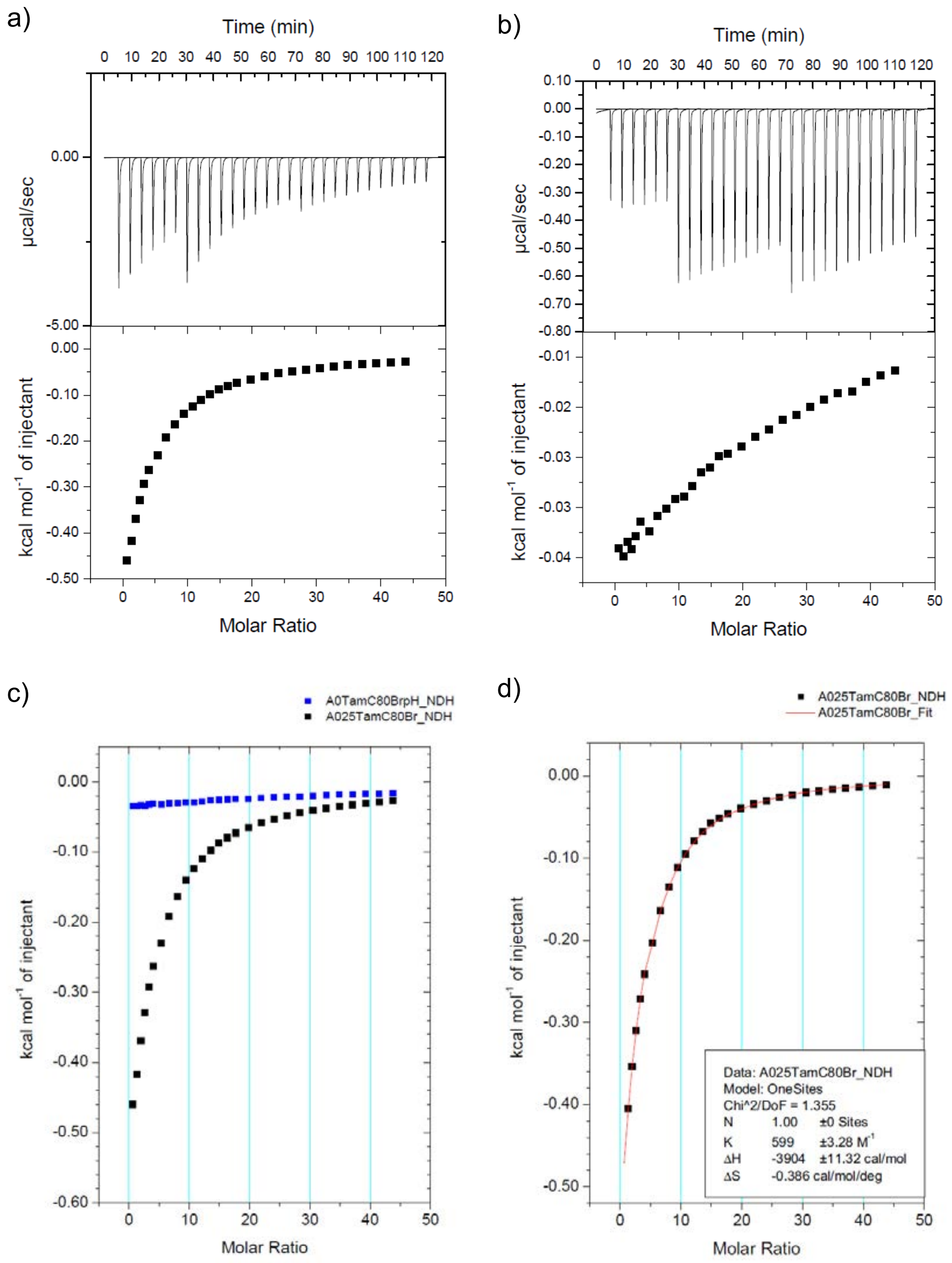

Figure S73. (a) ITC data for the complex formed between **1** and sodium bromide. An 80 mM solution of sodium bromide was titrated into 0.25 mM solution of **1** equilibrated at 25 ˚C. Both host and guest were in 10 mM phosphate buffer, pH 3.0. (b) ITC titration of sodium bromide into buffer alone. (c) An overlay of the ITC data shown in (a) and (b). (d) The resultant final binding curve after subtracting (a) from (b). N-value was set to 1 during curve fitting.

Chloride

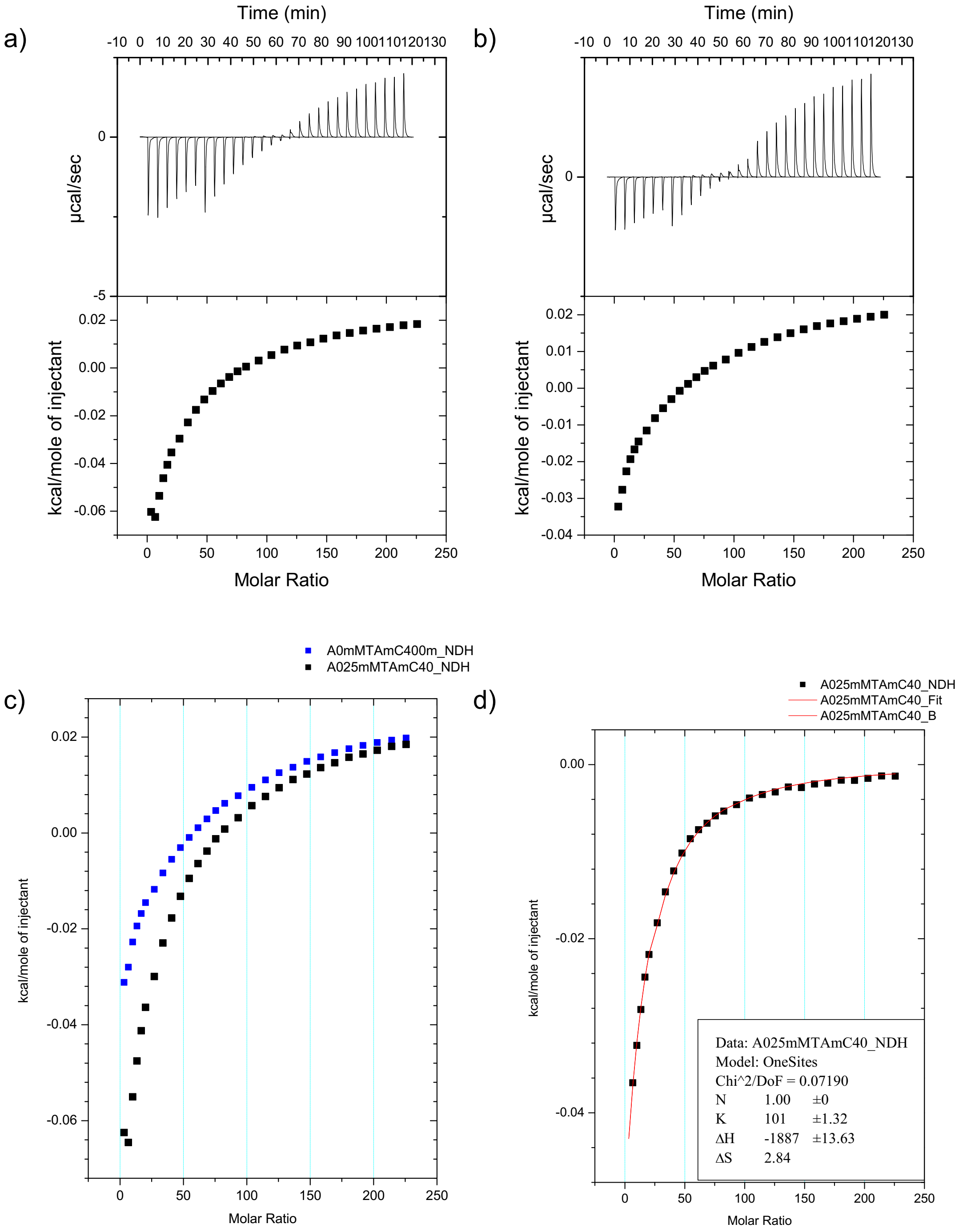

Figure S74. (a) ITC data for the complex formed between **1** and sodium chloride. A 400 mM solution of sodium chloride was titrated into 0.25 mM solution of **1** equilibrated at 25 ˚C. Both host and guest were in 10 mM phosphate buffer, pH 3.0. (b) ITC titration of sodium chloride into buffer alone. (c) An overlay of the ITC data shown in (a) and (b). (d) The resultant final binding curve after subtracting (a) from (b). N-value was set to 1 during curve fitting.

d. ITC data for titration of host **2** with salts

Perchlorate

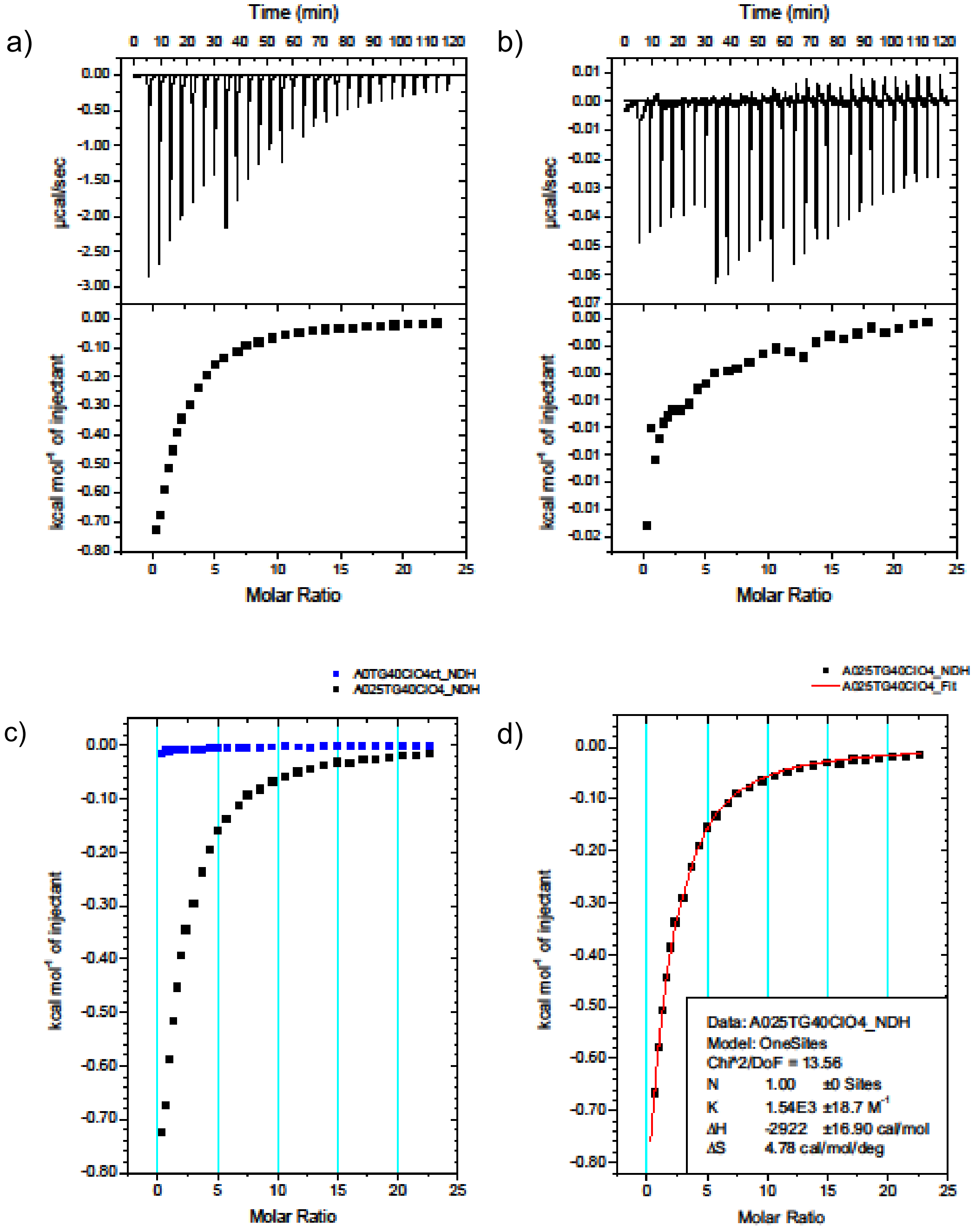

Figure S75. (a) ITC data for the complex formed between **2** and sodium perchlorate. A 40 mM solution of sodium perchlorate was titrated into 0.25 mM solution of **2** equilibrated at 25 ˚C. Both host and guest were in 10 mM phosphate buffer, pH 3.0. (b) ITC titration of sodium perchlorate into buffer alone. (c) An overlay of the ITC data shown in (a) and (b). (d) The resultant final binding curve after subtracting (a) from (b). N-value was set to 1 during curve fitting.

Iodide

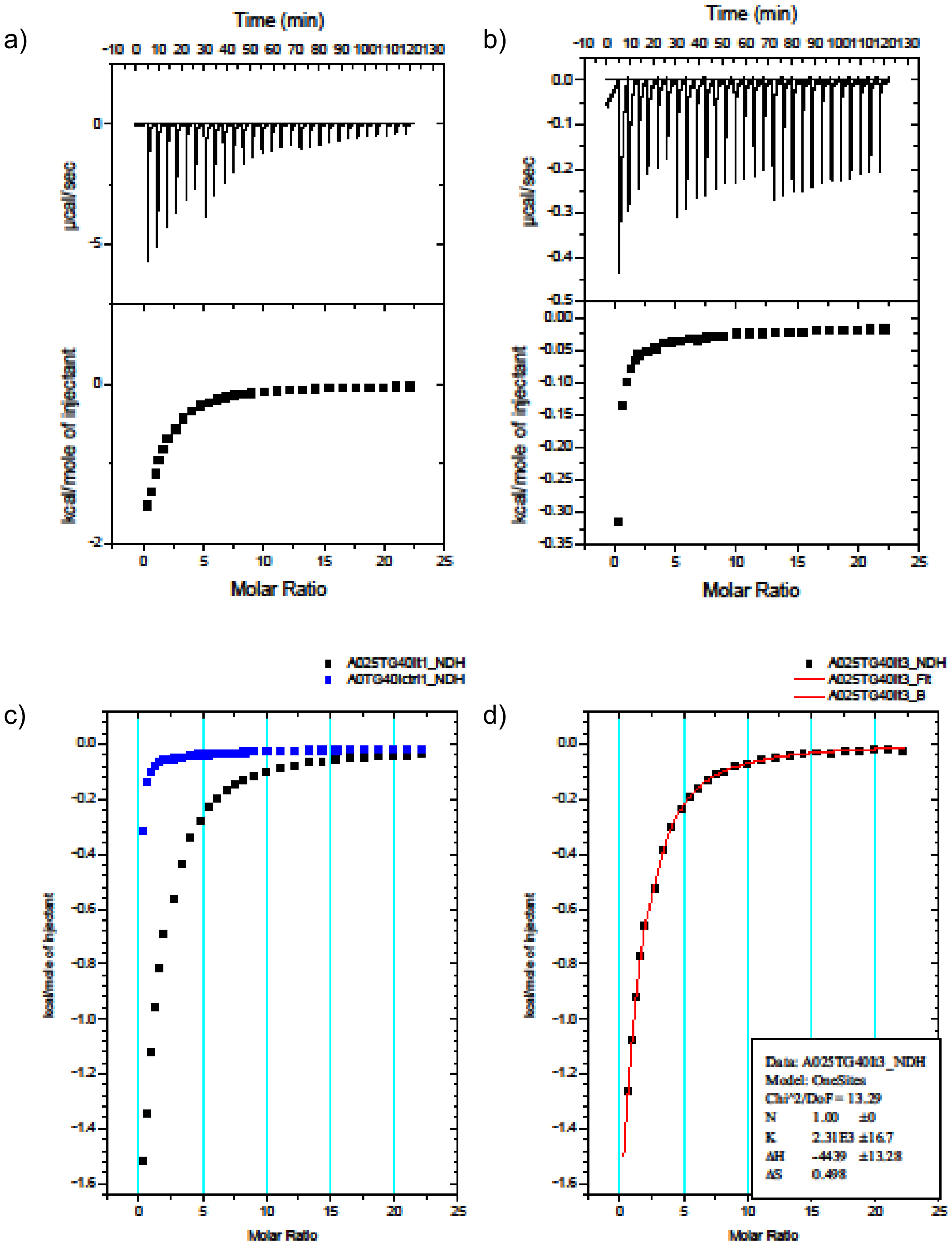

Figure S76. (a) ITC data for the complex formed between **2** and sodium iodide. A 40 mM solution of sodium iodide was titrated into 0.25 mM solution of **2** equilibrated at 25 ˚C. Both host and guest were in 10 mM phosphate buffer, pH 3.0. (b) ITC titration of sodium iodide into buffer alone. (c) An overlay of the ITC data shown in (a) and (b). (d) The resultant final binding curve after subtracting (a) from (b). N-value was set to 1 during curve fitting.

Triflate

a)

b)

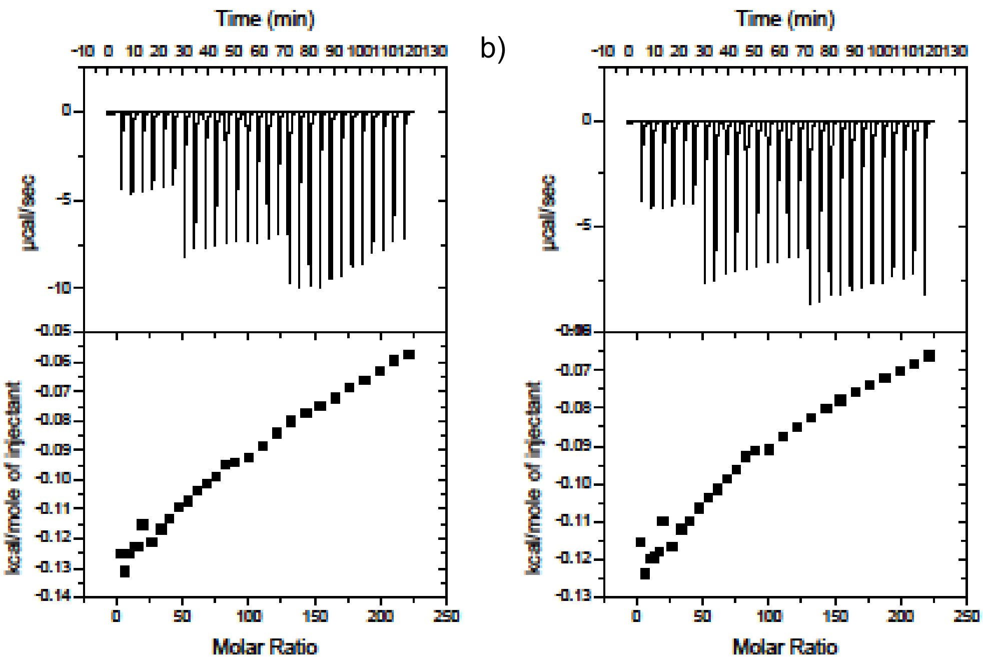

c)

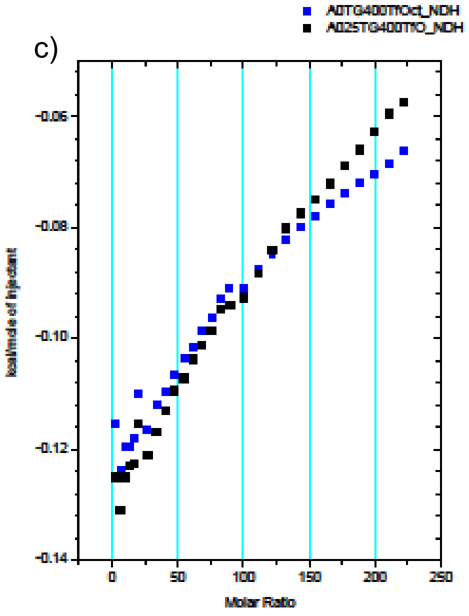

Figure S77. (a) ITC data for the complex formed between **2** and sodium triflate. A 400 mM solution of sodium triflate was titrated into 0.25 mM solution of **2** equilibrated at 25 ˚C. Both host and guest were in 10 mM phosphate buffer, pH 3.0. (b) ITC titration of sodium triflate into buffer alone. (c) An overlay of (a) and (b) ITC data.

Bromide

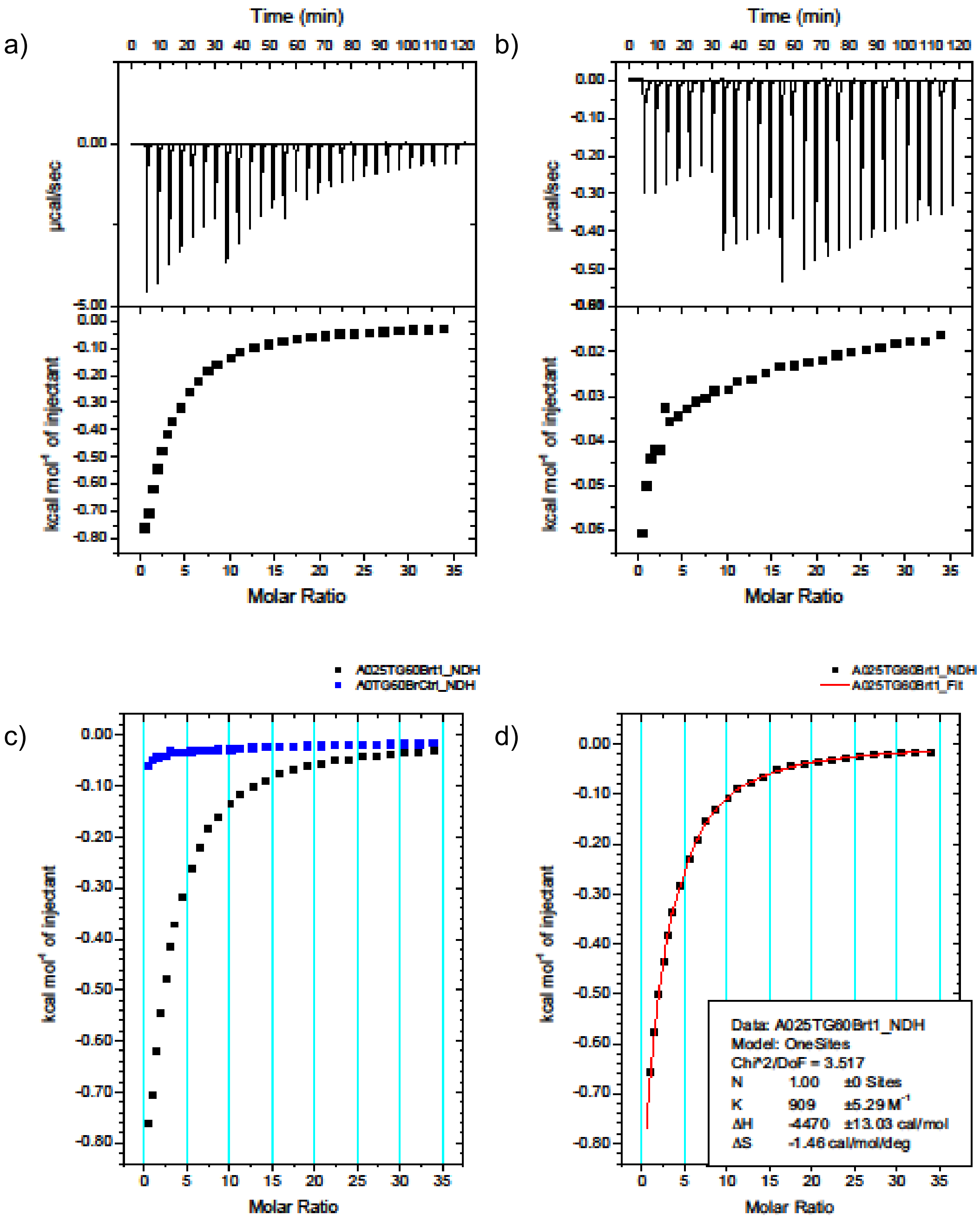

Figure S78. (a) ITC data for the complex formed between **2** and sodium bromide. A 400 mM solution of sodium chloride was titrated into 0.25 mM solution of **2** equilibrated at 25 ˚C. Both host and guest were in 10 mM phosphate buffer, pH 7.30. (b) ITC titration of sodium chloride into buffer alone. (c) An overlay of the ITC data shown in (a) and (b). (d) The resultant final binding curve after subtracting (a) from (b). N-value was set to 1 during curve fitting.

Chloride

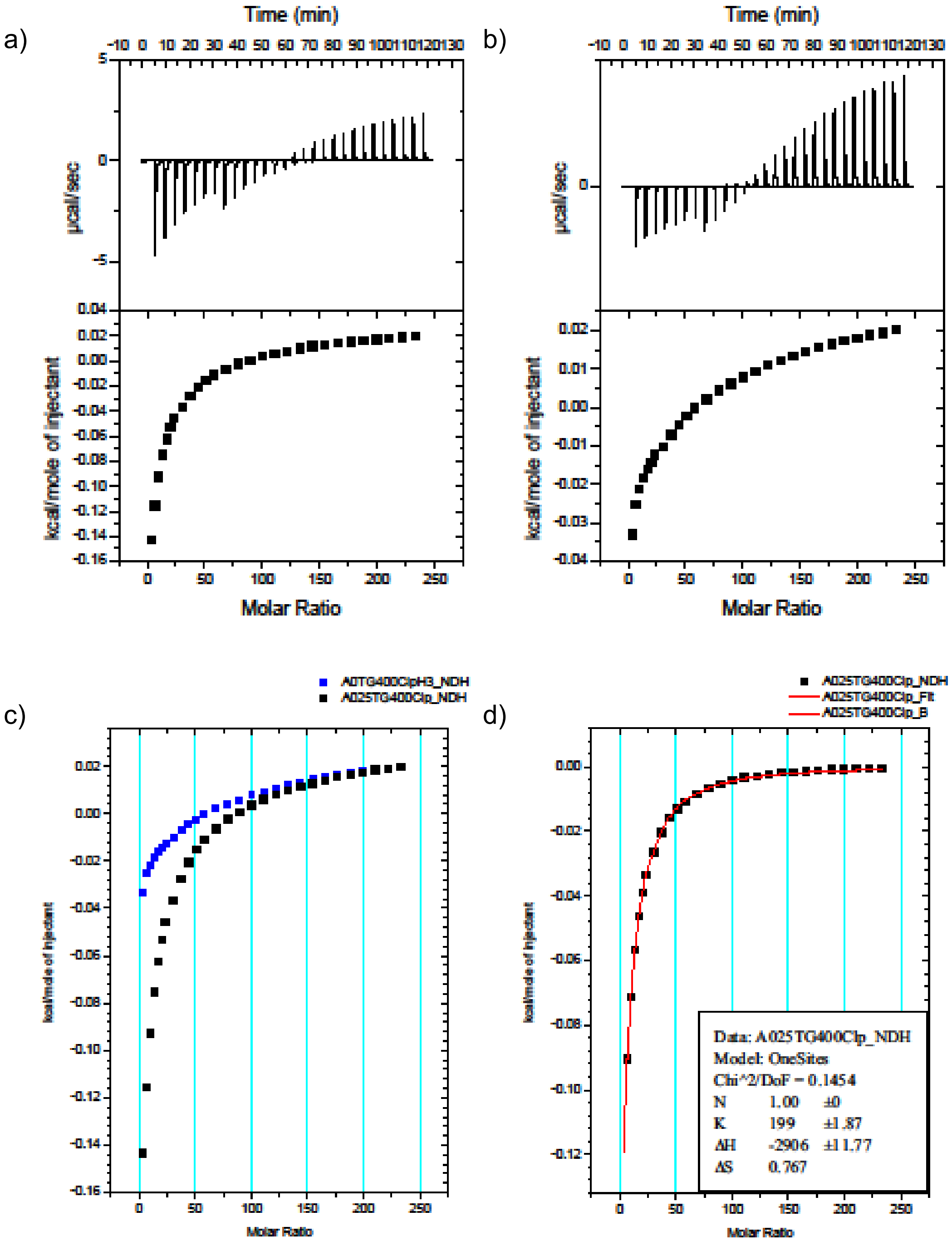

Figure S79. (a) ITC data for the complex formed between **2** and sodium chloride. A 400 mM solution of sodium chloride was titrated into 0.25 mM solution of **2** equilibrated at 25 ˚C. Both host and guest were in 10 mM phosphate buffer, pH 3.0. (b) ITC titration of sodium chloride into buffer alone. (c) An overlay of the ITC data shown in (a) and (b). (d) The resultant final binding curve after subtracting (a) from (b). N-value was set to 1 during curve fitting.

Hexafluorophosphate

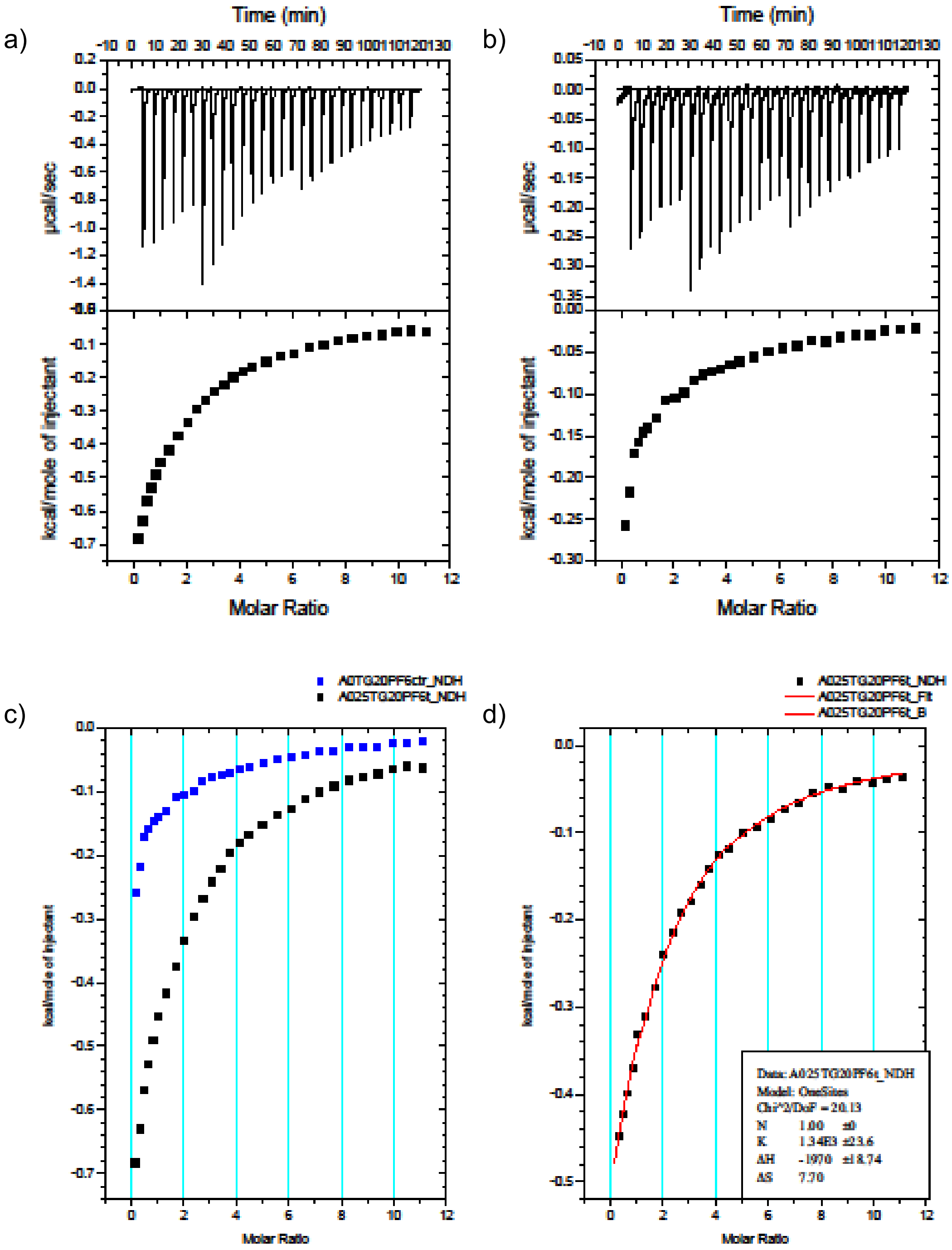

Figure S80. (a) ITC data for the complex formed between **2** and sodium hexafluorophosphate. A 20 mM solution of sodium hexafluorophosphate was titrated into 0.25 mM solution of **2** equilibrated at 25 ˚C. Both host and guest were in 10 mM phosphate buffer, pH 3.0. (b) ITC titration of sodium chloride into buffer alone. (c) An overlay of the ITC data shown in (a) and (b). (d) The resultant final binding curve after subtracting (a) from (b). N-value was set to 1 during curve fitting.

Critical precipitation assay data

Solutions were prepared as described in the sample preparation section. The total mixing time for each plate was <3.0 min. Plates were allowed to rest for 15 min and then read using an Enspire multimode plate reader at 500 nm equipped with a temperature controller set to 23 °C. Solution turbidity was assessed as an increase in absorbance relative to baseline values at 500 nm, reference-subtracted and normalized values were then plotted using OriginPro software. The two types of assays were precipitation screening, and critical precipitation concentration (CPC) determination.

a. Precipitation Screening

For screening assays, the hosts were prepared at twice the desired final concentration and added to wells containing prediluted salt solutions. Figure S81, shows the plot that reflects the reference subtracted and normalized screening data for hosts **1**. To further quantify to what extent each anion results in precipitation of the host, UV-Vis CPC determinations were conducted in at least triplicate based on the screening results for investigated anions.[11]

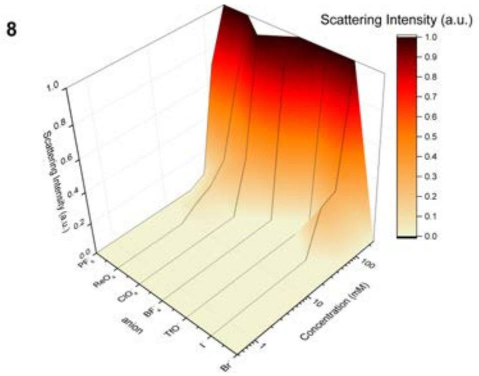

Figure S81. Surface plot of the normalized absorption intensity for host **1** in the presence of various anions as measured by UV-Vis spectroscopy; 10 mM phosphate buffer, pH 3.0.

b. Critical Precipitation Concentration (CPC) Determination

The bounds for precipitation were determined as the lowest concentration well containing detectable precipitate and the highest concentration well not containing precipitate.

Ranges for determination of the individual critical precipitation concentration (CPC) for each host fall between the lowest concentration well containing detectable precipitate and the highest concentration well not containing precipitate. For CPC determinations, measurements were performed in at least triplicate from separate stock solutions of hosts and salts. Wells were tested from ~10–40 % of the concentration of a stock salt solution. Dilutions were maintained at 4–6% increments of ½ the stock salt solution concentration (1–1.5 µL increments, 11 steps total) and buffer added for a final volume of 25 µL before addition of host. For CPC determination, 25 µL of host (2 × M_f) were added to wells containing 5–20 µL buffer, and 5–20 µL of salts of variable concentration for a final volume of 50 µL. Plate background was measured from eight non-precipitating control wells containing host and buffer for each plate and was typically ~0.038 ± 0.002. The resulting absorbance was plotted as a function of salt concentration. The wavelength selected was chosen to minimize interference from the intrinsic absorbance of the anions and to give sufficient signal for data analysis. Changes in reference subtracted absorbance levels associated with signal to noise (S/N) ratios of >5.0 (≈ +0.010) were considered significant. Precipitation concentrations were determined by averaging the concentration at which significant solution turbidity was measured with respect to the background. The resulting plots were then averaged, and the uncorrected data was plotted using OriginPro10.6 software and the results are summarized in Table S3.

Table S3. Summary of critical precipitation concentration data from turbidity assay by UV-Vis experiments. CPC was determined from at least three measurements from separate experiments. 10 mM phosphate buffer pH = 3.0.

**Anion**	Host 1			Host 2		
	CPC_{turb}	St.Dev	CV (%)	CPC_{turb}	St.Dev	CV (%)
PF_6^-	64.0	6.3	10	8.3	1.0	12
ReO_4^-	8.0	0.8	10	4.7	0.4	8.7
ClO_4^-	28.0	3.4	12	30.0	1.8	6.0
BF_4^-	154.0	11.0	7.0	51.0	1.8	3.5
TfO^-	30.0	2.7	9.0	20.0	1.8	8.8
I^-	72.0	5.9	8.2	59.0	3.5	6.0
NO_3^-	115.0	7.5	6.6	49.0	3.9	8.0
Br^-	—[a]	—	—	142.0	7.6	5.3

a) No precipitation occurred up to ~ 250 mM salt.

c. Relationship between CPC and thermodynamics of binding and ionic radii

Possible correlations were examined in the relationship between the CPC and: K_a, ΔH, $-T\Delta S$ for guest complexation, and the ionic radii of the guests. These are shown below in Figure S82 – Figure S85.

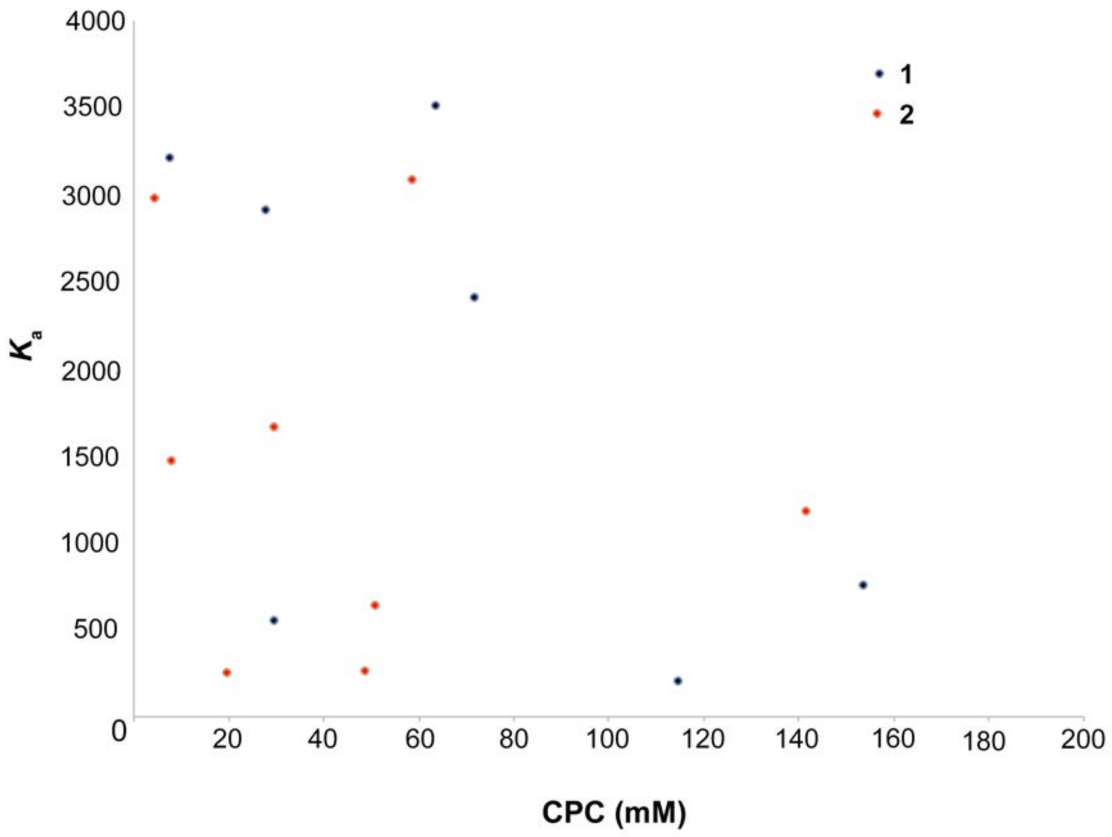

Figure S82. Plot of the CPC value for an anionic guest and its affinity constant.

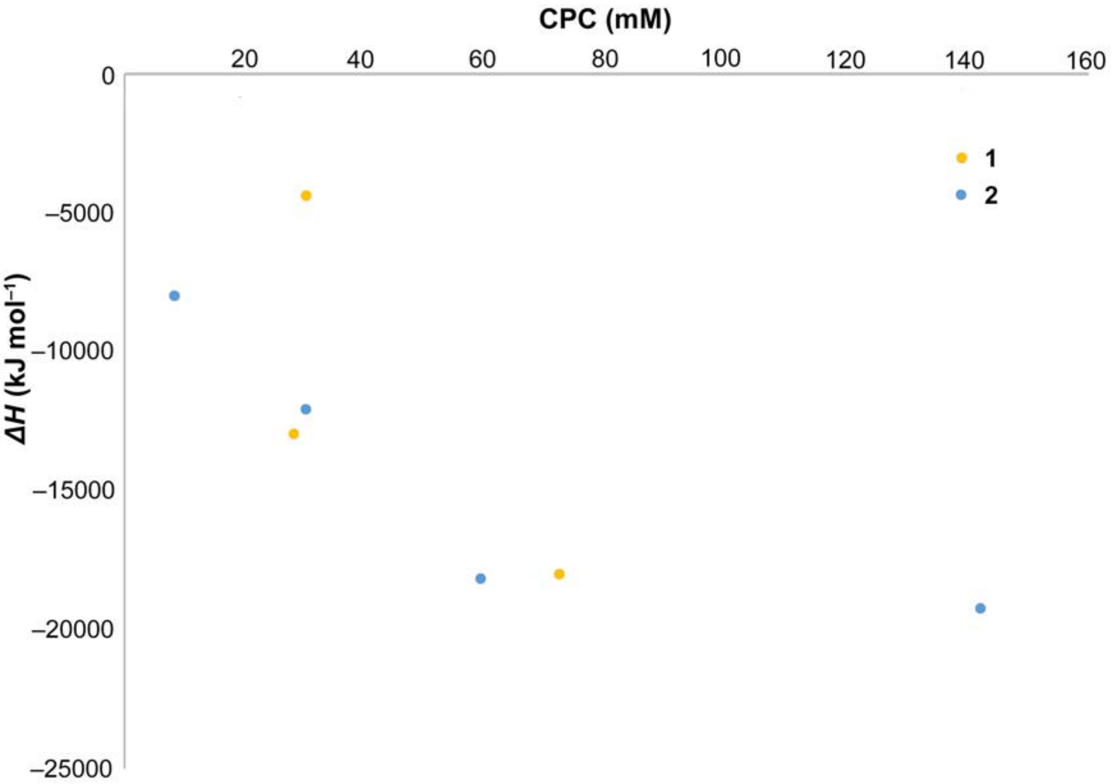

Figure S83. Plot of the CPC value for an anionic guest and the enthalpy of complexation.

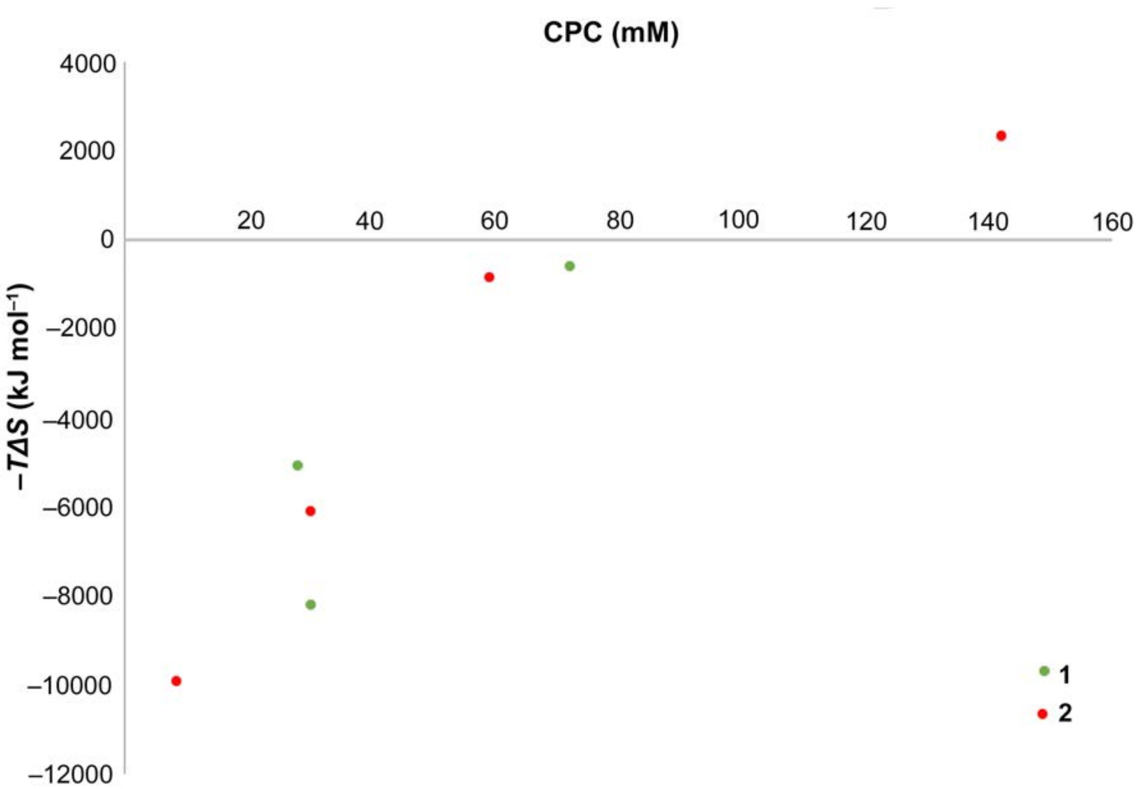

Figure S84. Plot of the CPC value for an anionic guest and the entropy of complexation.

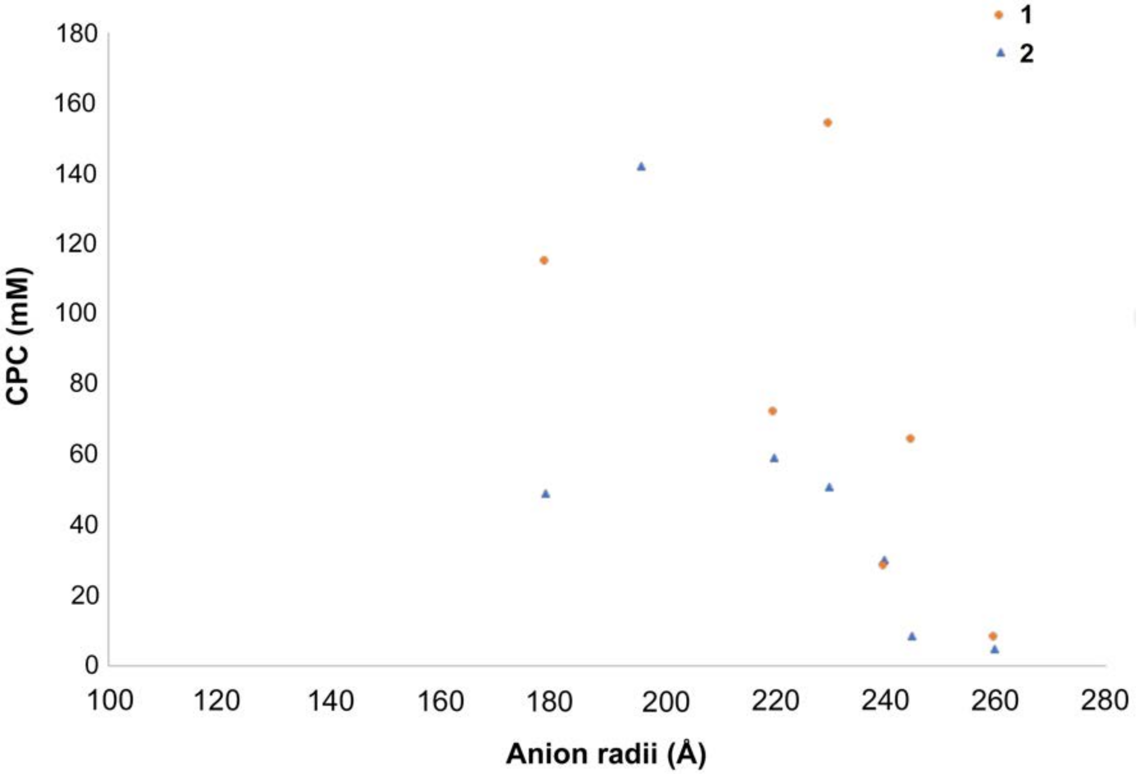

Figure S85. Plot of ionic radii of the anionic guests versus their CPC value.

Crystallographic analysis of complexes **1**.Cl⁻, **1**.Br⁻, **1**.ClO4⁻, and **2**.ClO_4^-.

a. General Crystallography Details

Suitable crystals of the complexes **1**.Cl⁻, **1**.Br⁻, **1**.ClO4⁻, and **2**.ClO_4^- were grown as follows: 1) **1**.Cl⁻ was crystallized by slow evaporation from 2 mL of a 5 mM solution in H_2O; 2) **1**.Br⁻ was crystallized by slow evaporation from 0.5 mL of a 1 mM solution **1**.Cl⁻ and 250 mM NaBr in H_2O; 3) **1**.ClO4⁻ was crystallized by slow evaporation from 0.5 mL of a 1 mM solution **1**.Cl⁻ with 100 mM $NaClO_4$ in H_2O, and; 4) **2**.ClO_4^- was crystallized by slow evaporation from 0.5 mL of a 1 mM solution **2**.Cl⁻ with 100 mM $NaClO_4$ in H_2O.

Suitable crystals of the compounds were mounted on polymer loops with a drop of heavy oil and placed in the cold nitrogen stream on the Bruker D8 QUEST diffractometer. Intensity data were collected under control of the APEX4 software[12] and converted to F^2 values by SAINT[12] which also performed global refinements of unit cell parameters. As the crystal of the complex between **1** and ClO_4^- was twinned, an empirical correction for absorption was applied with TWINABS[13] which also merged equivalent reflections. For the others, numerical absorption corrections and merging of equivalent reflections used SADABS.[14] The structures were solved by dual space methods (SHELXT[15]) and refined on F^2 by full-matrix, least-squares methods (SHELXL[16]). Some of the lattice water molecules and halide ions were disordered over two sites each and were refined by the split-atom model. In the perchlorate salts, disordered perchlorates were refined as rigid groups using two orientations for each disordered ion. There appeared to be more minor peaks in the difference map for **1** and Cl⁻ than for the others, indicating the presence of a significant number of disordered/partially occupied lattice water sites. The data were treated by PLATON SQUEEZE[17] which removed some but not all of the density which could not reasonably be modeled. Hydrogen atoms attached to carbon were included as riding contributions in idealized positions. Hydrogen atoms attached to nitrogen which could be located in difference maps were refined with DFIX 0.91 0.01 instructions. Those not located were included as riding contributions in idealized positions. Hydrogen atoms attached to oxygen were placed in locations derived from difference maps where possible. The remainder were placed in locations which would maintain idealized geometries for the water molecules, avoid short intermolecular H···H contacts and generate the best hydrogen bonding interactions. These hydrogen atoms were included as riding contributions. All hydrogen atoms were assigned isotropic displacement parameters tied to those of the attached atoms. Crystal and refinement details are presented in Table S4. All structures have been deposited in the Cambridge Crystallographic Data Center: **1**.Cl⁻ (CCDC # 2301765), **1**.Br⁻ (CCDC # 2301766), **1**.ClO_4^- (CCDC # 2301767) and **2**.ClO_4^- (CCDC # 2301995). Also given below are Responses to "Alerts", as well as representations of the four obtained structures (Figure S86 – Figure S101).

b. Crystal and refinement data

Table S4: Crystal and refinement data

	**Host 1.Cl⁻**	**Host 1.Br⁻**	**Host 1.ClO$_4$⁻**	**Host 2.ClO$_4$⁻**
Chemical formula	$C_{44}H_{72}Cl_4N_4O_{16}$	$C_{44}H_{69}Br_4N_4O_{14.50}$	$C_{44}H_{66}Cl_4N_4O_{29}$	$C_{48}H_{74}Cl_4N_{12}O_{29}$
Formula weight (g/mol)	1054.85	1205.67	1256.80 g/mol	1424.99
Temperature (K)	150(2)	150(2)	150(2)	170(2)
Wavelength (Å)	0.71073			
Crystal size (mm)	0.156 x 0.228 x 0.368	0.068 x 0.288 x 0.387	0.120 x 0.141 x 0.403	0.042x0.319x0.328
Crystal system	Triclinic	Triclinic	triclinic	triclinic
Space group	P-1	P-1	P-1	P-1
Unit cell dimensions (Å,°)				
a	13.4589(11)	13.4656(11)	11.9227(3)	13.6479(5)
b	14.7649(12)	15.1237(13)	12.7700(4)	15.7952(6)
c	15.3662(13)	15.4654(14)	19.0968(6)	15.8667(6)
α	114.213(2)	114.889(3)	96.4070(10)	113.645(2)
β	90.590(2)	90.595(3)	99.9080(10)	93.484(2)
γ	108.404(2)	108.490(3)	107.2860(10)	97.457(2)
Volume (Å^3)	2607.8(4)	2671.4(4)	2693.58(14)	3082.7(2)
Z	2	2	2	2
Density (calc. g/cm^3)	1.343	1.499	1.550	1.535
Absorp. Coeff. (mm^{-1})	0.296	3.077	0.318	0.291
F(000)	1120	1234	1316	1492
Diffractometer	Bruker D8 QUEST PHOTON 3 diffractometer			
Radiation source	fine-focus sealed tube (MoKα, λ = 0.71073 Å)			
θ range for data collection (°)	1.47 to 30.24°	2.00 to 26.69	1.10 to 33.22	1.90 to 30.69
Index ranges	-19≤h≤19, -20≤k≤20, -21≤l≤21	-16≤h≤16, -18≤k≤17, 0≤l≤19	-18≤h≤18, -19≤k≤19, -29≤l≤29	-19≤h≤19, -22≤k≤22, -22≤l≤22
Reflections collected	14563	19596	107208	205316
Independent reflections	14563	19596	20591 [R(int) = 0.0307]	19013 [R(int) = 0.0475]
Absorption Correction	Numerical mu Calculated (*SADAB*S, Kraus, et al., 2015)	Multi-scan (*TWINABS*, Sheldrick, 2009)	Numerical mu Calculated (*SADAB*S, Kraus, et al., 2015)	Numerical mu Calculated (*SADAB*S, Kraus, et al., 2015)
Max. & Min. Transmission	0.9550 and 0.8990	0.8180 and 0.3820	0.9630 and 0.8830	0.9880 and 0.9110
Structure Solution Techn.	Dual Space Methods			
Structure Solution Program	*SHELXT/5* (Sheldrick, 2015)			
Refinement method	Full-matrix least-squares on F^2			
Refinement program	*SHELXL-2019/1* (Lübben *et al*, 2019)			
Function minimized	Σ w(F_o^2 - F_c^2)^2			
Data/restraints/parameters	14563 / 30 / 642	19596 / 427 / 647	20591 / 12 / 732	19013 / 160 / 920
Goodness-of-fit on F^2	0.080	1.045	1.048	1.055
Δ/σ_{max}	0.001	0.001	0.001	0.001
Final R indices				
No. refl. I > 2σ(I)	11004; R1 = 0.0994, wR2 = 0.2733	15067; R1 = 0.0802, wR2 = 0.2250	17165; R1 = 0.0594, wR2 = 0.1486	13825: R1 = 0.0734, wR2 = 0.2159
All data	R1 = 0.1241, wR2 = 0.2896	R1 = 0.1038, wR2 = 0.2439	R1 = 0.0720, wR2 = 0.1561	R1 = 0.0969, wR2 = 0.2410
Weighting Scheme		w=1/[$\sigma^2(F_o^2)$+(0.1324P)^2+8.51 10P] where P=(F_o^2+2F_c^2)/3	w=1/[$\sigma^2(F_o^2)$+(0.0652P)^2+3.4964 P] where P=(F_o^2+2F_c^2)/3	w=1/[$\sigma^2(F_o^2)$+(0.1342P)^2+2.937 1P] where P=(F_o^2+2F_c^2)/3
Largest diff. peak and hole (eÅ^{-3})		2.041 and -1.857	1.849 and -1.498	1.115 and -1.080
R.M.S. deviation from mean (eÅ^{-3})		0.146	0.086	0.094

c. Responses to “Alerts”

Host 1.Cl⁻ (CCDC # 2301765)

_vrf_PLAT_097_ALERT_2_B

PROBLEM: Large Reported Max. (Positive) Residual Density 2.34 eA-3

RESPONSE: The structure features some disordered/partially occupied lattice water sites. Most were successfully modeled or removed with PLATON SQUEEZE[17] but this one, which is most likely another partially occupied water site, would not respond satisfactorily to either treatment.

_vrf_PLAT_420_ALERT_2_B

PROBLEM: D-H Bond Without Acceptor O10···H10A, O11···H11B, O16···H16C.

RESPONSE: These are some of the water hydrogens that couldn't be located in difference maps and so were positioned so as to retain idealized geometries for the respective water molecules while minimizing close contacts and providing for maximum hydrogen bonding interactions. For these, the first two restraints outweighed the third.

Host 1.Br⁻ (CCDC # 2301766)

_vrf_PLAT_417_ALERT_2_B

PROBLEM: Short Inter D-H..H-D H2C···H11B 2.06 Ang. x,y,z = 1_555

RESPONSE: This water molecule is held in place by 2 N---H···O hydrogen bonds from neighboring ammonium cations so it is not surprising that a short contact of this magnitude might occur.

_vrf_PLAT_420_ALERT_2_B

PROBLEM: D-H Bond Without Acceptor O9 --H9B, O15 --H15B.

RESPONSE: H9B is pointing directly towards one phenyl ring with an H···centroid distance of 2.62 \%A so is involved in a C---H···\p(ring) interaction. The water molecule based on O15 is disordered over two sites obviating the possibility for this hydrogen to have a close acceptor.

Host 1.ClO4⁻ (CCDC # 2301767)

_vrf_PLAT_097_ALERT_2_B

PROBLEM: Large Reported Max. (Positive) Residual Density 1.85 eA-3

RESPONSE: This is in the vicinity of one disordered perchlorate and is likely the result of additional disorder that was not modeled.

_vrf_PLAT_417_ALERT_2_B

PROBLEM: Short Inter D-H..H-D H4B···H28A 1.89 Ang. (1_555).

RESPONSE: H28A could not be definitely located in a difference map and so was positioned to maximize hydrogen bonding while maintaining idealized geometry for that water molecule. The nearby presence of a disordered water molecule may have affected this process but it could not be improved upon.

PROBLEM: D-H Without Acceptor O28 --H28B

RESPONSE: This is likely related to the previous problem.

Host 2.ClO4⁻ (CCDC # 2301995)

_vrf_PLAT_420_ALERT_2_B

PROBLEM: D-H Bond Without Acceptor O29···H29A, O29···H29B

RESPONSE: These are some of the water hydrogens that couldn't be located in difference maps and were positioned so as to retain idealized geometries for the respective water molecules while minimizing close contacts and providing for maximum hydrogen bonding interactions. For these, the first two restraints outweighed the third.

d. Packing Diagrams for Structures **1**.Cl⁻, **1**.Br⁻, **1**.ClO₄⁻ and **2**.ClO₄⁻

Shown below (Figure S86 – Figure S101) are the packing diagrams for the X-ray crystal structures of complexes **1**.Cl^-, **1**.Br^-, **1**.ClO_4^- and **2**.ClO_4^-.

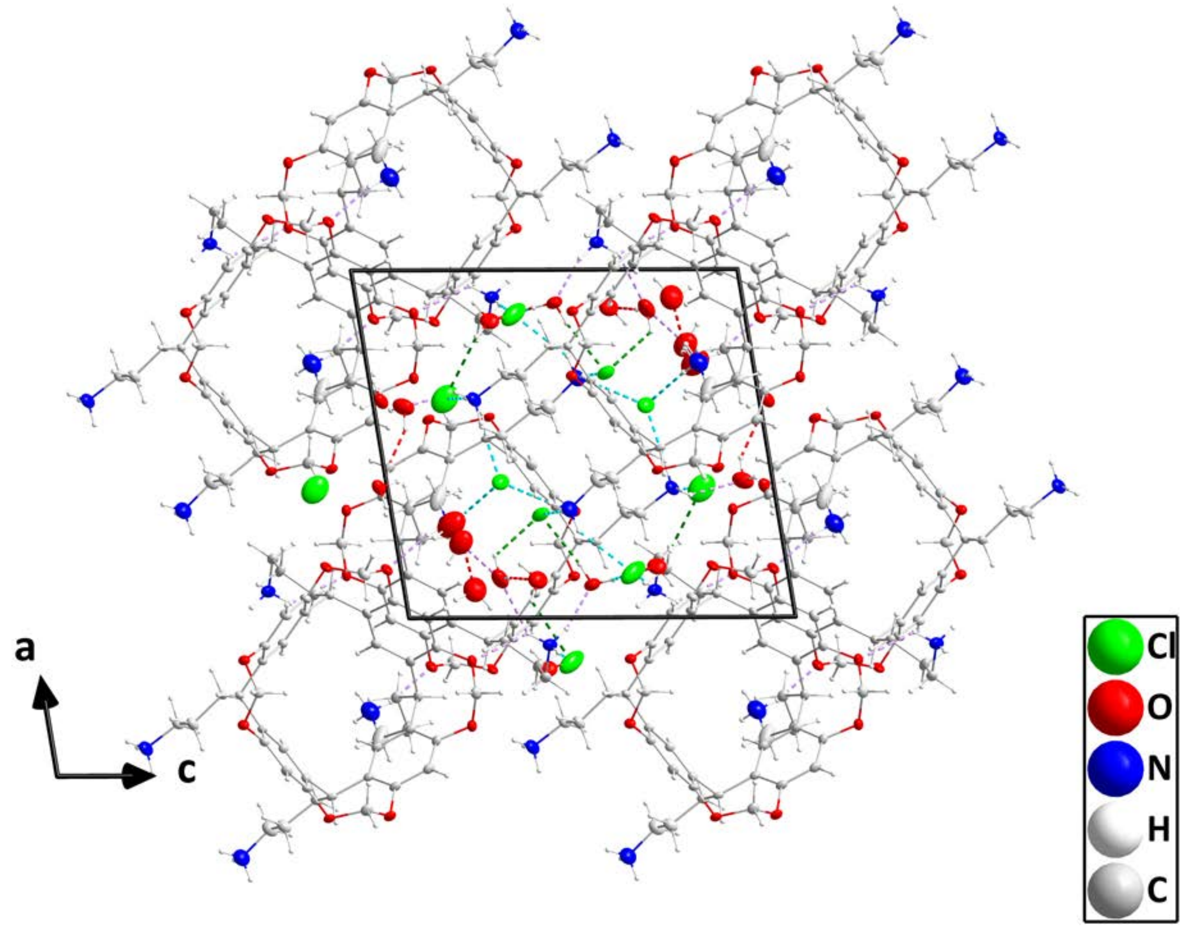

Figure S86. Packing for the complex between **1** and Cl^-, looking along the B axis of the unit cell. In this view, the O—H···O, N—H···O, O—H···Cl and N—H···Cl hydrogen bonds are depicted, respectively, by red, violet, green and light blue dashed lines. Only the major components of the disorder are shown, and ellipsoids are drawn at the 50% probability level.

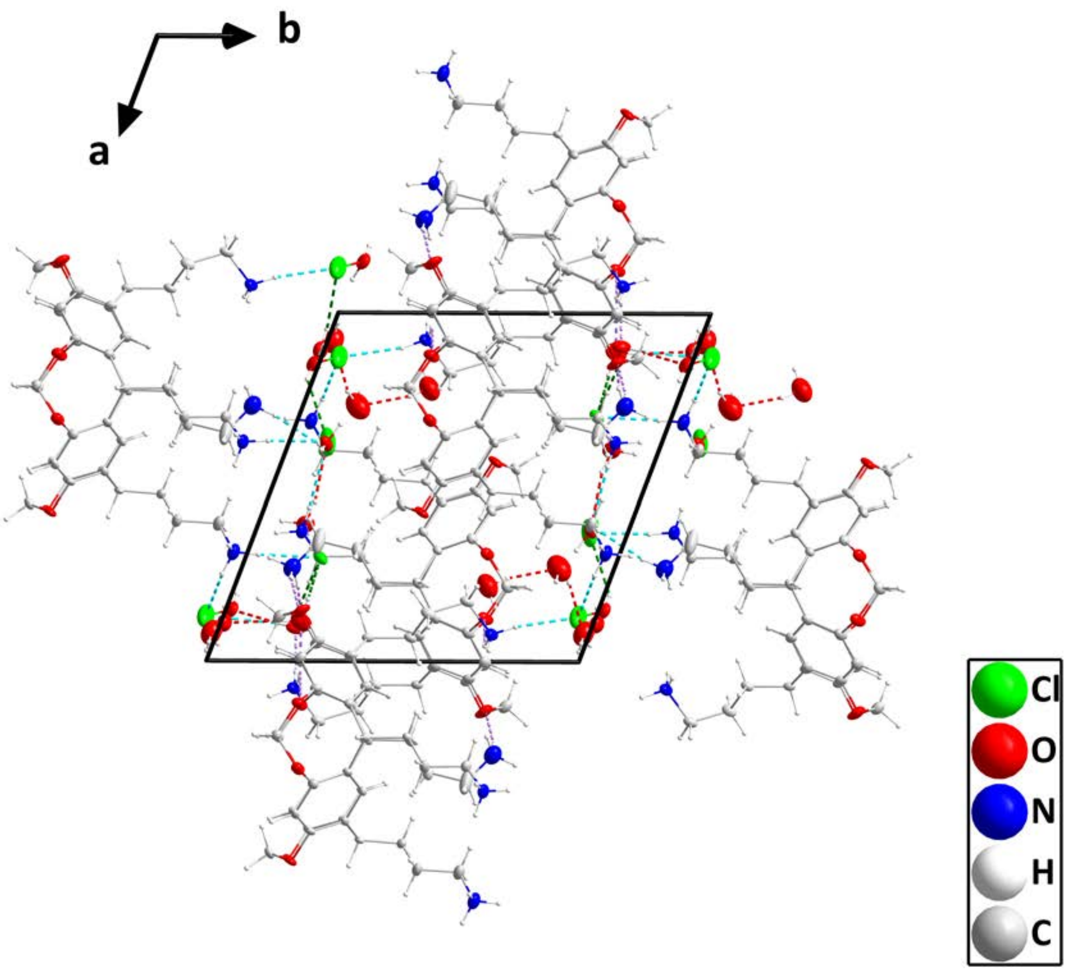

Figure S87. Packing for the complex between **1** and Cl⁻, looking along the C axis of the unit cell. In this view, the O—H···O, N—H···O, O—H···Cl and N—H···Cl hydrogen bonds are depicted, respectively, by red, violet, green and light blue dashed lines. Only the major components of the disorder are shown, and ellipsoids are drawn at the 50% probability level.

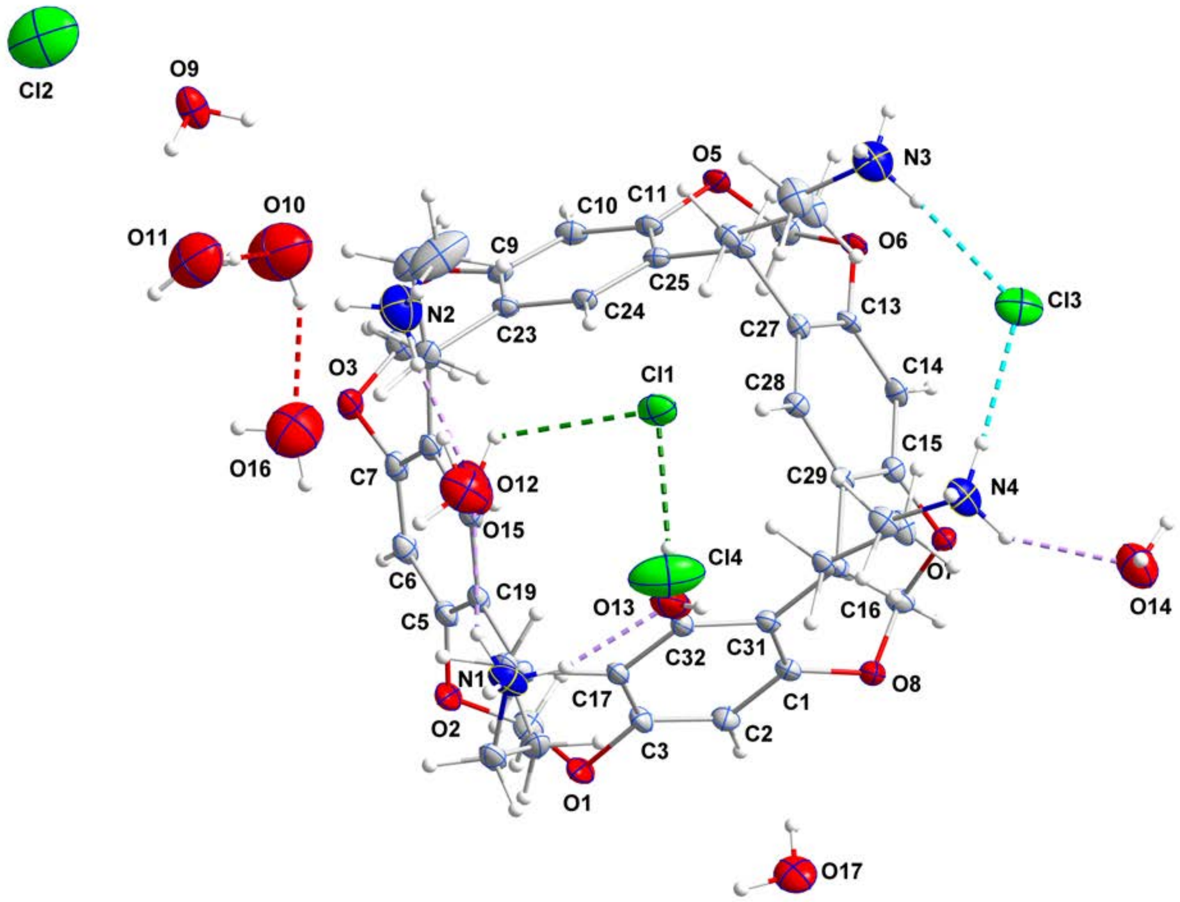

Figure S88. Unit cell representation of the central binding site looking down into the pocket of the complex between **1** and Cl⁻ with the cavitand bowl at rear. In this view, the O—H···O, N—H···O, O—H···Cl and N—H···Cl hydrogen bonds are depicted, respectively, by red, violet, green and light blue dashed lines. Only the major components of the disorder are shown, and ellipsoids are drawn at the 50% probability level.

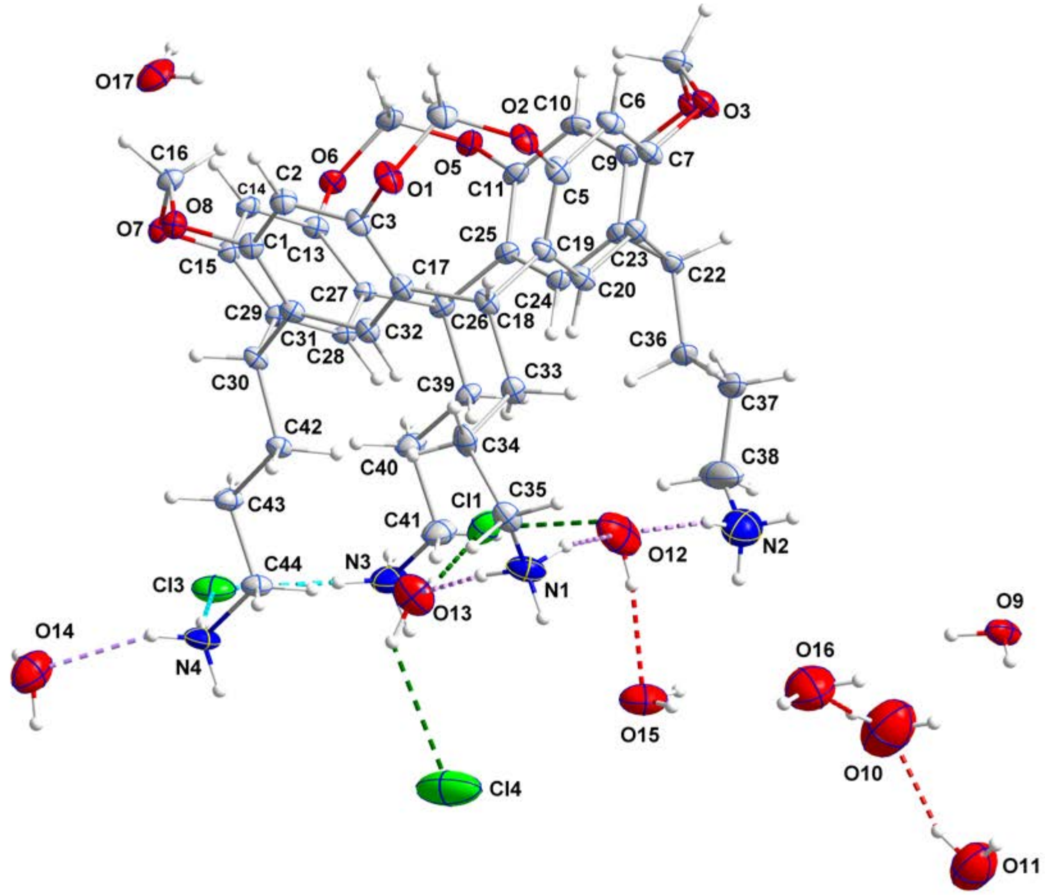

Figure S89. Unit cell representation of the central binding site looking from the side of the host-guest complex between **1** and Cl^{-}. In this view, the O—H···O, N—H···O, O—H···Cl and N—H···Cl hydrogen bonds are depicted, respectively, by red, violet, green and light blue dashed lines. Only the major components of the disorder are shown, and ellipsoids are drawn at the 50% probability level.

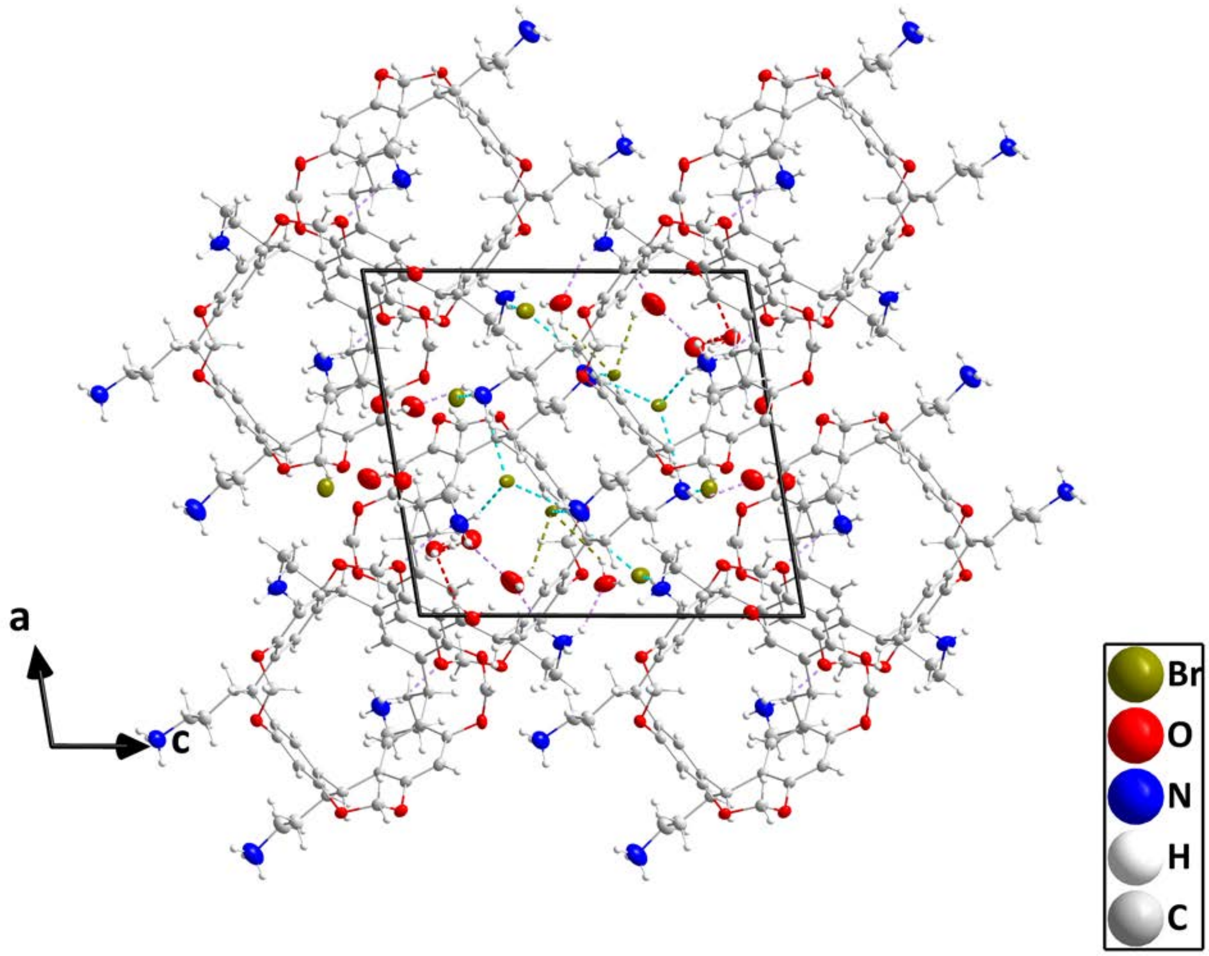

Figure S90. Packing for the complex between **1** and Br⁻, looking along the B axis of the unit cell. In this view, the O—H···O, N—H···O, O—H···Br and N—H···Br hydrogen bonds are depicted, respectively, by red, violet, olive green and light blue dashed lines. Only the major components of the disorder are shown, and ellipsoids are drawn at the 50% probability level.

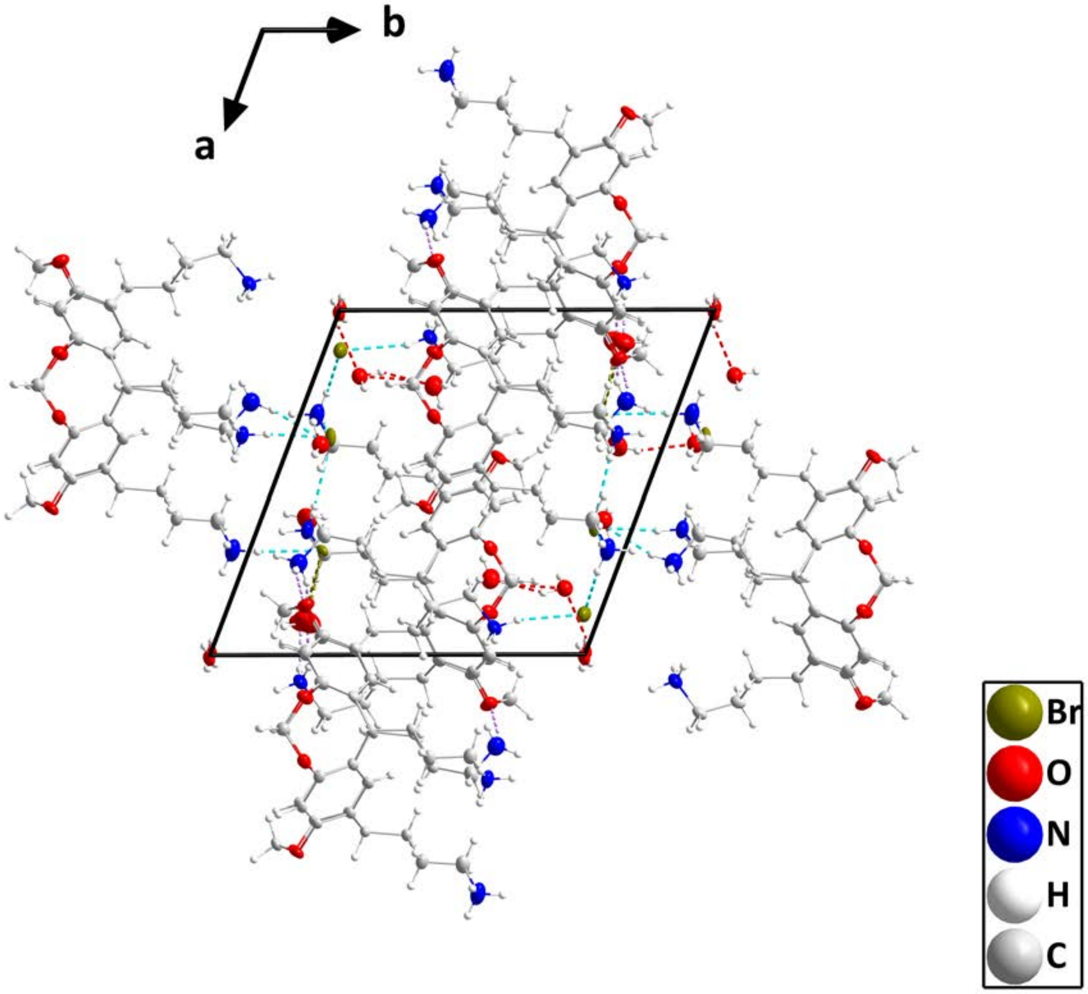

Figure S91. Packing for the complex between **1** and Br⁻, looking along the C axis of the unit cell. In this view, the O—H···O, N—H···O, O—H···Br and N—H···Br hydrogen bonds are depicted, respectively, by red, violet, olive green and light blue dashed lines. Only the major components of the disorder are shown, and ellipsoids are drawn at the 50% probability level.

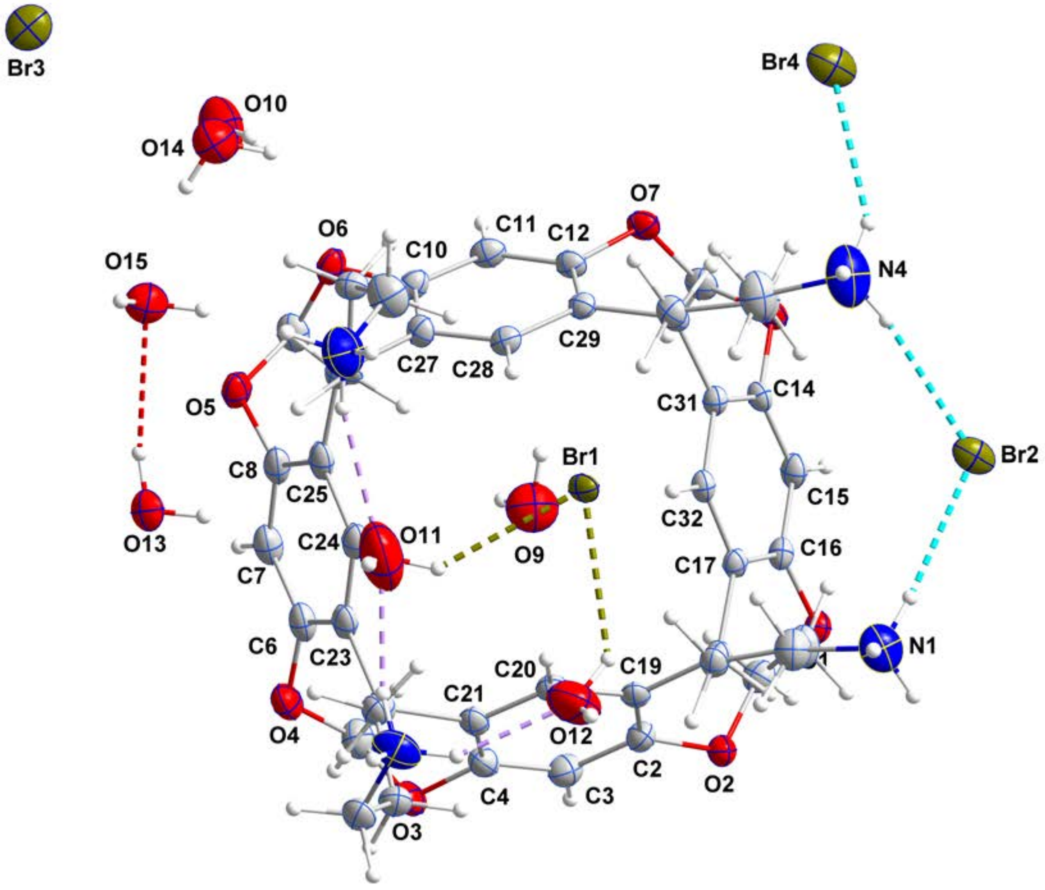

Figure S92. Unit cell representation of the central binding site looking down into the pocket of the complex between **1** and Br^- with the cavitand bowl at rear. In this view, the O—H···O, N—H···O, O—H···Br and N—H···Br hydrogen bonds are depicted, respectively, by red, violet, olive green and light blue dashed lines. Only the major components of the disorder are shown, and ellipsoids are drawn at the 50% probability level.

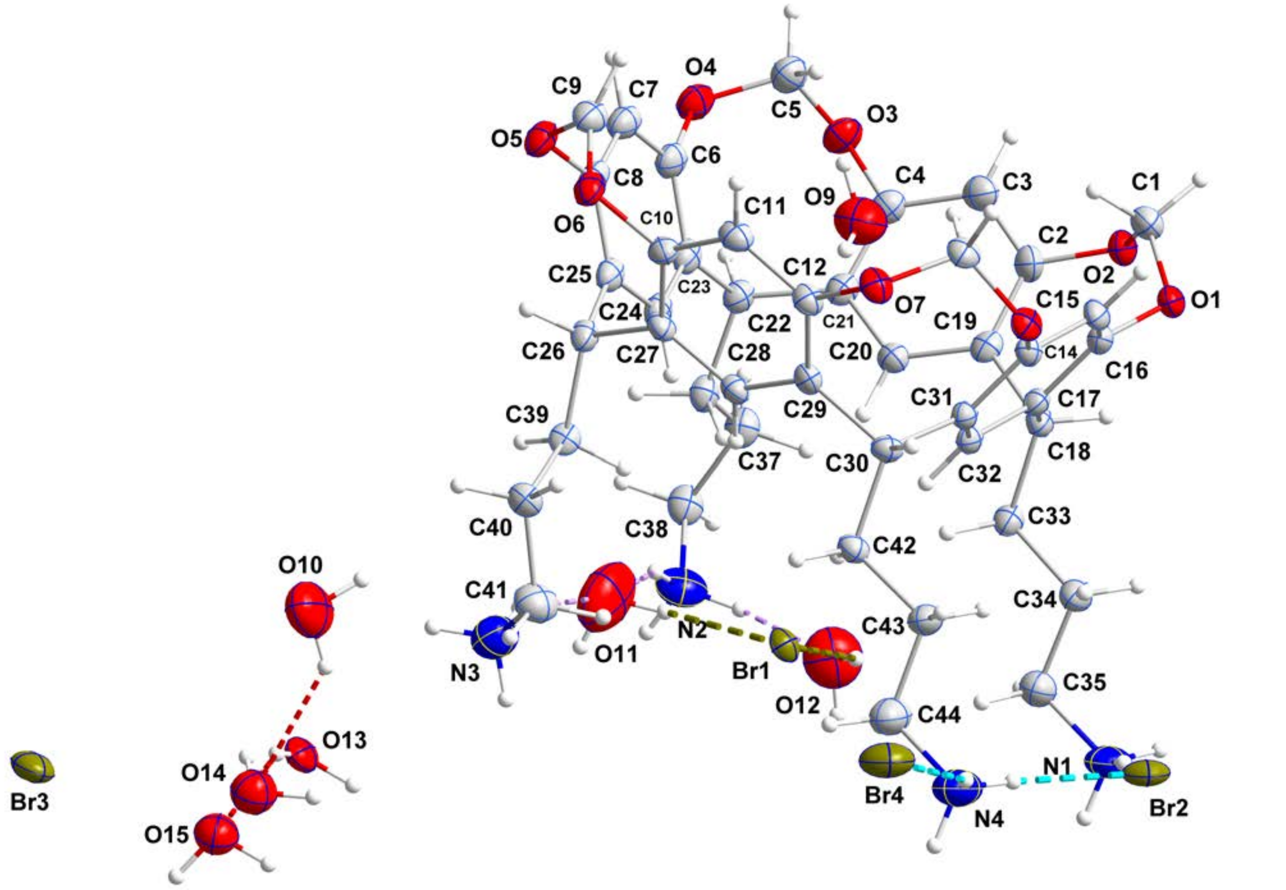

Figure S93. Unit cell representation of the central binding site looking from the side of the host-guest complex between **1** and Br⁻. In this view, the O—H···O, N—H···O, O—H···Br and N—H···Br hydrogen bonds are depicted, respectively, by red, violet, olive green and light blue dashed lines. Only the major components of the disorder are shown, and ellipsoids are drawn at the 50% probability level.

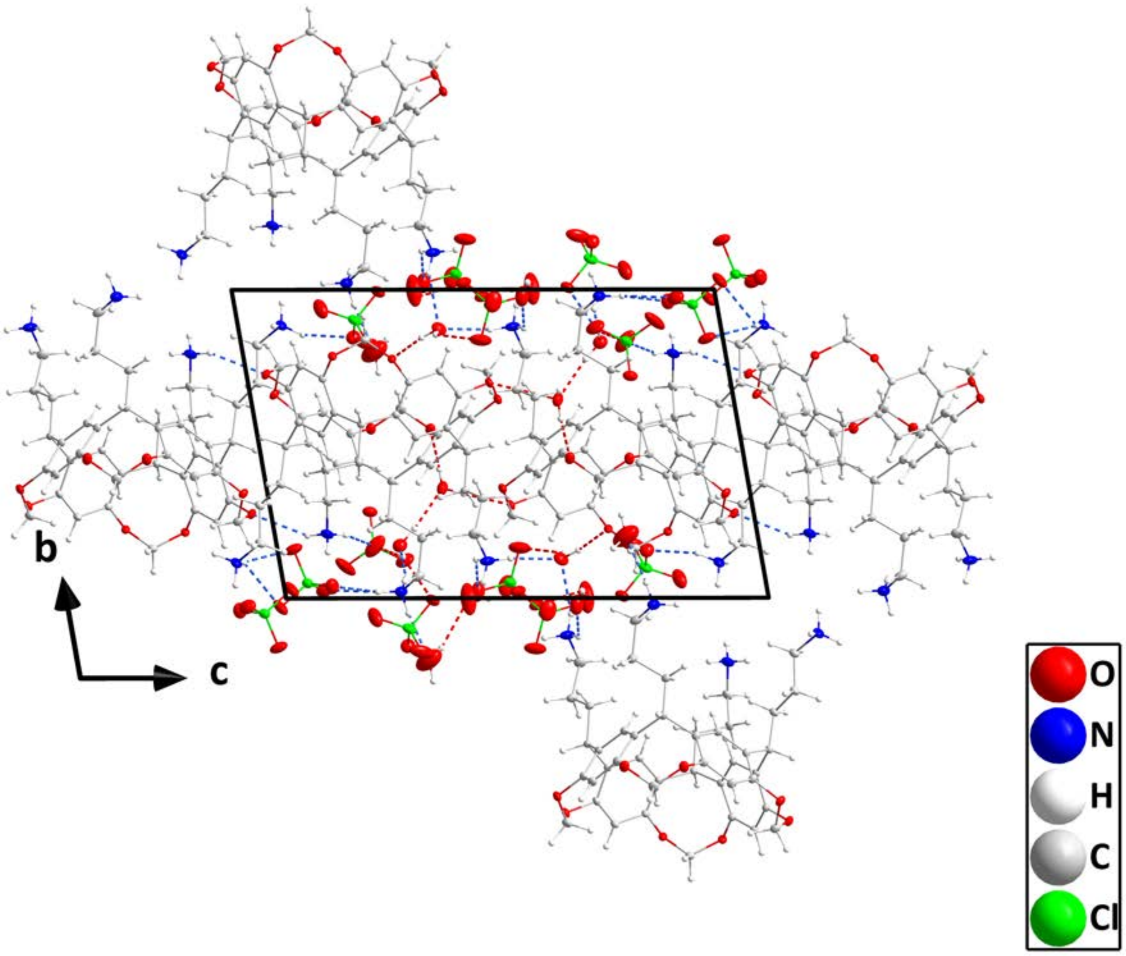

Figure S94. Packing for the complex between **1** and ClO_4^-, looking along the A axis of the unit cell. In this view, the O—H···O and N—H···O hydrogen bonds are depicted, respectively, by red and blue dashed lines. Only the major components of the disorder are shown, and ellipsoids are drawn at the 50% probability level.

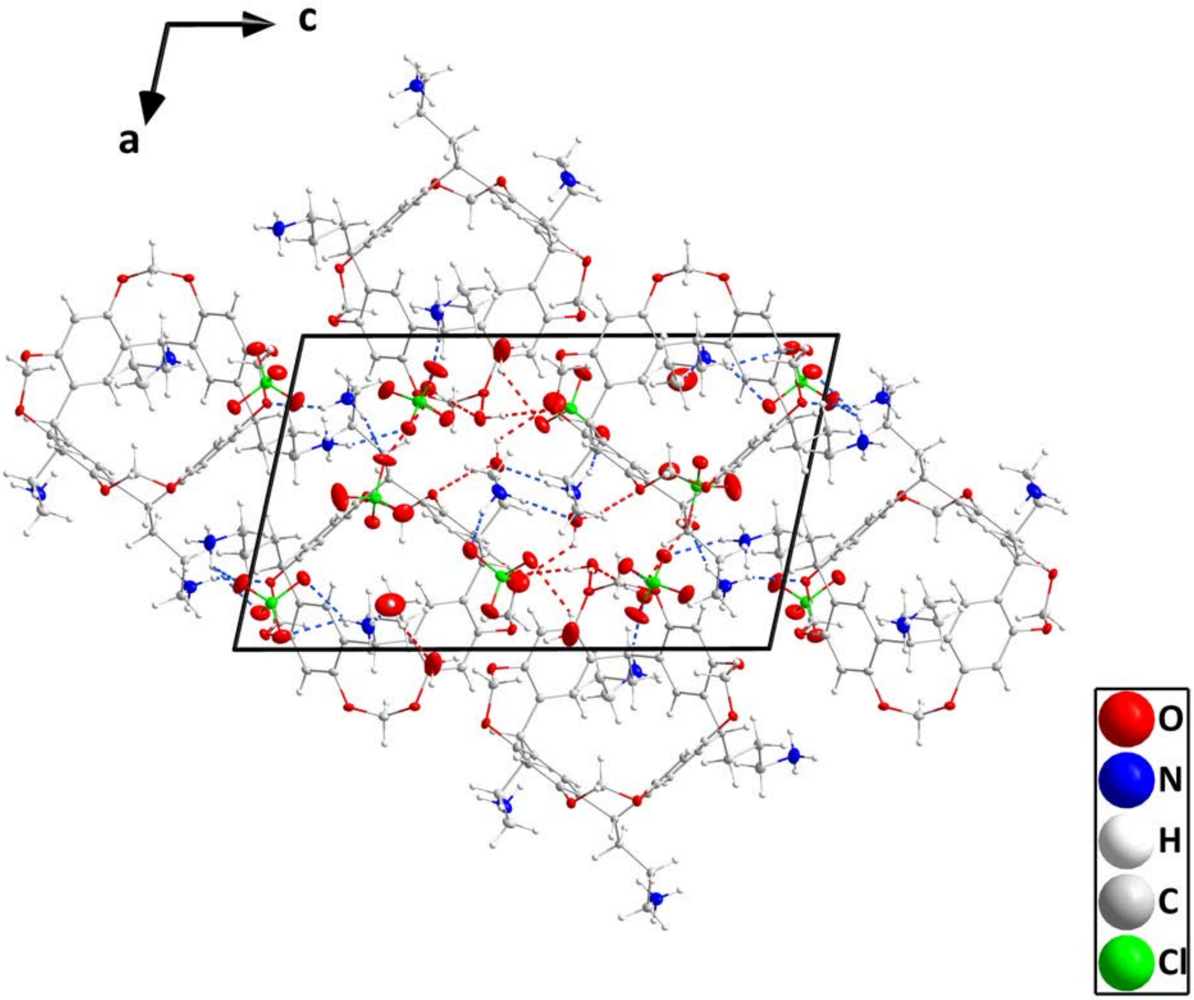

Figure S95. Packing for the complex between **1** and ClO_4^-, looking along the B axis of the unit cell. In this view, the O—H···O, N—H···O hydrogen bonds are depicted, respectively, by red and blue dashed lines. Only the major components of the disorder are shown, and ellipsoids are drawn at the 50% probability level.

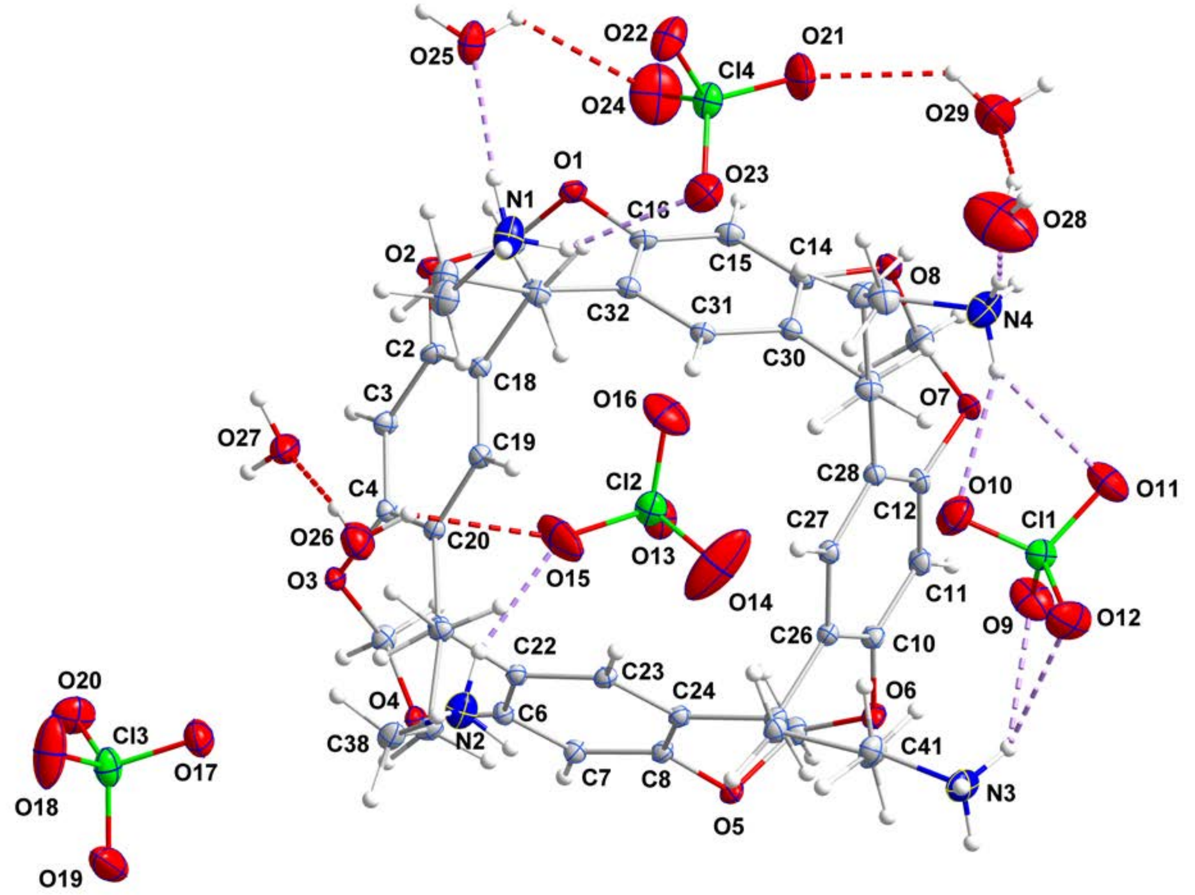

Figure S96. Unit cell representation of the central binding site looking down into the pocket of the complex between **1** and ClO_4^- with the cavitand bowl at rear. In this view, the O—H···O and N—H···O hydrogen bonds are depicted, respectively, by red and violet dashed lines. Only the major components of the disorder are shown, and ellipsoids are drawn at the 50% probability level.

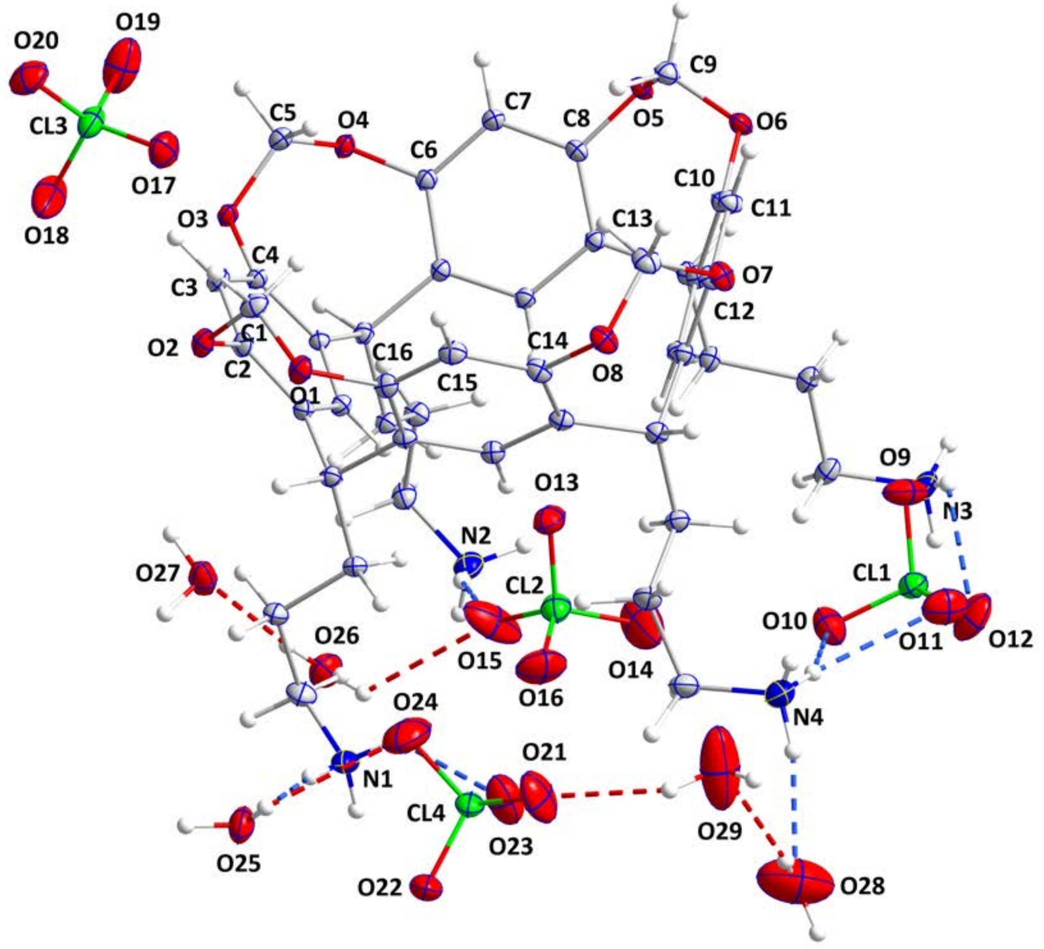

Figure S97. Unit cell representation of the central binding site looking from the side of the host-guest complex between **1** and ClO_4^-. In this view, the O—H···O and N—H···O hydrogen bonds are depicted, respectively, by red and blue dashed lines. Only the major components of the disorder are shown, and ellipsoids are drawn at the 50% probability level.

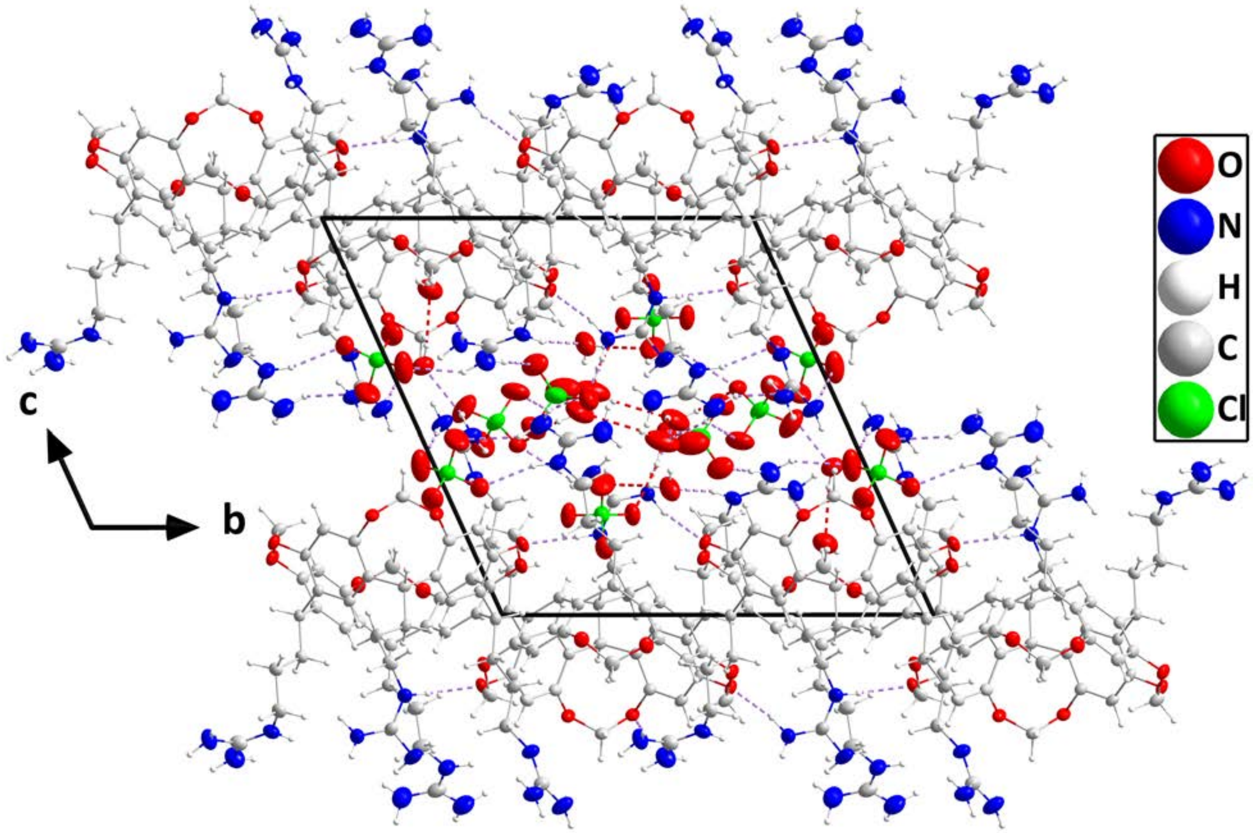

Figure S98. Packing for the complex between **2** and ClO_4^-, looking along the A axis of the unit cell. In this view, the O—H···O and N—H···O hydrogen bonds are depicted, respectively, by red and violet dashed lines. Only the major components of the disorder are shown, and ellipsoids are drawn at the 50% probability level.

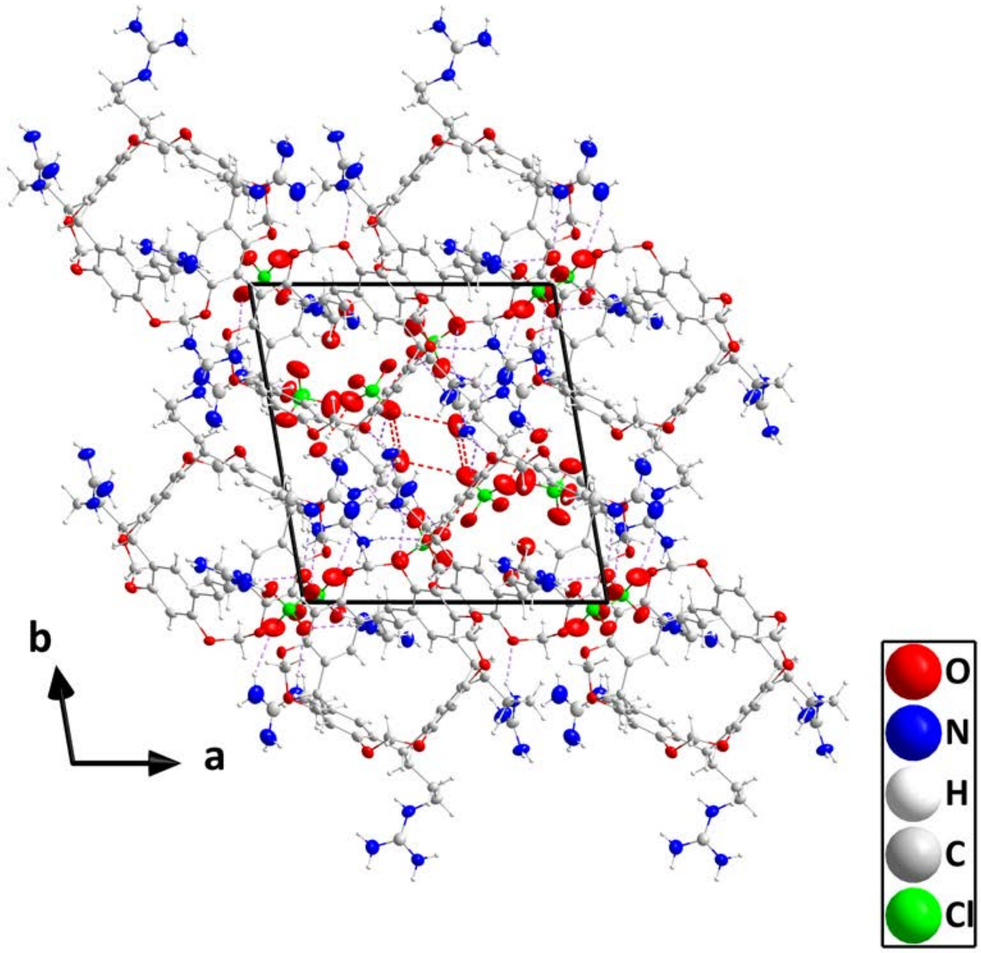

Figure S99. Packing for the complex between **2** and ClO_4^-, looking along the C axis of the unit cell. In this view, the O—H···O and N—H···O hydrogen bonds are depicted, respectively, by red and violet dashed lines. Only the major components of the disorder are shown, and ellipsoids are drawn at the 50% probability level.

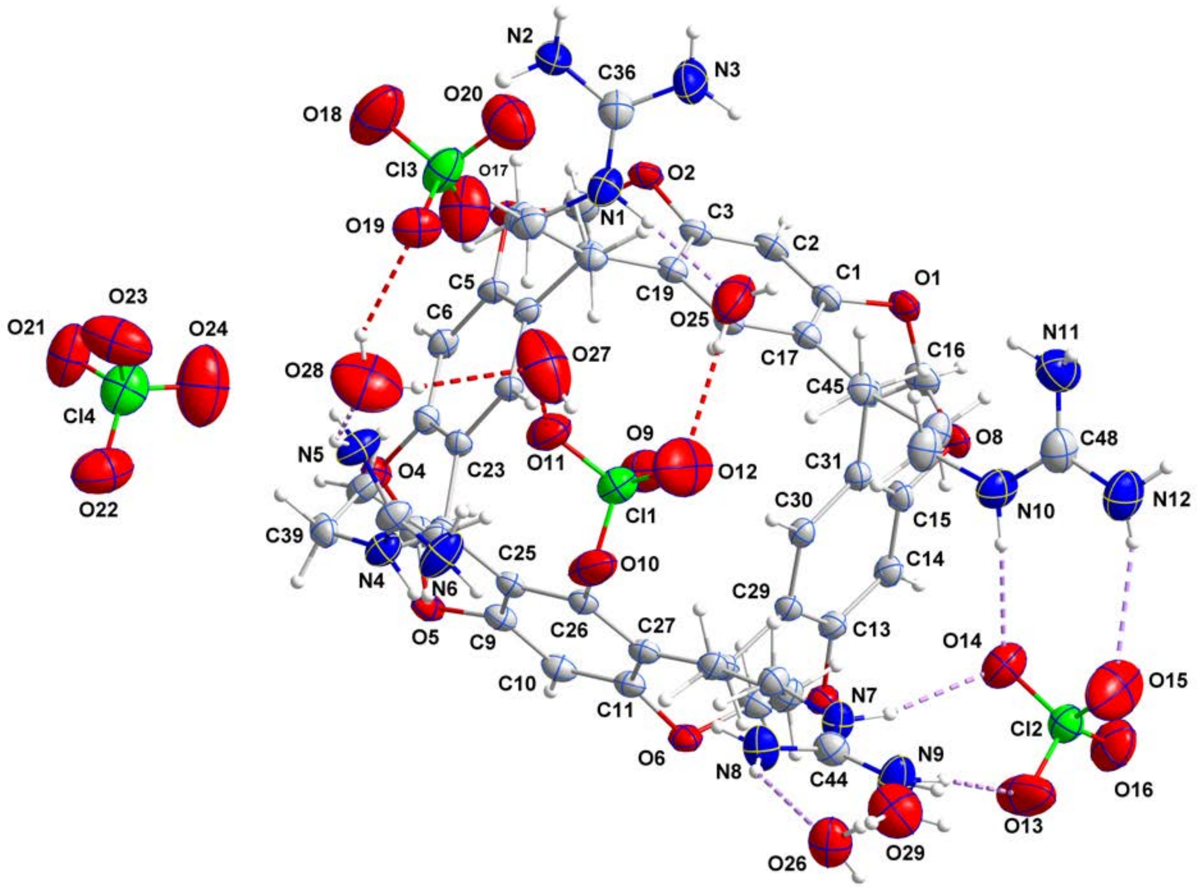

Figure S100. Unit cell representation of the central binding site looking down into the pocket of the complex between **2** and ClO_4^- with the cavitand bowl at rear. In this view, the O—H···O and N—H···O hydrogen bonds are depicted, respectively, by red and violet dashed lines. Only the major components of the disorder are shown, and ellipsoids are drawn at the 50% probability level.

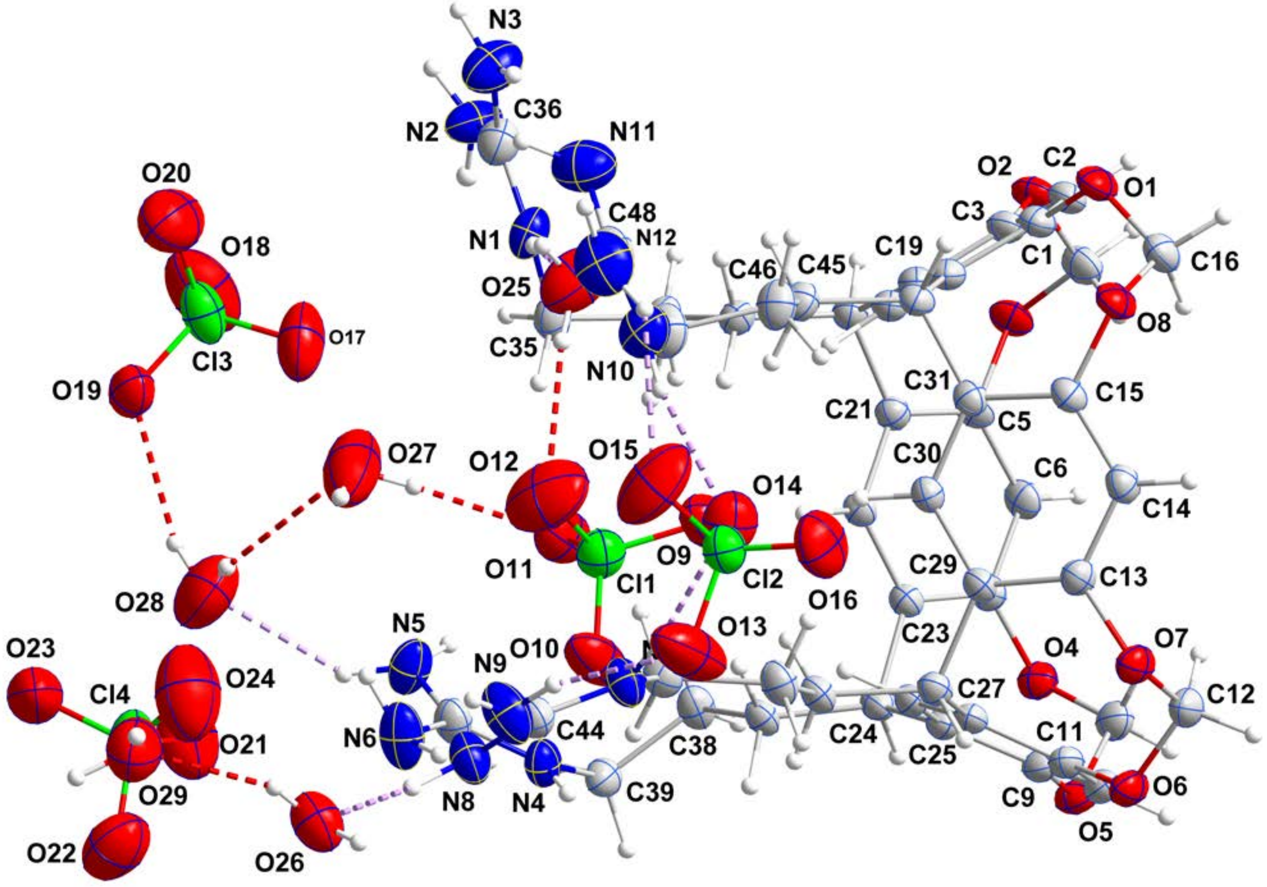

Figure S101. Unit cell representation of the central binding site looking from the side of the host-guest complex between **2** and ClO_4^-. In this view, the O—H···O and N—H···O hydrogen bonds are depicted, respectively, by red and violet dashed lines. Only the major components of the disorder are shown, and ellipsoids are drawn at the 50% probability level.

5. Molecular dynamics simulations and spatial distribution functions (SDFs)

A sequence of molecular dynamics simulations was executed employing the GROMACS 2016.3[18, 19] software package to analyze the behavior of hosts **1** and **2**. These simulations were conducted within a bulk water environment at a temperature of 25 °C and a pressure of 1 bar.

Host modelling

Each host was modelled with two starting conformations in vacuum: no counterion on the C4 axis (ammonium/guanidium groups face outside), and a Cl^{-} on the C4 axis and on the plane of the four terminal functional groups (ammonium/guanidium groups face inside). Each starting conformation was minimized with B3LYP/6-31G(d,p) method, resulting in four different host conformation combinations: **1** Confo. I, **1** Confo. II, **2** Confo. I and **2** Confo. II. Host **1** with outward feet, host **1** with inward feet, host **2** with outward feet, and host **2** with inward feet. Their Cartesian coordinates were given below.

Table S5. Cartesian coordinates of **1** Confo. I

Atom	x	y	z
H	3.573	2.790	-3.096
C	3.150	2.456	-2.155
C	2.117	1.643	0.299
C	3.629	1.302	-1.543
C	2.140	3.192	-1.542
C	1.606	2.805	-0.296
C	3.128	0.874	-0.299
H	1.695	1.314	1.247
C	3.657	-0.456	0.244
H	4.617	-0.596	-0.259
C	0.456	3.657	0.244
H	0.597	4.617	-0.259
C	2.805	-1.606	-0.296
C	1.302	-3.629	-1.543
C	3.192	-2.140	-1.542
C	1.643	-2.117	0.299
C	0.874	-3.128	-0.299
C	2.456	-3.150	-2.155
H	1.314	-1.695	1.247
H	2.790	-3.572	-3.096
C	-0.874	3.128	-0.299
C	-3.192	2.140	-1.542
C	-1.643	2.117	0.299
C	-1.302	3.629	-1.543
C	-2.456	3.150	-2.155
C	-2.805	1.606	-0.296
H	-1.314	1.695	1.247
H	-2.790	3.572	-3.096
C	-3.657	0.456	0.244
H	-4.617	0.597	-0.259
C	-0.456	-3.657	0.244
H	-0.597	-4.617	-0.259
C	-1.606	-2.805	-0.296
C	-3.629	-1.302	-1.543
C	-2.140	-3.192	-1.542
C	-2.117	-1.643	0.299
C	-3.128	-0.874	-0.299
C	-3.150	-2.456	-2.155
H	-1.695	-1.314	1.247
H	-3.572	-2.790	-3.096
C	0.479	3.994	1.755
H	0.020	3.212	2.370
C	3.994	-0.479	1.755
H	3.212	-0.019	2.370
O	-4.662	-0.622	-2.159
O	-4.361	1.733	-2.155
O	-1.733	-4.361	-2.155
O	4.662	0.623	-2.159
O	4.361	-1.733	-2.155
O	0.623	-4.662	-2.159
C	4.357	-0.537	-2.922
H	3.395	-0.414	-3.434
C	-0.537	-4.357	-2.922
H	-0.414	-3.395	-3.434
O	1.733	4.361	-2.155
C	0.537	4.357	-2.922
H	0.415	3.395	-3.434
O	-0.622	4.662	-2.159
C	-4.356	0.537	-2.923
H	-3.395	0.415	-3.434
H	4.085	-1.522	2.078
C	-0.479	-3.994	1.755
C	-3.994	0.479	1.754
H	1.522	4.086	2.077
H	-3.212	0.019	2.370
H	-4.085	1.522	2.078
H	-1.522	-4.085	2.078
H	-0.019	-3.212	2.370
C	0.241	-5.345	1.978
H	-0.205	-6.079	1.294
H	1.296	-5.248	1.694
C	-0.242	5.345	1.979
H	0.203	6.079	1.294
H	-1.297	5.246	1.695
C	-5.345	-0.241	1.978
H	-6.079	0.205	1.294
C	5.346	0.241	1.978
H	6.079	-0.206	1.295

H	-5.248	-1.296	1.694
H	5.248	1.296	1.694
C	5.881	0.131	3.403
H	5.296	0.697	4.131
H	5.955	-0.906	3.740
C	-0.130	5.881	3.403
H	0.907	5.956	3.738
H	-0.695	5.296	4.132
C	-5.881	-0.131	3.403
H	-5.955	0.906	3.739
H	-5.296	-0.696	4.131
C	0.130	-5.881	3.403
H	-0.907	-5.955	3.739
H	0.696	-5.296	4.132
N	7.305	0.690	3.481
N	-7.305	-0.689	3.481
N	0.689	-7.305	3.481
H	-5.177	0.643	-3.631
H	0.643	5.177	-3.630
H	-0.643	-5.177	-3.630
H	5.177	-0.643	-3.630
N	-0.690	7.304	3.481
H	-7.939	-0.174	2.858
H	-7.340	-1.675	3.194
H	-7.693	-0.630	4.430
H	0.174	-7.939	2.858
H	0.629	-7.693	4.430
H	1.674	-7.340	3.195
H	7.693	0.630	4.430
H	7.939	0.175	2.858
H	7.340	1.675	3.194
H	-1.676	7.339	3.196
H	-0.629	7.693	4.430
H	-0.176	7.939	2.858

Table S6. Cartesian coordinates of **1** Confo. II

Atom	x	y	z
H	3.533	-3.346	-3.156
C	2.602	-2.944	-2.773
C	0.195	-1.941	-1.816
C	1.998	-1.860	-3.404
C	1.998	-3.521	-1.660
C	0.768	-3.044	-1.163
C	0.770	-1.343	-2.945
H	-0.709	-1.498	-1.407
C	0.218	-0.126	-3.686
H	0.727	-0.173	-4.651
C	0.257	-3.690	0.124
H	0.778	-4.649	0.160
C	0.762	1.151	-3.048
C	2.052	3.366	-1.878
C	1.997	1.621	-3.539
C	0.214	1.817	-1.940
C	0.819	2.934	-1.349
C	2.630	2.721	-2.968
H	-0.693	1.428	-1.486
H	3.565	3.084	-3.380
C	0.825	-2.943	1.330
C	2.086	-1.642	3.480
C	0.250	-1.819	1.939
C	2.068	-3.387	1.820
C	2.688	-2.749	2.890
C	0.841	-1.159	3.029
H	-0.667	-1.420	1.515
H	3.632	-3.123	3.273
C	0.333	0.124	3.684
H	0.872	0.165	4.632
C	0.296	3.688	-0.128
H	0.827	4.641	-0.179
C	0.836	3.037	1.144
C	2.122	1.838	3.346
C	2.086	3.500	1.602
C	0.271	1.940	1.815
C	0.874	1.336	2.926
C	2.718	2.915	2.696
H	-0.650	1.507	1.434
H	3.665	3.307	3.050
C	-1.226	-4.148	0.206
H	-1.289	-5.091	-0.350
C	-1.272	-0.181	-4.127
H	-1.337	0.381	-5.066
O	2.756	1.322	4.457
O	2.719	-1.056	4.557
O	2.697	4.583	1.002
O	2.602	-1.351	-4.535
O	2.589	1.027	-4.635
O	2.687	4.483	-1.376
C	3.360	-0.152	-4.439
H	3.883	-0.108	-3.477
C	3.452	4.370	-0.183
H	3.955	3.397	-0.145
O	2.615	-4.611	-1.079
C	3.406	-4.407	0.084
H	3.918	-3.439	0.031
O	2.674	-4.513	1.298
C	3.496	0.114	4.337
H	3.988	0.065	3.359
H	-1.485	-1.226	-4.384
C	-1.183	4.164	-0.168
C	-1.140	0.195	4.174
H	-1.419	-4.402	1.256
H	-1.182	-0.373	5.111
H	-1.331	1.241	4.445
H	-1.397	4.436	-1.210
H	-1.223	5.100	0.403
C	-2.328	3.284	0.366
H	-2.361	2.303	-0.124
H	-2.178	3.112	1.439
C	-2.373	-3.264	-0.318
H	-2.391	-2.278	0.163
H	-2.238	-3.103	-1.394
C	-2.306	-0.321	3.308
H	-2.346	0.171	2.329

C	-2.400	0.356	-3.226
H	-2.417	-0.133	-2.245
H	-2.174	-1.395	3.133
H	-2.246	1.429	-3.059
C	-3.736	0.136	-3.931
H	-3.735	0.537	-4.947
H	-4.013	-0.919	-3.984
C	-3.699	-3.977	-0.072
H	-3.957	-4.027	0.989
H	-3.699	-4.995	-0.468
C	-3.614	-0.083	4.056
H	-3.870	0.977	4.126
H	-3.588	-0.491	5.069
C	-3.652	4.010	0.146
H	-3.926	4.071	-0.910
H	-3.636	5.026	0.549
N	-4.881	0.840	-3.201
N	-4.793	-0.760	3.358
N	-4.806	3.292	0.845
H	4.219	0.139	5.150
H	4.112	-5.235	0.102
H	4.167	5.190	-0.223
H	4.056	-0.184	-5.275
N	-4.858	-3.253	-0.757
H	-4.680	-1.777	3.395
H	-5.685	-0.543	3.817
H	-4.860	-0.486	2.355
H	-4.837	2.281	0.592
H	-5.713	3.715	0.620
H	-4.690	3.357	1.860
H	-5.789	0.634	-3.632
H	-4.921	0.569	-2.195
H	-4.751	1.855	-3.248
H	-4.754	-3.316	-1.774
H	-5.763	-3.674	-0.522
H	-4.882	-2.244	-0.498

Table S7. Cartesian coordinates of **2** Confo. I

Atom	x	y	z
C	0.124	-4.854	-1.987
C	-0.085	-2.075	-1.936
C	1.242	-4.065	-2.224
C	-1.093	-4.250	-1.702
C	-1.234	-2.845	-1.675
C	1.166	-2.655	-2.220
C	2.509	-1.939	-2.393
C	-2.626	-2.357	-1.256
C	3.165	-1.692	-1.031
C	4.383	-1.630	1.505
C	4.008	-2.736	-0.589
C	2.950	-0.618	-0.147
C	3.548	-0.555	1.125
C	4.620	-2.701	0.656
C	-2.711	-2.185	0.265
C	-2.738	-2.249	3.079
C	-2.312	-1.067	1.025
C	-3.124	-3.335	0.973
C	-3.150	-3.364	2.361
C	-2.323	-1.063	2.432
C	-1.812	0.038	3.369
C	3.324	0.502	2.213
C	2.099	0.142	3.061
C	0.055	-0.910	4.686
C	2.386	-0.679	4.175
C	0.744	0.417	2.796
C	-0.302	-0.091	3.591
C	1.381	-1.187	4.986
C	-3.421	-1.392	-2.178
C	2.737	-0.941	-3.555
O	-0.902	-1.416	5.548
O	-2.803	-2.329	4.457
O	3.689	-0.964	4.539
O	2.422	-4.720	-2.522
O	4.311	-3.807	-1.410
O	5.053	-1.624	2.715
C	3.392	-4.886	-1.495
C	4.360	-2.033	3.884
O	-2.178	-5.087	-1.513
C	-2.603	-5.381	-0.190
O	-3.568	-4.461	0.305
C	-1.606	-2.593	5.181
C	3.743	1.971	1.946
C	-2.502	1.428	3.404
C	3.047	3.029	1.065
C	-3.326	0.139	-2.136
C	-2.573	2.495	2.292
C	2.082	0.436	-3.770
C	2.792	1.039	-5.008
C	-4.215	0.776	-3.225
C	-3.392	3.646	2.909
C	3.961	4.267	1.161
N	1.928	1.858	-5.885
N	-3.674	4.793	2.014
N	3.529	5.455	0.399
N	-3.623	2.025	-3.753
C	-4.073	2.732	-4.793
C	2.359	2.397	-7.037
C	4.064	6.671	0.577
C	-4.711	5.631	2.131
N	-3.378	3.796	-5.229
N	3.617	2.216	-7.459
N	-4.613	6.894	1.667
N	5.061	6.878	1.445
N	3.587	7.721	-0.123
N	-5.880	5.239	2.651
N	-5.194	2.381	-5.441
H	0.201	-5.934	-2.028
H	-0.156	-0.990	-1.881
H	3.137	-2.738	-2.788
H	-3.222	-3.253	-1.420
H	2.270	0.178	-0.445
H	5.290	-3.499	0.958
H	-1.940	-0.187	0.505

H	-3.490	-4.252	2.881
H	-2.192	-0.305	4.330
H	4.133	0.271	2.905
H	0.492	1.014	1.920
H	1.631	-1.790	5.851
H	-4.465	-1.653	-1.953
H	-3.239	-1.727	-3.208
H	2.523	-1.507	-4.471
H	3.823	-0.777	-3.553
H	3.983	-5.751	-1.794
H	2.899	-5.046	-0.529
H	3.654	-2.838	3.648
H	5.129	-2.365	4.579
H	-1.741	-5.429	0.486
H	-3.118	-6.338	-0.255
H	-1.928	-3.045	6.118
H	-0.956	-3.265	4.609
H	4.770	1.883	1.569
H	3.835	2.417	2.945
H	-2.087	1.918	4.294
H	-3.545	1.196	3.658
H	2.040	3.267	1.428
H	2.967	2.695	0.022
H	-2.302	0.480	-2.319
H	-3.624	0.517	-1.151
H	-3.080	2.109	1.400
H	-1.578	2.848	1.991
H	1.002	0.327	-3.948
H	2.209	1.097	-2.907
H	3.194	0.205	-5.598
H	3.638	1.660	-4.696
H	-5.204	0.997	-2.799
H	-4.350	0.087	-4.066
H	-2.878	4.025	3.800
H	-4.347	3.241	3.249
H	4.967	3.978	0.829
H	4.029	4.570	2.210
H	0.930	1.716	-5.804
H	-2.884	5.154	1.495
H	3.073	5.299	-0.490
H	-2.975	2.506	-3.142
H	-3.798	4.500	-5.818
H	3.961	2.725	-8.262
H	-3.719	7.363	1.665
H	5.653	6.129	1.774
H	2.634	7.719	-0.454
H	4.084	8.600	-0.147
H	-6.079	4.265	2.823
H	-6.547	5.908	3.010
H	0.706	3.582	-7.370
H	1.748	3.390	-8.735
H	-5.918	1.852	-4.978
H	-5.449	2.858	-6.294
H	5.330	7.821	1.693
H	-2.402	3.906	-4.991
H	-5.433	7.485	1.631
H	4.161	1.419	-7.160
N	1.538	3.175	-7.770

Table S8. Cartesian coordinates of **2** Confo. II

Atom	x	y	z
C	-3.850	0.215	3.999
C	-1.415	0.142	2.663
C	-3.240	1.398	3.596
C	-3.241	-1.005	3.724
C	-1.999	-1.070	3.061
C	-1.998	1.391	2.929
C	-1.469	2.748	2.469
C	-1.471	-2.469	2.748
C	-1.997	3.062	1.070
C	-3.237	3.598	-1.397
C	-3.238	3.726	1.005
C	-1.413	2.663	-0.142
C	-1.997	2.930	-1.390
C	-3.847	4.002	-0.214
C	-1.999	-2.929	1.390
C	-3.240	-3.725	-1.006
C	-1.415	-2.663	0.142
C	-3.241	-3.596	1.396
C	-3.850	-4.000	0.213
C	-1.998	-3.061	-1.071
C	-1.470	-2.748	-2.469
C	-1.468	2.469	-2.748
C	-1.997	1.070	-3.061
C	-3.238	-1.396	-3.597
C	-3.238	1.006	-3.726
C	-1.414	-0.142	-2.663
C	-1.998	-1.390	-2.930
C	-3.848	-0.213	-4.001
C	-0.015	-2.821	3.139
C	-0.013	3.138	2.821
O	-3.864	-2.585	-3.924
O	-3.864	-4.176	-2.154
O	-3.863	2.154	-4.177
O	-3.864	2.587	3.923
O	-3.863	4.178	2.153
O	-3.862	3.924	-2.587
C	-4.621	3.258	2.925
C	-4.620	2.927	-3.257
O	-3.866	-2.152	4.176
C	-4.624	-2.924	3.255
O	-3.867	-3.922	2.585
C	-4.621	-3.256	-2.927
C	-0.012	2.820	-3.139
C	-0.013	-3.139	-2.820
C	1.231	2.212	-2.466
C	1.228	-2.214	2.467
C	1.230	-2.467	-2.212
C	1.230	2.466	2.213
C	2.467	3.138	2.819
C	2.465	-2.818	3.141
C	2.467	-3.141	-2.816
C	2.468	2.818	-3.140

N	3.727	2.574	2.305
N	3.727	-2.576	-2.303
N	3.728	2.304	-2.574
N	3.724	-2.306	2.575
C	4.937	-2.672	2.996
C	4.940	2.996	2.670
C	4.941	2.670	-2.996
C	4.939	-2.999	-2.667
N	6.027	-2.141	2.417
N	5.077	3.988	3.558
N	6.029	-2.421	-2.136
N	5.078	3.558	-3.988
N	6.030	2.140	-2.417
N	5.077	-3.991	-3.554
N	5.075	-3.560	3.988
H	-4.792	0.244	4.536
H	-0.497	0.111	2.085
H	-2.011	3.453	3.103
H	-2.014	-3.103	3.453
H	-0.495	2.085	-0.111
H	-4.789	4.539	-0.243
H	-0.497	-2.085	0.112
H	-4.792	-4.536	0.241
H	-2.011	-3.453	-3.103
H	-2.010	3.103	-3.453
H	-0.495	-0.112	-2.085
H	-4.789	-0.241	-4.538
H	0.061	-3.911	3.040
H	0.060	-2.609	4.214
H	0.064	3.037	3.911
H	0.063	4.213	2.611
H	-5.342	3.865	3.470
H	-5.117	2.531	2.271
H	-5.116	2.273	-2.530
H	-5.341	3.472	-3.864
H	-5.119	-2.271	2.527
H	-5.345	-3.469	3.861
H	-5.342	-3.862	-3.473
H	-5.118	-2.529	-2.274
H	0.065	3.911	-3.039

H	0.064	2.609	-4.213
H	0.064	-3.039	-3.911
H	0.062	-4.213	-2.610
H	1.251	1.123	-2.583
H	1.253	2.441	-1.395
H	1.248	-1.124	2.583
H	1.251	-2.443	1.396
H	1.249	-2.584	-1.122
H	1.253	-1.396	-2.440
H	1.252	1.394	2.439
H	1.251	2.585	1.123
H	2.443	3.020	3.910
H	2.441	4.212	2.592
H	2.441	-3.909	3.026
H	2.439	-2.588	4.214
H	2.444	-3.024	-3.907
H	2.440	-4.214	-2.588
H	2.444	3.908	-3.023
H	2.442	2.589	-4.213
H	3.687	1.814	1.634
H	3.686	-1.816	-1.632
H	3.688	1.632	-1.816
H	3.684	-1.634	1.817
H	6.954	-2.409	2.707
H	5.986	4.326	3.836
H	5.923	-1.666	-1.466
H	4.277	3.973	-4.438
H	5.925	1.469	-1.663
H	6.958	2.407	-2.708
H	4.275	-4.441	-3.970
H	5.986	-4.328	-3.834
H	5.924	1.665	1.467
H	6.957	2.707	2.408
H	4.274	-3.975	4.437
H	5.984	-3.841	4.322
H	5.987	3.840	-4.323
H	5.921	-1.469	1.664
H	6.956	-2.710	-2.405
H	4.276	4.436	3.975
N	6.029	2.420	2.137

Molecular Dynamics Simulation details

For MD simulations, the hosts and the ions (Na^+, Cl^-, ClO_4^-) were modelled using the generalized Amber force field (GAFF).[20] The ion partial charges were derived from AM1-BCC calculations,[21] and the water molecules were described using the TIP4P-Ew model.[22] Host topology files were provided. Both hosts were assigned a +4 charge. Each simulation encompassed a single host conformation along with 30 anions (Cl^-, ClO_4^-) immersed in a solution containing 5000 water molecules. The system was brought to neutralized by 26 Na^+ ions. Constraints were applied to all non-hydrogen atoms of the host. The simulations were conducted under isothermal-isobaric conditions, maintaining temperature using the Nosé-Hoover thermostat[23, 24] and pressure using the Parrinello-Rahman barostat.[25] The equations of motion were numerically integrated using a leapfrog algorithm with a time step of 2 femtoseconds. Electrostatic interactions were computed using the particle mesh Ewald summation method with a real space cutoff of 9 Å.[26] The simulations were run for a duration of 500 nanoseconds in a cubic simulation box with periodic boundary conditions.

Spatial distribution function

The interaction potentials between the anions (A) and the atoms of the host (H) were defined by the equation:

$$\psi(r_A,r_H)= \frac{q_A q_H}{r_{AH}} + 4\varepsilon_{AH}\left[\left(\frac{\sigma_{AH}}{r_{AH}}\right)^{12} - \left(\frac{\sigma_{AH}}{r_{AH}}\right)^{6}\right]$$

Here, the off-diagonal Lennard-Jones parameter σ_{AH} and the energy term ε_{AH} were determined using the combination rules:

$$\sigma_{AH}=\frac{(\sigma_A+\sigma_H)}{2}$$
$$\varepsilon_{AH}=\sqrt{\varepsilon_A\varepsilon_H}$$

Subsequently, the trajectories generated from these simulations were extracted and transformed into spatial distribution functions using the TRAVIS software.[27] Visualization of these results was carried out using ChimeraX.[28]

Dipole Moment Calculation

To calculate the dipole moment of the cavitand framework, the four pendent groups were truncated to methyl groups. The truncated model was optimized at the B3LYP/6-31G(d,p) level. The dipole moment extracted along the *z*-axis of the calculation was 0.9263 Debye.

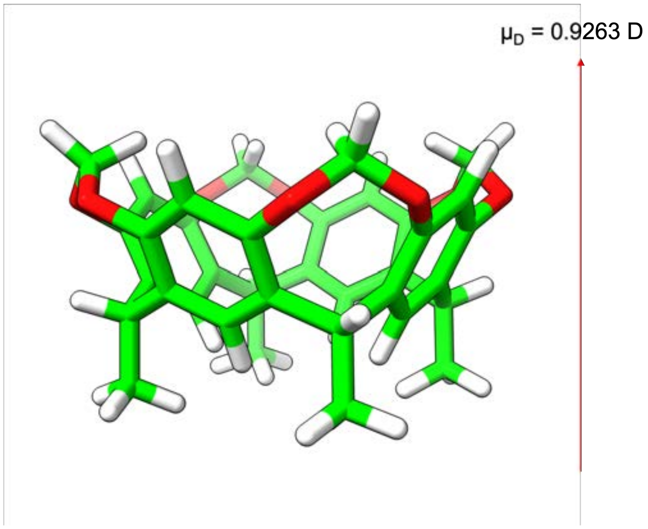

Figure S102. Structure and dipole of the methyl-footed cavitand.

6. References

(1) Gibb, C. L.; Gibb, B. C. Well-defined, organic nanoenvironments in water: the hydrophobic effect drives a capsular assembly. *J Am Chem Soc* **2004**, *126* (37), 11408-11409. DOI: 10.1021/ja0475611.

(2) Liu, S.; Whisenhunt-Ioup, S. E.; Gibb, C. L.; Gibb, B. C. An improved synthesis of 'octa-acid' deep-cavity cavitand. *Supramol Chem* **2011**, *23* (6), 480-485. DOI: 10.1080/10610278.2010.550290.

(3) Gibb, B. C.; Chapman, R. G.; Sherman, J. C. Synthesis of Hydroxyl-Footed Cavitands. *J. Org. Chem.* **1996**, *61* (4), 1505-1509, 10.1021/JO951633C. DOI: 10.1021/JO951633C.

(4) Hillyer, M. B.; Gibb, C. L.; Sokkalingam, P.; Jordan, J. H.; Ioup, S. E.; Gibb, B. C. Synthesis of Water-Soluble Deep-Cavity Cavitands. *Org Lett* **2016**, *18* (16), 4048-4051. DOI: 10.1021/acs.orglett.6b01903.

(5) Tancini, F.; Gottschalk, T.; Schweizer, W. B.; Diederich, F.; Dalcanale, E. Ion-pair complexation with a cavitand receptor. *Chem. - Eur. J.* **2010**, *16* (26), 7813-7819, S7813/7811-S7813/7815, 10.1002/chem.201000573. DOI: 10.1002/chem.201000573.

(6) http://supramolecular.org/ (accessed 01/06/2017).

(7) Turnbull, W. B.; Daranas, A. H. On the Value of c: Can Low Affinity Systems Be Studied by Isothermal Titration Calorimetry? *J. Am. Chem. Soc.* **2003**, *125* (48), 14859-14866, 10.1021/ja036166s. DOI: 10.1021/ja036166s.

(8) Tellinghuisen, J. Isothermal titration calorimetry at very low c. *Anal. Biochem.* **2008**, *373* (2), 395-397, 10.1016/j.ab.2007.08.039. DOI: 10.1016/j.ab.2007.08.039.

(9) Sokkalingam, P.; Shraberg, J.; Rick, S. W.; Gibb, B. C. Binding Hydrated Anions with Hydrophobic Pockets. *J. Am. Chem. Soc.* **2016**, *138* (1), 48-51, 10.1021/jacs.5b10937. DOI: 10.1021/jacs.5b10937.

(10) Sullivan, M. R.; Sokkalingam, P.; Nguyen, T.; Donahue, J. P.; Gibb, B. C. Binding of carboxylate and trimethylammonium salts to octa-acid and TEMOA deep-cavity cavitands. *J. Comput. Aided Mol. Des.* **2017**, *31* (1), 21-28. DOI: 10.1007/s10822-016-9925-0.

(11) Jordan, J. H.; Gibb, C. L. D.; Wishard, A.; Pham, T.; Gibb, B. C. Ion-Hydrocarbon and/or Ion-Ion Interactions: Direct and Reverse Hofmeister Effects in a Synthetic Host. *J. Am. Chem. Soc.* **2018**, *140* (11), 4092-4099. DOI: 10.1021/jacs.8b00196.

(12) *APEX4 and SAINT*; Bruker AXS LLS, Madison, WI: 2020. (accessed.

(13) University of Göttingen, Göttingen, Germany.: 2009. (accessed.

(14) Krause, L.; Herbst-Irmer, R.; G.M., S.; D., S. Comparison of silver and molybdenum microfocus X-ray sources for single-crystal structure determination. *J. Appl. Crystallogr.* **2015**, *48*, 3-10.

(15) Sheldrick, G. M. SHELXT - Integrated space-group and crystal-structure determination. *Acta Cryst.* **2915**, *A71*, 3–8.

(16) Lübben, J.; Wandke, C. M.; Hübschle, C. B.; Ruf, M.; Sheldrick, G. M.; Dittrich, B. Aspherical scattering factors for SHELXL – model, implementation and application. *Acta Cryst.* **2019**, *A75*, 50–62.

(17) Spek, A. L. PLATON SQUEEZE: a tool for the calculation of the disordered solvent contribution to the calculated structure factors. *Acta Cryst.* **2015**, *C71*, 9–18.

(18) Abraham, M. J.; Murtola, T.; Schulz, R.; Páll, S.; Smith, J. C.; Hess, B.; Lindahl, E. GROMACS: High performance molecular simulations through multi-level parallelism from laptops to supercomputers. *SoftwareX* **2015**, *1-2*, 19-25. DOI: 10.1016/j.softx.2015.06.001.

(19) Páll, S.; Abraham, M. J.; Kutzner, C.; Hess, B.; Lindahl, E. Tackling Exascale Software Challenges in Molecular Dynamics Simulations with GROMACS. In *Solving Software Challenges for Exascale*, Lecture Notes in Computer Science, 2015; pp 3-27.

(20) Siu, S. W.; Pluhackova, K.; Bockmann, R. A. Optimization of the OPLS-AA Force Field for Long Hydrocarbons. *J. Chem. Theory Comput.* **2012**, *8* (4), 1459-1470. DOI: 10.1021/ct200908r.

(21) Wang, J.; Wolf, R. M.; Caldwell, J. W.; Kollman, P. A.; Case, D. A. Development and testing of a general amber force field. *J. Comput. Chem.* **2004**, *25* (9), 1157-1174. DOI: 10.1002/jcc.20035.

(22) Horn, H. W.; Swope, W. C.; Pitera, J. W.; Madura, J. D.; Dick, T. J.; Hura, G. L.; Head-Gordon, T. Development of an improved four-site water model for biomolecular simulations: TIP4P-Ew. *J. Chem. Phys.* **2004**, *120* (20), 9665-9678. DOI: 10.1063/1.1683075.

(23) Nosé, S. A unified formulation of the constant temperature molecular dynamics methods. *J. Chem. Phys.* **1984**, *81* (1), 511-519. DOI: 10.1063/1.447334.

(24) Hoover, W. G. Canonical dynamics: Equilibrium phase-space distributions. *Phys. Rev. A: At., Mol., Opt. Phys.* **1985**, *31* (3), 1695-1697. DOI: 10.1103/physreva.31.1695.

(25) Parrinello, M.; Rahman, A. Polymorphic Transitions in Single-Crystals - a New Molecular-Dynamics Method. *Journal of Applied Physics* **1981**, *52* (12), 7182-7190. DOI: Doi 10.1063/1.328693.

(26) Darden, T.; York, D.; Pedersen, L. Particle mesh Ewald: AnN·log(N) method for Ewald sums in large systems. *J. Chem. Phys.* **1993**, *98* (12), 10089-10092. DOI: 10.1063/1.464397.

(27) Brehm, M.; Thomas, M.; Gehrke, S.; Kirchner, B. TRAVIS—A free analyzer for trajectories from molecular simulation. *The Journal of Chemical Physics* **2020**, *152* (16), 164105. DOI: 10.1063/5.0005078 (acccessed 8/15/2023).

(28) Pettersen, E. F.; Goddard, T. D.; Huang, C. C.; Meng, E. C.; Couch, G. S.; Croll, T. I.; Morris, J. H.; Ferrin, T. E. UCSF ChimeraX: Structure visualization for researchers, educators, and developers. *Protein Sci* **2021**, *30* (1), 70-82. DOI: 10.1002/pro.3943 From NLM.
